# Oxidative Photocyclization of Aromatic Schiff Bases in Synthesis of Phenanthridines and Other Aza-PAHs †

**DOI:** 10.3390/ijms21165868

**Published:** 2020-08-15

**Authors:** Martin Kos, Jaroslav Žádný, Jan Storch, Vladimír Církva, Petra Cuřínová, Jan Sýkora, Ivana Císařová, Febin Kuriakose, Igor. V. Alabugin

**Affiliations:** 1 Department of Advanced Materials and Organic Synthesis, Institute of Chemical Process Fundamentals of the Czech Academy of Sciences, v. v. i., Rozvojová 135, 165 02 Prague 6, Czech Republic; kos@icpf.cas.cz (M.K.); zadny@icpf.cas.cz (J.Ž.); storchj@icpf.cas.cz (J.S.); 2 Department of Analytical Chemistry, Institute of Chemical Process Fundamentals of the Czech Academy of Sciences, v. v. i., Rozvojová 135, 165 02 Prague 6, Czech Republic; curinova@icpf.cas.cz; 3 Department of Inorganic Chemistry, Faculty of Science, Charles University in Prague, Hlavova 2030, 128 40 Prague 2, Czech Republic; cisarova@natur.cuni.cz; 4 Department of Chemistry and Biochemistry, Florida State University, Tallahassee, FL 32306, USA; sykora@icpf.cas.cz

**Keywords:** imines, Schiff bases, photocyclization, phenanthridines, azahelicenes

## Abstract

The oxidative photocyclization of aromatic Schiff bases was investigated as a potential method for synthesis of phenanthridine derivatives, biologically active compounds with medical applications. Although it is possible to prepare the desired phenanthridines using such an approach, the reaction has to be performed in the presence of acid and TEMPO to increase reaction rate and yield. The reaction kinetics was studied on a series of substituted imines covering the range from electron-withdrawing to electron-donating substituents. It was found that imines with electron-withdrawing substituents react one order of magnitude faster than imines bearing electron-donating groups. The ^1^H NMR monitoring of the reaction course showed that a significant part of the *Z* isomer in the reaction is transformed into *E* isomer which is more prone to photocyclization. The portion of the *Z* isomer transformed showed a linear correlation to the Hammett substituent constants. The reaction scope was expanded towards synthesis of larger aromatic systems, namely to the synthesis of strained aromatic systems, e.g., helicenes. In this respect, it was found that the scope of oxidative photocyclization of aromatic imines is limited to the formation of no more than five *ortho*-fused aromatic rings.

## 1. Introduction

Phenanthridines consist of three nitrogen-containing, *ortho*-fused benzene rings. This structural motif is abundant in many natural products and medicinally relevant biologically active compounds [1,2,3,4,5,6]. Since substitution of phenanthridines is an effective strategy to modify biological activity [7], numerous synthetic methods for preparation of substituted phenanthridines have been developed over the last decade. The known synthetic procedures are based on visible light photocatalysis [8,9], anionic ring closure [10], radical routes [11,12], oxidative cyclization of 2-isocyanobiphenyls [13,14,15,16,17,18], Pictet-Spengler reaction [19,20], nitrogenation of 2-acetylbiphenyls [21], aryl halide/C-H intramolecular coupling [22,23], and C(sp^3^)-H amination [24,25]. Phenanthridines can also be prepared by oxidative photocyclization of stilbene-like molecules similarly to other polyaromatic hydrocarbons like e.g., helicenes. The method, originally developed by Mallory [26], consists of UV-light-promoted *E/Z* isomerization, subsequent cyclization of excited ^1^π,π* *E* isomer, and oxidation of the dihydrophenanthrene intermediate by either oxygen or iodine. Unlike the photocyclization of stilbenes, the transformation of nitrogen analogues containing an imine moiety instead of a carbon-carbon double bond is not very well established in the literature. The early efforts to achieve photocyclization of *N*-benzylidene aniline to phenanthridine failed [27]. This failure was originally explained by an extremely low concentration of *Z*-isomer in photochemical equilibrium at room temperature. A two percent yield was achieved at −10 °C, where the rate of the thermal *E/Z* isomerization is decreased [28]. Another factor that disfavors the photocyclization is the presence of an electron lone pair on nitrogen. The lowest singlet excited state of imines was suggested to be ^1^n,π*. [29] Therefore, a cyclization from the ^1^π,π* state has to compete with rapid internal conversion to the lower energy inactive n,π* state. This suggestion is supported by the fact that *N*-benzylideneaniline [30] and other imines readily cyclize to the corresponding phenanthridines in the presence of strong acid such as e.g., conc. H_2_SO_4_ [31,32,33]. In this case, the nitrogen lone pair is protonated and ^1^π,π* is the lowest energy state. However, these methods suffer from low reaction yields and formation of undesired side products.

Underreted by the possible drawbacks, we decided to examine the photochemical approach in the synthesis of aromatic systems containing a pyridine unit with a perspective to the synthesis of large aza-PAHs, namely aza[n]helicenes. Herein we report a detailed study of the photochemical synthesis of variously substituted 6-phenylphenathridines by oxidative photocyclization of *N*,1,1-triarylmethanimines available via a two-step procedure from inexpensive Schiff bases. The optimized reaction conditions were also tested for the preparation of aza-PAHs with more fused benzene rings, which can be utilized in material chemistry [34,35,36].

## 2. Results and Discussion

**Scheme 1:**
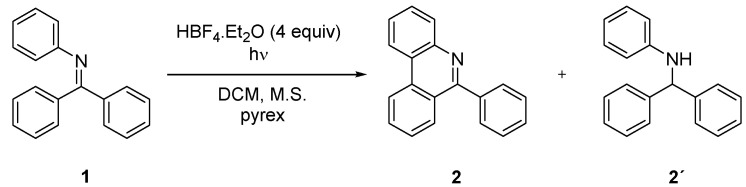
Photochemical synthesis of 6-phenylphenanthridine 2.

A possibility of photochemical synthesis of variously substituted phenanthridines from corresponding imines was explored. *N*-phenylbenzophenone imine (**1**) was selected as a model compound for optimization of the photocyclization reaction conditions. Irradiation of 322 mg of this imine for 67 h in 250 mL of cyclohexane using a 400-watt mercury lamp provided 6-phenylphenanthridine (**2**) in 46% isolated yield [28]. According to the literature [31,32,33], the reaction on similar substrates can be accelerated via protonation of starting imine by addition of acid. Indeed, according to GC-MS analysis, a full conversion and promising 35% yield was achieved using 100 mg of **1** in the presence of 4 equivalents of HBF_4_·Et_2_O within 4 h according to GC-MS analysis. However, beside the desired phenanthridine **2**, a significant amount of *N*-benzyhydrylaniline (**2’**) was formed by partial reduction of starting imine 1 (Scheme 1). The formation of **2’** can be prevented by addition of TEMPO similarly to oxidative photocyclization of stilbene [37]. Two equivalents of TEMPO led to an increase in isolated yield of **2** to 71% in chlorinated solvents. Iodine was also tested as an oxidant; however, the imine reduction was not fully suppressed. Hence, TEMPO was used as an additive in all subsequent studies.

**Scheme 2:**
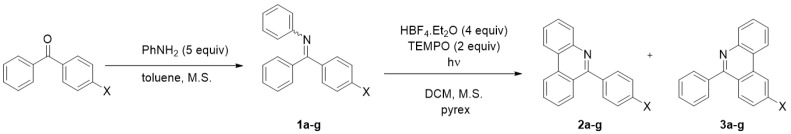
Photocyclization of imines **1a-g**.

The optimized reaction conditions were used in the study of the photocyclization regioselectivity using substituted arylimines. A series of diversely substituted imines **1a**-**g** covering both the electron-donating and electron-withdrawing groups was prepared from commercially available ketones (Scheme 2). All obtained imines **1a**-**g** were obtained as a mixture of (*E*)- and (*Z*)- isomers. The isolation of individual imine isomers was not attempted due to their facile hydrolysis. Where possible, the *E*/*Z* ratio was evaluated tentatively by NMR spectroscopy. Subsequent protonation of imines by HBF_4_.Et_2_O changes the *E*/*Z* ratio only negligibly. A final photocyclization affords a mixture of regioisomeric cyclization products in good yields with modest regioselectivity in most cases, generally preferring reaction at the phenyl ring (**2a**-**g**) over the substituted aryl (**3a**-**g**); see Table 1. 

Obviously, the observed regioselectivity does not correlate with the donor/acceptor properties of the substituents. Therefore, a detailed analysis of reaction conditions and the species involved was conducted with special emphasis on isomerization of the starting imines prior to and during cyclization, as the isomer ratio during the course of the reaction has to be reflected in the ratio of the final products.

The isomerization rates of starting imines were found negligible at ambient temperature. A variable temperature ^1^H NMR experiment performed with -OMe (**1a**) and -NO_2_ (**1g**) derivatives covering both ends of the Hammett series showed the coalescence slightly above 100 °C. The Gibbs energy of isomerization was found to be 18.6 and 19.3 kcal·mol^−1^ for -OMe and -NO_2_ derivative, respectively. On the contrary, the protonated imines did not show any signs of isomerization within ^1^H NMR accessible temperature range. Therefore, the protonation preserves the *E*/*Z* ratio prior to the photoreaction itself.

**Figure 1:**
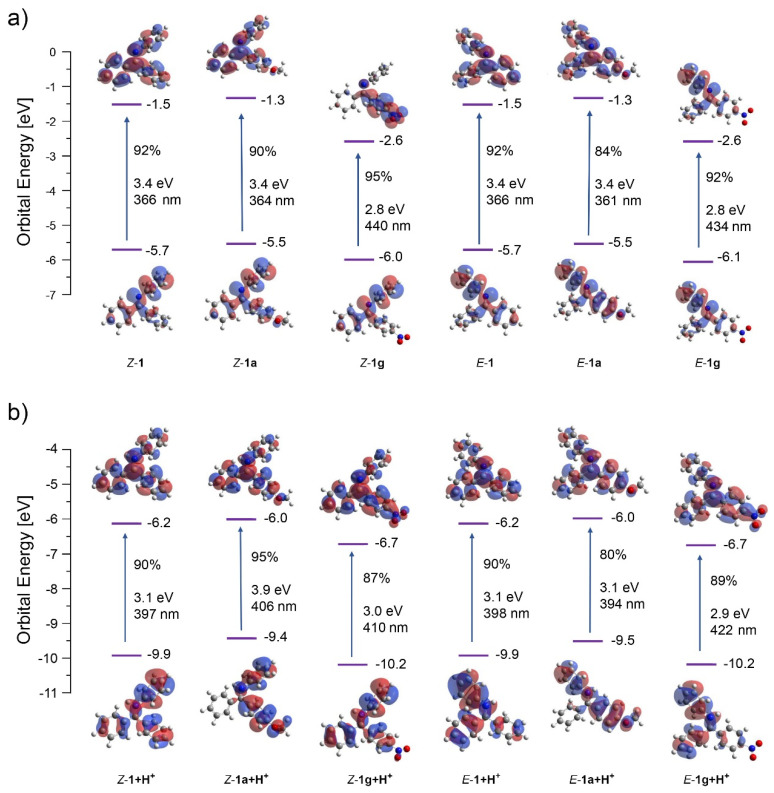
Orbital energies, excitation wavelengths, and dominating orbital contributions to the excited states for *E* and *Z* isomers of neutral (**a**) and protonated versions (**b**) of reactants **1**, **1a**, and **1g**. *E* and *Z* isomers are identical for **1** but are shown for the illustrative purposes.

TD-DFT calculations of starting imines in excited state provided some insight into the nature and energy of molecular orbitals of the species prior to cyclization (Figure 1). The excitation was generally dominated by the HOMO-LUMO transition (80–95% contribution). The HOMO and LUMO for the neutral and protonated imines are highly delocalized as expected for these conjugated molecules. In agreement to the literature, the lowest singlet excited states can be described as the ^1^n,π* transitions for the neutral imines and ^1^π,π* transitions for their protonated counterparts. The MO energies (including both HOMO and LUMO) are significantly lowered upon protonation. In all cases, the symmetry of the LUMO was optimal for conrotatory cyclization. Significant LUMO orbital coefficients at the reacting sites may contribute to the increased efficiency of the cyclization under acidic conditions. However, the energies of *E* and *Z* isomers are rather similar for both –OMe (**1a**) and –NO_2_ (**1g**) substituents indicating that the observed regioselectivity results from the different reactivity of their respective excited states as they evolve at the excited state surfaces after the initial excitation step.

**Figure 2:**
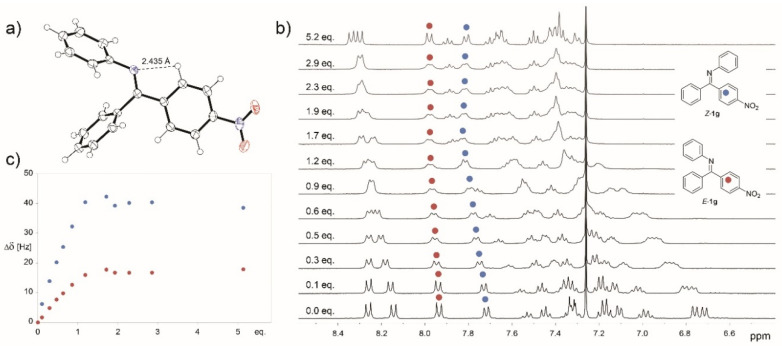
ORTEP projection of the crystal structure of *E*-**1g** (**a**), ^1^H NMR titration of **1g** with HBF_4_ (**b**), and the dependence of Δδ on the HBF_4_ equivalents added (**c**).

X-ray structure of *E*-**1g** indicated some involvement of nitrogen lone pair in the stabilization of *E* isomer via intramolecular hydrogen bonding (Figure 2a). This stabilization could prevent protonation of *E* isomers especially of imines bearing electron-withdrawing groups. However, the ^1^H NMR titration of starting imines with HBF_4_ did not show significant differences among the compounds studied (Figure 2b). The imines with electron-donating and electron-withdrawing groups are fully protonated upon addition of 2 equivalents of the acid (Figure 2c).

**Figure 3:**
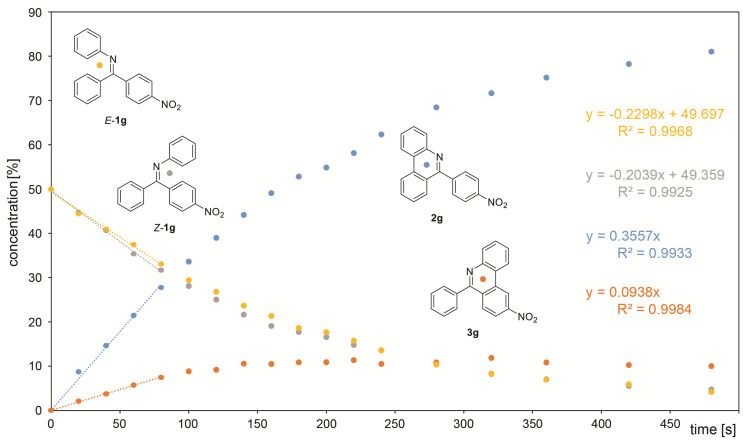
Monitoring of reaction kinetics of **1g**. The trendlines indicate a linear part of the dependence used for calculation of the reaction rates.

**Figure 4:**
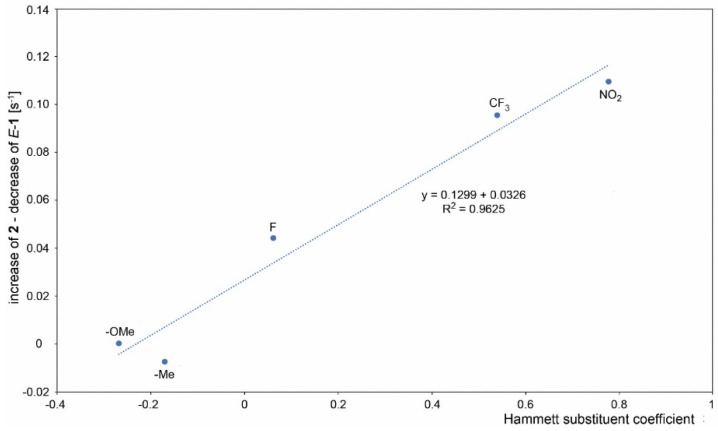
The contribution of *Z*-**1** to the formation of **2**, calculated as the rate of **2** formation minus a decrease of *E*-**1** concentration and its correlation to the Hammett para-substituent coefficient.

A course of the reaction was subsequently monitored by ^1^H or ^19^F NMR. For this purpose, the reaction was performed directly in an NMR tube and checked off-line at regular intervals. The differentiation of signals of individual species was rather difficult as the signals are concentrated in the aromatic region except for compounds containing fluorine and/or methyl groups. However, this approach enabled in all cases an observation of the gradual formation of both products. In some cases, it was even possible to distinguish signals of both isomers of the starting imine. In some cases, a presence of benzophenone formed by imine hydrolysis was observed. However, its concentration does not change during the reaction. UV-irradiation induced a change in the *E*/*Z* ratio of the starting imines in some cases. However, the photostationary state in the reaction mixture was usually established instantly. The only exception is **1c**, where it was achieved approximately within 1 min of UV irradiation. It was found that the photocyclization rate increases when moving from electron-donating groups to electron-withdrawing ones. The difference in the speed of product formation found between –OMe (**1a**) and –NO_2_ (**1g**) is one order of magnitude. It was also found that the products **2a**-**g** coming from imines with *E* configuration are formed faster than **3a**-**g** from *Z* imines and a significant part of the *Z* isomer is transformed upon irradiation to *E* isomer prior to cyclization. Therefore, the decrease of concentration of starting *Z* isomer is faster than the formation of corresponding product **3** and vice versa—the product **2** is formed faster than the corresponding decrease of the starting *E* imine. Due to the complexity of the reaction where the *E*/*Z* isomerization significantly affects the final **2**/**3** product ratio, the reaction kinetics cannot be fitted by first-order kinetics. Moreover, the gradual product formation also changes the UV absorption of the whole reaction mixture. For these reasons, the determination of the overall reaction rates was not attempted, and only the rates of individual product formation and the decrease of the starting imines were calculated from the initial linear part of the concentration dependence on reaction time (Figure 3). The obtained rates of individual product formation as well as concentration decrease of the starting imines are summarized in Table 2. The obtained values plotted against the Hammett coefficient indicates a linear correlation between the rate and the substituent. As mentioned above, the *Z* imine in the reaction can provide product **3** or can be transformed into *E* isomer which undergoes photocyclization to product **2**. The extent of this contribution can be calculated as the concentration of the *E* imine minus the concentration of product **2**. The plot of this contribution against the Hammett coefficient also provides a linear correlation showing that the extent of contribution of *Z*-**1** to the formation of **2** depends on the substitution (Figure 4).

**Scheme 3:**
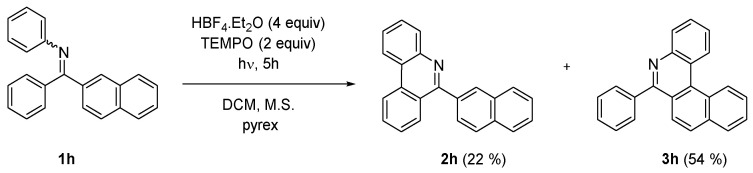
Photocyclization of naphthyl imine **1h**.

In order to expand the scope of the method towards synthesis of larger aromatic systems—namely helicenes—the competition of the photocyclization reaction at phenyl and naphthyl rings was examined in the reaction of naphthyl phenyl imine **1h** (Scheme 3). The reaction provided phenanthridine **2h** and 6-phenyl-5-aza [4] helicene (**3h**) in approximate 1:2 ratio indicating that the attack at naphthalene is slightly preferred.

**Scheme 4:**
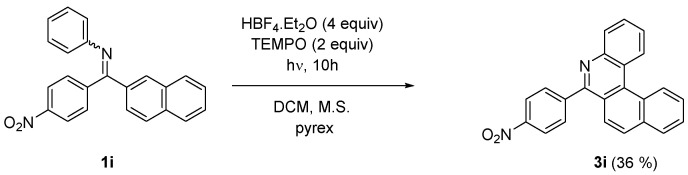
Photocyclization of naphthyl imine **1i**.

On the other hand, a similar reaction performed on naphthyl 4-nitrophenyl imine (**1i**) provided exclusively 6-(4-nitrophenyl)-5-aza [4] helicene (**3i**) (Scheme 4). The nitro substituent in the imine works as an auxiliary group enhancing the photocyclization of **1i** in *E* configuration into 6-aryl-5-aza [4] helicene **3i** as a single product.

**Figure 5:**
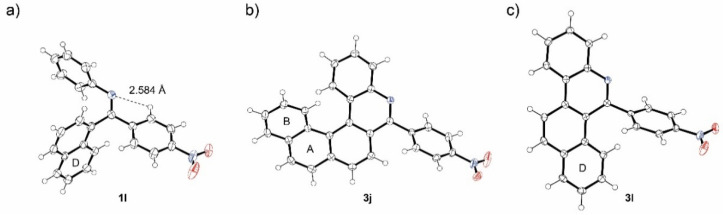
ORTEP projection of the crystal structure of **1l** (**a**), **3j** (**b**) and **3l** (**c**). Thermal ellipsoids are shown with 50% probability.

**Scheme 5:**
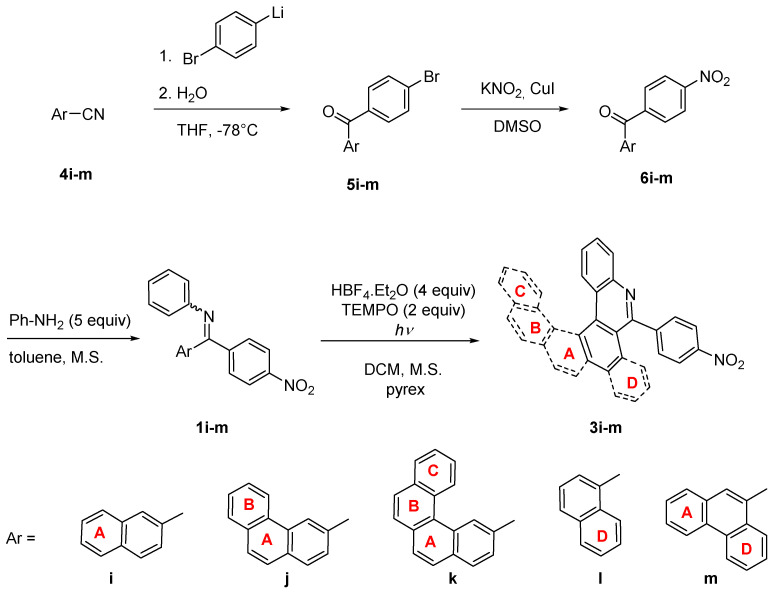
Synthesis of nitro-aza-PAHs **3i-m**.

High regioselectivity of photocyclization of nitro-imine **1i** indicates a general trend in imines using 4-nitrophenyl as a directing group. For confirmation, imines **1j-m** combining 4-nitrophenyl and aryl groups with up to four condensed rings were synthesized and tested in photocyclization (Scheme 5). Generally, the synthesis of imines **1i-m** starts from aryl-carbonitriles **4i**-**m** which are commercially available or can be prepared from the corresponding bromides [38] (for details see experimental part in SI). The reaction of nitriles **4i-m** with monolithiated 1,4-dibromobenzene furnished bromoaryl ketones **5i**-**m**. Bromoarenes **5i**-**m** are subsequently subjected to copper catalyzed *ipso*-nitration [39] providing nitroketones **6i**-**m**, which were transformed to corresponding imines. Surprisingly, the imines **1l** and **1m** were obtained almost exclusively as single isomers. Their *Z* configuration was deduced from NOESY experiments and confirmed by X-ray analysis in the case of **1l** (Figure 5a). The crystal structure of **1l** showed again a conformation stabilization by intramolecular hydrogen bond. In the last step, the nitro-imines **1l-m** were subjected to photocyclization (Table 3). No reaction with benzo[*c*]phenanthryl imine **1k** took place, only the unreacted starting material was recovered from the reaction mixture. In all other cases, the corresponding aza-PAHs **3l**-**m** were obtained and isolated in modest yields. No attack at the 4-nitrophenyl moiety was observed. The product structure was deduced from 2D-NMR spectra. In the case of **3j** and **3l**, suitable single crystals were obtained from the chloroform solution and the structures were confirmed by X-ray crystallography (Figure 5b,c).

**Scheme 6:**
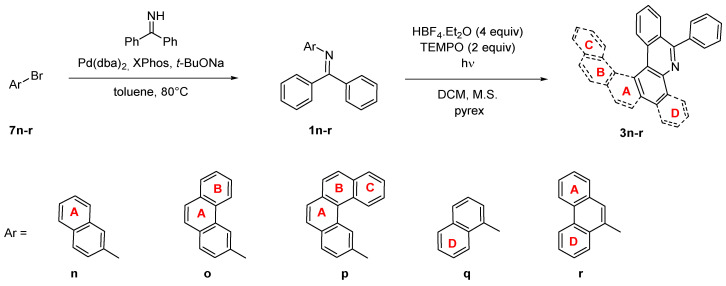
Synthesis of aza-PAHs **3n-r**.

Larger systems containing a nitrogen atom in the aromatic skeleton can also be prepared by photocyclization of imines with the arene at the nitrogen atom. The resulting aza-PAHs **3n**-**r** (Scheme 6)then contains nitrogen in a different position than products **3i**-**m**. The imines **1n**-**r** were prepared from benzophenone imine and corresponding bromoarenes **7n**-**r** by Buchwald-Hartwig amination [40]. Advantageously, the photocyclization of imines **1n**-**r** provides the same product of both arrangements, which is then reflected in higher reaction yields. On the other hand, the reaction rate is significantly decreased, similarly as observed for the reaction of unsubstituted imine **1** when compared to the nitro derivative **1g**. A full conversion was achieved for the imines **1n**,**o**,**r** (Table 4) In the case of **1q**, 40% conversion was achieved within 28 h, which is then reflected in decreased isolated yield. The irradiation of benzo[*c*]phenanthryl imine **1p** did not lead to the formation of product **3p**, similarly to **3k**. Obviously, the imines cannot provide via photocyclization highly strained aromatic systems such as [6]helicenes possessing six *ortho*-fused aromatic rings, contrary to cyclization of their carbo-analogues.

**Scheme 7:**
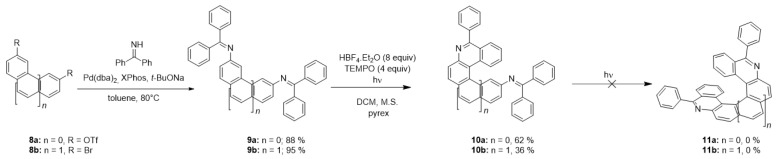
Synthesis of diaza-PAHs **11a,b**.

A sequential photocyclization of diimines could lead to the formation of helical diaza-PAHs, similarly to the widespread photochemical synthesis of helicenes [41,42] which often utilizes a double cyclization of two double stilbene-like bonds. For this purpose, diimines **9a**,**b** were synthesized from bis-triflate **8a** or dibromoarene **8b**, respectively, by coupling with benzophenone imine (Scheme 7). However, the irradiation of diimines **9a**,**b** led only to the formation of products of monocyclization **10a**,**b** (Table 5) containing four and five *ortho*-fused aromatic rings, respectively. A formation of the desired helicenes **11a**,**b** was not observed even after prolonged irradiation of **10a**,**b**. This fact is in accordance with the results obtained for photocyclization of imines **1k** and **1p** containing four condensed rings.

**Scheme 8:**
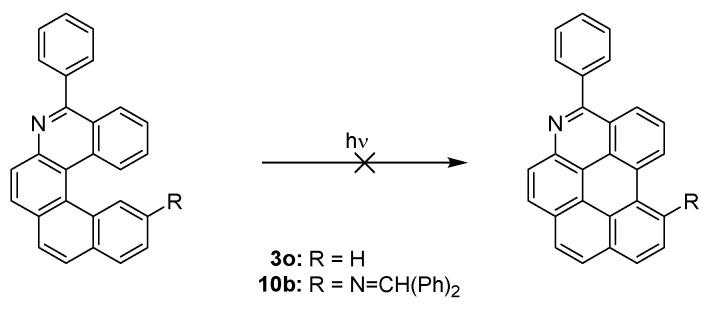
Possible overannulation of helicenes **3o** and **10b**.

It is noteworthy that formation of aza [5] helicenes **3j**, **3o**, and **10b** does not suffer from undesired overannulation—the formation of benzo[*ghi*]perylene derivative [43] (Scheme 8). In the case of **3j**, it is in agreement with the theoretical prediction [44] that substitution in position 5 and 10 should suppress overannulation.

**Figure 6:**
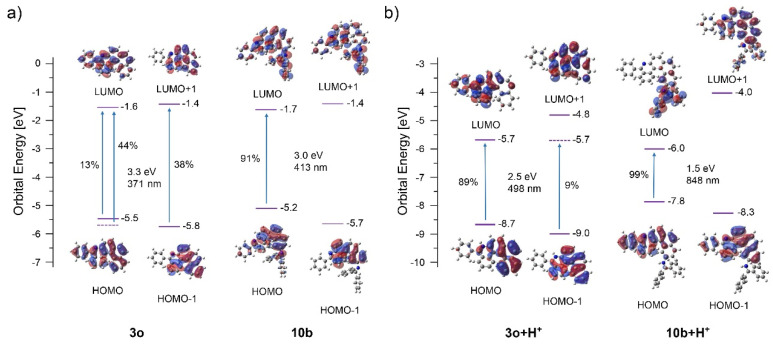
Orbital energies, excitation wavelengths, and dominating orbital contributions to the excited states of neutral (**a**) and protonated versions (**b**) of reactants **3o** and **10b**.

TD-DFT calculations were performed to evaluate the S_1_ nature for 6-aza [5] helicenes **3o** and **10b**. The calculations showed that the lowest excitation for neutral helicene **3o** is a superposition of three orbital transitions: HOMO to LUMO (13%), HOMO-1 to LUMO (44%) and HOMO to LUMO+1 (38%) (Figure 6). The symmetry of LUMO+1 is suitable for conrotatory cyclization (~C_2_ symmetric), but the weight of LUMO+1 in this excited state is low in comparison to the LUMO. In the protonated molecule **3o+H**^+^, the lowest excitation has two transitions: HOMO to LUMO (89%) and HOMO-1 to LUMO (9%). Again, the symmetry of LUMO is not suitable for conrotatory cyclization, potentially disfavouring overannulation. Unlike **3o**, the lowest excitation in **10b** consists of only transitions from HOMO to LUMO (91%) and the symmetry of LUMO is not appropriate for conrotatory cyclization. The same trend is also observed in protonated helicene-based imine **10b+H**^+^. In this case, the excitation is based entirely on the HOMO to LUMO (99%) transition with LUMO not suitable for the cyclization. These features may account for the absence of overannulation in **3o** and **10b**.

## 3. Conclusions

The oxidative photocyclization of aromatic Schiff bases is a suitable method for synthesis of phenanthridines, medicinally relevant biologically active compounds. However, the reaction has to be performed in the presence of acid, in order to increase the reaction rate. The presence of TEMPO increases the yield of desired phenanthridines by preventing formation of the hydrogenation products. The study conducted on a series of substituted imines covering the range from electron-withdrawing to electron-donating substituents indicated only a modest substituent effect on reaction regioselectivity. In all cases, the cyclization was preferred at the phenyl ring over the substituted aryl. However, the substituent significantly affects the reaction rate. The rates found for imines with electron-withdrawing substituents were one order of magnitude higher than those of electron-donating ones. The rates of product formation indicate a linear correlation to the Hammett substituent constants. A detailed reaction kinetics study also revealed that upon UV excitation a significant portion of the *Z* isomer is transformed to the *E* isomer which is more prone to the photocyclization. The amount transformed also showed a linear correlation to the Hammett substituent constants.

The scope of the reaction was also expanded towards synthesis of larger aromatic systems. It was found that electron-withdrawing substituents can significantly affect the reaction regioselectivity and the photocyclization provides exclusively products in *E* configuration, leaving the substituted aryl untouched. Regarding the preparation of strained aromatic systems such as helicenes, the reaction is restricted only to the formation of five *ortho*-fused aromatic rings, providing at most aza [5] helicenes. The formation of more strained aza [6] helicene was not achieved. On the other hand, the presence of a nitrogen atom in the aza [5] helicene moiety prevents undesired overannulation to benzo[*ghi*]perylene which is common to carbo [5] helicenes under UV irradiation. In general, the oxidative photocyclization of aromatic imines can be employed in the synthesis of aza-PAHs—valued compounds in material chemistry.

## 4. Materials and Methods

Commercially available reagent-grade materials were used as obtained from Sigma-Aldrich, Acros Organics, Apollo Scientific, and Fluorochem. Bromoarenes **7o [45]**, **7p [46]**, **8b [42]** and bis-triflate **8a [47]** were prepared according to published procedures. All solvents (Lach-Ner) were of a reagent grade and used without any further purification, except for tetrahydrofuran and toluene, which were distilled from sodium benzophenone ketyl, and dichloromethane, which was distilled from calcium hydride. Melting points were determined with Santiago KB T300 melting point apparatus (Czech Republic) and are uncorrected. TLC was carried out using silica gel 60 F254-coated aluminum sheets, and compounds were visualized with UV light (254 and 366 nm). Column chromatography was performed using Biotage HPFC systems (Isolera One) with prepacked flash silica gel columns. The standard Schlenk technique was used for all reactions. Microwave experiments were performed on an Anton Paar Monowave 300 equipped with simultaneous temperature measurement with an IR sensor. ^1^H, ^13^C{^1^H}, and ^19^F{^1^H} NMR spectra were recorded using a Bruker Avance and Varian Inova spectrometer at 400 and 500 MHz, respectively (^1^H NMR), 101 and 126 MHz, respectively (^13^C NMR), and 376 MHz (^19^F NMR). Chemical shifts (δ) are reported in parts per million (ppm) relative to TMS, and C_6_F_6_ (δ = −164.90 ppm), or referenced to residuals of CDCl_3_ (δ = 7.26 and 77.00 ppm, respectively). The coupling constants (*J*) are given in hertz (Hz) and corresponding multiplicity (s = singlet, d = doublet, t = triplet, m = multiplet). The NMR spectra and details on NMR titration, variable temperature measurement, and reation kinetics monitoring can be found in Supplementary Materials. The IR spectra were measured in CHCl_3_ (Nicolet 6700). Characteristic IR absorptions are reported in cm^−1^ and denoted as strong (s), medium (m), and weak (w). The low-resolution electron impact (EI) mass spectra were recorded on a Thermo Finnigan Focus DSQ mass spectrometer at an ionizing voltage of 70 eV in a positive mode, and the m/z values are given along with their relative intensities (%). Conversions were determined from uncorrected GC-MS chromatograms. For exact mass measurement, the spectra were internally calibrated using Na-formate or APCI-TOF tuning mix. ESI and APCI high-resolution mass spectra were measured in a positive mode using a micrOTOF QIII mass spectrometer (Bruker) and were determined by software Compass Data Analysis. Diffraction data of **1g**, **2g**, **1l**, **3j**, **3l** and **3r** were collected on a Bruker D8 VENTURE Kappa Duo PHOTON 100 CMOS with the monochromated Mo/Cu-Kα radiation. The structures were solved by direct methods (SHELXT [48]) and refined by full-matrix least-squares on F^2^ values (CRYSTALS [49]). All heavy atoms were refined anisotropically. Hydrogen atoms were usually localized from the expected geometry and difference electron density maps and were refined isotropically. ORTEP-3 [50] was used for structure presentation. The crystallographic data for the structures reported in this paper have been deposited with the Cambridge Crystallographic Data Centre as a supplementary publication; CCDC 2017712−2017717. Copies of the data can be obtained free of charge on application to CCDC, e-mail: deposit@ccdc.cam.ac.uk. For experimental procedures and data see Supplementary Materials. All DFT calculations were performed at Gaussian 09 software using the B3LYP DFT functional and the 6-31G(d) basis set. Frequency calculations were conducted for all structures, confirming that they correspond to a minimum. The Gibbs Free energy values are reported at 298 K. Time-dependent DFT calculations were performed the B3LYP/6-31G(d) level of theory. Molecules and orbitals were rendered with GaussView.

## Figures and Tables

**Table 1:** Photocyclization of imines **1a**-**g**—regioselectivities and yields.

Starting Material	X=	Reaction Time [h]	Ratio of Isomers ^a^	Ratio of Products ^b^ 2a-g:3a-g [%]	Sum of Isolated Yields [%]
**1a**	OMe	14	63:37	86:14	42 ^c^
**1b**	Me	8	52:48	63:37	80
**1**	H	4	-	-	71
**1c**	F	4	61:39	83:17	72
**1d**	Cl	4	63:37	73:27	67
**1e**	Br	5	56:44	74:26	70
**1f**	CF_3_	5	51:49	78:22	64
**1g**	NO_2_	4	52:48	90:10	58 ^c^

^a^ determined by ^1^H NMR, ^b^ determined by GC-MS, ^c^ the minor isomer was not isolated.

**Table 2:** Summary of photocyclization kinetics performed on the series of imines 1a-g.

Substituent	Hammett Substituent Constant	Rates						
		Decrease			Increase		Difference	
		Σ 1	*E*-1	Z-1	2	3	2—*E*-1	*Z*-1—3
**1a** (OMe)	−0.268	0.039	0.029	0.010	0.028	0.003	−0.001	0.007
**1b** (Me)	−0.170	0.117	0.077	0.040	0.072	0.044	−0.006	−0.004
**1** (H)	0	0.122	-	-	0.122	-	-	-
**1c** (F)	0.062	0.216	0.111	0.105	0.159	0.079	0.048	0.026
**1d** (Cl)	0.227	0.370	-	-	0.268	0.102	-	-
**1e** (Br)	0.232	0.516	-	-	0.380	0.136	-	-
**1f** (CF_3_)	0.540	0.534	0.307	0.227	0.417	0.120	0.110	0.107
**1g** (NO_2_)	0.778	0.434	0.230	0.204	0.356	0.094	0.126	0.110

**Table 3:** Photocyclization of nitro-imines **1i-m**.

Starting Material	Reaction Time [h]	Conversion ^a^ [%]	Isolated Yield 3i-m [%]
**1i**	10	100	36
**1j**	9	100	50
**1k**	13	0	0
**1l**	10	100	42
**1m**	10	100	40

^a^ Determined by GC-MS.

**Table 4:** Photocyclization of N-aryl imines **1n-r**.

Starting Material	Reaction Time [h]	Conversion ^a^ [%]	Isolated Yield 3n-r [%]
**1n**	8	100	64
**1o**	31	100	72
**1p**	22	0	0
**1q**	28	40	24
**1r**	15	100	72

^a^ Determined by GC-MS.

**Table 5:** Photocyclization of diimines **9a,b**.

Compound	Reaction Time [h]	Isolated Yield of 10a,b [%]	Isolated Yield of 11a,b [%]
**9a**	8	62	0
**9b**	40	36	0

## Supplementary Materials

Supplementary Materials can be found at https://www.mdpi.com/1422-0067/21/16/5868/s1.

## Abbreviations

APCI	Atmospheric pressure chemical ionization
dba	Dibenzylidenaceton
DCM	Dichloromethane
DMSO	Dimethyl sulfoxide
ESI	Electrospray ionization
GC-MS	Gas chromatography–mass spectrometry
HOMO	Highest occupied molecular orbital
IR	Infrared
LUMO	Lowest unoccupied molecular orbital
M.S.	Molecular sieves
NMP	N-Methyl-2-pyrrolidone
NMR	Nuclear magnetic resonance
NOESY	Nuclear Overhauser effect spectroscopy
ORTEP	Oak ridge thermal ellipsoid plot
PAH	Polycyclic aromatic hydrocarbon
PTFE	Polytetrafluoroethylene
TD-DFT	Time-dependent density functional theory
TEMPO	2,2,6,6-Tetramethyl-1-piperidinyloxy
THF	Tetrahydrofuran
TLC	Thin-layer chromatography
TMS	Tetramethylsilane
TOF	Time of flight
UV	Ultraviolet
XPhos	2-Dicyclohexylphosphino-2′,4′,6′-triisopropylbiphenyl
