# Supplementary material for: Oxidative Photocyclization of Aromatic Schiff Bases in Synthesis of Phenanthridines and Other Aza-PAHs
Source: Int J Mol Sci. 2020 Aug 15;21(16):5868. doi: 10.3390/ijms21165868 (PMC7461585; doi:10.3390/ijms21165868)
Supplement: Supplementary file 1 [file ijms-21-05868-s001.pdf]

International Journal of *Molecular Sciences*

MDPI

Article

Oxidative photocyclization of aromatic Schiff bases in synthesis of phenanthridines and other aza-PAHs

Martin Kos [1], Jaroslav Žádný [1], Jan Storch [1], Vladimír Církva [1*], Petra Cuřínová [2], Jan Sýkora [2*], Ivana Císařová [3], Febin Kuriakose [4], and Igor. V. Alabugin [4*]

1 Department of Advanced Materials and Organic Synthesis, Institute of Chemical Process Fundamentals of the Czech Academy of Sciences, v. v. i., Rozvojová 135, 165 02 Prague 6, Czech Republic; kos@icpf.cas.cz, zadny@icpf.cas.cz, storchj@icpf.cas.cz, cirkva@icpf.cas.cz
2 Department of Analytical Chemistry, Institute of Chemical Process Fundamentals of the Czech Academy of Sciences, v. v. i., Rozvojová 135, 165 02 Prague 6, Czech Republic; curinova@icpf.cas.cz, sykora@icpf.cas.cz
3 Department of Inorganic Chemistry, Faculty of Science, Charles University in Prague, Hlavova 2030, 128 40 Prague 2, Czech Republic; cisarova@natur.cuni.cz
4 Department of Chemistry and Biochemistry, Florida State University, Tallahassee, Florida 32306, USA; fk16@my.fsu.edu, alabugin@chem.fsu.edu
* Correspondence: cirkva@icpf.cas.cz; sykora@icfp.cas.cz; alabugin@chem.fsu.edu

Content

1. Experimental part S3
2. X-ray crystallographic studies S25
3. Variable temperature NMR experiments S31
4. NMR Kinetics measurements S32
5. DFT Calculations S37
6. References S60
7. Abbreviations S62
8. NMR Spectra S63

1. Experimental part

Commercially available reagent grade materials were used as obtained from Sigma-Aldrich, Acros Organics, Apollo Scientific, and Fluorochem. Bromoarenes **7o**[1], **7p**[2], **8b**[3] and bis-triflate **8a**[4] were prepared according to published procedures. All solvents (Lach-Ner) were of a reagent grade and used without any further purification, except for tetrahydrofuran and toluene, which were distilled from sodium benzophenone ketyl, and dichloromethane, which was distilled from calcium hydride. Melting points were determined with Santiago KB T300 melting point apparatus (Czech Republic) and are uncorrected. TLC was carried out using silica gel 60 F254-coated aluminum sheets, and compounds were visualized with UV light (254 and 366 nm). Column chromatography was performed using Biotage HPFC systems (Isolera One) with prepacked flash silica gel columns. The standard Schlenk technique was used for all reactions. Microwave experiments were performed on an Anton Paar Monowave 300 equipped with simultaneous temperature measurement with an IR sensor. ^{1}H, ^{13}C{^{1}H}, and ^{19}F{^{1}H} NMR spectra were recorded using a Bruker Avance and Varian Inova spectrometer at 400 and 500 MHz, respectively (^{1}H NMR), 101 and 126 MHz, respectively (^{13}C NMR), and 376 MHz (^{19}F NMR). Chemical shifts (δ) are reported in parts per million (ppm) relative to TMS, and C_6F_6 (δ = −164.90 ppm), or referenced to residuals of $CDCl_3$ (δ = 7.26 and 77.00 ppm, respectively). The coupling constants (*J*) are given in hertz (Hz) and corresponding multiplicity (s = singlet, d = doublet, t = triplet, m = multiplet). The IR spectra were measured in $CHCl_3$ (Nicolet 6700). Characteristic IR absorptions are reported in cm^{-1} and denoted as strong (s), medium (m), and weak (w). The low-resolution electron impact (EI) mass spectra were recorded on a Thermo Finnigan Focus DSQ mass spectrometer at an ionizing voltage of 70 eV in a positive mode, and the m/z values are given along with their relative intensities (%). Conversions were determined from uncorrected GC-MS chromatograms. For exact mass measurement, the spectra were internally calibrated using Na-formate or APCI-TOF tuning mix. ESI and APCI high-resolution mass spectra were measured in a positive mode using a micrOTOF QIII mass spectrometer (Bruker) and were determined by software Compass Data Analysis. Diffraction data of **1g**, **2g**, **1l**, **3j**, **3l** and **3r** were collected on a Bruker D8 VENTURE Kappa Duo PHOTON 100 CMOS with the monochromated Mo/Cu-Kα radiation. The structures were solved by direct methods (SHELXT[5]) and refined by full-matrix least-squares on F^2 values (CRYSTALS[6]). All heavy atoms were refined anisotropically. Hydrogen atoms were usually localized from the expected geometry and difference electron density maps and were refined isotropically. ORTEP-3[7] was used for structure presentation. The crystallographic data for the structures reported in this paper have been deposited with the Cambridge Crystallographic Data Centre as a supplementary publication; CCDC 2017712–2017717. Copies of the data can be obtained free of charge on application to CCDC, e-mail: deposit@ccdc.cam.ac.uk.

General procedure for the synthesis of imines 1 and 1a-m (GP1)

A round-bottom flask (50 mL) was charged with a ketone (1 equiv), aniline (5 equiv), and 4Å molecular sieves. Toluene was added (0.05 M). The resulting reaction mixture was refluxed overnight under an inert atmosphere. After completion of the reaction (GC-MS), the mixture was filtered and evaporated under vacuum. The residue was purified by column chromatography.

General procedure for photocyclization of imines 1, 1a-r, and 9a,b (GP2)

50 mg of imine precursor (**1**, **1a-r**, and **9a,b**) and TEMPO (2 equiv) were dissolved in dry DCM (50 mL) under argon atmosphere. $HBF_4.Et_2O$ (4 equiv) was added dropwise with stirring. Molecular sieves (4Å) were added and the reaction mixture was irradiated with 400 W Mercury lamp. After completion of the reaction (GC-MS), the mixture was neutralized by addition of K_2CO_3 (10%) and the organic phase was dried over anhydrous $MgSO_4$. After evaporation of solvent, the product was isolated by column chromatography.

General procedure for the synthesis of aryl-carbonitriles 4j,k (GP3) [8]

A microwave vial was charged with aryl bromide **7o,p** (3.26 mmol, 1 equiv) and CuCN (5 equiv). The vial was capped with PTFE septa and NMP (14 mL) was added. Argon was bubbled through the reaction mixture for 10 min in order to remove the oxygen. It was reacted in a microwave initiator for 4 h at 210°C. After the reaction, an aqueous saturated solution of NH_4Cl was added and the product was extracted with EtOAc, dried over anhydrous $MgSO_4$, filtered and the solvent was evaporated at the reduced pressure. The crude product was purified by column chromatography.

General procedure for the synthesis of aryl-bromophenyl ketones 5i-m (GP4)

A solution of 1,4-dibromobenzene in dry THF (0.1 M) was cooled to -78°C. The *n*-BuLi (2.5 M in hexane, 1 equiv) was added dropwise. The reaction mixture was stirred for 15 minutes. Corresponding aryl-carbonitriles **4i-m** (0.3 equiv) were added as a solution in THF (3.5 M). The solution was stirred for an hour at -78°C then warmed to room temperature. Aqueous HCl (10%) was added and the mixture was further stirred for an additional hour. The mixture was extracted by DCM (3 x 20 mL) and dried over anhydrous $MgSO_4$. After the evaporation of the solvent, the residue was purified by column chromatography.

General procedure for the synthesis of aryl-nitrophenyl ketones 6i-m (GP5)[9]

A Schlenk flask was charged with corresponding bromo-ketone (**5i-m**, 1 equiv), KNO_2 (3.0 equiv), and CuI (1.5 equiv). Dry DMSO (0.2 M) was added and the mixture was stirred at 130°C for 48 hours. After completion of the reaction (GC-MS), the mixture was diluted with EtOAc (25 mL), filtered, washed with water, and dried over anhydrous $MgSO_4$. After the evaporation of the solvent, the residue was purified by column chromatography.

General procedure for the synthesis of N-Arylbenzophenone imines 1n-r and 9a,b (GP6)[10]

A Schlenk flask was charged with aryl bromide (**7n-r**, **8b**, 0.5-1 equiv) or bis-triflate (**8a**, 0.5 equiv) , *t*-BuONa (2 equiv), $Pd(dba)_2$ (5 mol %), XPhos (10 mol %) and dry toluene (0.14 M). Benzophenone imine (1 equiv) was added and the mixture was stirred at 80°C overnight under argon atmosphere. After completion of the reaction (GC-MS), the mixture was filtered, evaporated, and purified by column chromatography.

***N*-Phenylbenzophenone imine (1)**

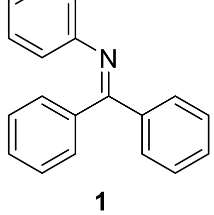

1

The GP1 was followed with 500 mg (2.74 mmol) of benzophenone and 1.25 mL (13.72 mmol) of aniline. Column chromatography using PE/EtOAc (95 : 5) provided 671 mg (2.61 mmol, 95 %) of **1** as a yellow powder.

1H NMR (400 MHz, $CDCl_3$) δ 7.79 – 7.73 (m, 2H), 7.48 (t, *J* = 7.3 Hz, 1H), 7.41 (t, *J* = 7.4 Hz, 2H), 7.31 – 7.24 (m, 3H), 7.17 – 7.11 (m, 4H), 6.93 (t, *J* = 7.4 Hz, 1H), 6.75 (d, *J* = 7.5 Hz, 2H).

^{13}C {^{1}H} NMR (101 MHz, $CDCl_3$) δ 168.23, 151.19, 139.63, 136.18, 130.69, 129.49, 129.30, 128.53, 128.43, 128.17, 127.86, 123.12, 120.91.

NMR spectra are in accordance with published data[11].

***N*-Phenyl-4-methoxybenzophenone imine (1a)**

The GP1 was followed with 250 mg (1.18 mmol) of 4-methoxybenzophenone and 0.54 mL (5.89 mmol) of aniline. Column chromatography using PE/EtOAc (96 : 4) provided 310 mg (1.08 mmol, 92 %) of **1a** as a yellow oil (*Z* and *E* isomeric ratio: 1 : 1.7).

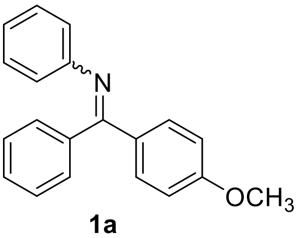

1a

^{1}H NMR (400 MHz, $CDCl_3$) δ 7.76 – 7.68 (m, 2H), 7.50 – 7.37 (m, 1H), 7.28 – 7.22 (m, 2H), 7.20 – 7.02 (m, 4H), 6.97 – 6.87 (m, 2H), 6.79 – 6.68 (m, 3H), 3.86 and 3.78 (s, 3H).

^{13}C {^{1}H} NMR (101 MHz, $CDCl_3$) δ 167.80, 167.42, 161.66, 159.57, 151.48, 151.35, 140.18, 136.35, 132.23, 131.33, 130.90, 130.46, 129.35, 128.48, 128.30, 128.28, 128.18, 128.04, 127.74, 122.88, 122.76, 121.00, 120.87, 113.41, 113.14, 55.27, 55.02.

NMR spectra are in accordance with published data[11].

***N*-Phenyl-4-methylbenzophenone imine (1b)**

The GP1 was followed with 250 mg (1.27 mmol) of 4-methylbenzophenone and 0.58 mL (6.37 mmol) of aniline. Column chromatography using PE/EtOAc (97 : 3) provided 315 mg (1.16 mmol, 91 %) of **1b** as a yellow oil (*Z* and *E* isomeric ratio: 1 : 1.1).

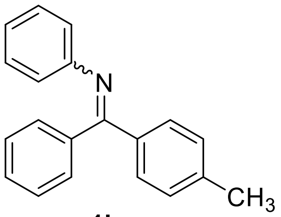

1b

^{1}H NMR (400 MHz, $CDCl_3$) δ 7.79 – 7.71 (m, 2H), 7.69 – 7.61 (m, 2H), 7.51 – 7.42 (m, 1H), 7.45 – 7.36 (m, 2H), 7.29 – 7.18 (m, 5H), 7.20 – 7.06 (m, 6H), 7.06 (d, *J* = 8.0 Hz, 2H), 7.05 – 6.98 (m, 2H), 6.98 – 6.86 (m, 2H), 6.77 – 6.68 (m, 4H), 2.41 (s, 3H), 2.31 (s, 3H).

^{13}C {^{1}H} NMR (101 MHz, $CDCl_3$) δ 168.12, 167.90, 151.34, 151.24, 140.87, 139.87, 138.35, 136.86, 136.26, 133.06, 130.45, 129.49, 129.34, 129.24, 129.19, 128.79, 128.45, 128.36, 128.29, 128.01, 127.70, 122.88, 122.84, 120.86, 120.79, 21.32, 21.21.

NMR spectra are in accordance with the published data[12].

***N*-Phenyl-4-fluorobenzophenone imine (1c)**

The GP1 was followed with 500 mg (2.50 mmol) of 4-fluorobenzophenone and 1.14 mL (12.49 mmol) of aniline. Column chromatography using PE/EtOAc (96 : 4) and subsequent recrystallization from EtOH provided 500 mg (1.82 mmol, 73 %) of **1c** as a yellow powder (*Z* and *E* isomeric ratio: 1 : 1.6).

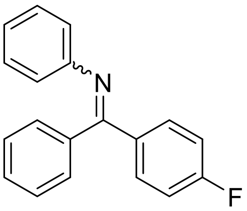

1c

^{1}H NMR (400 MHz, $CDCl_3$) δ 7.82 – 7.68 (m, 4H), 7.53 – 7.45 (m, 1H), 7.45 – 7.38 (m, 1H), 7.33 – 7.21 (m, 4H), 7.21 – 7.03 (m, 10H), 7.00 – 6.88 (m, 4H), 6.77 – 6.67 (m, 4H).

^{13}C {^{1}H} NMR (101 MHz, $CDCl_3$) δ 167.19, 166.99, 164.42 (d, *J* = 251.4 Hz), 162.48 (d, *J* = 249.3 Hz), 151.07, 150.99, 139.53, 135.93, 135.83 (d, *J* = 3.0 Hz), 132.09 (d, *J* = 4.0 Hz), 131.55 (d, *J* = 8.2 Hz), 131.40 (d, *J* = 8.7 Hz), 130.84, 129.38, 129.26, 128.68, 128.59, 128.46, 128.25, 127.97, 123.26, 123.21, 120.91, 120.80, 115.17 (d, *J* = 21.7 Hz), 115.07 (d, *J* = 21.6 Hz).

^{19}F NMR (376 MHz, $CDCl_3$) δ -112.95, -114.75.

IR ($CHCl_3$, ν cm^{-1}): 1616m (C=N), 1592s, 1576m(sh), 1506vs, 1484m, 1287m, 1271m, 1143m, 1014m, 942m, 829w(sh), 698s, 619w, 483w, 462w.

HRMS (APCI/QTOF) *m/z* $[M + H]^+$ calculated for $C_{19}H_{15}FN$ 276.1183; found 276.1181.

EI MS: 275 (100 %, M^+), 198 (57 %), 183 (43 %), 180 (39 %), 77 (81 %), 51 (24 %).

Melting point: 122.7-123.5°C

N-Phenyl-4-chlorobenzophenone imine (1d)

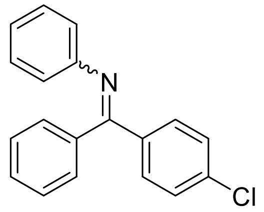

1d

The GP1 was followed with 250 mg (1.15 mmol) of 4-chlorobenzophenone and 0.530 mL (5.77 mmol) of aniline. Column chromatography using PE/EtOAc (97 : 3) provided 320 mg (1.10 mmol, 95 %) of **1d** as a yellow oil (*Z* and *E* isomeric ratio: 1 : 1.7).

^{1}H NMR (400 MHz, $CDCl_3$) δ 7.75 – 7.66 (m, 4H), 7.51 – 7.32 (m, 5H), 7.31 – 7.20 (m, 5H), 7.20 – 7.02 (m, 8H), 6.98 – 6.88 (m, 2H), 6.74 – 6.66 (m, 4H).

^{13}C {^{1}H} NMR (101 MHz, $CDCl_3$) δ 166.98, 166.96, 150.92, 150.91, 139.28, 138.08, 136.86, 135.70, 134.59, 134.51, 130.91, 130.88, 130.57, 129.35, 129.20, 128.72, 128.61, 128.46, 128.38, 128.26, 128.24, 128.00, 123.34, 123.30, 120.83, 120.72.

NMR spectra are in accordance with published data[11].

IR ($CHCl_3$, ν cm^{-1}): 1613m (C=N), 1588s, 1488m, 1566m, 1286m, 1274m, 1175m, 1143m, 1015m, 939w, 838m, 698vs, 617w.

N-Phenyl-4-bromobenzophenone imine (1e)

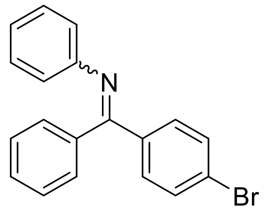

1e

The GP1 was followed with 200 mg (0.77 mmol) of 4-bromobenzophenone and 0.350 mL (3.83 mmol) of aniline. Column chromatography using PE/EtOAc (96 : 4) provided 250 mg (0.74 mmol, 97 %) of **1e** as a yellow oil (*Z* and *E* isomeric ratio: 1 : 1.25).

^{1}H NMR (400 MHz, $CDCl_3$) δ 7.75 – 7.69 (m, 2H), 7.62 (d, *J* = 8.7 Hz, 2H), 7.54 (d, *J* = 8.6 Hz, 2H), 7.50 – 7.45 (m, 1H), 7.45 – 7.37 (m, 3H), 7.31 – 7.22 (m, 4H), 7.20 – 7.07 (m, 6H), 7.00 (d, *J* = 8.4 Hz, 2H), 6.98 – 6.89 (m, 2H), 6.71 (d, *J* = 7.4 Hz, 4H).

^{13}C {^{1}H} NMR (101 MHz, $CDCl_3$) δ 167.14, 167.01, 150.90, 139.23, 138.55, 135.64, 135.00, 131.38, 131.20, 131.16, 130.92, 130.82, 129.38, 129.22, 128.78, 128.65, 128.49, 128.29, 128.03, 125.44, 123.39, 123.36, 122.94, 120.83, 120.75 (one carbon is missing due to overlapping).

IR ($CHCl_3$, ν cm^{-1}): 1613m (C=N), 1584s, 1563m, 1488m-s, 1287m, 1271m(sh), 1175w, 1142m, 1012m, 942m, 837m, 697vs, 618w, 489w, 461w.

EI MS: 335 (79 %, M^+), 256 (36 %), 245 (21 %), 180 (61 %), 77 (100 %), 51 (29 %).

HRMS (APCI/QTOF) m/z $[M + H]^+$ calculated for $C_{19}H_{15}{}^{79}BrN$ 336.0382; found 336.0383.

^{1}H NMR spectrum is in accordance with published data[13].

N-Phenyl-4-trifluoromethylbenzophenone imine (1f)

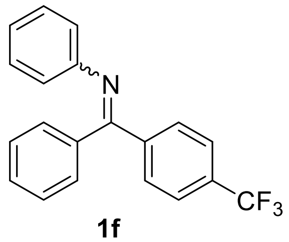

1f

The GP1 was followed with 200 mg (0.80 mmol) of 4-trifluoromethylbenzophenone and 0.37 mL (4.05 mmol) of aniline. Column chromatography using PE/EtOAc (96 : 4) provided 252 mg (0.78 mmol, 97 %) of **1f** as a yellow oil (*Z* and *E* isomeric ratio: 1 : 1.05).

^{1}H NMR (400 MHz, $CDCl_3$) δ 7.84 (d, *J* = 8.1 Hz, 2H), 7.74 – 7.66 (m, 2H), 7.63 (d, *J* = 8.2 Hz, 2H), 7.54 – 7.41 (m, 3H), 7.43 – 7.34 (m, 2H), 7.32 – 7.16 (m, 5H), 7.20 – 7.04 (m, 6H), 6.97 – 6.87 (m, 2H), 6.75 – 6.63 (m, 4H).
^{13}C {^{1}H} NMR (126 MHz, $CDCl_3$) δ 166.84, 166.64, 142.92, 142.90, 139.88, 139.87, 138.86, 135.46, 132.20 (q, *J* = 32.4 Hz) 131.06, 130.47 (q, *J* = 32.5 Hz), 129.78, 129.56, 129.35, 129.12, 128.94, 128.64, 128.54, 128.34, 128.12, 125.10 (q, *J* = 3.8 Hz), 124.94 (q, *J* = 3.8 Hz), 123.59, 123.96 (q, *J* = 272.4 Hz), 123.75 (q, *J* = 273.1 Hz), 123.53, 120.74, 120.64.
^{19}F NMR (376 MHz, $CDCl_3$) δ -65.89, -65.90.
IR ($CHCl_3$, *ν* cm^{-1}): 1614m (C=N), 1593m, 1578m, 1291m, 1325 (CF_3) 1289m, 1272m (CF_3), 1239m, 1171s, 1133s (CF_3), 1067s, 1001w, 997s, 990w, 964m, 926w, 848m, 827w, 659m(sh), 620w ($ν_{as}$-CF_3), 617w, 611w.
EI MS: 325 (90 %, M^+), 256 (26 %), 248 (40 %), 233 (27 %), 180 (71 %), 77 (100 %), 51 (26 %).
HRMS (APCI/QTOF) *m/z* $[M + H]^+$ calculated for $C_{20}H_{15}F_3N$ 326.1151; found 326.1149.

_N_-Phenyl-4-nitrobenzophenone imine (1g)

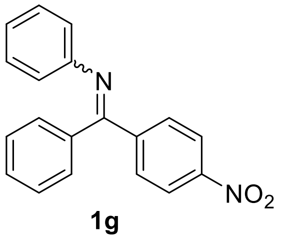

1g

The GP1 was followed with 250 mg (1.10 mmol) of 4-nitrobenzophenone and 0.50 mL (5.50 mmol) of aniline. Column chromatography using PE/EtOAc (95 : 5) and subsequent recrystallization from EtOH provided 240 mg (0.79 mmol, 72 %) of **1g** as a yellow powder (*Z* and *E* isomeric ratio: 1 : 1.1).
^{1}H NMR (500 MHz, $CDCl_3$) δ 8.29 – 8.24 (m, 2H), 8.17 – 8.12 (m, 2H), 7.95 – 7.88 (m, 2H), 7.71 – 7.67 (m, 2H), 7.56 – 7.51 (m, 1H), 7.47 – 7.43 (m, 2H), 7.38 – 7.28 (m, 5H), 7.22 – 7.15 (m, 4H), 7.12 – 7.09 (m, 2H), 7.03 – 6.95 (m, 2H), 6.75 (dd, *J* = 8.4, 1.2 Hz, 2H), 6.72 – 6.68 (m, 2H).
^{13}C {^{1}H} NMR (126 MHz, $CDCl_3$) δ 166.33, 166.16, 150.07, 149.97, 148.51, 147.05, 145.14, 142.64, 137.95, 134.54, 131.40, 130.37, 130.12, 129.30, 129.25, 128.92, 128.73, 128.59, 128.44, 128.22, 123.89, 123.78, 123.32, 123.21, 120.60, 120.46.
IR ($CHCl_3$, *ν* cm^{-1}): 1618m (C=N), 1600m, 1591m, 1578s(sh), 1524vs ($ν_{as}NO_2$) 1483m, 1407w, 1317m, 1350s ($ν_{as}NO_2$), 1302w, 1288w, 1171w, 1143w, 1108w, 1072w, 1026w, 1001w, 853m ($β_s$ NO_2), 825w, 697m.
EI MS:302 (100 %, M^+), 255 (27 %), 225 (23 %), 210 (16 %), 180 (71 %), 77 (64 %), 51 (16 %).
HRMS (APCI/QTOF) *m/z* $[M + H]^+$ calculated for $C_{19}H_{15}N_2O_2$ 303.1128; found 303.1130.
Melting point: 128.8-131.5°C

_N_-((Naphthalen-2-yl)phenylmethylene)aniline (1h)

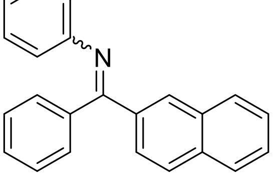

1h

The GP1 was followed with 200 mg (0.86 mmol) of naphthalen-2-yl(phenyl)methanone and 0.40 mL (4.31 mmol) of aniline. Column chromatography using PE/EtOAc (96 : 4) provided 249 mg (0.81 mmol, 94 %) of **1h** as a yellow solid (*Z* and *E* isomeric ratio: 1 : 1.35).
^{1}H NMR (400 MHz, $CDCl_3$) 8.12 (dd, *J* = 8.7, 1.8 Hz, 1H), 8.00 (s, 1H), 7.94 – 7.85 (m, 2H), 7.84 – 7.75 (m, 4H), 7.75 – 7.68 (m, 2H), 7.65 (s, 1H), 7.59 – 7.38 (m, 7H), 7.36 – 7.27 (m, 3H), 7.23 – 7.08 (m, 7H), 6.97 – 6.91 (m, 1H), 6.91 – 6.86 (m, 1H), 6.82 – 6.72 (m, 4H).
^{13}C {^{1}H} NMR (101 MHz, $CDCl_3$) δ 168.00, 167.91, 151.22, 151.09, 139.81, 136.95, 136.13, 134.43, 133.63, 132.82, 132.62, 132.40, 130.63, 130.58, 129.45, 129.36, 129.34, 128.92, 128.49, 128.45, 128.38, 128.18,

128.12, 127.88, 127.85, 127.61, 127.56, 127.41, 127.26, 126.72, 126.66, 126.29, 126.24, 125.39, 123.12, 123.06, 120.89, 120.82.

IR ($CHCl_3$, ν cm^{-1}): 1655m, 1611m (C=N), 1591s, 1577m, 1505w, 1468w, 1291m, 1171w, 1145m, 1001w, 825m, 697s, 615w.

EI MS: 307 (100 %, M^+), 230 (50 %), 215 (60 %), 180 (37 %), 127 (17 %), 77 (55 %), 51 (14 %).

HRMS (APCI/QTOF) *m/z* $[M + H]^+$ Calculated for $C_{23}H_{18}N$ 308.1433; found 308.1430.

Melting point: 75.5-79.5°C

1-(Naphthalen-2-yl)-1-(4-nitrophenyl)-*N*-phenylmethanimine (1i)

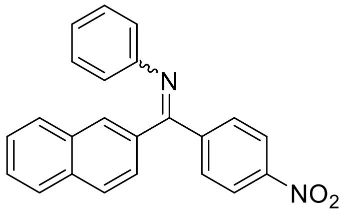

1i

The GP1 was followed with 70 mg (0.252 mmol) of **6i** and 115 µL (1.260 mmol) of aniline. Column chromatography using PE/EtOAc (96 : 4) provided 74 mg (0.210 mmol, 83 %) of **1i** as a yellow solid (*Z* and *E* isomeric ratio: 1 : 1.2).

^1H NMR (400 MHz, $CDCl_3$) δ 8.26 (d, *J* = 8.9 Hz, 2H), 8.18 (d, *J* = 8.8 Hz, 2H), 8.12 (dd, *J* = 8.7, 1.8 Hz, 1H), 7.97 – 7.91 (m, 3H), 7.90 (d, *J* = 8.2 Hz, 1H), 7.85 (d, *J* = 1.3 Hz, 1H), 7.80 (dd, *J* = 12.3, 8.0 Hz, 2H), 7.76 – 7.70 (m, 2H), 7.62 (s, 1H), 7.60 – 7.47 (m, 4H), 7.38 (d, *J* = 8.8 Hz, 2H), 7.23 – 7.08 (m, 5H), 7.01 – 6.90 (m, 2H), 6.78 (dd, *J* = 8.4, 1.1 Hz, 2H), 6.72 (dd, *J* = 8.4, 1.1 Hz, 2H).

^{13}C {^1H} NMR (101 MHz, $CDCl_3$) δ 165.97, 165.92, 150.42, 150.33, 148.94, 147.53, 145.47, 142.94, 135.67, 134.66, 133.10, 132.56, 132.50, 132.48, 130.62, 130.37, 130.20, 129.41, 128.95, 128.74, 128.66, 128.37, 128.27, 128.01, 127.79, 127.75, 127.70, 127.23, 126.70, 126.65, 126.14, 124.78, 124.00, 123.78, 123.30, 123.24, 120.75, 120.46.

IR ($CHCl_3$, ν cm^{-1}): 1612m (C=N), 1590s, 1520 (ν-NO_2), 1483m, 1347vs (ν-NO_2), 1197w, 1143w, 1107w, 1072vw, 1015w, 935m, 850m, 751m (ν-NO_2), 694m, 478m.

EI MS: 352 (100 %, M^+), 305 (24 %), 260 (24 %), 230 (66 %), 179 (19 %), 77 (26 %).

HRMS (APCI/QTOF) *m/z* $[M + H]^+$ calculated for $C_{23}H_{17}N_2O_2$ 353.1284; found 353.1289.

Melting point: 107.1-110.7°C

1-(4-Nitrophenyl-1-(phenanthrene-3-yl)-*N*-phenylmethanimine (1j)

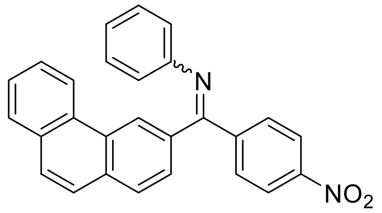

1j

The GP1 was followed with 80 mg (0.24 mmol) of **6j** and 110 µl (1.22 mmol) of aniline. Column chromatography using PE/EtOAc (92 : 8) provided 79 mg (0.20 mmol, 80 %) of **1j** as a yellow amorphous solid (*Z* and *E* isomeric ratio: 1 : 1.1).

^1H NMR (400 MHz, $CDCl_3$) δ 8.99 – 8.93 (m, 1H), 8.54 – 8.49 (m, 1H), 8.47 – 8.43 (m, 1H), 8.37 (dd, *J* = 6.1, 3.4 Hz, 1H), 8.30 – 8.25 (m, 2H), 8.24 – 8.18 (m, 2H), 8.04 – 7.97 (m, 3H), 7.97 – 7.88 (m, 3H), 7.85 (d, *J* = 8.8 Hz, 1H), 7.82 – 7.75 (m, 3H), 7.70 (d, *J* = 8.9 Hz, 1H), 7.67 – 7.58 (m, 4H), 7.47 – 7.39 (m, 2H), 7.30 (dd, *J* = 8.2, 1.6 Hz, 1H), 7.23 – 7.17 (m, 2H), 7.17 – 7.10 (m, 2H), 7.03 – 6.96 (m, 1H), 6.96 – 6.90 (m, 1H), 6.85 – 6.79 (m, 2H), 6.79 – 6.72 (m, 2H).

^{13}C {^1H} NMR (101 MHz, $CDCl_3$) δ 166.12, 166.02, 150.41, 150.38, 148.90, 147.50, 145.46, 142.97, 136.02, 133.73, 132.84, 132.07, 132.02, 131.98, 130.40, 130.23, 130.19, 129.77, 129.70, 129.62, 128.91, 128.71, 128.67, 128.63, 128.61, 128.49, 128.33, 127.03, 127.00, 126.96, 126.90, 126.19, 124.30, 124.14, 123.94, 123.74, 123.27, 123.21, 122.51, 122.33, 120.65, 120.45.

IR ($CHCl_3$, ν cm^{-1}):1617w (sh), 1591m, 1577w, 1524s (ν_{as}-NO_2), 1348vs (NO_2), 1288w, 1265m-w, 1239w, 1171w, 1150w, 1108w, 1092w, 1073w, 1015w, 1001w, 969w, 866m, 844m, 824w, 618w, 500w.

EI MS: 402 (100 %, M^+), 355 (20 %), 310 (33 %), 280 (57 %), 177 (24 %), 77 (47 %).

HRMS (APCI/QTOF) *m/z* $[M + H]^+$ calculated for $C_{27}H_{19}N_2O_2$ 403.1441; found 403.1442.

Melting point: amorphous

1-(Benzo[*c*]phenanthren-2-yl)-1-(4-nitrophenyl)-*N*-phenylmethanimine (1k)

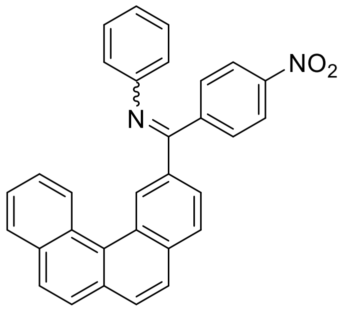

1k

The GP1 was followed with 50 mg (0.132 mmol) of **6k** and 60 μL (0.657 mmol) of aniline. Column chromatography using PE/EtOAc (90 : 10) provided 45 mg (0.099 mmol, 75 %) of **1k** as a yellow solid (*Z* and *E* isomeric ratio: 1 : 1.4).

^1H NMR (400 MHz, $CDCl_3$) δ 9.02 (s, 1H), 8.77 (s, 1H), 8.61 (d, *J* = 8.6 Hz, 1H), 8.35 (dd, *J* = 8.5, 1.7 Hz, 1H), 8.28 – 8.18 (m, 4H), 8.12 (d, *J* = 8.5 Hz, 1H), 8.02 (d, *J* = 8.2 Hz, 1H), 7.99 – 7.76 (m, 13H), 7.62 – 7.51 (m, 2H), 7.51 – 7.29 (m, 7H), 7.24 – 7.15 (m, 2H), 7.08 – 6.95 (m, 4H), 6.78 (dd, *J* = 8.4, 1.1 Hz, 2H).

^{13}C {^1H} NMR (101 MHz, $CDCl_3$) δ 166.47, 166.17, 150.59, 150.57, 148.97, 147.53, 145.59, 143.63, 135.93, 134.98, 133.53, 133.38, 133.27, 132.54, 131.32, 131.27, 131.05, 130.35, 130.22, 129.73, 129.72, 129.56, 129.41, 129.28, 129.02, 129.00, 128.92, 128.79, 128.69, 128.65, 128.47, 128.29, 128.08, 127.94, 127.63, 127.35, 127.24, 127.22, 126.94, 126.88, 126.60, 126.53, 126.33, 126.10, 124.72, 124.30, 123.86, 123.37, 123.34, 121.48, 120.61 (3 carbons are missing due to overlapping).

IR ($CHCl_3$, ν cm^{-1}): 1614m (C=N), 1590m, 1520s (ν-NO_2), 1482m, 1397w, 1345vs (ν-NO_2), 1271m, 1216m, 1105w, 1013w, 949m, 849s, 755m (ν-NO_2), 696m, 616w.

EI MS: 452 (100 %, M^+), 422 (43 %), 405 (26 %), 330 (39 %), 226 (40 %), 77 (30 %).

HRMS (APCI/QTOF) *m/z* $[M + H]^+$ calculated for $C_{31}H_{21}N_2O_2$ 453.1597; found 453.1599.

Melting point: 228.5-229.1°C

(*Z*)-1-(naphthalen-1-yl)-1-(4-nitrophenyl)-*N*-phenylmethanimine (1l)

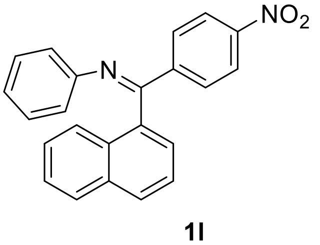

1l

The GP1 was followed with 74 mg (0.267 mmol) of **6l** and 122 μL (1.340 mmol) of aniline. Column chromatography using PE/EtOAc (91 : 9) provided 81 mg (0.230 mmol, 86 %) of **1l** as a yellow solid .

^1H NMR (400 MHz, $CDCl_3$) δ 8.20 (d, *J* = 9.0 Hz, 2H), 7.92 (d, *J* = 9.0 Hz, 2H), 7.88 – 7.80 (m, 2H), 7.56 (d, *J* = 8.5 Hz, 1H), 7.47 (ddd, *J* = 8.2, 6.9, 1.3 Hz, 1H), 7.44 – 7.37 (m, 2H), 7.23 (dd, *J* = 7.0, 1.2 Hz, 1H), 7.06 – 6.97 (m, 2H), 6.86 (tt, *J* = 7.0, 1.2 Hz, 1H), 6.72 (dd, *J* = 8.5, 1.2 Hz, 2H).

^{13}C {^1H} NMR (101 MHz, $CDCl_3$) δ 165.81, 150.22, 148.98, 144.86, 133.42, 133.15, 130.79, 129.48, 129.32, 128.64, 128.31, 127.02, 126.90, 126.33, 125.40, 124.96, 124.18, 123.49, 119.88.

IR ($CHCl_3$, ν cm^{-1}): 1619m (C=N), 1598s, 1521vs (ν-NO_2), 1507m(sh), 1484m, 1448w, 1407w, 1394w, 1347vs (ν-NO_2), 1254m, 1196m, 1112m, 1071w, 1042w, 1012m, 942m, 865m, 799s, 778s, 759s (ν-NO_2), 695s.

EI MS: 352 (100 %, M^+), 305 (29 %), 230 (29 %), 77 (26 %).

HRMS (APCI/QTOF) *m/z* $[M + H]^+$ calculated for $C_{23}H_{16}N_2O_2$ 353.1284; found 353.1286.

Melting point: 142.9-145.0°C

(*Z*)-1-(4-nitrophenyl)-1-(phenanthren-9-yl)-*N*-phenylmethanimine (1m)

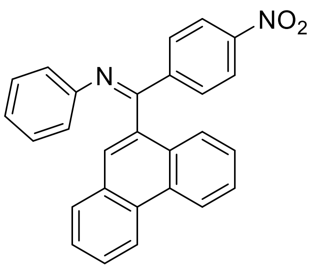

1m

The GP1 was followed with 65 mg (0.199 mmol) of **6m** and 91 µL (0.997 mmol) of aniline. Column chromatography using PE/EtOAc (90 : 10) provided 75 mg (0.186 mmol, 94 %) of **1m** as a yellow solid.

^{1}H NMR (400 MHz, $CDCl_3$) δ 8.70 (d, *J* = 8.3 Hz, 1H), 8.66 (d, *J* = 8.3 Hz, 1H), 8.18 (d, *J* = 8.9 Hz, 2H), 7.99 (d, *J* = 8.9 Hz, 2H), 7.80 (dd, *J* = 7.9, 1.1 Hz, 1H), 7.71 – 7.62 (m, 3H), 7.62 – 7.56 (m, 1H), 7.56 – 7.42 (m, 2H), 7.08 – 6.96 (m, 2H), 6.90 – 6.75 (m, 3H).

^{13}C {^{1}H} NMR (101 MHz, $CDCl_3$) δ 165.48, 150.13, 148.99, 144.73, 132.31, 130.42, 130.32, 130.14, 129.50 (2C), 128.96, 128.41, 128.18, 127.62, 127.14, 127.12, 127.07, 126.38, 124.29, 123.50, 123.23, 122.58, 119.82.

IR ($CHCl_3$, *ν* cm^{-1}): 1619m (C=N), 1599m, 1520s (ν-NO_2), 1484m, 1450m, 1406w, 1346vs (ν-NO_2), 1260m, 1189m, 1107w, 1040w, 1012w, 847s, 750s (ν-NO_2), 694m, 616m.

EI MS: 402 (100 %, M$^+$), 355 (34 %), 280 (28 %), 176 (20 %), 77 (34 %).

HRMS (APCI/QTOF) *m*/*z* [M + H]$^+$ calculated for $C_{27}H_{19}N_2O_2$ 403.1441; found 403.1441.

Melting point: 171.5-172.5°C

***N*-(Naphthalen-2-yl)-1,1-diphenylmethanimine (1n)**

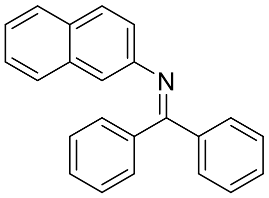

1n

The GP6 was followed with 250 mg (1.207 mmol) of 2-bromonaphthalene (**7n**) and 200 µL (1.190 mmol) of benzophenone imine. Column chromatography using PE/EtOAc (96 : 4) provided 330 mg (1.074 mmol, 89 %) of **1n** as a yellow solid.

^{1}H NMR (400 MHz, $CDCl_3$) δ 7.84 – 7.75 (m, 2H), 7.71 (d, *J* = 7.7 Hz, 1H), 7.67 – 7.59 (m, 2H), 7.53 – 7.47 (m, 1H), 7.47 – 7.40 (m, 2H), 7.38 (ddd, *J* = 8.2, 6.9, 1.5 Hz, 1H), 7.33 (ddd, *J* = 8.1, 6.9, 1.4 Hz, 1H), 7.26 – 7.11 (m, 6H), 6.93 (dd, *J* = 8.6, 2.1 Hz, 1H).

^{13}C {^{1}H} NMR (101 MHz, $CDCl_3$) δ 168.49, 148.83, 139.65, 136.10, 133.85, 130.74, 130.21, 129.49, 129.35, 128.60, 128.18, 128.13, 127.95, 127.55, 127.36, 125.91, 124.36, 121.74, 117.46.

NMR spectra are in accordance with published data[14].

***N*-(Phenanthren-3-yl)-1,1-diphenylmethanimine (1o)**

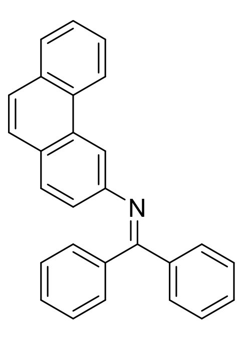

1o

The GP6 was followed with 200 mg (0.778 mmol) of 3-bromphenanthrene (**7o**) and 130 µL (0.775 mmol) of benzophenone imine. Column chromatography using PE/EtOAc (95 : 5) provided 260 mg (0.727 mmol, 93 %) of **1o** as a yellow solid.

^{1}H NMR (400 MHz, $CDCl_3$) δ 8.51 – 8.41 (m, 1H), 8.10 (d, *J* = 2.0 Hz, 1H), 7.90 – 7.79 (m, 3H), 7.64 (d, *J* = 8.4 Hz, 1H), 7.63 – 7.42 (m, 7H), 7.25 – 7.16 (m, 5H), 6.96 (dd, *J* = 8.4, 2.0 Hz, 1H).

^{13}C {^{1}H} NMR (101 MHz, $CDCl_3$) δ 168.71, 149.63, 139.69, 136.15, 132.17, 130.83, 130.77, 130.10, 129.60, 129.40, 128.78, 128.69, 128.44, 128.35, 128.25, 127.98, 126.62, 126.44, 126.21, 125.39, 122.66, 121.20, 114.39.

IR ($CHCl_3$, *ν* cm^{-1}): 1620m (C=N), 1612m, 1596m, 1581m, 1510m-w(sh), 1288m, 1236w, 1095w, 1035w, 1001w, 990w, 960m, 841m, 698s, 619m.

EI MS: 357 (100 %, M$^+$), 280 (43 %), 176 (37 %), 165 (17 %).

HRMS (APCI/QTOF) *m/z* [M + H]+ calculated for $C_{27}H_{20}N$ 358.1590; found 358.1590.

Melting point: 167.6-169.5°C

N-(Benzo[c]phenanthren-2-yl)-1,1-diphenylmethanimine (1p)

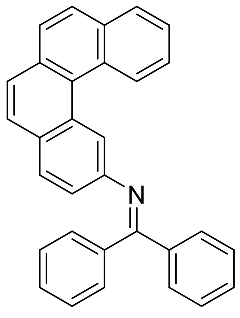

1p

The GP6 was followed with 240 mg (0.775 mmol) of 2-bromobenzo[*c*]phenathrene (**7p**) and 130 µL (0.78 mmol) of benzophenone imine. Column chromatography using PE/EtOAc (95 : 5) provided 280 mg (0.687 mmol, 88 %) of **1p** as a yellow solid.

^{1}H NMR (400 MHz, $CDCl_3$) δ 8.24 (s, 1H), 8.09 (d, *J* = 8.5 Hz, 1H), 7.97 – 7.89 (m, 2H), 7.86 – 7.79 (m, 4H), 7.76 (d, *J* = 8.5 Hz, 1H), 7.69 (d, *J* = 8.5 Hz, 1H), 7.59 – 7.49 (m, 2H), 7.49 – 7.40 (m, 3H), 7.40 – 7.27 (m, 6H).

^{13}C {^{1}H} NMR (101 MHz, $CDCl_3$) δ 168.51, 149.84, 139.92, 136.23, 133.13, 131.19, 130.81, 130.71, 130.23, 130.14, 129.87, 129.55, 129.14, 128.86, 128.28, 128.24, 128.18, 127.48, 127.33, 127.15, 127.00, 126.78, 125.99, 125.47, 125.41, 121.70, 117.58.

IR ($CHCl_3$, *ν* cm^{-1}): 3055w, 3009w, 1620m (C=N), 1612m, 1595s, 1490m, 1446m, 1288m, 1275m, 1178w, 1138m, 1075w, 1026w, 954m, 930w, 843vs, 790m, 753s, 694s, 618m, 523w.

EI MS: 407 (100 %, M+), 330 (14 %), 226 (54 %), 165 (13 %).

HRMS (APCI/QTOF) *m/z* [M + H]+ calculated for $C_{31}H_{22}N$ 408.1746; found 408.1747.

Melting point: 210.0-212.5°C

N-(Naphthalen-1-yl)-1,1-diphenylmethanimine (1q)

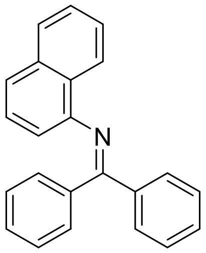

1q

The GP6 was followed with 250 mg (1.207 mmol) of 1-bromonaphthalene (**7q**) and 200 µL (1.190 mmol) of benzophenone imine. Column chromatography using PE/EtOAc (96 : 4) provided 345 mg (1.122 mmol, 93 %) of **1q** as a yellow solid.

^{1}H NMR (400 MHz, $CDCl_3$) δ 8.11 – 8.05 (m, 1H), 7.94 – 7.87 (m, 2H), 7.82 – 7.76 (m, 1H), 7.57 – 7.42 (m, 6H), 7.24 – 7.13 (m, 4H), 7.11 – 7.05 (m, 2H), 6.47 (dd, *J* = 7.3, 1.1 Hz, 1H).

^{13}C NMR (101 MHz, $CDCl_3$) δ 168.63, 147.91, 139.36, 136.17, 133.75, 130.82, 129.48, 128.67, 128.57, 128.23, 127.82, 127.80, 127.52, 125.96, 125.59, 125.40, 123.95, 123.15, 114.24.

NMR spectra are in accordance with published data[15].

N-(Phenanthren-9-yl)-1,1-diphenylmethanimine (1r)

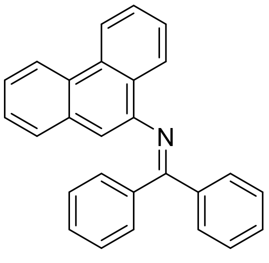

1r

The GP6 was followed with 250 mg (0.972 mmol) of 9-bromophenanthrene (**7r**) and 160 µL (0.952 mmol) of benzophenone imine. Column chromatography using PE/EtOAc (95 : 5) provided 313 mg (0.876 mmol, 90 %) of **1r** as a yellow solid.

^{1}H NMR (400 MHz, $CDCl_3$) δ 8.67 (d, *J* = 7.0 Hz, 1H), 8.58 (d, *J* = 8.0 Hz, 1H), 8.16 (dd, *J* = 8.0, 1.5 Hz, 1H), 7.97 – 7.87 (m, 2H), 7.68 (ddd, *J* = 8.3, 6.9, 1.5 Hz, 1H), 7.61 (ddd, *J* = 8.1, 6.9, 1.3 Hz, 1H), 7.58 – 7.51 (m, 2H), 7.51 – 7.40 (m, 4H), 7.18 – 7.11 (m, 5H), 6.72 (s, 1H).

^{13}C {^{1}H} NMR (101 MHz, $CDCl_3$) δ 168.91, 146.37, 139.35, 135.97, 132.36, 130.88, 130.57, 129.52, 128.64, 128.42, 128.31, 128.25, 127.95, 127.91 (2C), 126.71, 126.48, 126.46, 124.78, 124.57, 122.71, 122.36, 112.67.

IR ($CHCl_3$, *ν* cm^{-1}): 1613m (C=N), 1559m, 1578m, 1289m, 1239w, 1195w, 1036m, 1001w, 985w, 958m, 849w, 701, 618w.

EI MS: 357 (100 %, M^+), 280 (54 %), 176 (36 %), 165 (16 %).

HRMS (APCI/QTOF) *m/z* $[M + H]^+$ calculated for $C_{27}H_{20}N$ 358.1590; found 358.1587.

Melting point: 172.0-175.0°C

6-Phenylphenanthridine (2)

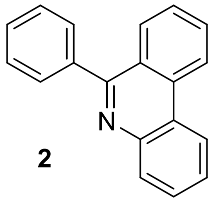

2

The GP2 was followed with 50 mg (0.194 mmol) of **1** (4 hours irradiation). Column chromatography using PE/EtOAc (95 : 5) provided 35 mg (0.137 mmol, 71 %) of **2** as a white powder.

^{1}H NMR (400 MHz, $CDCl_3$) δ 8.71 (d, *J* = 8.3 Hz, 1H), 8.63 (dd, *J* = 8.1, 1.4 Hz, 1H), 8.26 (dd, *J* = 8.1, 1.0 Hz, 1H), 8.16 – 8.08 (m, 1H), 7.86 (ddd, *J* = 8.3, 7.0, 1.3 Hz, 1H), 7.81 – 7.73 (m, 3H), 7.70 (ddd, *J* = 8.3, 7.0, 1.4 Hz, 1H), 7.66 – 7.50 (m, 4H).

^{13}C {^{1}H} NMR (101 MHz, $CDCl_3$) δ 161.24, 143.73, 139.72, 133.43, 130.55, 130.32, 129.71, 128.91, 128.83, 128.70, 128.41, 127.10, 126.92, 125.22, 123.72, 122.17, 121.92.

NMR spectra are in accordance with published data[16].

6-(4-Methoxyphenyl)phenanthridine (2a)

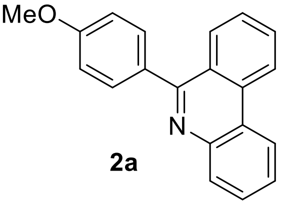

2a

The GP2 was followed with 50 mg (0.174 mmol) of **1a** (14 hours irradiation). Column chromatography using PE/EtOAc (95 : 5) provided 21 mg (0.074 mmol, 42 %) of **2a** as a white solid.

^{1}H NMR (500 MHz, $CDCl_3$) δ 8.70 (d, *J* = 8.3 Hz, 1H), 8.61 (d, *J* = 8.1 Hz, 1H), 8.23 (d, *J* = 8.1 Hz, 1H), 8.17 (d, *J* = 8.3 Hz, 1H), 7.86 (t, *J* = 7.7 Hz, 1H), 7.79 – 7.65 (m, 4H), 7.63 (t, *J* = 7.6 Hz, 1H), 7.10 (d, *J* = 8.6 Hz, 2H), 3.92 (s, 3H).

^{13}C {^{1}H} NMR (126 MHz, $CDCl_3$) δ 160.88, 160.13, 143.89, 133.51, 132.32, 131.17, 130.44, 130.27, 128.94, 128.77, 127.05, 126.71, 125.36, 123.61, 122.20, 121.90, 113.89, 55.45.

NMR spectra are in accordance with the published data[17].

6-(4-Methylphenyl)phenanthridine (2b) and 9-methyl-6-phenylphenanthridine (3b)

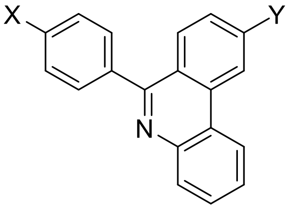

2b: X = Me, Y = H
3b: X = H, Y = Me

The GP2 was followed with 50 mg (0.184 mmol) of **1b** (8 hours irradiation). Column chromatography using PE/EtOAc (93 : 7) provided 25 mg (0.093 mmol, 50 %, white powder) of **2b** and 15 mg (0.056 mmol, 30 %, white powder) of **3b**.

2b

^{1}H NMR (400 MHz, $CDCl_3$) δ 8.70 (d, *J* = 8.3 Hz, 1H), 8.61 (dd, *J* = 8.1, 1.4 Hz, 1H), 8.25 (dd, *J* = 8.1, 1.0 Hz, 1H), 8.20 – 8.09 (m, 1H), 7.85 (ddd, *J* = 8.3, 7.0, 1.3 Hz, 1H), 7.76 (ddd, *J* = 8.2, 7.0, 1.4 Hz, 1H), 7.71 – 7.55 (m, 4H), 7.38 (d, *J* = 7.7 Hz, 2H), 2.49 (s, 3H).

^{13}C {^{1}H} NMR (101 MHz, $CDCl_3$) δ 161.28, 143.80, 138.57, 136.86, 133.42, 130.45, 130.29, 129.66, 129.07, 128.96, 128.76, 127.02, 126.77, 125.29, 123.65, 122.14, 121.89, 21.37.

NMR spectra are in accordance with the published data[16].

3b

^{1}H NMR (400 MHz, $CDCl_3$) δ 8.61 (dd, *J* = 8.3, 1.5 Hz, 1H), 8.48 (s, 1H), 8.24 (dd, *J* = 8.1, 1.4 Hz, 1H), 7.99 (d, J = 8.4 Hz, 1H), 7.79 – 7.70 (m, 3H), 7.70 – 7.64 (m, 1H), 7.61 – 7.49 (m, 3H), 7.43 (dd, *J* = 8.4, 1.2 Hz, 1H), 2.66 (s, 3H).

^{13}C {^{1}H} NMR (101 MHz, $CDCl_3$) δ 161.09, 143.94, 141.01, 139.89, 133.62, 130.27, 129.73, 128.84, 128.72, 128.65, 128.41, 128.39, 126.71, 123.63, 123.39, 121.94, 121.87, 22.29.

NMR spectra are in accordance with the published data[18].

6-(4-Fluorophenyl)phenanthridine (2c) and 9-fluoro-6-phenylphenanthridine (3c)

The GP2 was followed with 50 mg (0.182 mmol) of **1c** (4 hours irradiation). Column chromatography using PE/EtOAc (94 : 6) provided 30 mg (0.110 mmol, 60 %, white powder) of **2c** and 6 mg (0.02 mmol, 12 %, white powder) of **3c**.

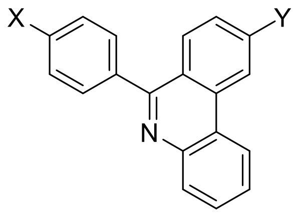

2c: X = F, Y = H
3c: X = H, Y = F

2c

^{1}H NMR (500 MHz, $CDCl_3$) δ 8.70 (d, *J* = 8.3 Hz, 1H), 8.61 (d, *J* = 8.1 Hz, 1H), 8.24 (d, *J* = 8.1 Hz, 1H), 8.07 (d, *J* = 8.2 Hz, 1H), 7.86 (t, *J* = 7.6 Hz, 1H), 7.80 – 7.66 (m, 4H), 7.62 (t, *J* = 7.6 Hz, 1H), 7.31 – 7.19 (m, 2H).

^{13}C {^{1}H} NMR (126 MHz, $CDCl_3$) δ 163.15 (d, *J* = 248.1 Hz), 160.09, 143.64, 135.76 (d, *J* = 3.3 Hz), 133.47, 131.59 (d, *J* = 8.3 Hz), 130.62, 130.24, 128.90, 128.57, 127.19, 127.03, 125.09, 123.69, 122.26, 121.92, 115.41 (d, *J* = 21.6 Hz).

NMR spectra are in accordance with published data[19].

3c

^{1}H NMR (500 MHz, $CDCl_3$) δ 8.49 (d, *J* = 8.1 Hz, 1H), 8.29 (dd, *J* = 10.3, 2.3 Hz, 1H), 8.25 (d, *J* = 8.1 Hz, 1H), 8.13 (dd, *J* = 9.0, 5.8 Hz, 1H), 7.79 (t, *J* = 7.3 Hz, 1H), 7.75 – 7.64 (m, 3H), 7.61 – 7.48 (m, 3H), 7.34 (td, *J* = 8.9, 2.3 Hz, 1H).

^{13}C {^{1}H} NMR (126 MHz, $CDCl_3$) δ 163.81 (d, *J* = 252.3 Hz), 160.66 (d, *J* = 1.1 Hz), 144.05, 139.52, 135.88 (d, *J* = 9.5 Hz), 131.92 (d, *J* = 9.4 Hz), 130.41, 129.66, 129.53, 128.88, 128.51, 126.99, 123.32 (d, *J* = 4.1 Hz), 122.26 (d, *J* = 1.7 Hz), 122.13 (d, *J* = 0.6 Hz), 116.19 (d, *J* = 23.6 Hz), 107.37 (d, *J* = 22.3 Hz).

NMR spectra are in accordance with published data[20].

6-(4-Chlorophenyl)phenanthridine (2d) and 9-chloro-6-phenylphenanthridine (3d)

The GP2 was followed with 50 mg (0.171 mmol) of **1d** (4 hours irradiation). Column chromatography using PE/EtOAc (92 : 8) provided 24 mg (0.083 mmol, 49 %, white powder) of **2d** and 9 mg (0.031 mmol, 18 %) of **3d** as white powder.

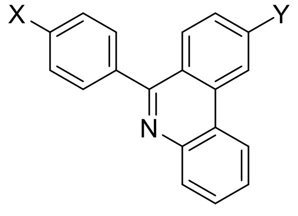

2d: X = Cl, Y = H
3d: X = H, Y = Cl

2d

^{1}H NMR (400 MHz, $CDCl_3$) δ 8.71 (d, *J* = 8.8 Hz, 1H), 8.62 (dd, *J* = 8.1, 1.5 Hz, 1H), 8.24 (dd, *J* = 8.0, 1.5 Hz, 1H), 8.06 (dd, *J* = 8.5, 0.8 Hz, 1H), 7.87 (ddd, *J* = 8.3, 7.0, 1.3 Hz, 1H), 7.77 (ddd, *J* = 8.3, 7.0, 1.5 Hz, 1H), 7.74 – 7.67 (m, 3H), 7.63 (ddd, *J* = 8.3, 7.0, 1.2 Hz, 1H), 7.58 – 7.51 (m, 2H).

^{13}C {^{1}H} NMR (101 MHz, $CDCl_3$) δ 159.93, 143.67, 138.16, 134.88, 133.47, 131.13, 130.68, 130.30, 128.94, 128.65, 128.47, 127.24, 127.13, 124.96, 123.73, 122.31, 121.95.

NMR spectra are in accordance with the published data[21].

3d

^{1}H NMR (400 MHz, $CDCl_3$) 8.66 (d, *J* = 2.0 Hz, 1H), 8.53 (dd, *J* = 8.2, 1.0 Hz, 1H), 8.25 (d, *J* = 8.1 Hz, 1H), 8.05 (d, *J* = 8.8 Hz, 1H), 7.79 (ddd, *J* = 8.3, 7.1, 1.4 Hz, 1H), 7.75 – 7.66 (m, 3H), 7.64 – 7.49 (m, 4H).

^{13}C {^1H} NMR (101 MHz, $CDCl_3$) δ 160.71, 144.10, 139.28, 137.16, 134.79, 130.57, 130.40, 129.67, 129.55, 128.95, 128.54, 127.74, 127.24, 123.51, 122.71, 121.99, 121.95.

NMR spectra are in accordance with the published data[19].

6-(4-Bromophenyl)phenanthridine (2e) and 9-bromo-6-phenylphenanthridine (3e)

The GP2 was followed with 50 mg (0.150 mmol) of **1e** (5 hours irradiation). Column chromatography using PE/EtOAc (96 : 4) provided 26 mg (0.078 mmol, 52 %, white powder) of **2e** and 9 mg (0.027 mmol, 18 %, white powder) of **3e**.

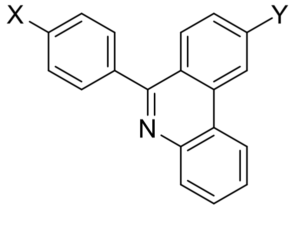

2e: X = Br, Y = H
3e: X = H, Y = Br

2e

^1H NMR (400 MHz, $CDCl_3$) δ 8.71 (d, *J* = 8.3 Hz, 1H), 8.62 (dd, *J* = 8.1, 1.3 Hz, 1H), 8.23 (dd, *J* = 8.1, 1.1 Hz, 1H), 8.12 – 8.01 (m, 1H), 7.87 (ddd, *J* = 8.3, 7.0, 1.3 Hz, 1H), 7.77 (ddd, *J* = 8.2, 7.1, 1.5 Hz, 1H), 7.74 – 7.67 (m, 3H), 7.67 – 7.60 (m, 3H).

^{13}C {^1H} NMR (101 MHz, $CDCl_3$) δ 159.95, 143.66, 138.61, 133.47, 131.60, 131.41, 130.71, 130.30, 128.95, 128.46, 127.26, 127.15, 124.90, 123.73, 123.14, 122.32, 121.96.

NMR spectra are in accordance with the published data[16].

3e

^1H NMR (400 MHz, $CDCl_3$) δ 8.84 (d, *J* = 1.9 Hz, 1H), 8.54 (dd, *J* = 8.2, 1.3 Hz, 1H), 8.30 – 8.20 (m, 1H), 7.97 (d, *J* = 8.8 Hz, 1H), 7.79 (ddd, *J* = 8.3, 7.1, 1.4 Hz, 1H), 7.76 – 7.66 (m, 4H), 7.62 – 7.46 (m, 3H).

^{13}C {^1H} NMR (101 MHz, $CDCl_3$) δ 160.81, 144.10, 139.25, 134.99, 130.56, 130.42 (2C), 129.67, 129.55, 128.95, 128.55, 127.27, 125.76, 125.17, 123.75, 122.52, 121.97.

IR ($CHCl_3$, ν cm^{-1}): 3059w, 3030vw, 1608w (sh), 1599s, 1562m, 1508w, 1480m, 1458m, 1442m, 1074m, 1002w, 824m, 760 vs (sh), 756vs, 730m, 697s, 539m.

EI MS: 334 (92 %), 333(60 %, M^+), 254 (100 %), 127 (49 %).

HRMS (APCI/QTOF) *m/z* $[M + H]^+$ calculated for $C_{19}H_{13}{}^{79}BrN$ 334.0225 found 334.0223.

Melting point: 145.1-148.4°C

6-(4-Trifluoromethylphenyl)phenanthridine (2f) and 6-phenyl-9-(trifluoromethyl)-phenanthridine (3f)

The GP2 was followed with 50 mg (0.154 mmol) of **1f** (5 hours irradiation). Column chromatography using PE/EtOAc (95 : 5) provided 25 mg (0.077 mmol, 50 %, white powder) of **2f** and 7 mg (0.022 mmol, 14 %, white powder) of **3f**.

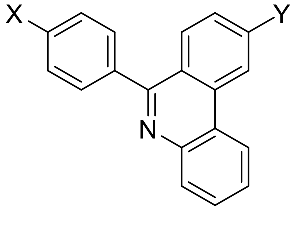

2f: X = CF_3, Y = H
3f: X = H, Y = CF_3

2f

^1H NMR (400 MHz, $CDCl_3$) δ 8.71 (d, *J* = 8.8 Hz, 1H), 8.62 (dd, *J* = 8.1, 1.5 Hz, 1H), 8.24 (dd, *J* = 8.0, 1.5 Hz, 1H), 8.06 (dd, J = 8.5, 0.8 Hz, 1H), 7.87 (ddd, *J* = 8.3, 7.0, 1.3 Hz, 1H), 7.77 (ddd, *J* = 8.3, 7.0, 1.5 Hz, 1H), 7.74 – 7.67 (m, 3H), 7.63 (ddd, *J* = 8.3, 7.0, 1.2 Hz, 1H), 7.58 – 7.51 (m, 2H).

^{13}C {^1H} NMR (101 MHz, $CDCl_3$) δ 159.68, 143.63, 143.32 (q, *J* = 1.2 Hz), 133.48, 130.83, 130.80 (q, *J* = 32.6 Hz), 130.38, 130.14, 129.04, 128.29, 127.36, 125.43 (q, *J* = 3.8 Hz), 124.82, 124.15 (q, *J* = 272.2 Hz), 123.82, 122.39, 122.00.

NMR spectra are in accordance with the published data[21].

3f

^{1}H NMR (400 MHz, $CDCl_3$) δ 8.98 (s, 1H), 8.65 (dd, *J* = 8.2, 1.5 Hz, 1H), 8.29 (dd, *J* = 8.1, 1.1 Hz, 1H), 8.26 (d, *J* = 8.6 Hz, 1H), 7.89 – 7.79 (m, 2H), 7.79 – 7.71 (m, 3H), 7.63 – 7.52 (m, 3H).

^{13}C {^{1}H} NMR (101 MHz, $CDCl_3$) δ 160.64, 144.10, 139.08, 133.26, 131.99 (q, *J* = 32.6 Hz), 130.60, 129.90, 129.74, 129.68, 129.07, 128.60, 127.61, 126.58, 123.94 (m, *J* = 272.8 Hz), 123.14, 123.02 (q, *J* = 3.2 Hz), 121.95, 119.79 (q, *J* = 4.3 Hz).

^{19}F {^{1}H} NMR (376 MHz, $CDCl_3$) δ -62.70.

IR ($CHCl_3$, ν cm^{-1}): 1611w, 1586m, 1569m, 1521w, 1495m, 1389w, 1348s (CF_3), 1298m, 1269m (CF_3), 1137s (CF_3), 1001w, 701m, 625w (ν_{as}-CF_3), 618w.

EI MS: 323 (100 %, M^+), 254 (50 %).

HRMS (APCI/QTOF) *m/z* $[M + H]^+$ calculated for $C_{20}H_{13}F_3N$ 324.0994; found 324.0993.

Melting point: 135.7-154.5°C

6-(4-Nitrophenyl)phenanthridine (2g)

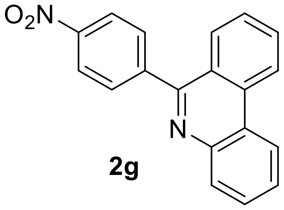

2g

The GP2 was followed with 50 mg (0.165 mmol) of **1g** (4 hours irradiation). Column chromatography using PE/EtOAc (94 : 6) provided 29 mg (0.097 mmol, 58 %) of **2g** as a yellow powder.

^{1}H NMR (400 MHz, $CDCl_3$) δ 8.76 (d, *J* = 8.3 Hz, 1H), 8.66 (dd, *J* = 8.1, 1.4 Hz, 1H), 8.44 (d, *J* = 8.7 Hz, 2H), 8.28 (d, *J* = 7.8 Hz, 1H), 7.99 (d, *J* = 8.3 Hz, 1H), 7.97 – 7.90 (m, 3H), 7.85 – 7.72 (m, 2H), 7.67 (ddd, *J* = 8.2, 7.1, 1.0 Hz, 1H).

^{13}C {^{1}H} NMR (101 MHz, $CDCl_3$) δ 158.63, 148.10, 145.69, 143.21, 133.63, 131.23, 130.91, 130.20, 129.31, 128.03, 127.80, 127.64, 124.51, 123.93, 123.68, 122.58, 122.08.

NMR spectra are in accordance with the published data[21].

6-(Naphthalen-2-yl)phenanthridine (2h) and 6-phenyl-5-aza[4]helicene (3h)

The GP2 was followed with 50 mg (0.163 mmol) of **1h** (4 hours irradiation). Column chromatography using PE/EtOAc (95 : 5) provided 11 mg (0.036 mmol, 22 %, white powder) of **2h** and 27 mg (0.088 mmol, 54 %, white powder) of **3h**.

2h

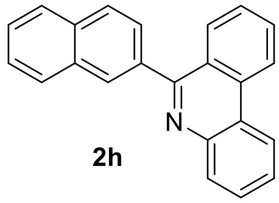

2h

^{1}H NMR (400 MHz, $CDCl_3$) δ 8.75 (d, *J* = 8.3 Hz, 1H), 8.66 (dd, *J* = 8.1, 1.5 Hz, 1H), 8.30 (d, *J* = 8.2 Hz, 1H), 8.24 (s, 1H), 8.17 (dd, *J* = 8.2, 0.7 Hz, 1H), 8.04 (d, *J* = 8.2 Hz, 1H), 8.00 – 7.93 (m, 2H), 7.93 – 7.84 (m, 2H), 7.79 (ddd, *J* = 8.2, 7.0, 1.5 Hz, 1H), 7.72 (ddd, *J* = 8.3, 7.0, 1.5 Hz, 1H), 7.63 (ddd, *J* = 8.3, 7.0, 1.2 Hz, 1H), 7.60 – 7.54 (m, 2H).

^{13}C {^{1}H} NMR (101 MHz, $CDCl_3$) δ 161.17, 143.48, 136.71, 133.66, 133.49, 133.20, 130.91, 130.10, 129.47, 129.20, 129.08, 128.57, 128.15, 127.84, 127.38 (2C), 127.19, 126.75, 126.51, 125.35, 123.84, 122.33, 122.04.

NMR spectra are in accordance with the published data[19].

3h

^{1}H NMR (400 MHz, $CDCl_3$) δ. 9.23 – 9.17 (m, 1H), 9.07 (ddd, *J* = 8.4, 1.5, 0.7 Hz, 1H), 8.40 (d, *J* = 8.2 Hz, 1H), 8.08 – 8.03 (m, 1H), 7.99 (d, *J* = 8.9 Hz, 1H), 7.89 (d, *J* = 8.8 Hz, 1H), 7.82 (ddd, *J* = 8.2, 6.9, 1.4 Hz, 1H), 7.79 – 7.70 (m, 5H), 7.63 – 7.51 (m, 3H).

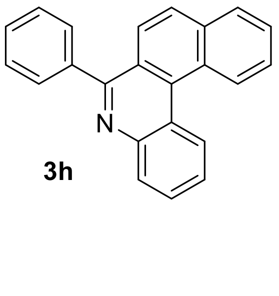

3h

^{13}C {^1H} NMR (101 MHz, $CDCl_3$) δ 160.28, 145.57, 139.91, 134.75, 132.78, 130.10, 128.99, 128.72, 128.64, 128.53, 128.44, 128.00, 127.85, 127.01, 126.83, 126.60, 124.96, 124.06, 123.75 (2 carbons are missing due to overlapping).

NMR spectra are in accordance with the published data[22].

6-(4-Nitrophenyl)-5-aza[4]helicene (3i)

The GP2 was followed with 50 mg (0.142 mmol) of **1i** (10 hours irradiation). Column chromatography using PE/EtOAc (88 : 12) provided 18 mg (0.051 mmol, 36 %) of **3i** as a yellow solid.

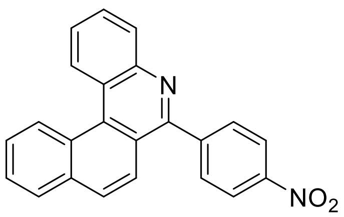

3i

^1H NMR (400 MHz, $CDCl_3$) δ 9.26 – 9.15 (m, 1H), 9.09 (d, *J* = 8.4 Hz, 1H), 8.45 (d, *J* = 8.8 Hz, 2H), 8.39 (d, *J* = 7.9 Hz, 1H), 8.10 – 8.06 (m, 1H), 7.99 – 7.90 (m, 3H), 7.89 – 7.76 (m, 5H).

^{13}C {^1H} NMR (101 MHz, $CDCl_3$) δ 157.64, 148.09, 146.07, 145.10, 134.83, 133.21, 131.28, 130.08, 129.02, 128.94, 128.72, 128.70, 128.56, 128.52, 127.45, 127.32, 127.15, 124.28, 123.80, 123.72, 123.10.

IR ($CHCl_3$, *ν* cm^{-1}): 1598m, 1509vs (ν-NO_2), 1476m, 1382w, 1352m (ν-NO_2), 1107m, 1051w, 1013w, 957vw, 851m, 799w, 745s (ν-NO_2), 700m.

NMR spectra are in accordance with published data[22].

6-(4-Nitrophenyl)-5-aza[5]helicene (3j)

The GP2 was followed with 50 mg (0.124 mmol) of **1j** (9 hours irradiation). Column chromatography using PE/EtOAc (90 : 10) provided 25 mg (0.062 mmol, 50 %) of **3j** as a yellow solid.

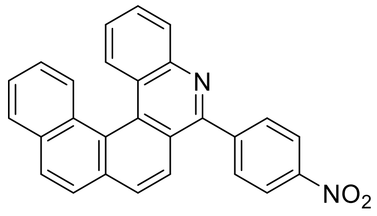

3j

^1H NMR (400 MHz, $CDCl_3$) δ 8.63 (d, *J* = 8.5 Hz, 1H), 8.52 (ddd, *J* = 8.5, 1.4, 0.7 Hz, 1H), 8.28 (dd, *J* = 8.3, 1.3 Hz, 1H), 8.07 (d, *J* = 8.6 Hz, 1H), 8.04 – 7.99 (m, 3H), 7.91 – 7.87 (m, 3H), 7.72 (ddd, *J* = 8.3, 6.9, 1.3 Hz, 1H), 7.61 (ddd, *J* = 8.0, 6.9, 1.2 Hz, 1H), 7.43 – 7.33 (m, 2H).

^{13}C {^1H} NMR (101 MHz, $CDCl_3$) δ 157.63, 148.10, 146.15, 144.47, 134.21, 132.81, 132.31, 131.44, 130.64, 130.02, 129.52, 129.13, 128.60, 128.15, 128.13, 127.60, 127.16, 125.81, 125.72, 125.45, 125.29, 125.02, 124.78, 124.24, 123.68.

IR ($CHCl_3$, *ν* cm^{-1}): 3059w, 1596m, 1519s (ν-NO_2), 1562m, 1345vs (ν-NO_2), 1107w, 1015w, 954w, 860w, 853m, 800w, 754m (ν-NO_2), 704m, 615m.

EI MS: 400 (100 %, M^+), 353 (53 %), 176 (30 %).

HRMS (APCI/QTOF) *m*/*z* $[M + H]^+$ calculated for $C_{27}H_{17}N_2O_2$ 401.1284; found 401.1286.

Melting point: 253.3-261.0°C

5-(4-Nitrophenyl)-6-aza-chrysene (3l)

The GP2 was followed with 50 mg (0.142 mmol) of **1l** (10 hours irradiation). Column chromatography using PE/EtOAc (85 : 15) provided 21 mg (0.060 mmol, 42 %) of **3l** as a yellow solid.

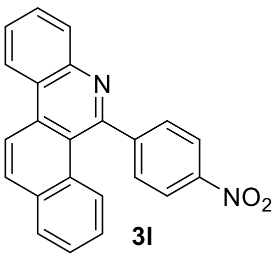

3l

^1H NMR (400 MHz, $CDCl_3$) δ 8.78 – 8.64 (m, 2H), 8.38 (d, *J* = 8.8 Hz, 2H), 8.30 – 8.21 (m, 2H), 8.00 (dd, *J* = 8.0, 1.5 Hz, 1H), 7.91 – 7.79 (m, 3H), 7.77 (ddd, *J* = 8.3, 7.0, 1.5 Hz, 1H), 7.70 (d, *J* = 8.7 Hz, 1H), 7.56 (ddd, *J* = 8.0, 7.0, 1.1 Hz, 1H), 7.33 – 7.19 (m, 1H).

^{13}C {^1H} NMR (101 MHz, $CDCl_3$) δ 156.56, 150.63, 147.73, 143.84, 134.64, 133.20, 132.79, 130.12, 129.95, 129.48, 129.44, 128.82, 128.01, 127.64, 126.81, 126.25, 124.29, 123.71, 122.56, 120.84, 119.93.

IR ($CHCl_3$, ν cm^{-1}): 1600m, 1517s (ν-NO_2), 1467w, 1345vs (ν-NO_2), 1107w, 1015w, 940m, 853m, 800w, 758s (ν-NO_2), 697m.

EI MS: 350 (99 %, M^+), 303 (100 %), 151 (26 %).

HRMS (APCI/QTOF) *m/z* $[M+H]^+$ calculated for $C_{23}H_{15}N_2O_2$ 351.1128; found 351.1129.

Melting point: 203.9-261.0°C

5-(4-Nitrophenyl)-6-aza-benzo[*g*]chrysene (3m)

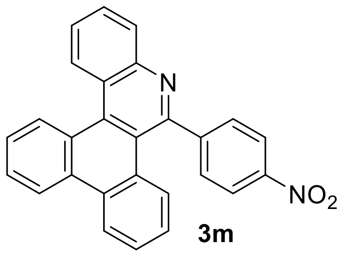

3m

The GP2 was followed with 50 mg (0.124 mmol) of **1m** (10 hours irradiation). Column chromatography using PE/EtOAc (90 : 10) provided 20 mg (0.050 mmol, 40 %) of **3m** as a yellow solid.

^1H NMR (500 MHz, $CDCl_3$) δ 8.88 (d, *J* = 8.2 Hz, 1H), 8.77 (d, *J* = 8.4 Hz, 1H), 8.74 (d, *J* = 8.0 Hz, 1H), 8.62 (d, *J* = 8.1 Hz, 1H), 8.35 – 8.21 (m, 3H), 8.00 – 7.91 (m, 2H), 7.88 – 7.83 (m, 1H), 7.83 – 7.74 (m, 2H), 7.69 (ddd, *J* = 8.3, 6.9, 1.3 Hz, 1H), 7.65 – 7.60 (m, 1H), 7.57 (ddd, *J* = 8.2, 7.1, 1.1 Hz, 1H), 7.23 (ddd, *J* = 8.3, 7.1, 1.1 Hz, 1H).

^{13}C {^1H} NMR (126 MHz, $CDCl_3$) δ 154.91, 149.73, 147.73, 145.75, 136.10, 132.49, 130.86, 130.64, 129.74, 129.34, 129.05, 129.03, 129.02, 128.25, 127.77, 127.42, 127.24, 127.20, 127.09, 126.30, 124.02, 123.84, 123.48, 123.29, 121.07.

IR ($CHCl_3$, ν cm^{-1}): 1596m, 1517s (ν-NO_2), 1487w, 1461m, 1390w, 1344vs (ν-NO_2), 1106w, 1047w, 1014w, 951vw, 853m, 810vw, 754s (ν-NO_2), 701w, 612m.

EI MS: 400 (100 %, M^+), 353 (66 %), 176 (27 %).

HRMS (APCI/QTOF) *m/z* $[M + H]^+$ calculated for $C_{27}H_{17}N_2O_2$ 401.1284; found 401.1285.

Melting point: 97.6-111.5°C

5-Phenyl-6-aza[4]helicene (3n)

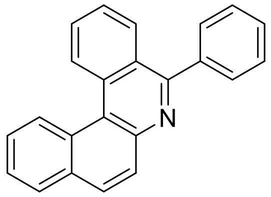

3n

The GP2 was followed with 50 mg (0.163 mmol) of **1n** (8 hours irradiation). Column chromatography using PE/EtOAc (94 : 6) provided 32 mg (0.105 mmol, 64 %) of **3n** as a white solid.

^1H NMR (400 MHz, $CDCl_3$) δ 9.19 (d, *J* = 8.5 Hz, 1H), 9.13 (d, *J* = 8.3 Hz, 1H), 8.28 (d, *J* = 7.7 Hz, 1H), 8.22 (d, *J* = 8.7 Hz, 1H), 8.15 – 8.05 (m, 2H), 7.95 – 7.84 (m, 3H), 7.81 – 7.74 (m, 1H), 7.74 – 7.52 (m, 5H).

^{13}C NMR (101 MHz, $CDCl_3$) δ 159.79, 142.64, 138.95, 133.11, 132.77, 129.68, 129.48, 129.12, 128.92, 128.30, 128.25, 128.13, 127.99, 127.97, 127.10, 126.37, 126.06, 126.01, 125.75, 125.62, 119.51.

IR ($CHCl_3$, ν cm^{-1}): 1619w, 1606w, 1578w (sh), 1569m (sh), 1516m, 1507m (sh), 1494w, 1444m, 1386m, 1180w, 1149w, 1031m, 618m

EI MS: 305 (66 %, M^+), 304 (100 %), 151 (16 %).

HRMS (APCI/QTOF) *m/z* $[M]^+$ calculated for $C_{23}H_{15}N$ 305.1199; found 305.1192.

Melting point: 89.5-92.7°C

5-Phenyl-6-aza[5]helicene (3o)

The GP2 was followed with 50 mg (0.140 mmol) of **1o** (31 hours irradiation). Column chromatography using PE/EtOAc (93 : 7) provided 36 mg (0.101 mmol, 72 %) of **3o** as a yellow solid.

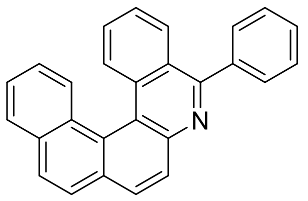

3o

^{1}H NMR (400 MHz, $CDCl_3$) δ 8.65 (d, *J* = 8.5 Hz, 1H), 8.58 (d, *J* = 7.8 Hz, 1H), 8.19 (d, *J* = 8.4 Hz, 1H), 8.16 (dd, *J* = 8.2, 1.5 Hz, 1H), 8.06 – 7.94 (m, 5H), 7.91 (d, *J* = 8.5 Hz, 1H), 7.68 – 7.60 (m, 2H), 7.60 – 7.55 (m, 2H), 7.52 (ddd, *J* = 8.3, 6.9, 1.4 Hz, 1H), 7.47 (ddd, *J* = 8.3, 6.9, 1.5 Hz, 1H), 7.36 (ddd, *J* = 8.4, 6.8, 1.4 Hz, 1H).

^{13}C {^{1}H} NMR (101 MHz, $CDCl_3$) δ 160.11, 144.01, 139.17, 134.45, 132.72, 132.22, 130.57, 130.14, 129.40, 129.02, 128.72, 128.66, 128.55, 128.28, 128.13, 127.87, 127.83, 127.60, 126.91, 126.55, 126.44, 126.26, 124.77, 124.67, 119.88.

IR ($CHCl_3$, ν cm^{-1}): 3054m, 1608m, 1513m, 1503m, 1445m, 1028w, 968m, 839vs, 793m, 765vs, 699vs, 620m, 546m.

EI MS: 355 (84 %, M^+), 354 (100 %), 276 (21 %), 176 (27 %).

HRMS (APCI/QTOF) *m/z* $[M + H]^+$ calculated for $C_{27}H_{18}N$ 356.1433; found 356.1432.

Melting point: 172.5-175.0°C

6-Phenyl-5-aza-chrysene (3q)

The GP2 was followed with 50 mg (0.163 mmol) of **1q** (28 hours irradiation). Column chromatography using PE/EtOAc (94 : 6) provided 12 mg (0.039 mmol, 24 %) of **3q** as a white solid.

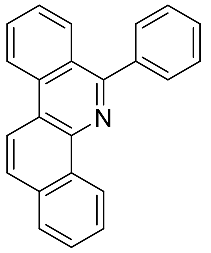

3q

^{1}H NMR (400 MHz, $CDCl_3$) δ 9.49 (d, *J* = 8.2 Hz, 1H), 8.78 (d, *J* = 8.4 Hz, 1H), 8.61 (d, *J* = 9.0 Hz, 1H), 8.31 (d, *J* = 8.3 Hz, 1H), 8.05 (d, *J* = 9.0 Hz, 1H), 7.99 (d, *J* = 8.3 Hz, 1H), 7.96 – 7.85 (m, 3H), 7.78 – 7.53 (m, 6H).

^{13}C NMR (101 MHz, $CDCl_3$) δ 159.41, 140.67, 140.22, 133.84, 133.37, 132.15, 130.41, 130.28, 128.68 (2C), 128.29, 127.65, 127.53, 127.36, 126.81, 126.77, 125.27, 125.22, 122.59, 120.42, 119.76.

NMR spectra are in accordance with published data[23].

6-Phenyl-5-aza-benzo[*g*]chrysene (3r)

The GP2 was followed with 50 mg (0.140 mmol) of **1r** (15 hours irradiation). Column chromatography using PE/EtOAc (95 : 5) provided 36 mg (0.101 mmol, 72 %) of **3r** as a white solid.

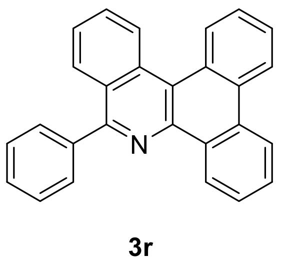

3r

^{1}H NMR (400 MHz, $CDCl_3$) δ 9.44 (dd, *J* = 6.3, 3.4 Hz, 1H), 9.02 (d, *J* = 8.6 Hz, 1H), 8.98 – 8.91 (m, 1H), 8.83 – 8.75 (m, 1H), 8.68 (dd, *J* = 6.2, 3.3 Hz, 1H), 8.33 (d, *J* = 8.4 Hz, 1H), 8.08 – 8.00 (m, 2H), 7.83 (ddd, *J* = 8.5, 6.8, 1.3 Hz, 1H), 7.80 – 7.68 (m, 4H), 7.68 – 7.54 (m, 4H).

^{13}C {^{1}H} NMR (101 MHz, $CDCl_3$) δ 158.93, 140.19, 139.66, 133.86, 131.16, 131.03, 130.85, 130.51, 129.94, 128.92, 128.88, 128.58, 128.42, 128.33, 128.10, 127.36, 127.07, 126.94, 126.41, 126.37, 126.31, 125.61, 123.68, 122.26, 118.40.

IR ($CHCl_3$, ν cm^{-1}): 1609w, 1572w(sh), 1506w, 1492w, 1451m, 1136w, 1044w, 1029w, 978m, 869w, 795m, 761vs, 701s, 617m, 436m.

EI MS: 355 (91 %, M^+), 354 (100 %), 176 (16 %).

HRMS (APCI/QTOF) *m/z* $[M + H]^+$ calculated for $C_{27}H_{18}N$ 356.1433; found 356.1429.

Melting point: 172.6-175.5°C

Phenanthrene-3-carbonitrile (4j)

The GP3 was followed with 840 mg (3.267 mmol) of 3-bromophenanthrene (**7o**) and 1460 mg of CuCN (16.30 mmol). Column chromatography using PE/EtOAc (90 : 10) provided 590 mg (2.90 mmol, 89 %) of **4j** as a white solid.

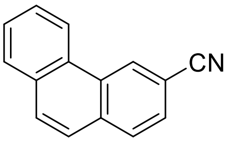

4j

^{1}H NMR (400 MHz, $CDCl_3$) δ 9.07 – 8.99 (m, 1H), 8.70 – 8.62 (m, 1H), 7.99 – 7.93 (m, 2H), 7.91 (d, *J* = 8.8 Hz, 1H), 7.82 – 7.65 (m, 4H).

^{13}C {^{1}H} NMR (101 MHz, $CDCl_3$) δ 134.26, 132.22, 130.36, 129.99, 129.49, 129.35, 128.90, 128.21, 127.95, 127.82, 127.72, 126.05, 122.56, 119.55, 109.80.

NMR spectra are in accordance with the published data[24]

Benzo[*c*]phenanthrene-2-carbonitrile (4k)

The GP3 was followed with 1000 mg (3.26 mmol) of 2-bromobenzo[c]phenanthrene (**7p**) and 1460 mg of CuCN (16.28 mmol). Column chromatography using PE/EtOAc (85 : 15) provided 620 mg (2.45 mmol, 75 %) of **4k** as a white solid.

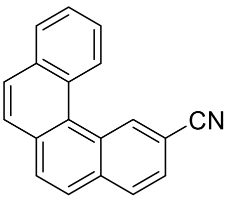

4k

^{1}H NMR (500 MHz, $CDCl_3$) δ 9.46 (s, 1H), 8.96 (d, *J* = 8.4 Hz, 1H), 8.09 (d, *J* = 8.3 Hz, 1H), 8.07 (dd, *J* = 8.0, 1.1 Hz, 1H), 7.99 (dd, *J* = 8.5, 3.8 Hz, 2H), 7.93 (d, *J* = 8.6 Hz, 1H), 7.86 (d, *J* = 8.5 Hz, 1H), 7.81 – 7.76 (m, 2H), 7.71 (ddd, *J* = 8.0, 7.0, 1.1 Hz, 1H).

^{13}C {^{1}H} NMR (126 MHz, $CDCl_3$) δ 135.22, 133.73, 133.42, 131.55, 130.17, 129.66, 129.63, 129.56, 128.86, 128.81, 127.41, 127.19, 126.92, 126.81 (2C), 126.72, 126.47, 119.80, 109.43.

IR ($CHCl_3$, ν cm^{-1}): 2229m (CN), 1695vw, 1605w, 1519w, 1422w, 1227w, 1137w, 1109w, 1045w, 956w, 870w, 849vs, 820w, 614m, 575m, 541m.

EI MS: 253 (100 %, M^+), 225 (11 %), 112 (10 %).

HRMS (APCI/QTOF) *m/z* $[M + H]^+$ calculated for $C_{19}H_{12}N$ 254.0964; found 254.0968.

Melting point: 175.5-178.9°C

(4-Bromophenyl)(naphthalen-2-yl)methanone (5i)

The GP4 was followed with 500 mg (3.26 mmol) of 2-naphthonitrile (**4i**) and 2300 mg (9.79 mmol) of 1,4-dibromobenzene. Column chromatography using PE/EtOAc (95 : 5) provided 750 mg (2.41 mmol, 74 %) of **5i** as a white solid.

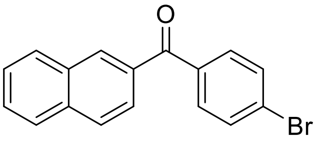

5i

^{1}H NMR (400 MHz, $CDCl_3$) δ 8.23 (s, 1H), 7.99 – 7.88 (m, 4H), 7.74 (d, *J* = 8.6 Hz, 2H), 7.69 – 7.54 (m, 4H).

^{13}C {^{1}H} NMR (101 MHz, $CDCl_3$) δ 195.64, 136.61, 135.33, 134.40, 132.21, 131.79, 131.66, 131.59, 129.40, 128.50, 128.47, 127.84, 127.44, 126.94, 125.55.

NMR spectra are in accordance with published data[25].

(4-Bromophenyl)(phenathren-3-yl)methanone (5j)

The GP4 was followed with 300 mg (1.476 mmol) of **4j** and 1045 mg (4.43 mmol) of 1,4-dibromobenzene. Column chromatography using PE/EtOAc (95 : 5) provided 430 mg (1.190 mmol, 81 %) of **5j** as a white solid.

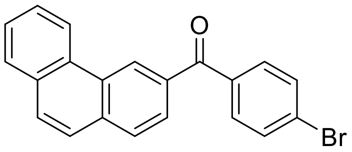

5j

^{1}H NMR (400 MHz, $CDCl_3$) δ 9.11 (s, 1H), 8.65 (d, *J* = 8.1 Hz, 1H), 8.00 (s, 2H), 7.94 (dd, *J* = 7.8, 1.4 Hz, 1H), 7.89 (d, *J* = 8.8 Hz, 1H), 7.83 – 7.76 (m, 3H), 7.73 – 7.62 (m, 4H).

^{13}C {^{1}H} NMR (101 MHz, $CDCl_3$) δ 195.73, 136.69, 134.77, 134.71, 132.20, 131.70, 131.68, 130.47, 129.74, 129.60, 128.83, 128.78, 127.52, 127.32, 127.27, 126.97, 126.27, 125.72, 122.72.

IR ($CHCl_3$, ν cm^{-1}): 1658s (C=O), 1612m, 1599w, 1587s, 1565w, 1514w, 1483w, 1426w, 1395m, 1312m-s, 1262ws, 1239m, 1177m, 1070m, 1012m-s, 950m, 855m, 848m, 807m, 623w.

EI MS: 360 (71 %, M^+), 205 (100 %), 177 (70 %), 151 (23 %), 76 (16 %).

HRMS (APCI/QTOF) *m/z* $[M + H]^+$ calculated for $C_{21}H_{14}{}^{79}BrO$ 361.0222; found 361.0224.

Melting point: 93.9-94°C

Benzo[*c*]phenanthren-2-yl(4-bromophenyl)methanone (5k)

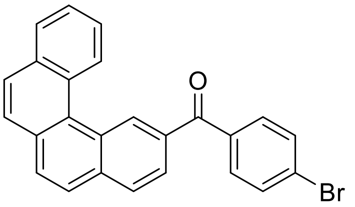

5k

The GP4 was followed with 500 mg (1.974 mmol) of **4k** and 1400 mg (5.930 mmol) of 1,4-dibromobenzene. Column chromatography using PE/EtOAc (90 : 10) provided 655 mg (1.59 mmol, 81%) of **5k** as a white solid.

^{1}H NMR (400 MHz, $CDCl_3$) δ 9.51 (d, *J* = 0.8 Hz, 1H), 9.00 – 8.91 (m, 1H), 8.11 (d, *J* = 8.3 Hz, 1H), 8.07 – 8.00 (m, 2H), 8.00 – 7.92 (m, 3H), 7.85 (d, *J* = 8.5 Hz, 1H), 7.83 – 7.78 (m, 2H), 7.73 – 7.67 (m, 2H), 7.67 – 7.58 (m, 2H).

^{13}C {1H} NMR (101 MHz, $CDCl_3$) δ 195.94, 136.79, 135.78, 134.52, 133.63, 131.68, 131.56, 131.37, 131.34, 129.86, 129.60, 129.38, 128.83, 128.71, 128.22, 127.98, 127.55, 127.43, 126.92, 126.65 (2C), 126.38, 125.98.

IR ($CHCl_3$, ν cm^{-1}): 1656m (C=O), 1622w, 1585s, 1565w, 1480w, 1395m, 1279s, 1271s, 1249m, 1176w, 1106w, 1070m, 1011m, 927m, 850vs, 830m, 795m, 755vs, 697w, 509m, 469w.

EI MS: 410 (88 %, M^+), 255 (30 %), 226 (100 %).

HRMS (APCI/QTOF) *m/z* $[M]^+$ calculated for $C_{25}H_{15}{}^{81}BrO$ 412.0283; found 412.0280.

Melting point: 132.1-135.0°C

(4-Bromophenyl)(naphthalen-1-yl)methanone (5l)

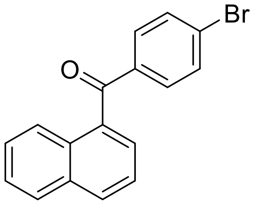

5l

The GP4 was followed with 500 mg (3.26 mmol) of 1-naphthonitrile (**4l**) and 2300 mg (9.79 mmol) of 1,4-dibromobenzene. Column chromatography using PE/EtOAc (90 : 10) provided 780 mg (2.51 mmol, 77 %) of **5l** as a white solid.

^{1}H NMR (400 MHz, $CDCl_3$) δ 7.94 (d, *J* = 8.3 Hz, 1H), 7.91 (d, *J* = 8.2 Hz, 1H), 7.65 (d, J = 8.6 Hz, 1H), 7.63 – 7.58 (m, 2H), 7.56 – 7.47 (m, 4H), 7.45 – 7.39 (m, 2H).

^{13}C {^{1}H} NMR (101 MHz, $CDCl_3$) δ 176.84, 138.19, 137.54, 133.51, 131.59, 130.14, 129.93, 129.41, 128.43, 126.80, 126.36, 125.74, 125.36, 125.25, 125.14.

NMR spectra are in accordance with published data[25].

(4-Bromophenyl)(phenathren-9-yl)methanone (5m)

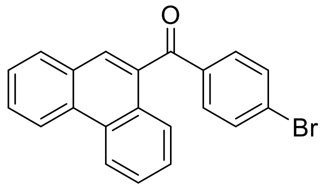

5m

The GP4 was followed with 500 mg (2.46 mmol) of phenanthrene-9-carbonitrile (**4m**) and 1740 mg (7.38 mmol) of 1,4-dibromobenzene. Column chromatography using PE/EtOAc (94 : 6) provided 730 mg (2.02 mmol, 82 %) of **5m** as a white solid.

^{1}H NMR (400 MHz, $CDCl_3$) δ 8.80 – 8.76 (m, 1H), 8.74 (d, *J* = 8.1 Hz, 1H), 8.13 – 8.05 (m, 1H), 7.90 (dd, *J* = 7.9, 1.3 Hz, 1H), 7.85 – 7.74 (m, 4H), 7.71 (ddd, *J* = 8.4, 7.0, 1.3 Hz, 1H), 7.68 – 7.57 (m, 4H).

$^{13}C\{^1H\}$ NMR (101 MHz, $CDCl_3$) δ 196.78, 136.94, 134.77, 131.87, 131.84, 131.37, 130.62, 129.94, 129.54, 129.34, 129.11, 128.65, 128.51, 127.30 (2C), 127.23, 126.47, 123.01, 122.73.

IR ($CHCl_3$, ν cm^{-1}): 1659vs (C=O), 1614w, 1583s, 1566m, 1481m, 1445s, 1396m, 1254vs, 1173m, 1105w, 1070s, 1042m, 952w, 847s, 752s, 790w, 752s, 685w, 515m, 458w.

EI MS: 362 (100 %, M^+), 360 (96 %), 281 (47 %), 252 (23 %), 205 (80 %), 177 (73 %), 126 (24 %).

HRMS (APCI/QTOF) *m/z* $[M + H]^+$ calculated for $C_{21}H_{14}{}^{79}BrO$ 361.0222; found 361.0220.

Melting point: 111.7-114.3°C

Naphthalen-2-yl(4-nitrophenyl)methanone (6i)

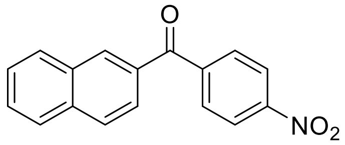

6i

The GP5 was followed with 650 mg (2.09 mmol) of **5i**, 534 mg (6.27 mmol) of KNO_2, and 600 mg (3.15 mmol) of CuI. Column chromatography using PE/EtOAc (94 : 6) provided 325 mg (1.17 mmol, 56 %) of **6i** as a yellow powder.

^1H NMR (400 MHz, $CDCl_3$) δ 8.36 (d, *J* = 8.7 Hz, 2H), 8.23 (s, 1H), 8.04 – 7.87 (m, 6H), 7.65 (ddd, *J* = 8.2, 6.9, 1.3 Hz, 1H), 7.59 (ddd, *J* = 8.1, 6.9, 1.2 Hz, 1H).

$^{13}C\{^1H\}$ NMR (101 MHz, $CDCl_3$) δ 194.68, 149.69, 143.14, 135.55, 133.46, 132.33, 132.09, 130.61, 129.47, 128.93, 128.74, 127.84, 127.13, 125.12, 123.50.

NMR spectra are in accordance with published data[26].

(4-Nitrophenyl)(phenathren-3-yl)methanone (6j)

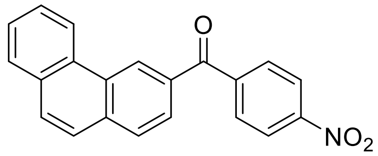

6j

The GP5 was followed with 280 mg (0.78 mmol) of **5j**, 200 mg (2.35 mmol) of KNO_2, and 221 mg (1.16 mmol) of CuI. Column chromatography using PE/EtOAc (95 : 5) provided 131 mg (0.40 mmol, 51 %) of **6j** as a yellow powder.

^1H NMR (400 MHz, $CDCl_3$) δ 9.13 (s, 1H), 8.68 – 8.55 (m, 1H), 8.39 (d, *J* = 8.8 Hz, 2H), 8.08 – 7.98 (m, 4H), 7.95 (dd, *J* = 7.7, 1.7 Hz, 1H), 7.91 (d, *J* = 8.7 Hz, 1H), 7.81 (d, *J* = 8.8 Hz, 1H), 7.74 – 7.63 (m, 2H).

$^{13}C\{^1H\}$ NMR (101 MHz, $CDCl_3$) δ 194.86, 149.84, 143.30, 135.16, 133.84, 132.23, 130.79, 130.38, 130.28, 129.70, 129.05, 128.92, 127.50, 127.47, 126.80, 126.19, 126.06, 123.62, 122.62.

IR ($CHCl_3$, ν cm^{-1}): 1662s (C=O), 1612m, 1528s (ν_{as}-NO_2), 1507m (sh), 1426w, 1351s (NO_2), 1262ws, 1239m (sh), 1176w, 1108w, 1040w, 1015w, 978w (sh), 955m, 943m, 867m, 850m, 841s (β_s-NO_2) 809m, 505w.

EI MS: 327 (100 %, M^+), 205 (69 %), 177 (54 %).

HRMS (APCI/QTOF) *m/z* $[M]^+$ calculated for $C_{21}H_{13}NO_3$ 327.0889; found 327.0888.

Melting point: 145.9-152.0°C

Benzo[*c*]phenanthren-2-yl(4-nitrophenyl)methanone (6k)

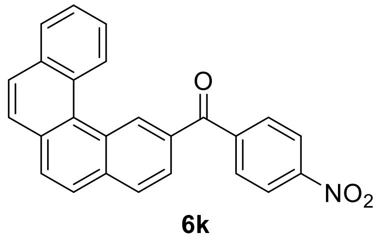

6k

The GP5 was followed with 600 mg (1.46 mmol) of **5k**, 373 mg (4.38 mmol) of KNO_2, and 417 mg (2.19 mmol) of CuI. Column chromatography using PE/EtOAc (90 : 10) provided 260 mg (0.69 mmol, 47 %) of **6k** as a yellow powder.

^1H NMR (400 MHz, $CDCl_3$) 9.51 (s, 1H), 8.90 (d, J = 8.1 Hz, 1H), 8.41 (d, J = 8.9 Hz, 2H), 8.15 (d, J = 8.3 Hz, 1H), 8.10 – 7.94 (m, 7H), 7.87 (d, J = 8.6 Hz, 1H), 7.67 – 7.57 (m, 2H).

$^{13}C\{^1H\}$ NMR (101 MHz, $CDCl_3$) δ 195.07, 149.76, 143.56, 136.17, 133.70, 133.56, 131.95, 131.44, 130.62, 130.13, 129.73, 129.39, 129.15, 128.82, 128.40, 127.98, 127.38, 126.88, 126.66, 126.64, 126.54, 125.71, 123.60.

IR ($CHCl_3$, ν cm^{-1}): 1645s (C=O), 1598m, 1516s (ν-NO_2), 1401m, 1346m (ν-NO_2), 1150m, 1043w, 1009w, 930m, 851vs, 803m, 756s (ν-NO_2), 698m, 621m.

EI MS: 377 (100 %, M^+), 255 (16 %), 226 (48 %).

HRMS (APCI/QTOF) m/z $[M]^+$ calculated for $C_{25}H_{15}NO_3$ 377.1046; found 377.1039.

Melting point: 216.0-220.3°C

Naphthalen-1-yl(4-nitrophenyl)methanone (6l)

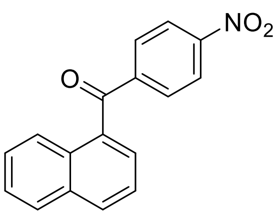

6l

The GP5 was followed with 600 mg (1.93 mmol) of **5l**, 492 mg (5.78 mmol) of KNO_2, and 550 mg (2.89 mmol) of CuI. Column chromatography using PE/EtOAc (90 : 10) provided 283 mg (1.02 mmol, 53 %) of **6l** as a white solid).

^1H NMR (400 MHz, $CDCl_3$) δ 8.31 (d, J = 8.7 Hz, 2H), 8.20 – 8.13 (m, 1H), 8.08 (d, J = 8.0 Hz, 1H), 8.01 (d, J = 8.7 Hz, 2H), 7.99 – 7.93 (m, 1H), 7.63 – 7.50 (m, 4H).

$^{13}C\{^1H\}$ NMR (101 MHz, $CDCl_3$) δ 196.01, 150.23, 143.35, 134.66, 133.82, 132.61, 131.14, 130.78, 128.92, 128.62, 127.88, 126.84, 125.37, 124.26, 123.64.

NMR spectra are in accordance with published data[27].

(4-Nitrophenyl)(phenathren-9-yl)methanone (6m)

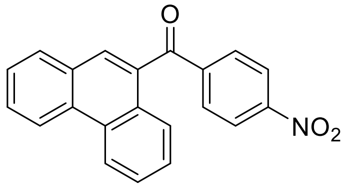

6m

The GP5 was followed with 300 mg (0.83 mmol) of **5m**, 212 mg (2.49 mmol) of KNO_2, and 238 mg (1.25 mmol) of CuI. Column chromatography using PE/EtOAc (93 : 7) provided 122 mg (0.37 mmol, 45 %) of **6m** as a yellow solid.

^1H NMR (400 MHz, $CDCl_3$) δ 8.79 (d, J = 8.3 Hz, 1H), 8.75 (d, J = 8.4 Hz, 1H), 8.33 (d, J = 8.9 Hz, 2H), 8.21 – 8.13 (m, 1H), 8.08 (d, J = 8.9 Hz, 2H), 7.94 – 7.88 (m, 1H), 7.86 (s, 1H), 7.80 (ddd, J = 8.4, 7.1, 1.4 Hz, 1H), 7.74 (ddd, J = 8.3, 7.0, 1.3 Hz, 1H), 7.70 – 7.65 (m, 1H), 7.63 (ddd, J = 8.2, 7.0, 1.2 Hz, 1H).

$^{13}C\{^1H\}$ NMR (101 MHz, $CDCl_3$) δ 195.88, 150.28, 143.15, 133.76, 131.68, 131.14, 130.77, 130.69, 129.73, 129.65, 129.04, 128.80, 127.52, 127.50, 127.37, 126.32, 123.68, 123.09, 122.77.

IR ($CHCl_3$, ν cm^{-1}): 1663s (C=O), 1602m, 1525vs (ν-NO_2), 1450m, 1446m, 1406m, 1347m (ν-NO_2), 1194m, 1147m, 1105m, 1043w, 955m, 1013w, 838vs, 791m, 749s (ν-NO_2), 706m, 616w.

EI MS: 327 (100 %, M^+), 205 (53 %), 177 (53 %).

HRMS (APCI/QTOF) m/z $[M]^+$ calculated for $C_{21}H_{13}NO_3$ 327.0889; found 327.0895.

Melting point: 129.4-131.0°C

***N,N′*-(Naphthalene-2,7-diyl)bis(1,1-diphenylmethanimine) (9a)**

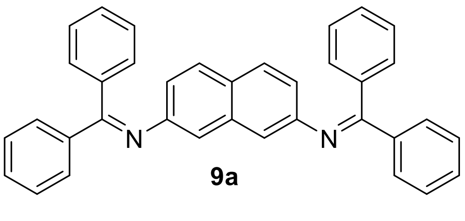

9a

The GP6 was followed with 1000 mg (2.36 mmol) of naphthalene-2,7-diylbis(trifluoromethanesulfonate) (**8a**) and 790 µL (4.71 mmol, 2 equiv) of benzophenone imine. Column chromatography using PE/EtOAc (92 : 8) provided 1010 mg (2.07 mmol, 88 %) of **9a** as a yellow powder.

^{1}H NMR (500 MHz, $CDCl_3$) δ 7.76 (d, *J* = 7.0 Hz, 4H), 7.52 – 7.44 (m, 4H), 7.41 (dd, *J* = 8.3, 6.8 Hz, 4H), 7.25 – 7.17 (m, 6H), 7.12 (dd, *J* = 8.1, 1.5 Hz, 4H), 6.89 (d, *J* = 2.0 Hz, 2H), 6.79 (dd, *J* = 8.6, 2.0 Hz, 2H).
^{13}C NMR (126 MHz, $CDCl_3$) δ 168.35, 149.00, 139.72, 136.13, 134.32, 130.71, 129.49, 129.34, 128.61, 128.18, 127.91, 127.88, 127.12, 120.41, 117.09.

IR ($CHCl_3$, *ν* cm^{-1}): 1613m (C=N), 1596m, 1576m, 1573m, 1447m, 1291m, 1249m, 1001w, 983w, 699vs, 617w.

EI MS: 486 (100 %, M^+), 409 (13 %).

HRMS (APCI/QTOF) *m/z* $[M + H]^+$ calculated for $C_{36}H_{27}N_2$ 487.2168; found 487.2158.

Melting point: 196.0-196.6°C

***N,N′*-(Phenanthrene-3,6-diyl)bis(1,1-diphenylmethanimine) (9b)**

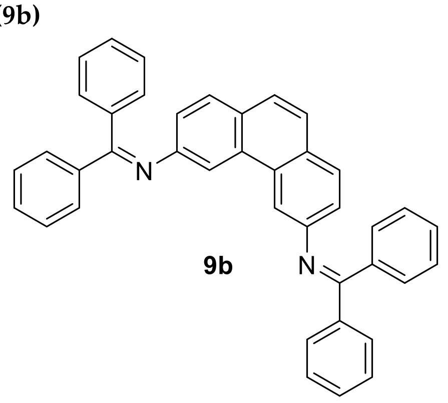

9b

The GP6 was followed with 400 mg (1.190 mmol) of 3,6-dibromobenzophenanthrene (**8b**) and 400 μL (2.380 mmol, 2 equiv) of benzophenone imine. Column chromatography using PE/EtOAc (90 : 10) provided 610 mg (1.136 mmol, 95 %) of **9b** as a yellow solid.

^{1}H NMR (400 MHz, $CDCl_3$) δ 7.87 (s, 2H), 7.84 – 7.77 (m, 4H), 7.58 (d, *J* = 8.4 Hz, 2H), 7.54 – 7.39 (m, 8H), 7.25 – 7.13 (m, 10H), 6.92 (d, *J* = 8.4 Hz, 2H).
^{13}C NMR (101 MHz, $CDCl_3$) δ 168.64, 149.46, 139.69, 136.17, 130.80, 130.65, 129.60, 129.39, 128.70, 128.64, 128.51, 128.24, 128.00, 125.14, 121.05, 114.25.

IR ($CHCl_3$, *ν* cm^{-1}): 3055m, 1620m (C=N), 1610s, 1598s, 1507m, 1446m, 1290m, 1272m, 1187w, 1140m, 1074w, 1028w, 959m, 843m, 784m, 757s, 695vs, 635w, 529vw.

EI MS: 536 (100 %, M^+).

HRMS (APCI/QTOF) *m/z* $[M + H]^+$ calculated for $C_{40}H_{29}N_2$ 537.2325; found 537.2307.

Melting point: 195.7-197.9°C

1,1-Diphenyl-*N*-(5-phenylbenzo[*a*]phenanthridin-11-yl)methanimine (10a)

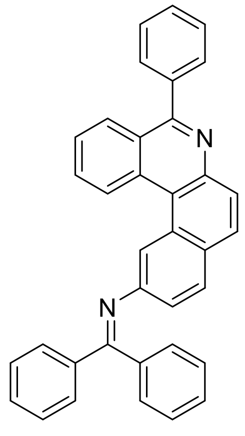

10a

The GP2 was followed with 50 mg (0.103 mmol) of **9a** (8 hours irradiation). Column chromatography using PE/EtOAc (84 : 16) provided 31 mg (0.064 mmol, 62 %) of **10a** as a yellow solid.

^{1}H NMR (400 MHz, $CDCl_3$) δ 8.22 (s, 1H), 8.16 (d, *J* = 9.7 Hz, 2H), 8.04 (d, *J* = 8.7 Hz, 1H), 7.99 – 7.90 (m, 2H), 7.89 – 7.78 (m, 4H), 7.65 (t, *J* = 8.4 Hz, 1H), 7.61 – 7.50 (m, 5H), 7.50 – 7.43 (m, 2H), 7.42 – 7.28 (m, 6H).
^{13}C NMR (101 MHz, $CDCl_3$) δ 168.69, 160.22, 150.18, 143.42, 139.81, 139.49, 136.19, 133.59, 130.93, 130.12, 130.06, 129.94, 129.88, 129.70, 129.56, 129.36, 129.34, 128.93, 128.73, 128.44, 128.42, 128.29, 128.23, 127.07, 126.56, 126.18, 125.84, 122.17, 119.78, 117.60.

IR ($CHCl_3$, *ν* cm^{-1}): 1613m (C=N), 1596m, 1569m, 1573w (sh), 1526w (sh), 1493m, 1385w, 1290m, 1245m-w, 1001w, 701s.

EI MS: 484 (100 %, M^+), 407 (11 %).

HRMS (APCI/QTOF) *m/z* $[M + H]^+$ calculated for $C_{36}H_{25}N_2$ 485.2012; found 485.2009.

Melting point: 202.0-202.9°C

1,1-Diphenyl-*N*-(1-phenylnaphtho[1,2-*a*]phenanthridin-9-yl)methanimine (10b)

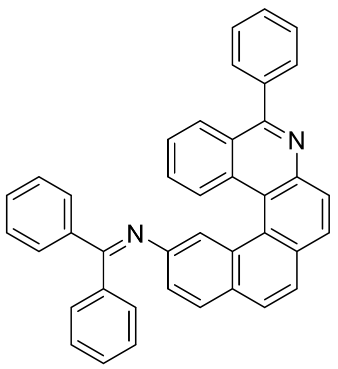

10b

The GP2 was followed with 50 mg (0.093 mmol) of **9b** (40 hours irradiation). Column chromatography using PE/EtOAc (89 : 11) provided 18 mg (0.03 mmol, 36 %) of **10b** as a yellow solid.

^{1}H NMR (400 MHz, $CDCl_3$) δ 8.75 – 8.62 (m, 1H), 8.28 (d, *J* = 2.0 Hz, 1H), 8.15 (d, *J* = 8.4 Hz, 1H), 8.11 (dd, *J* = 7.3, 2.3 Hz, 1H), 8.06 – 7.93 (m, 3H), 7.82 (d, *J* = 8.6 Hz, 1H), 7.77 (d, *J* = 8.6 Hz, 1H), 7.74 – 7.66 (m, 3H), 7.65 – 7.59 (m, 2H), 7.59 – 7.43 (m, 4H), 7.43 – 7.34 (m, 2H), 7.31 – 7.26 (m, 2H), 7.25 – 7.19 (m, 2H), 6.75 (dd, *J* = 8.5, 2.0 Hz, 1H).

^{13}C NMR (101 MHz, $CDCl_3$) 168.41, 159.85, 148.12, 139.50, 137.58, 136.20, 134.52, 132.60, 132.39, 131.38, 130.84, 130.18, 130.04, 129.83, 129.48, 129.35, 129.29, 129.13, 128.79, 128.57, 128.47, 128.39, 128.25, 128.21, 128.03, 127.72, 127.53, 127.05, 125.95, 125.10, 124.80, 121.06, 120.47, 120.14.

IR ($CHCl_3$, ν cm^{-1}): 1658m (C=N), 1607m, 1508m, 1446m, 1277m, 1142m, 1074m, 1028m, 958m, 850m, 840m, 762s, 697vs, 639m,

EI MS: 534 (19 %, M^+) 455 (20 %), 368 (100 %), 353 (80 %), 228 (27 %), 176 (32 %).

HRMS (APCI/QTOF) *m*/*z* $[M + H]^+$ calculated for $C_{40}H_{27}N_2$ 535.2168; found 535.2156.

Melting point: 105.0-112.7°C

2. X-ray crystallographic studies

Diffraction data of **1g**, **2g**, **1l**, **3j**, **3l** and **3r** were collected on a Bruker D8 VENTURE Kappa Duo PHOTON 100 CMOS with the monochromated Mo/Cu-Kα radiation. The structures were solved by direct methods (SHELXT) and refined by full-matrix least-squares on F^2 values (CRYSTALS). All heavy atoms were refined anisotropically. Hydrogen atoms were usually localized from the expected geometry and difference electron density maps and were refined isotropically. ORTEP-3 was used for structure presentation. The crystallographic data for the structures reported in this paper have been deposited with the Cambridge Crystallographic Data Centre as a supplementary publication; CCDC 2017712–2017717. Copies of the data can be obtained free of charge on application to CCDC, e-mail: deposit@ccdc.cam.ac.uk.

X-ray data of ***N*-phenyl-4-nitrobenzophenone imine 1g**: $C_{19}H_{14}N_2O_2$, M=302.33 g.mol^{-1}, monoclinic system, space group *P2₁/c*, a=11.50220(2), b=9.57550(2), c=14.27590(2) Å, β=106.092(3) °, Z=4, V=1510.73(2) Å^3, Dc=1.33 g.cm^{-3}, μ(Mo Kα)=0.09 mm^{-1}, T=150 K, crystal dimensions of 0.14 x 0.28 x 0.71 mm, yellow bar. The structure converged to the final R=0.0423 and R_w=0.0960 using 2645 independent reflections for 264 refined parameters (θ_{max}=27.49°). CCDC registration number 2017712.

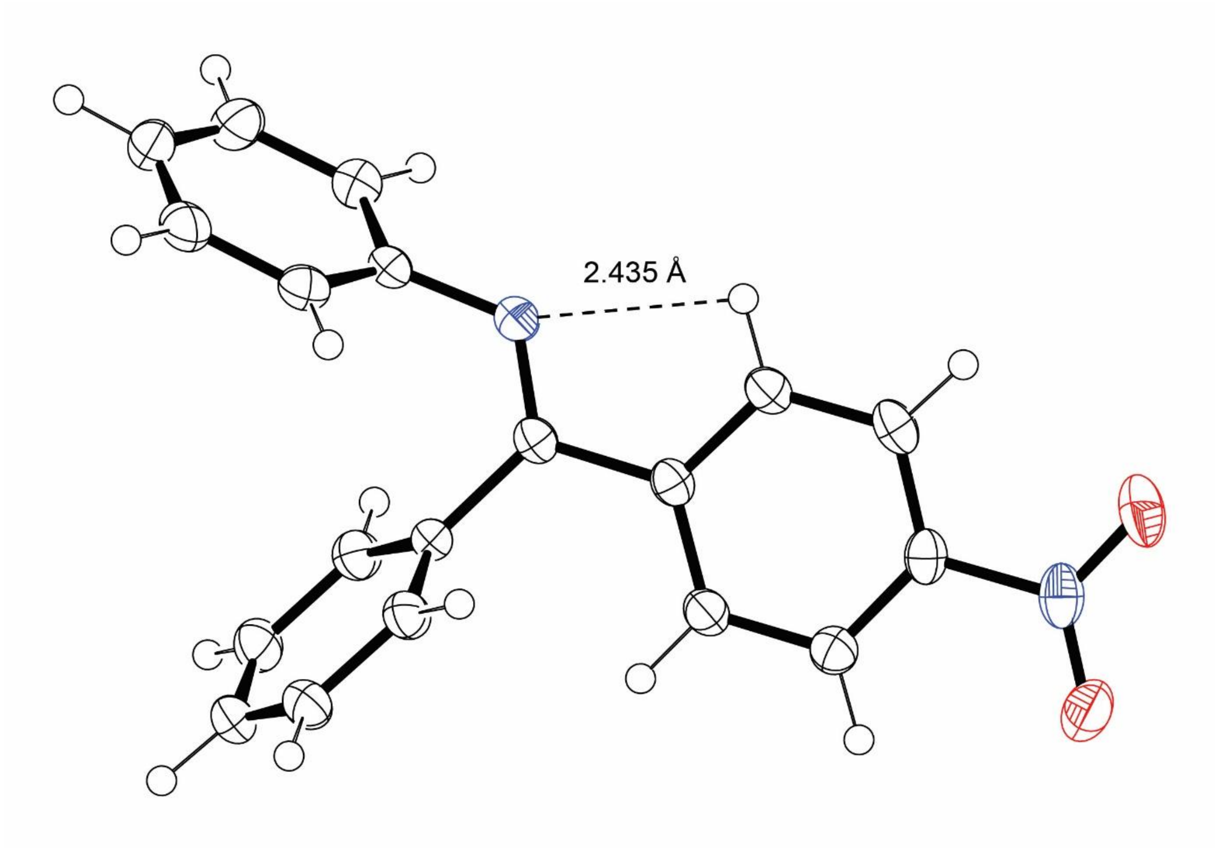

Figure S1. ORTEP projection of the crystal structure of *E*-**1g.**

X-ray data of **(*Z*)-1-(naphthalen-1-yl)-1-(4-nitrophenyl)-*N*-phenylmethanimine 1l**: $C_{23}H_{16}N_2O_2$, M=352.39 g.mol^{-1}, triclinic system, space group *P-1*, a=8.7721(5), b=12.8370(7), c=10.83260(2) Å, α=117.642(2), β= 102.931(2), γ= 99.793(2) °, Z=2, V=854.10(2) Å^3, Dc=1.32 g.cm^{-3}, μ(Cu Kα)=0.78 mm^{-1}, T=120 K, crystal dimensions of 0.04 x 0.06 x 0.33 mm, yellow bar. The structure converged to the final R=0.0608 and R_w=0.01224 using 4738 independent reflections for 496 refined parameters (θ_{max}=72.49°). CCDC registration number 2017713.

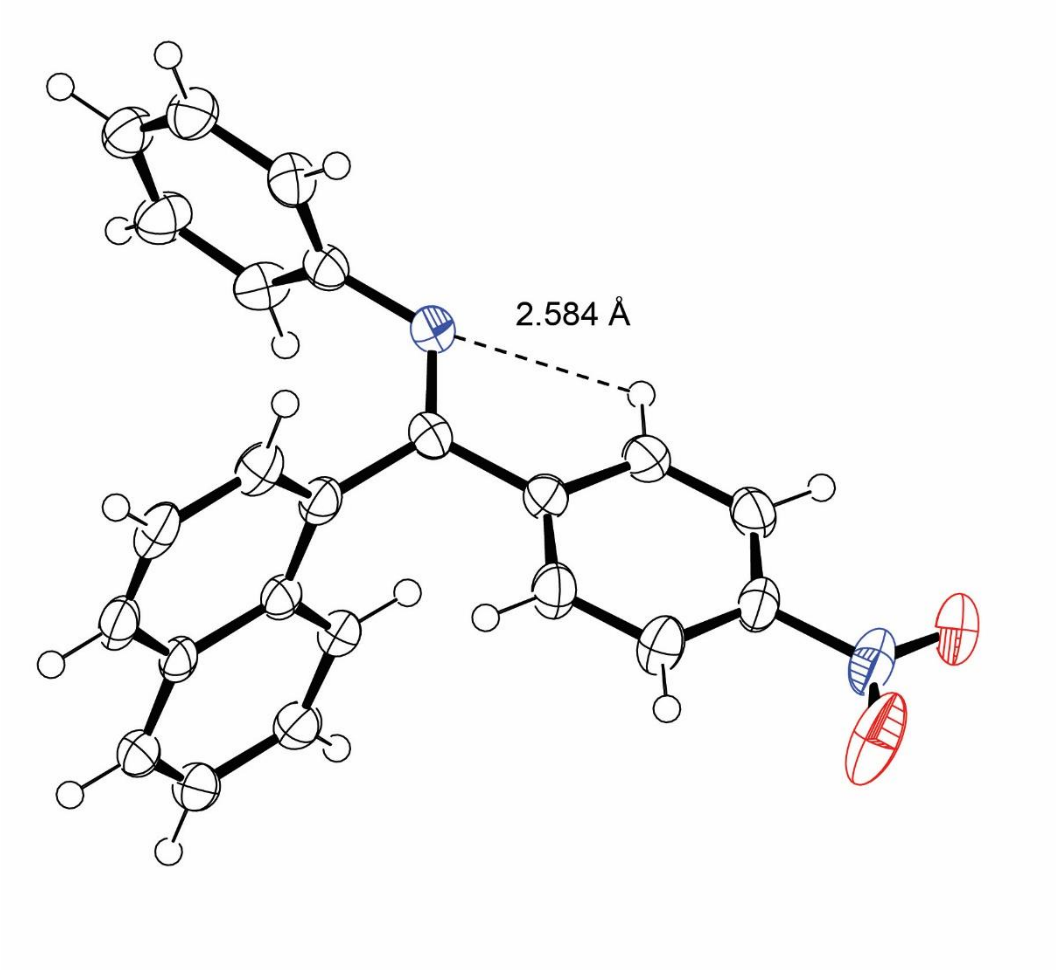

Figure S2. ORTEP projection of the crystal structure of **1l.**

X-ray data of **6-(4-nitrophenyl)phenanthridine 2g**: $C_{19}H_{12}N_2O_2{\cdot}HBF_4$ M=388.13 g.mol^{-1}, triclinic system, space group *P-1*, a=9.45190(2), b=10.18780(2), c= 14.27590(2) Å, α=105.939(1), β=95.362(2), γ=97.071(1) °, Z=2, V=854.10(2) Å^3, Dc=1.51 g.cm^{-3}, μ(Cu Kα)=1.10 mm^{-1}, T=120 K, crystal dimensions of 0.03 x 0.09 x 0.23 mm, yellow prism. The structure converged to the final R=0.0316 and R_w=0.0825 using 3036 independent reflections for 305 refined parameters (θ_{max}=70.22°). CCDC registration number 2017716.

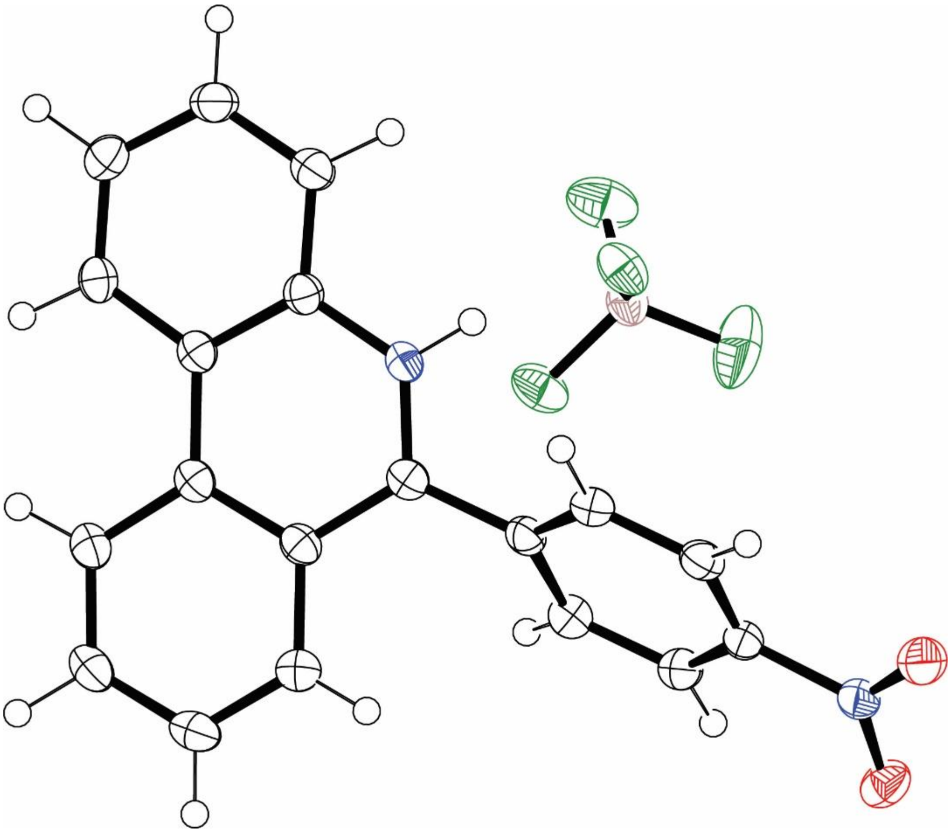

Figure S3. ORTEP projection of the crystal structure of **2g**.

X-ray data of **6-(4-nitrophenyl)-5-aza[5]helicene 3j**: $C_{27}H_{16}N_2O_2$, M=400.44 g.mol^{-1}, triclinic system, space group *P-1*, a=9.4107(5), b=9.7529(5), c=11.6942(6) Å, α=78.031(2), β=88.989(2), γ=67.123(2) °, Z=2, V=965.03(9) Å^3, Dc=1.38 g.cm^{-3}, μ(Cu Kα)=0.70 mm^{-1}, T=120 K, crystal dimensions of 0.10 x 0.29 x 0.33 mm, colorless prism. The structure converged to the final R=0.0361 and R_w=0.0926 using 3329 independent reflections for 344 refined parameters (θ_{max}=70.12°). CCDC registration number 2017714.

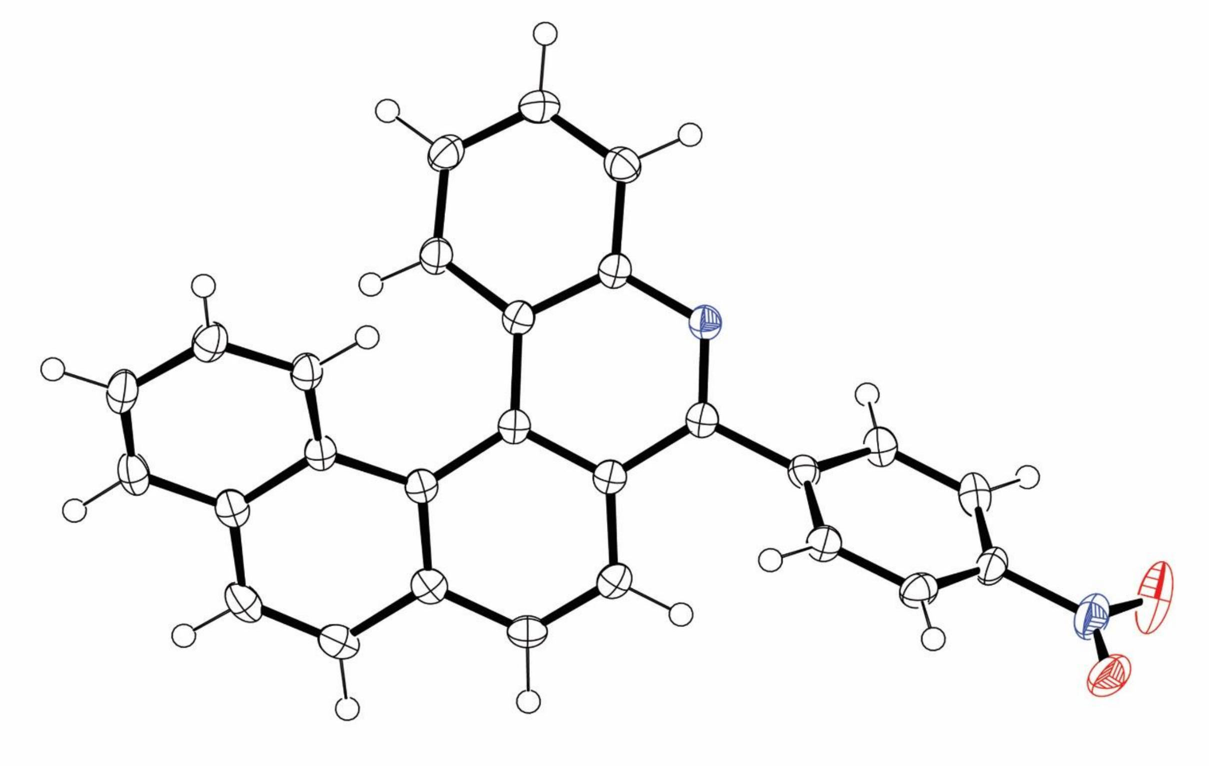

Figure S4. ORTEP projection of the crystal structure of **3j.**

X-ray data of **5-(4-nitrophenyl)-6-aza-chrysene 3l**: $C_{23}H_{14}N_2O_2$, M=350.38 g.mol^{-1}, monoclinic system, space group *P2₁/c*, a=20.4555(12), b=5.5335(3), c=14.4895(8) Å, β=91.154(2) °, Z=4, V= 1639.74(16) Å^3, Dc=1.42 g.cm^{-3}, μ(Cu Kα)=0.74 mm^{-1}, T=120 K, crystal dimensions of 0.05 x 0.13 x 0.38 mm, colorless bar. The structure converged to the final R=0.0348 and R_w=0.0898 using 2733 independent reflections for 300 refined parameters (θ_{max}=70.10°). CCDC registration number 2017715.

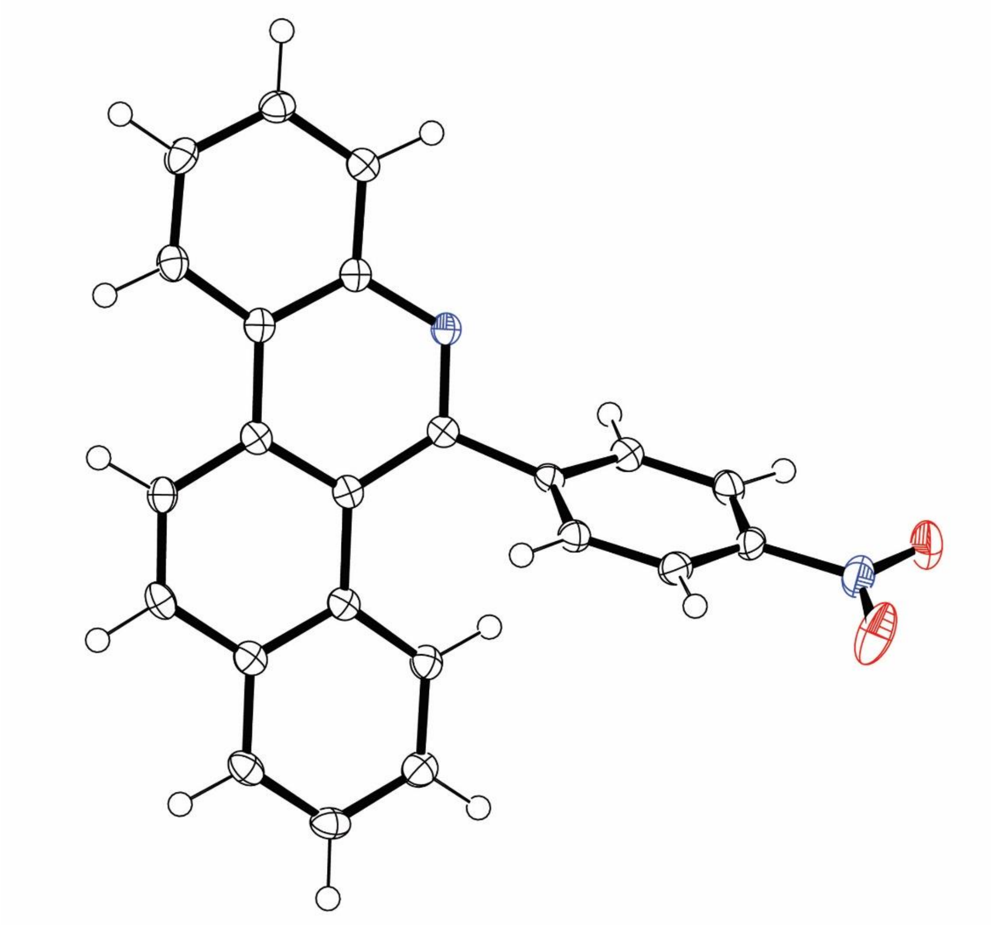

Figure S5. ORTEP projection of the crystal structure of **3l.**

X-ray data of **6-phenyl-5-aza-benzo[*g*]chrysene 3r**: $C_{27}H_{17}N$, M=355.44 g.mol^{-1}, monoclinic system, space group *P2₁*, a=11.4190(6), b=7.5358(4), c=11.7151(6) Å, β=118.720(1) °, Z=2, V=884.08(8) Å^3, Dc=1.34 g.cm^{-3}, μ(Cu Kα)=0.59 mm^{-1}, T=120 K, crystal dimensions of 0.10 x 0.25 x 0.32 mm, colorless prism. The structure converged to the final R=0.0271 and R_w=0.0705 using 3238 independent reflections for 322 refined parameters (θ_{max}=70.13°). CCDC registration number 2017717.

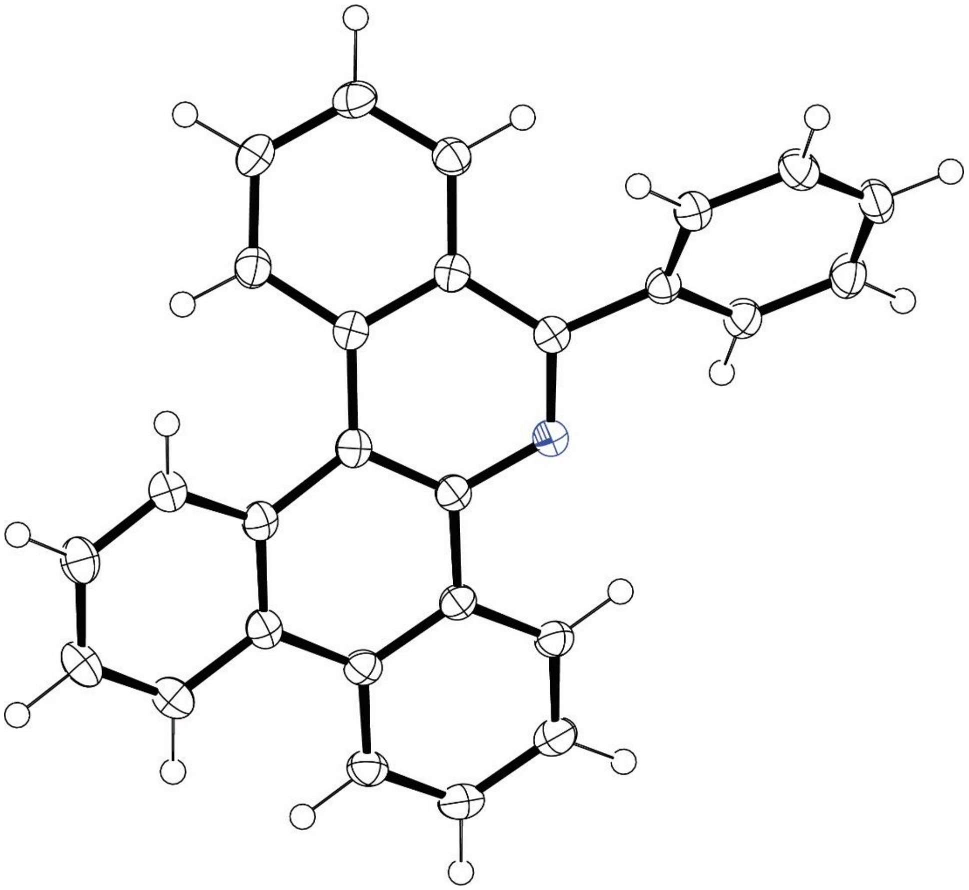

Figure S6. ORTEP projection of the crystal structure of **3r.**

3. Variable temperature NMR experiments

Variable temperature ^1H NMR experiment performed with -OMe (**1a**) and $-NO_2$ (**1g**) derivatives. ^1H NMR spectra were analysed using gNMR program. Activation parameters were obtained from modified Eyring equation: $\ln(k/T) = -\Delta H^{\ddagger}/RT + (23.76 + \Delta S^{\ddagger}/R)$ using least-squares fit to linear plot $\ln(k/T)$ versus 1/T.

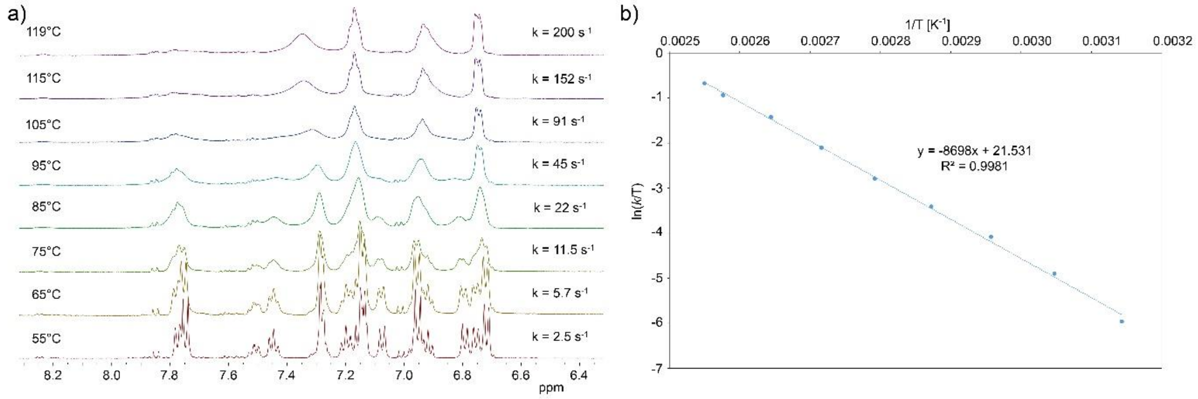

Figure S7. Variable temperature ^1H NMR experiment performed with -OMe (**1a**)

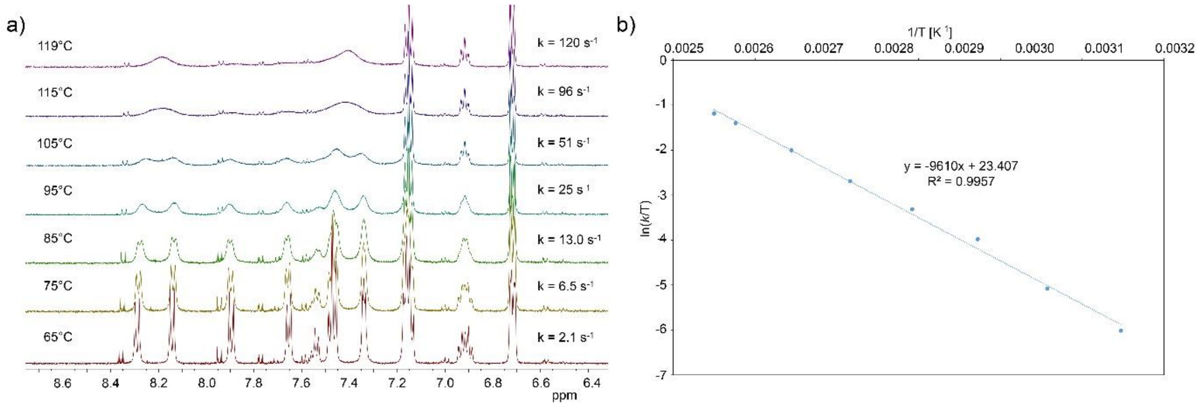

Figure S8. Variable temperature ^1H NMR experiment performed with $-NO_2$ (**1g**)

4. NMR Kinetics measurements

An NMR tube was charged with 7.5 µmol of starting imine, 15 µmol of TEMPO, and 2 mL of $CDCl_3$. 30 µmol of $HBF_4.Et_2O$ was added via syringe. The resulting mixture was homogenized by vigorous shaking. An NMR tube was irradiated and checked off-line at regular intervals. A course of the reaction was subsequently monitored and by ^1H or ^{19}F NMR. The obtained Spectra were visualized and compared in the program MestReNova (Mestrelab).

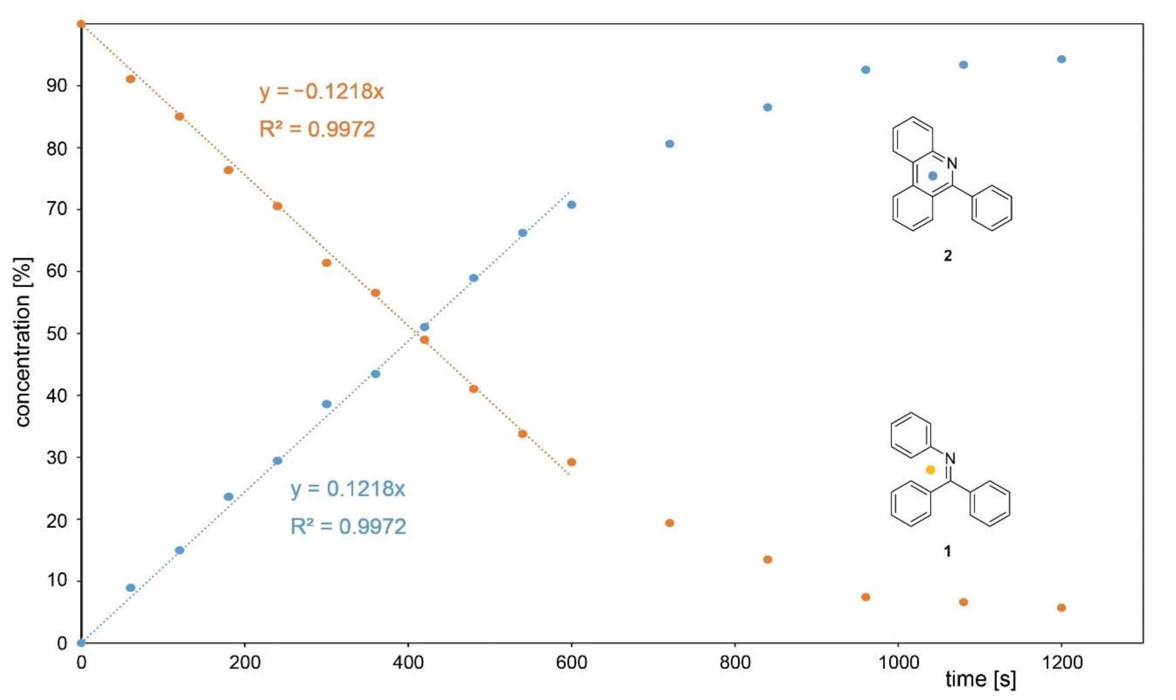

Figure S9. Monitoring of reaction kinetics of **1**.

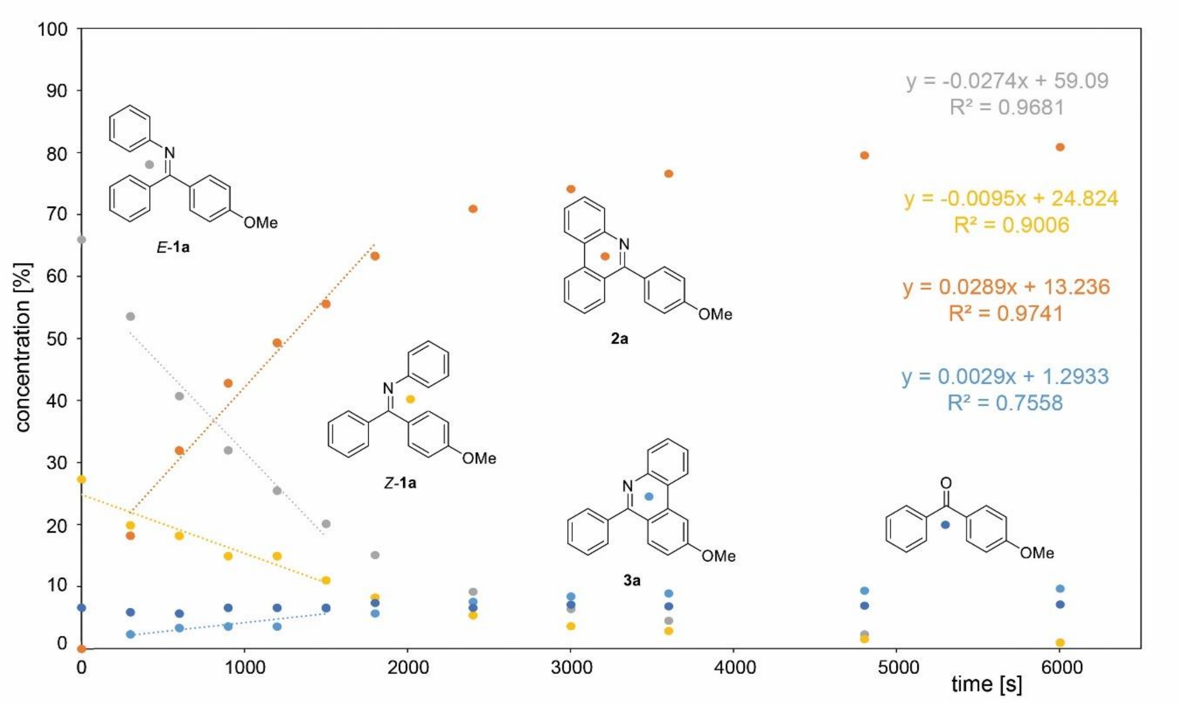

Figure S10. Monitoring of reaction kinetics of **1a.**

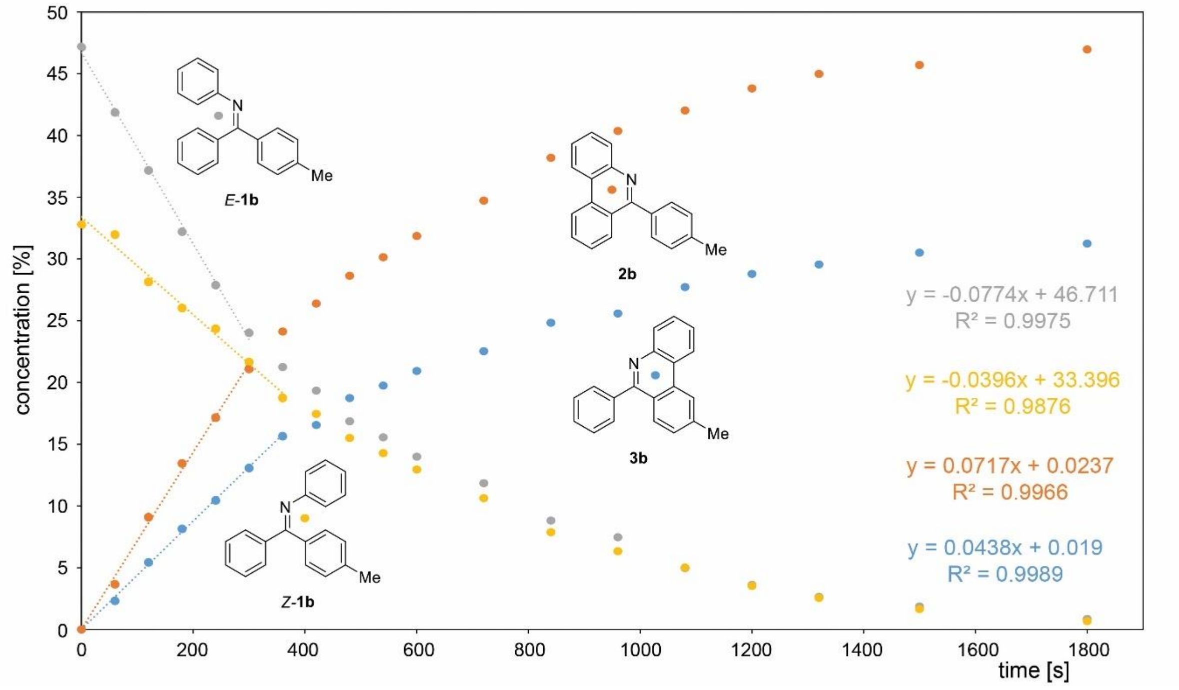

Figure S11. Monitoring of reaction kinetics of **1b.**

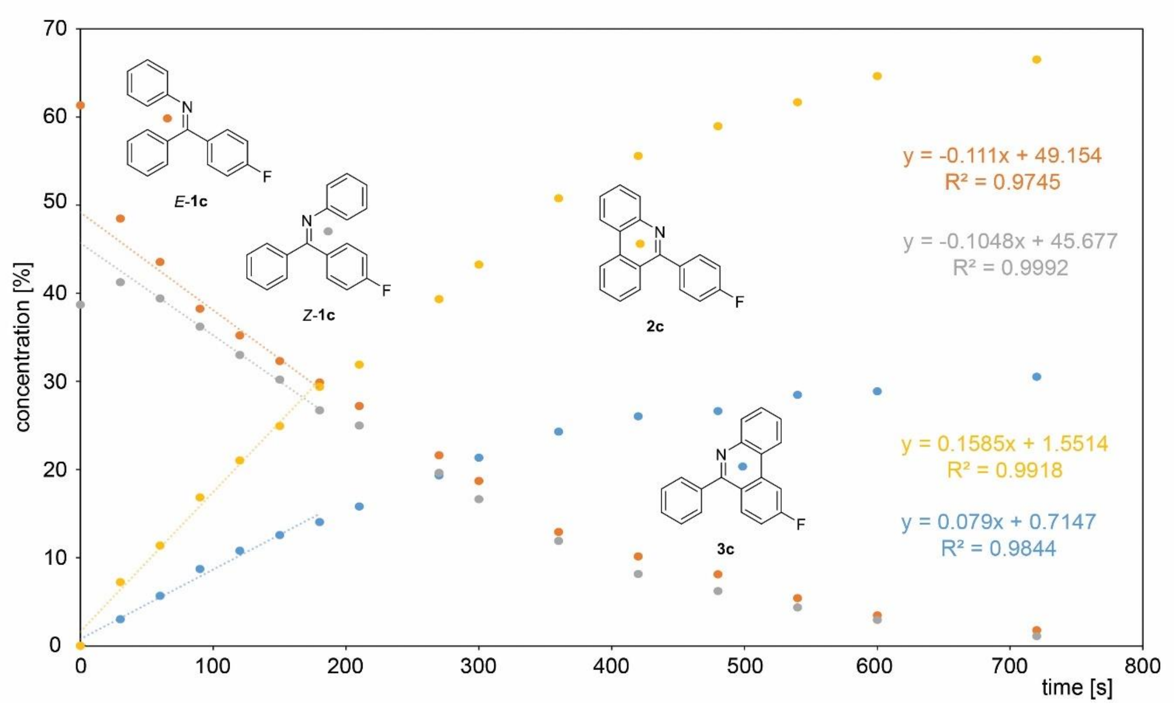

Figure S12. Monitoring of reaction kinetics of **1c.**

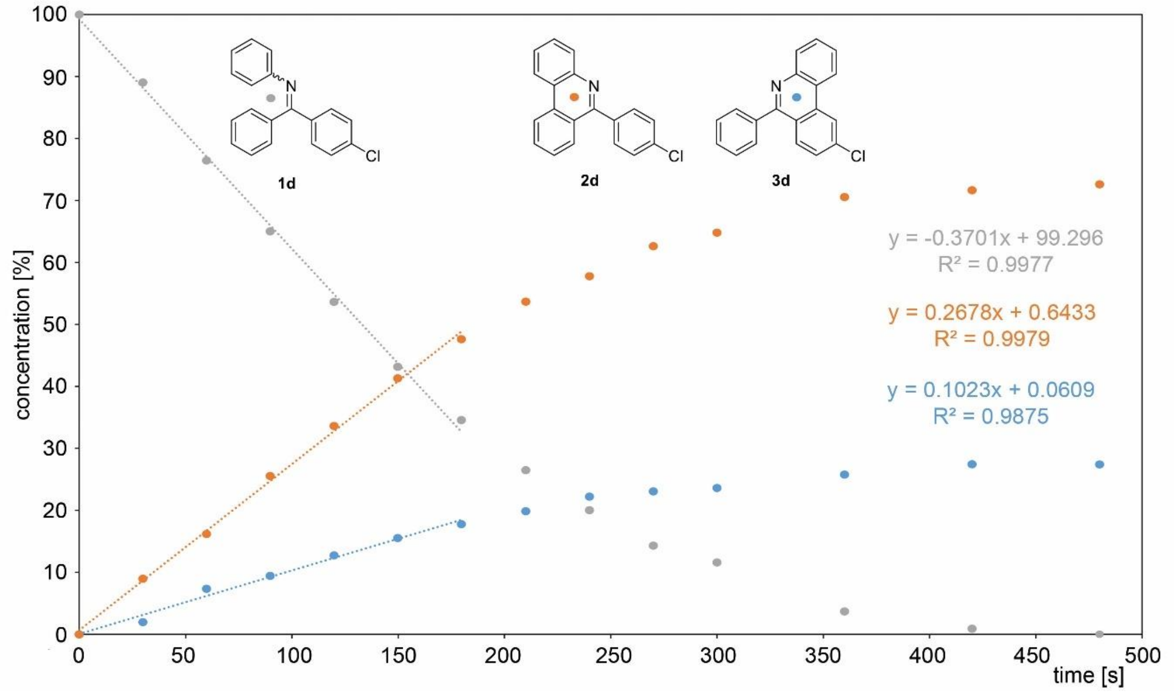

Figure S13. Monitoring of reaction kinetics of **1d.**

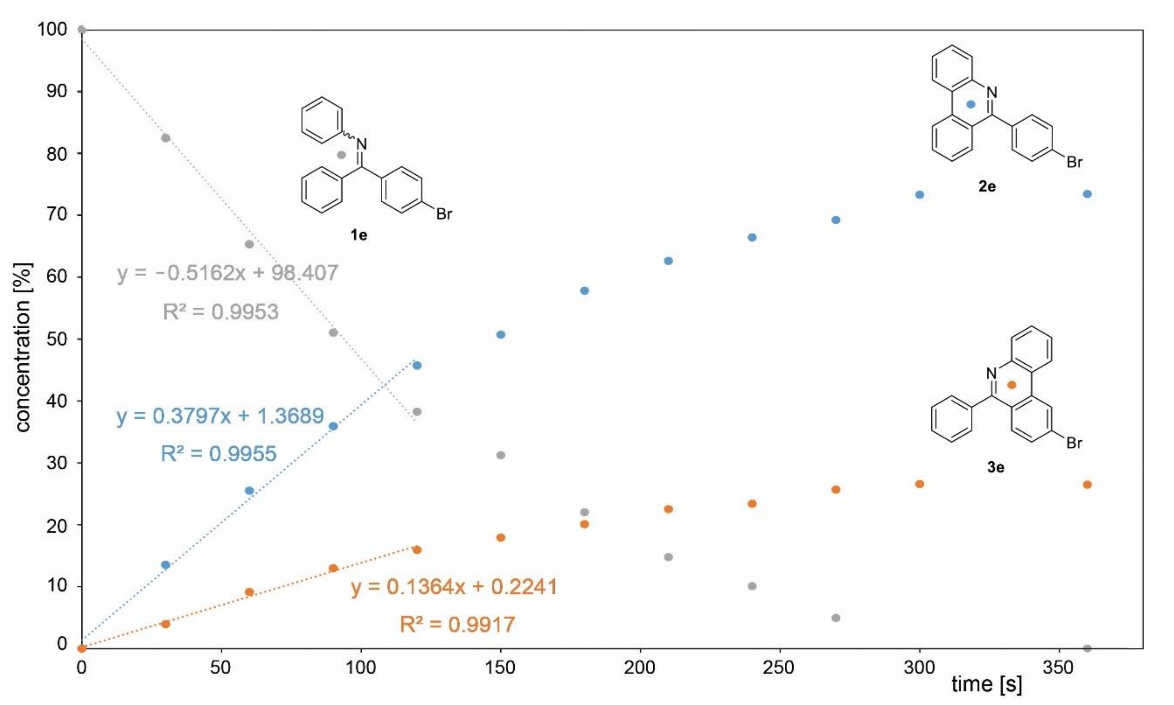

Figure S14. Monitoring of reaction kinetics of **1e.**

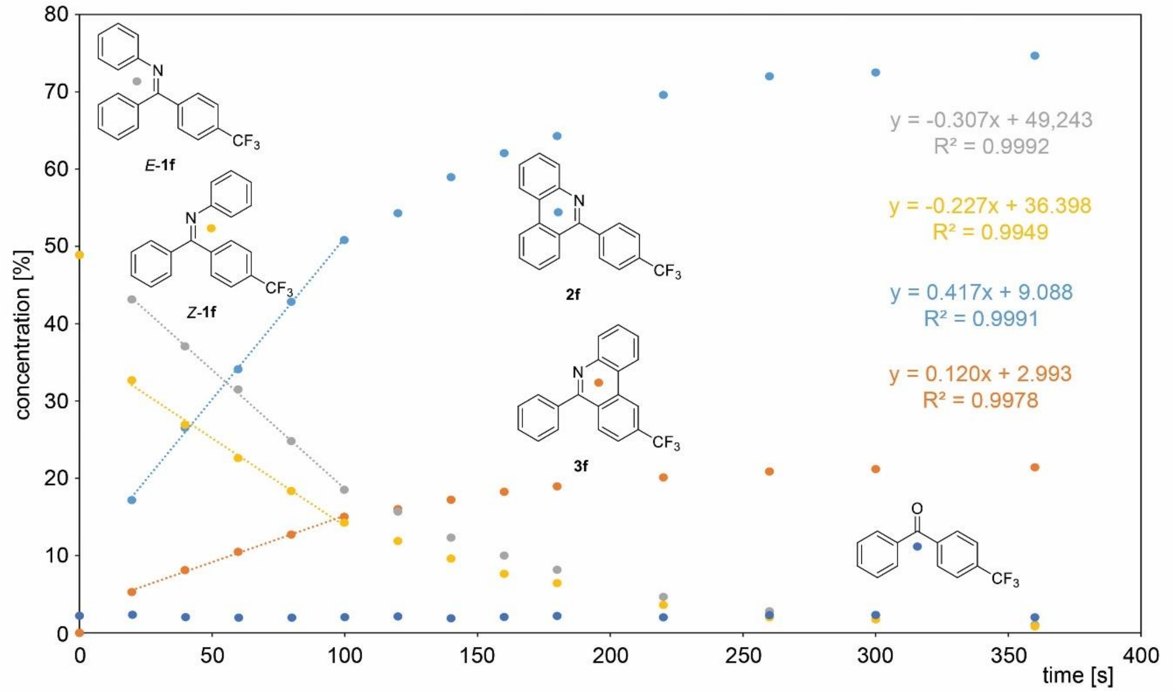

Figure S15. Monitoring of reaction kinetics of **1f.**

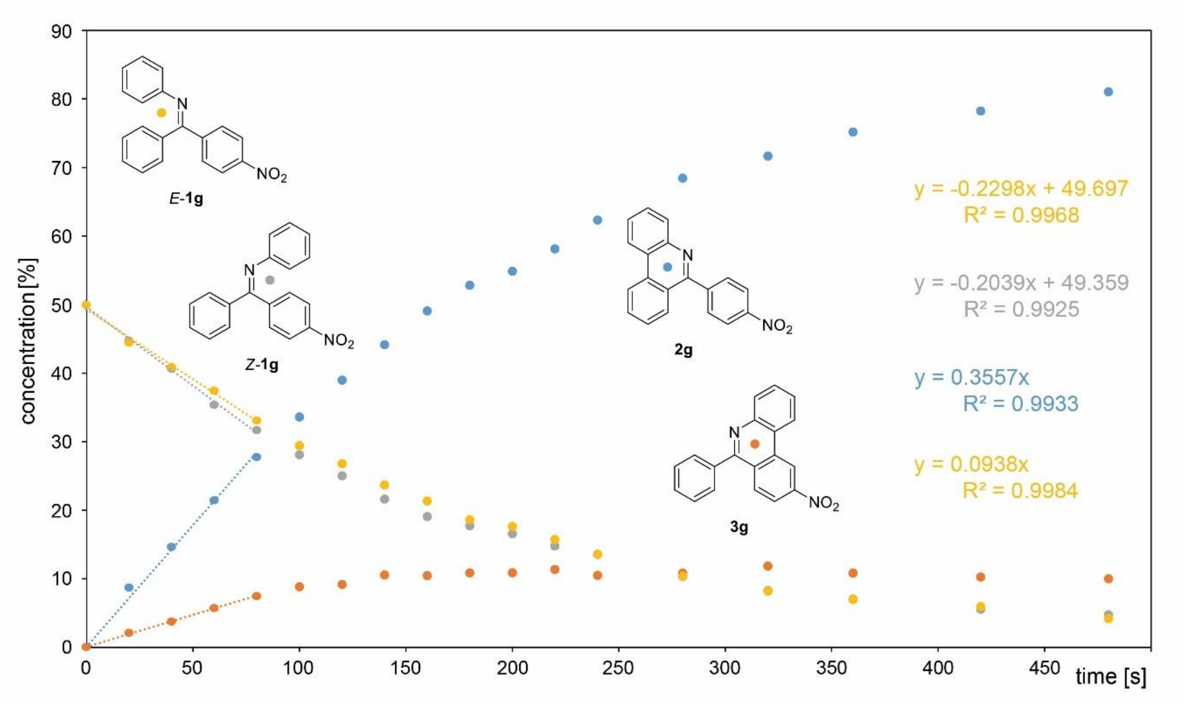

Figure S16. Monitoring of reaction kinetics of **1g.**

5. DFT Calculations

All DFT calculations were performed at Gaussian 09 software using the B3LYP DFT functional and the 6-31G(d) basis set. Frequency calculations were conducted for all structures, confirming that they correspond to a minimum. The Gibbs Free energy values are reported at 298 K. Time-dependent DFT calculations were performed the B3LYP/6-31G(d) level of theory. Molecules and orbitals were rendered with GaussView.

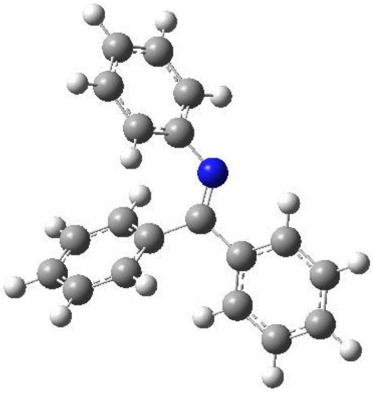

1

Charge = 0 Multiplicity = 1

Symbol	X	Y	Z
C	2.34435	-1.37389	-0.96389
C	1.13615	-2.15009	-0.89761
C	3.58527	-1.99023	-1.09478
C	1.28894	-3.55305	-0.72968
C	3.68504	-3.39723	-1.09724
H	4.66181	-3.85976	-1.23524
C	2.56198	-4.1673	-0.88496
H	2.63669	-5.25116	-0.82012
N	2.34202	0.05399	-0.95206
C	3.32622	0.7992	-1.33367
C	4.90605	0.36266	-3.22388
C	6.13567	-0.09131	-3.7093
C	7.10324	-0.57881	-2.82436
C	6.84243	-0.6129	-1.4505
C	5.61454	-0.16201	-0.95832

C	4.64638	0.32305	-1.84737
H	4.15288	0.74521	-3.91126
H	6.3393	-0.06537	-4.77853
H	8.05956	-0.93373	-3.20528
H	7.59366	-0.99655	-0.76059
H	5.40903	-0.19443	0.11279
C	2.81402	5.05789	-1.18431
C	4.05516	4.48893	-0.87859
C	4.22388	3.10335	-0.9279
C	3.14384	2.27882	-1.28195
C	1.89737	2.85045	-1.58185
C	1.73717	4.23804	-1.53518
H	2.68697	6.13854	-1.14783
H	4.8928	5.12692	-0.60181
H	5.19307	2.66386	-0.6901
H	0.77008	4.67943	-1.77014
H	4.48974	-1.38927	-1.17877
H	1.07073	2.22448	-1.84593
H	0.4377	-4.15299	-0.48399
H	0.17071	-1.69461	-0.97098

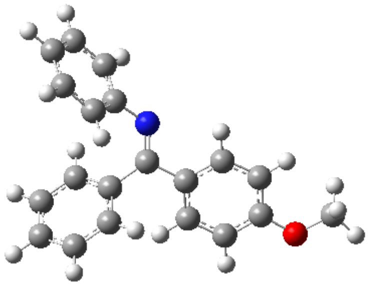

E-**1a**

Charge = 0 Multiplicity = 1

Symbol	X	Y	Z
C	2.34435	-1.37389	-0.96389
C	1.13615	-2.15009	-0.89761
C	3.58527	-1.99023	-1.09478
C	1.28894	-3.55305	-0.72968
C	3.68504	-3.39723	-1.09724

H	4.66181	-3.85976	-1.23524
C	2.56198	-4.1673	-0.88496
H	2.63669	-5.25116	-0.82012
N	2.34202	0.05399	-0.95206
C	3.32622	0.7992	-1.33367
C	4.90605	0.36266	-3.22388
C	6.13567	-0.09131	-3.7093
C	7.10324	-0.57881	-2.82436
C	6.84243	-0.6129	-1.4505
C	5.61454	-0.16201	-0.95832
C	4.64638	0.32305	-1.84737
H	4.15288	0.74521	-3.91126
H	6.3393	-0.06537	-4.77853
H	8.05956	-0.93373	-3.20528
H	7.59366	-0.99655	-0.76059
H	5.40903	-0.19443	0.11279
C	2.81402	5.05789	-1.18431
C	4.05516	4.48893	-0.87859
C	4.22388	3.10335	-0.9279
C	3.14384	2.27882	-1.28195
C	1.89737	2.85045	-1.58185
C	1.73717	4.23804	-1.53518
H	4.8928	5.12692	-0.60181
H	5.19307	2.66386	-0.6901
H	0.77008	4.67943	-1.77014
H	4.48974	-1.38927	-1.17877
H	1.07073	2.22448	-1.84593
H	0.4377	-4.15299	-0.48399
H	0.17071	-1.69461	-0.97098
O	2.64714	6.47731	-1.1364
C	2.02273	6.84317	0.09696
H	2.01226	7.90916	0.18898
H	1.01853	6.47404	0.11159
H	2.57019	6.42008	0.91316

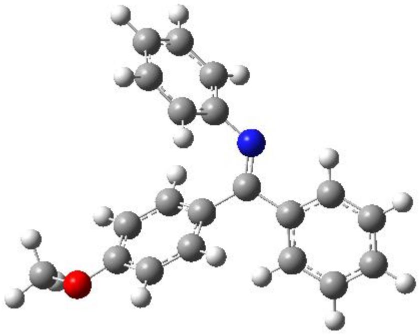

Z-**1a**

Charge = 0 Multiplicity = 1

Symbol	X	Y	Z
C	1.01986	0.52024	-0.68095
C	0.07293	-0.24194	0.08654
C	0.7	1.7878	- 1.15829
C	-1.25989	0.25009	0.12357
C	-0.58939	2.32169	-0.95327
H	-0.80864	3.32932	-1.30495
C	-1.56435	1.55497	-0.3525
H	-2.57996	1.93179	-0.24722
N	2.34202	0.05399	-0.95206
C	3.32622	0.7992	-1.33367
C	5.13418	0.19883	-2.95544
C	6.43255	-0.23886	-3.23177
C	7.31357	-0.52896	-2.18457
C	6.89706	-0.38133	-0.8574
C	5.59978	0.0544	-0.57378
C	4.71877	0.34146	-1.62471
H	4.44806	0.4279	-3.76962
H	6.75737	-0.3543	- 4.26451
H	8.32383	-0.87169	-2.40254
H	7.58102	-0.61124	-0.04078
H	5.27319	0.16359	0.46159
C	2.57338	4.987	-1.89641
C	2.29441	4.07929	-2.92395
C	2.53766	2.71603	-2.74204

C	3.06692	2.2556	-1.52567
C	3.35269	3.16813	-0.49803
C	3.10219	4.53026	-0.68536
H	1.88607	4.4352	-3.86834
H	2.31777	2.01164	-3.54485
H	3.32266	5.23603	0.11373
H	1.43869	2.36882	-1.70876
H	3.76189	2.82121	0.42777
H	-2.0419	-0.36741	0.5135
H	0.35717	-1.13448	0.60372
O	2.31889	6.38137	-2.08582
C	3.53951	7.04872	-2.4169
H	3.33039	8.06415	-2.68162
H	4.19735	7.0283	-1.57326
H	4.00413	6.55306	-3.24355

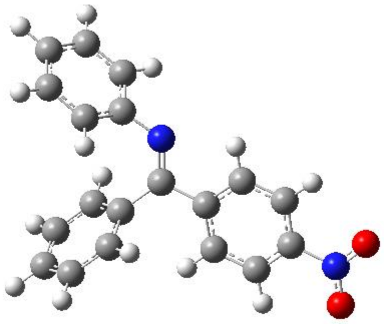

E-**1g**

Charge = 0 Multiplicity = 1

Symbol	X	Y	Z
C	2.34435	-1.37389	-0.96389
C	1.13615	-2.15009	-0.89761
C	3.58527	-1.99023	-1.09478
C	1.28894	-3.55305	-0.72968
C	3.68504	-3.39723	-1.09724
H	4.66181	-3.85976	-1.23524
C	2.56198	-4.1673	-0.88496
H	2.63669	-5.25116	-0.82012

N	2.34202	0.05399	-0.95206
C	3.32622	0.7992	-1.33367
C	4.90605	0.36266	-3.22388
C	6.13567	-0.09131	-3.7093
C	7.10324	-0.57881	-2.82436
C	6.84243	-0.6129	-1.4505
C	5.61454	-0.16201	-0.95832
C	4.64638	0.32305	-1.84737
H	4.15288	0.74521	-3.91126
H	6.3393	-0.06537	-4.77853
H	8.05956	-0.93373	-3.20528
H	7.59366	-0.99655	-0.76059
H	5.40903	-0.19443	0.11279
C	2.81402	5.05789	-1.18431
C	4.05516	4.48893	-0.87859
C	4.22388	3.10335	-0.9279
C	3.14384	2.27882	-1.28195
C	1.89737	2.85045	-1.58185
C	1.73717	4.23804	-1.53518
H	4.8928	5.12692	-0.60181
H	5.19307	2.66386	-0.6901
H	0.77008	4.67943	-1.77014
H	4.48974	-1.38927	-1.17877
H	1.07073	2.22448	-1.84593
H	0.4377	-4.15299	-0.48399
H	0.17071	-1.69461	-0.97098
N	2.64248	6.51702	-1.13506
O	1.84772	6.84472	-0.08122
O	2.06411	6.94469	-2.28926

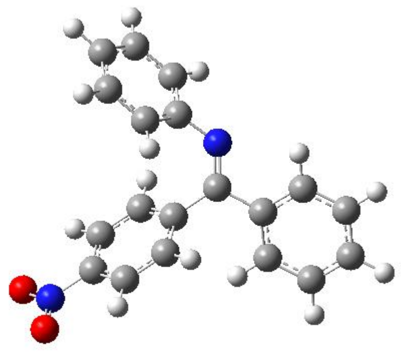

Z-**1g**

Charge = 0 Multiplicity = 1

Symbol	X	Y	Z
C	1.15674	0.52821	-0.31237
C	0.27566	-0.33711	0.42352
C	0.91477	1.8964	-0.39231
C	-0.95862	0.22401	0.84962
C	-0.24201	2.45064	0.19444
H	-0.39522	3.52843	0.15011
C	-1.18075	1.62665	0.77681
H	-2.10729	2.03587	1.17505
N	2.34202	0.05399	-0.95206
C	3.32622	0.7992	-1.33367
C	4.90605	0.36266	-3.22388
C	6.13567	-0.09131	-3.7093
C	7.10324	-0.57881	-2.82436
C	6.84243	-0.6129	-1.4505
C	5.61454	-0.16201	-0.95832
C	4.64638	0.32305	-1.84737
H	4.15288	0.74521	-3.91126
H	6.3393	-0.06537	-4.77853
H	8.05956	-0.93373	-3.20528
H	7.59366	-0.99655	-0.76059
H	5.40903	-0.19443	0.11279
C	2.79009	5.05515	-1.19012
C	1.92918	4.26467	-1.95928
C	2.1015	2.87944	-2.00612

C	3.14384	2.27882	-1.28195
C	4.01101	3.07371	-0.51606
C	3.8296	4.4587	-0.47001
H	1.12194	4.7293	-2.52297
H	1.42769	2.26672	-2.60578
H	4.50208	5.07312	0.12626
H	1.60903	2.54577	-0.9239
H	4.81008	2.61973	0.03193
H	-1.72828	-0.41525	1.22892
H	0.53594	-1.35225	0.63951
N	2.60215	6.51224	-1.14067
O	3.62073	7.12344	-1.8029
O	2.5922	6.92433	0.15535

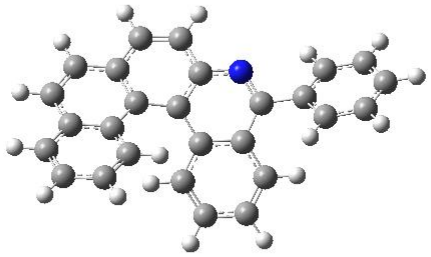

3o

Charge = 0 Multiplicity = 1

Symbol	X	Y	Z
C	-2.07256	-1.97917	0.84216
C	-1.44113	-0.76265	0.47186
C	-0.03682	-0.6234	0.6088
C	0.68497	-1.67843	1.23575
C	0.05181	-2.8462	1.60669
C	-1.33716	-3.0113	1.38602
C	0.58626	0.63081	0.21664
C	2.55843	-1.3954	-0.95705
C	2.95574	-0.17768	-0.33346
C	2.00171	0.85289	0.0512
C	4.34278	0.07311	-0.18704

C	5.28548	-0.94443	-0.50705
C	4.86415	-2.1437	-1.03941
C	3.487	-2.35716	-1.29481
H	-3.1512	-2.07996	0.70634
H	-1.81521	-3.94775	1.66792
H	6.34431	-0.75309	-0.33953
H	5.57932	-2.92644	-1.28646
C	-2.20822	0.40234	0.03942
C	-0.27809	1.73987	0.07179
C	0.2362	3.07904	0.07819
C	1.5797	3.27834	0.22283
C	2.48644	2.1676	0.21003
H	-0.47228	3.90505	-0.00537
H	1.98939	4.28454	0.3127
C	4.79414	1.38451	0.21267
H	5.86175	1.54018	0.36106
C	3.9039	2.403	0.34384
H	4.23421	3.41683	0.56924
H	0.6127	-3.64952	2.08448
H	3.17315	-3.28508	-1.7703
H	1.50423	-1.55308	-1.19311
H	1.74812	-1.54425	1.44723
C	-6.39578	0.05461	-0.78563
C	-5.95717	0.86955	0.26253
C	-4.59121	0.98992	0.53481
C	-3.66329	0.28261	-0.24267
C	-4.10069	-0.5303	-1.29995
C	-5.46708	-0.6411	-1.56779
H	-7.45993	-0.03506	-0.9968
H	-6.67936	1.41732	0.86598
H	-4.24351	1.63788	1.33965
H	-3.37574	-1.06434	-1.91301
H	-5.80912	-1.26761	-2.38994
N	-1.6654	1.60009	-0.07749

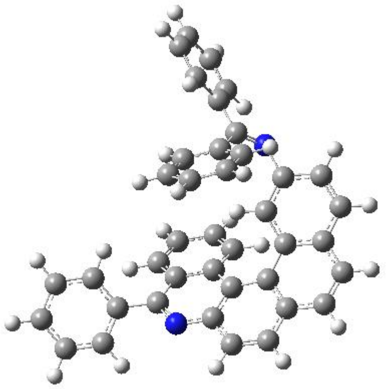

10b

Charge = 0 Multiplicity = 1

Symbol	X	Y	Z
C	3.35813	1.92025	1.60976
C	3.26216	0.74655	0.82136
C	2.2846	-0.2438	1.14836
C	1.48464	-0.02703	2.29933
C	1.59898	1.12547	3.05003
C	2.533	2.11756	2.69774
C	2.27231	-1.48957	0.3965
C	-0.7728	-1.00879	0.59485
C	-0.16847	-2.28448	0.68653
C	1.25268	-2.51662	0.44123
C	-1.02598	-3.40524	0.92752
C	-2.39901	-3.18195	1.19513
C	-2.952	-1.92418	1.1337
C	-2.13723	-0.81625	0.78281
H	4.12348	2.65047	1.37387
H	2.62823	3.01763	3.29866
H	-3.02842	-4.03981	1.42063
H	-4.00795	-1.77185	1.33132
C	4.21855	0.44202	- 0.22536
C	3.46339	-1.75463	-0.32934
C	3.77843	-3.08166	-0.7359
C	2.93411	-4.108	-0.42501
C	1.65157	-3.85049	0.14217
H	4.7196	-3.2375	-1.25334

H	3.19746	-5.13575	-0.66248
C	-0.51298	-4.73255	0.82007
H	-1.17814	-5.56851	1.02262
C	0.75954	-4.94304	0.38144
H	1.12666	-5.95148	0.20745
H	0.97831	1.25265	3.93286
H	-0.17772	-0.13973	0.34744
H	0.79033	-0.79587	2.61211
C	6.87651	3.32063	-2.00202
C	7.32365	2.03286	-1.69444
C	6.45897	1.11102	-1.11016
C	5.13387	1.46385	-0.80619
C	4.69194	2.75741	-1.12844
C	5.55628	3.67674	-1.72388
H	7.55114	4.03873	-2.46068
H	8.34913	1.74622	-1.91274
H	6.79288	0.10398	-0.88336
H	3.66004	3.03646	-0.9368
H	5.19365	4.66969	-1.97641
N	4.36184	-0.77999	-0.67793
N	-2.6353	0.49568	0.72867
C	-2.58747	1.25788	-0.3083
C	-1.50995	1.9481	-2.48692
C	-0.84345	1.64015	-3.67296
C	-0.4856	0.31993	-3.95284
C	-0.80396	-0.69182	-3.04449
C	-1.48645	-0.38799	-1.86753
C	-1.84825	0.93572	-1.57521
H	-1.77825	2.97791	-2.26776
H	-0.59958	2.43261	-4.37552
H	0.04075	0.081	-4.87303
H	-0.5237	-1.72064	-3.25321
H	-1.74164	-1.17834	-1.16846
C	-4.56082	5.07377	-0.02773
C	-4.81961	4.23961	-1.11517
C	-4.19526	2.99527	-1.20953

C	-3.29545	2.56951	-0.21885
C	-3.03677	3.42354	0.86819
C	-3.66554	4.66038	0.96408
H	-5.05182	6.0404	0.04826
H	-5.50664	4.55657	-1.89522
H	-4.39801	2.35886	-2.06443
H	-2.33404	3.09471	1.62609
H	-3.46011	5.30525	1.81449

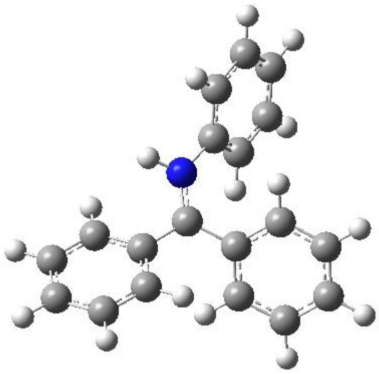

1+H

Charge = 1 Multiplicity = 1

Symbol	X	Y	Z
C	-1.65715	-1.26699	-0.02255
C	-2.41944	-1.9803	0.91854
C	-2.31832	-0.62562	-1.0849
C	-3.809	-2.00899	0.82659
C	-3.70751	-0.68186	-1.18419
H	-4.20284	-0.18699	-2.01588
C	-4.4617	-1.36252	-0.2263
H	-5.54465	-1.3992	-0.30567
N	-0.25684	-1.33631	0.07347
C	0.53331	-0.32069	0.04956
C	-0.77764	1.57511	1.07363
C	-1.18987	2.9066	1.09576
C	-0.75335	3.79373	0.10949
C	0.10686	3.3447	-0.89413
C	0.53446	2.01693	-0.90673

C	0.08934	1.1144	0.07164
H	-1.12741	0.88571	1.83583
H	-1.85557	3.25061	1.88269
H	-1.08045	4.82993	0.12439
H	0.45003	4.02885	-1.66565
H	1.21208	1.67333	-1.68347
C	4.74756	-1.20218	0.01437
C	4.30695	0.05411	0.43051
C	2.94429	0.35452	0.43343
C	2.00007	-0.60216	0.02695
C	2.45657	-1.86755	-0.38287
C	3.81581	-2.16258	-0.3924
H	5.80948	-1.43357	0.00614
H	5.02323	0.80373	0.75616
H	2.61269	1.33256	0.76598
H	4.15243	-3.14277	-0.71978
H	-1.73633	-0.09825	-1.83422
H	1.7234	-2.60605	-0.68886
H	-4.38383	-2.55253	1.5722
H	-1.90184	-2.5033	1.71752
H	0.15637	-2.24209	0.16736

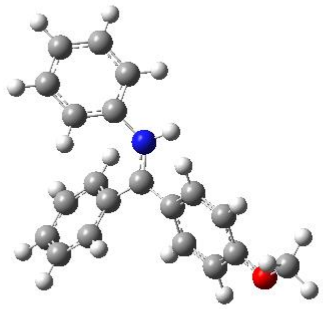

E-**1a+H**

Charge = 1 Multiplicity = 1

Symbol	X	Y	Z
C	-2.10106	-1.45248	-0.01879
C	-2.73202	-2.28936	0.91841
C	-2.85696	-0.94956	-1.09278

C	-4.09111	-2.57437	0.81102
C	-4.21109	-1.26009	-1.20669
H	-4.77886	-0.86797	-2.04691
C	-4.83914	-2.06387	-0.2531
H	-5.89591	-2.29936	-0.34413
N	-0.71428	-1.26242	0.09609
C	-0.12228	-0.11814	0.06818
C	-1.77057	1.50794	1.06751
C	-2.42671	2.73785	1.0752
C	-2.1606	3.68223	0.08151
C	-1.22599	3.3942	-0.91476
C	-0.55581	2.17057	-0.91281
C	-0.82748	1.20933	0.07295
H	-1.98846	0.77135	1.83467
H	-3.14947	2.95729	1.85642
H	-2.67737	4.63822	0.08484
H	-1.01385	4.12371	-1.69191
H	0.17824	1.95295	-1.68378
C	4.18682	-0.20317	0.08382
C	3.50976	0.9699	0.44716
C	2.12264	1.00881	0.43208
C	1.36589	-0.11915	0.06113
C	2.06068	-1.28672	-0.29186
C	3.45101	-1.33852	-0.28784
H	4.09384	1.83633	0.74113
H	1.6196	1.92401	0.72592
H	3.9499	-2.25709	-0.57537
H	-2.37285	-0.3278	-1.83934
H	1.48525	-2.16318	-0.57024
H	-4.56506	-3.21099	1.55404
H	-2.13705	-2.70382	1.7272
O	5.5466	-0.13945	0.12233
C	6.28783	-1.3004	-0.22316
H	7.33874	-1.02573	-0.11677
H	6.06141	-2.13791	0.44931
H	6.09615	-1.60749	-1.25953

H	-0.14357	-2.07592	0.20792

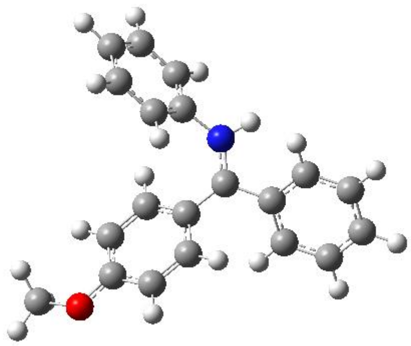

Z-**1a+H**

Charge = 1 Multiplicity = 1

Symbol	X	Y	Z
C	0.28866	2.34846	0.01191
C	0.61125	3.30226	0.99279
C	1.12999	2.21696	-1.10737
C	1.76844	4.06959	0.87972
C	2.27235	3.00677	-1.22498
H	2.90758	2.89767	-2.1006
C	2.60511	3.92905	-0.23043
H	3.49891	4.53949	-0.32517
N	-0.93011	1.66051	0.13236
C	-1.0664	0.38223	0.05178
C	-2.76063	-1.45784	0.45745
C	-4.07937	-1.91328	0.48531
C	-5.12179	-1.06723	0.10679
C	-4.83939	0.24282	-0.29265
C	-3.52588	0.70083	-0.31316
C	-2.46543	-0.14453	0.05782
H	-1.95796	-2.12151	0.76159
H	-4.29031	-2.93011	0.80574
H	-6.14826	-1.42428	0.12224
H	-5.64677	0.90677	-0.59038
H	-3.29437	1.71746	-0.61238
C	2.18839	-2.44804	-0.15292

C	1.1324	-2.51606	-1.07467
C	0.08076	-1.61414	-0.99693
C	0.05886	-0.6045	-0.01677
C	1.12089	-0.54914	0.89377
C	2.17511	-1.46107	0.84093
H	1.15887	-3.28696	-1.83843
H	-0.73348	-1.68378	-1.71262
H	2.97396	-1.39109	1.57006
H	0.87394	1.50295	-1.88394
H	1.12689	0.21825	1.66135
H	2.00926	4.79243	1.65527
H	-0.05931	3.42505	1.83846
O	3.16793	-3.38236	-0.30667
C	4.26949	-3.35913	0.58889
H	4.92352	-4.17585	0.27855
H	4.81832	-2.41046	0.52728
H	3.94975	-3.52486	1.62603
H	-1.75399	2.20412	0.29274

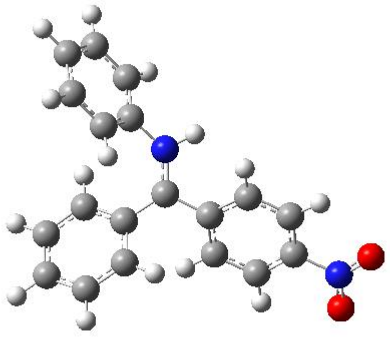

E-**1g+H**

Charge = 1 Multiplicity = 1

Symbol	X	Y	Z
C	-2.38699	-1.41824	-0.01581
C	-3.00126	-2.28514	0.9039
C	-3.16422	-0.84708	-1.03869
C	-4.37017	-2.53175	0.83417
C	-4.52774	-1.12405	-1.11908

H	-5.11469	-0.68383	-1.92104
C	-5.14044	-1.95555	-0.17917
H	-6.20489	-2.16294	-0.24335
N	-0.99142	-1.26513	0.06523
C	-0.36978	-0.13954	0.03098
C	-1.92781	1.55147	1.05885
C	-2.52834	2.80919	1.07905
C	-2.23701	3.74269	0.08206
C	-1.33329	3.41657	-0.93088
C	-0.71791	2.16466	-0.94271
C	-1.01622	1.21508	0.0469
H	-2.1642	0.82397	1.82924
H	-3.22668	3.05913	1.87294
H	-2.71027	4.72065	0.09593
H	-1.1025	4.13785	-1.71007
H	-0.00881	1.91764	-1.72829
C	3.90123	-0.40136	0.01788
C	3.30715	0.79421	0.41222
C	1.91797	0.88605	0.40513
C	1.125	-0.20676	0.01565
C	1.7598	-1.40256	-0.36902
C	3.14395	-1.5065	-0.37339
H	3.92962	1.62594	0.71784
H	1.44801	1.81203	0.71673
H	3.64518	-2.4182	-0.67425
H	-2.69196	-0.20328	-1.77367
H	1.14277	-2.24506	-0.65973
H	-4.83377	-3.19105	1.56331
H	-2.38765	-2.75108	1.66929
N	5.36892	-0.50141	0.01343
O	6.01107	0.4885	0.36507
O	5.86815	-1.56904	-0.34178
H	-0.44029	-2.09455	0.15643

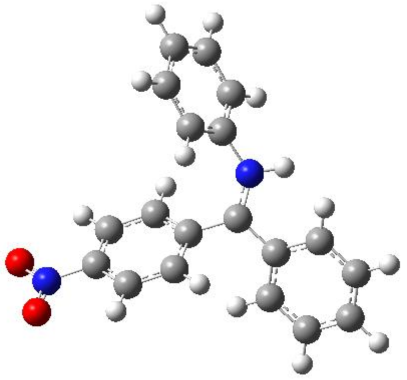

Z-**1g+H**

Charge = 1 Multiplicity = 1

Symbol	X	Y	Z
C	0.50615	2.46634	0.04615
C	0.49692	3.50974	-0.89534
C	-0.40436	2.50695	1.11635
C	-0.43732	4.53805	-0.79792
C	-1.31762	3.555	1.22128
H	-2.01022	3.57702	2.0587
C	-1.34853	4.56807	0.26123
H	-2.06599	5.37948	0.34404
N	1.51049	1.48832	-0.06392
C	1.30437	0.21914	-0.04456
C	2.41813	-2.0188	-0.4479
C	3.55772	-2.82356	-0.4653
C	4.79043	-2.3016	-0.07351
C	4.88105	-0.96517	0.32869
C	3.74796	-0.15885	0.33971
C	2.4978	-0.67562	-0.04511
H	1.46731	-2.43561	-0.76375
H	3.48003	-3.85829	-0.78759
H	5.67653	-2.93064	-0.08071
H	5.83837	-0.55342	0.63669
H	3.80619	0.88077	0.6433
C	-2.57575	-1.59582	-0.05505
C	-1.67675	-1.9337	0.95423
C	-0.41369	-1.34926	0.94497

C	-0.05741	-0.42165	-0.04771
C	-0.98826	-0.10194	-1.04885
C	-2.24873	-0.69005	-1.06239
H	-1.97123	-2.64228	1.71845
H	0.30279	-1.61024	1.71786
H	-2.97572	-0.4587	-1.83096
H	-0.37781	1.72674	1.87078
H	-0.72213	0.61375	-1.81942
H	-0.44324	5.32829	-1.54412
H	1.22695	3.49232	-1.69929
N	-3.90954	-2.21834	-0.05936
O	-4.17184	-3.01258	0.84361
O	-4.68206	-1.90777	-0.96546
H	2.45437	1.80288	-0.16464

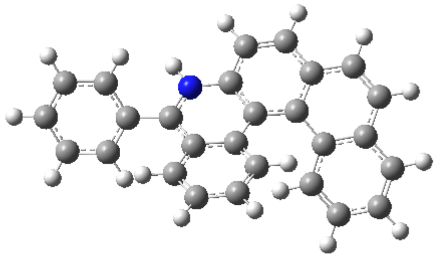

3o+H

Charge = 1 Multiplicity = 1

Symbol	X	Y	Z
C	2.07788	-2.03587	-0.98641
C	1.45543	-0.84743	-0.52941
C	0.03551	-0.71379	-0.62598
C	-0.6866	-1.76909	-1.24019
C	-0.05476	-2.91524	-1.67886
C	1.33822	-3.06116	-1.53895
C	-0.57448	0.55255	-0.24966
C	-2.64062	-1.37692	0.99777
C	-2.98043	-0.15772	0.35571

C	-1.98876	0.81655	-0.08666
C	-4.37162	0.16499	0.26303
C	-5.34311	-0.78121	0.67306
C	-4.97404	-1.98565	1.23313
C	-3.60646	-2.2678	1.42333
H	3.15764	-2.11552	-0.93816
H	1.83296	-3.95973	-1.89678
H	-6.39396	-0.52186	0.56715
H	-5.72936	-2.69684	1.55606
C	2.22183	0.30556	-0.09706
C	0.32577	1.64841	-0.18622
C	-0.15867	2.98428	-0.2674
C	-1.4961	3.217	-0.40949
C	-2.43613	2.15118	-0.29772
H	0.57387	3.78483	-0.25359
H	-1.8731	4.22736	-0.54699
C	-4.76983	1.47028	-0.16209
H	-5.83092	1.69315	-0.24178
C	-3.83584	2.43869	-0.37081
H	-4.13698	3.45724	-0.60321
H	-0.63697	-3.69707	-2.1593
H	-3.30627	-3.18673	1.91962
H	-1.59837	-1.60743	1.17871
H	-1.75161	-1.65869	-1.39819
C	6.38833	0.19275	0.96538
C	5.87594	1.24609	0.20326
C	4.52774	1.26915	-0.14421
C	3.66871	0.23003	0.24926
C	4.19173	-0.81882	1.02299
C	5.5404	-0.83497	1.38006
H	7.44001	0.17699	1.23889
H	6.52925	2.05269	-0.11902
H	4.11765	2.09121	-0.72135
H	3.53466	-1.61112	1.36936
H	5.92573	-1.64872	1.98874
N	1.68081	1.49846	-0.04503

H	2.25876	2.30186	0.09817

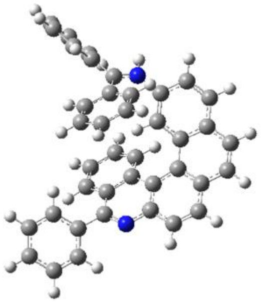

10b+H

Charge = 1 Multiplicity = 1

Symbol	X	Y	Z
C	2.28614	-1.73538	-2.20422
C	2.43567	-0.69242	-1.25614
C	1.61194	0.47176	-1.35356
C	0.71127	0.55336	-2.44649
C	0.58509	-0.47764	-3.35539
C	1.36723	-1.64107	-3.22901
C	1.85035	1.57629	-0.43709
C	-1.23401	1.58608	-0.41667
C	-0.44347	2.76029	-0.40095
C	1.00812	2.73856	-0.24744
C	-1.12463	4.01874	-0.4094
C	-2.52958	4.04462	-0.59727
C	-3.26114	2.88538	-0.70225
C	-2.61716	1.63011	-0.55405
H	2.93735	-2.59951	-2.14152
H	1.27405	-2.44457	-3.95427
H	-3.02852	5.00991	-0.64291
H	-4.33539	2.90606	-0.85882
C	3.49618	-0.69667	-0.26707
C	3.11306	1.54636	0.2111
C	3.66164	2.73307	0.77389
C	2.97379	3.90878	0.68856
C	1.63316	3.93595	0.2035

H	4.64672	2.66565	1.22431
H	3.41236	4.83649	1.04778
C	-0.40141	5.22202	-0.15463
H	-0.93599	6.16859	-0.17873
C	0.91324	5.17165	0.20068
H	1.44573	6.07557	0.48592
H	-0.10566	-0.37532	-4.18802
H	-0.75838	0.62026	-0.31404
H	0.13257	1.45681	-2.58755
C	5.76565	-4.17767	0.84791
C	6.3937	-2.93996	0.68472
C	5.65294	-1.82081	0.31346
C	4.27119	-1.92032	0.08181
C	3.64852	-3.16673	0.25858
C	4.38995	-4.2852	0.64074
H	6.34415	-5.05012	1.14001
H	7.46408	-2.84712	0.84912
H	6.12949	-0.85273	0.2005
H	2.57484	- 3.25535	0.12158
H	3.88936	-5.23945	0.78261
N	3.86408	0.40677	0.33764
N	-3.40787	0.47824	-0.67775
C	-3.42765	-0.49947	0.15959
C	-1.85328	-1.54042	1.83718
C	-1.19184	-1.52868	3.06528
C	-1.41548	-0.49055	3.97121
C	-2.29813	0.5398	3.64022
C	-2.94556	0.53884	2.40586
C	-2.73013	-0.50123	1.48955
H	-1.68264	-2.3538	1.13736
H	-0.50363	-2.33178	3.31464
H	-0.9047	-0.48421	4.93022
H	-2.48007	1.34864	4.3426
H	-3.62326	1.34691	2.14771
C	-5.78458	-3.91907	-0.9729
C	-5.4633	-3.71337	0.36875

C	-4.68888	-2.61514	0.7445
C	-4.23048	-1.70055	-0.21743
C	-4.56783	-1.91477	-1.56573
C	-5.33351	-3.01427	-1.93927
H	-6.38245	-4.77807	-1.26606
H	-5.81651	-4.40668	1.12737
H	-4.45062	-2.461	1.79161
H	-4.21801	-1.20206	-2.30476
H	-5.57896	-3.16977	-2.98659
H	-4.00264	0.41121	-1.47884

6. References

1. Lefebvre, Q.; Jentsch, M.; Rueping, M. Continuous Flow Photocyclization Of Stilbenes-Scalable Synthesis Of Functionalized Phenanthrenes And Helicenes. *Beilstein J. Org. Chem.* **2013**, *9*, 1883–1890, doi:10.3762/bjoc.9.221.
2. Jakubec, M.; Beránek, T.; Jakubík, P.; Sýkora, J.; Žádný, J.; Církva, V.; Storch, J. 2-Bromo[6]Helicene As A Key Intermediate For [6]Helicene Functionalization. *J. Org. Chem.* **2018**, *83*, 3607–3616, doi:10.1021/acs.joc.7b03234.
3. Talele, H.R.; Chaudhary, A.R.; Patel, P.R.; Bedekar, A. V. Expeditious Synthesis Of Helicenes Using An Improved Protocol Of Photocyclodehydrogenation Of Stilbenes. *Arkivoc* **2011**, *2011*, 15, doi:10.3998/ark.5550190.0012.902.
4. Hacker, A.S.; Pavano, M.; Wood, J.E.; Immoos, C.E.; Hashimoto, H.; Genis, S.P.; Frantz, D.K. Synthesis And Electronic Properties Of Fluoreno[2,1-A]Fluorenedione And Fluoreno[1,2-A]Fluorenedione. *J. Org. Chem.* **2018**, *83*, 510–515, doi:10.1021/acs.joc.7b02699.
5. Sheldrick, G.M. Crystal Structure Refinement With SHELXL. *Acta Crystallogr. Sect. C Struct. Chem.* **2015**, *71*, 3–8, doi:10.1107/S2053229614024218.
6. Betteridge, P.W.; Carruthers, J.R.; Cooper, R.I.; Prout, K.; Watkin, D.J. CRYSTALS Version 12: Software For Guided Crystal Structure Analysis. *J. Appl. Crystallogr.* **2003**, *36*, 1487–1487, doi:10.1107/S0021889803021800.
7. Farrugia, L.J. ORTEP -3 For Windows - A Version Of ORTEP -III With A Graphical User Interface (GUI). *J. Appl. Crystallogr.* **1997**, *30*, 565–565, doi:10.1107/S0021889897003117.
8. Žádný, J.; Velíšek, P.; Jakubec, M.; Sýkora, J.; Církva, V.; Storch, J. Exploration Of 9-Bromo[7]Helicene Reactivity. *Tetrahedron* **2013**, *69*, 6213–6218, doi:10.1016/j.tet.2013.05.039.
9. Amal Joseph, P.J.; Priyadarshini, S.; Lakshmi Kantam, M.; Maheswaran, H. Copper Catalyzed Ipso-Nitration Of Iodoarenes, Bromoarenes And Heterocyclic Haloarenes Under Ligand-Free Conditions. *Tetrahedron Lett.* **2012**, *53*, 1511–1513, doi:10.1016/j.tetlet.2012.01.056.
10. Huang, X.; Anderson, K.W.; Zim, D.; Jiang, L.; Klapars, A.; Buchwald, S.L. Expanding Pd-Catalyzed C-N Bond-Forming Processes: The First Amidation Of Aryl Sulfonates, Aqueous Amination, And Complementarity With Cu-Catalyzed Reactions. *J. Am. Chem. Soc.* **2003**, *125*, 6653–6655, doi:10.1021/ja035483w.
11. Van As, D.J.; Connell, T.U.; Brzozowski, M.; Scully, A.D.; Polyzos, A. Photocatalytic And Chemoselective Transfer Hydrogenation Of Diarylimines In Batch And Continuous Flow. *Org. Lett.* **2018**, *20*, 905–908, doi:10.1021/acs.orglett.7b03565.
12. Rudolphi, F.; Song, B.; Gooßen, L.J. Synthesis Of Azomethines From A -Oxocarboxylates , Amines And Aryl Bromides Via One-Pot Three-Component Decarboxylative Coupling. *Adv. Synth. Catal.* **2011**, *353*, 337–342, doi:10.1002/adsc.201000798.
13. Theodorou, V.; Skobridis, K.; Karkatsoulis, A. Base-Induced Rearrangement Of Tritylamines To Imines: Discovery And Investigation Of The Mechanism. *Tetrahedron* **2007**, *63*, 4284–4289, doi:10.1016/j.tet.2007.03.055.
14. Liu, S.; Yu, Y.; Liebeskind, L.S. N-Substituted Imines By The Copper-Catalyzed N-Imination Of Boronic Acids And Organostannanes With O-Acyl Ketoximes. *Org. Lett.* **2007**, *9*, 1947–1950, doi:10.1021/ol070561w.
15. Albert, J.; Granell, J.; Qadir, R.; Quirante, J.; Calvis, C.; Messeguer, R.; Badía, J.; Baldomà, L.;

Font-Bardia, M.; Calvet, T. Cyclopalladated Benzophenone Imines: Synthesis, Antitumor Activity, Cell Accumulation, DNA Interaction, And Cathepsin B Inhibition. *Organometallics* **2014**, *33*, 7284–7292, doi:10.1021/om501060f.

16. Pan, C.; Zhang, H.; Han, J.; Cheng, Y.; Zhu, C. Metal-Free Radical Oxidative Decarboxylation/Cyclization Of Acyl Peroxides And 2-Isocyanobiphenyls. *Chem. Commun.* **2015**, *51*, 3786–3788, doi:10.1039/c4cc10015h.
17. Jaiswal, Y.; Kumar, Y.; Pal, J.; Subramanian, R.; Kumar, A. Rapid Synthesis Of Polysubstituted Phenanthridines From Simple Aliphatic/Aromatic Nitriles And Iodo Arenes: Via Pd(II) Catalyzed Domino C-C/C-C/C-N Bond Formation. *Chem. Commun.* **2018**, *54*, 7207–7210, doi:10.1039/c8cc03556c.
18. Tobisu, M.; Koh, K.; Furukawa, T.; Chatani, N. Modular Synthesis Of Phenanthridine Derivatives By Oxidative Cyclization Of 2-Isocyanobiphenyls With Organoboron Reagents. *Angew. Chem. Int. Ed.* **2012**, *51*, 11363–11366, doi:10.1002/anie.201206115.
19. Li, X.; Liang, D.; Huang, W.; Sun, H.; Wang, L.; Ren, M.; Wang, B.; Ma, Y. Metal-Free Photocatalyzed Cross Coupling Of Aryl (Heteroaryl) Bromides With Isonitriles. *Tetrahedron* **2017**, *73*, 7094–7099, doi:10.1016/j.tet.2017.10.074.
20. Ge, J.; Wang, X.; Liu, T.; Shi, Z.; Xiao, Q.; Yin, D. Assembly Of Substituted Phenanthridines Via A Cascade Palladium-Catalyzed Coupling Reaction, Deprotection And Intramolecular Cyclization. *RSC Adv.* **2016**, *6*, 19571–19575, doi:10.1039/c6ra00249h.
21. Tang, C.; Yuan, Y.; Jiao, N. Metal-Free Nitrogenation Of 2-Acetylbiphenyls: Expeditious Synthesis Of Phenanthridines. *Org. Lett.* **2015**, *17*, 2206–2209, doi:10.1021/acs.orglett.5b00797.
22. Zheng, Y.-H.; Lu, H.-Y.; Li, M.; Chen, C.-F. Synthesis, Structures, And Optical Properties Of Aza[4]Helicenes. *Eur. J. Org. Chem.* **2013**, *2013*, 3059–3066, doi:10.1002/ejoc.201300002.
23. Maestri, G.; Lacôte, E.; Ollivier, C.; Larraufie, M.-H.; Derat, É.; Malacria, M.; Fensterbank, L. Expeditious Synthesis Of Phenanthridines From Benzylamines Via Dual Palladium Catalysis. *Org. Lett.* **2010**, *12*, 5692–5695, doi:10.1021/ol102509n.
24. Li, H.; He, K.-H.; Liu, J.; Wang, B.-Q.; Zhao, K.-Q.; Hu, P.; Shi, Z.-J. Straightforward Synthesis Of Phenanthrenes From Styrenes And Arenes. *Chem. Commun.* **2012**, *48*, 7028–7030, doi:10.1039/c2cc33100d.
25. Biju, A.T.; Glorius, F. Intermolecular N-Heterocyclic Carbene Catalyzed Hydroacylation Of Arynes. *Angew. Chem. Int. Ed.* **2010**, *49*, 9761–9764, doi:10.1002/anie.201005490.
26. Zhou, Q.; Wei, S.; Han, W. In Situ Generation Of Palladium Nanoparticles: Ligand-Free Palladium Catalyzed Pivalic Acid Assisted Carbonylative Suzuki Reactions At Ambient Conditions. *J. Org. Chem.* **2014**, *79*, 1454–1460, doi:10.1021/jo402366p.
27. Zhu, S.; Zhang, Z.; Huang, X.; Jiang, H.; Guo, Z. Gold-Catalyzed Reactions Of Enynals/Enynones With Norbornenes: Generation And Trapping Of Cyclic O-Quinodimethanes (O-QDMs). *Chem. Eur. J.* **2013**, *19*, 4695–4700, doi:10.1002/chem.201300232.

7. Abbreviations

APCI	Atmospheric pressure chemical ionization
dba	Dibenzylidenaceton
DCM	Dichloromethane
DMSO	Dimethyl sulfoxide
ESI	Electrospray ionization
GC-MS	Gas chromatography–mass spectrometry
HOMO	Highest occupied molecular orbital
IR	Infrared
LUMO	Lowest unoccupied molecular orbital
M.S.	Molecular sieves
NMP	N-Methyl-2-pyrrolidone
NMR	Nuclear magnetic resonance
NOESY	Nuclear Overhauser effect spectroscopy
ORTEP	Oak ridge thermal ellipsoid plot
PAH	Polycyclic aromatic hydrocarbon
PE	Petroleum ether
PTFE	Polytetrafluoroethylene
TD-DFT	Time-dependent density functional theory
TEMPO	2,2,6,6-Tetramethyl-1-piperidinyloxy
THF	Tetrahydrofuran
TLC	Thin-layer chromatography
TMS	Tetramethylsilane
TOF	Time of flight
UV	Ultraviolet
XPhos	2-Dicyclohexylphosphino-2′,4′,6′-triisopropylbiphenyl

8. NMR Spectra

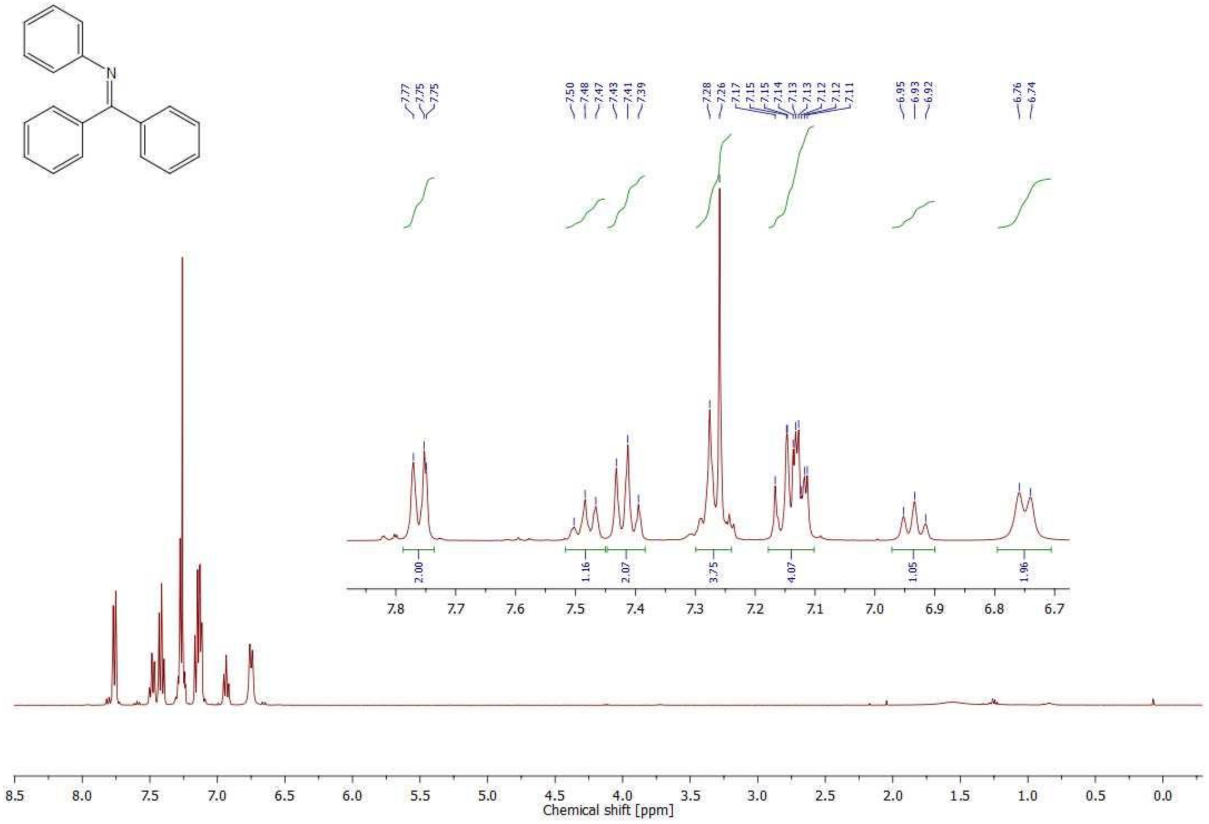

Figure S17.^{1}H NMR spectrum of compound **1** (400 MHz, $CDCl_3$)

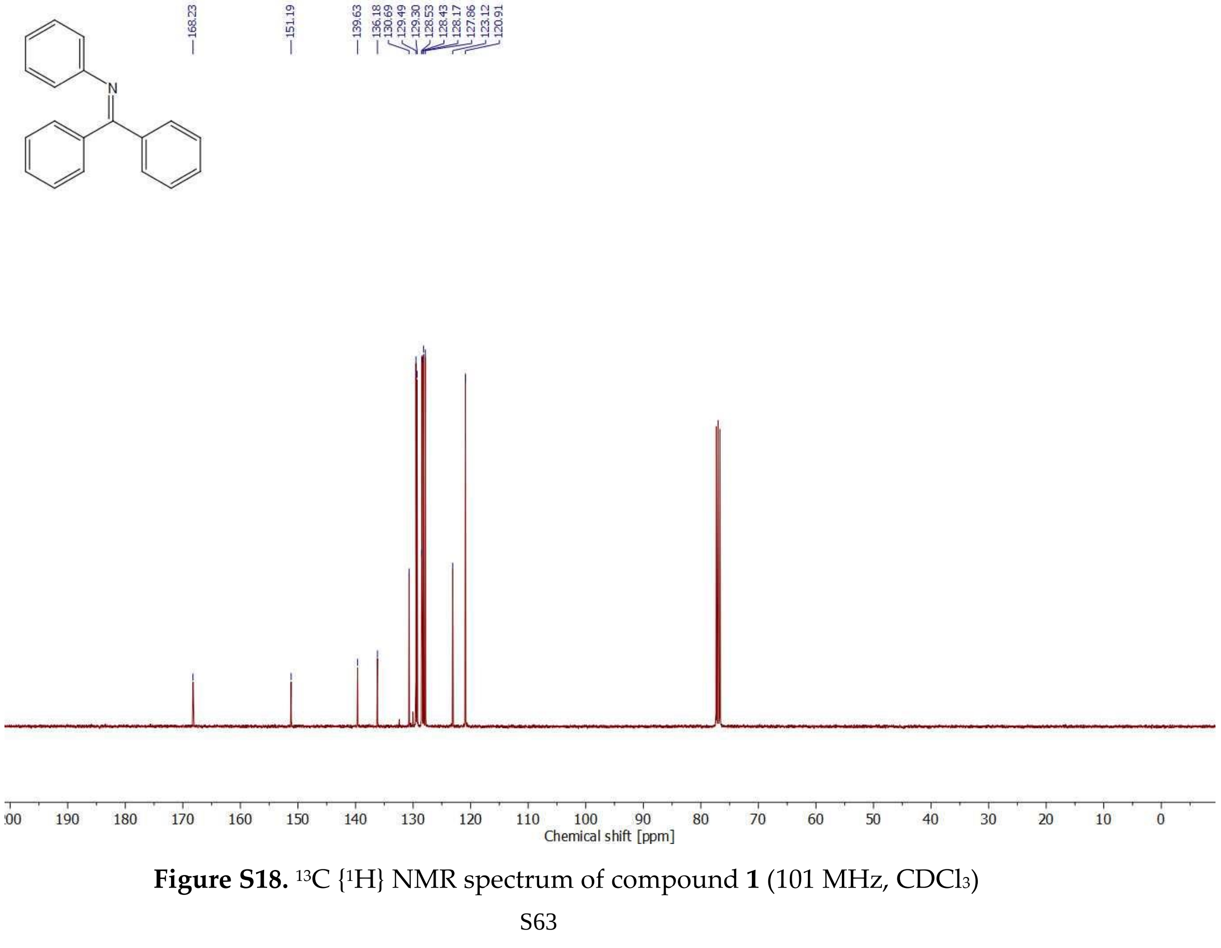

Figure S18. ^{13}C {^{1}H} NMR spectrum of compound **1** (101 MHz, $CDCl_3$)

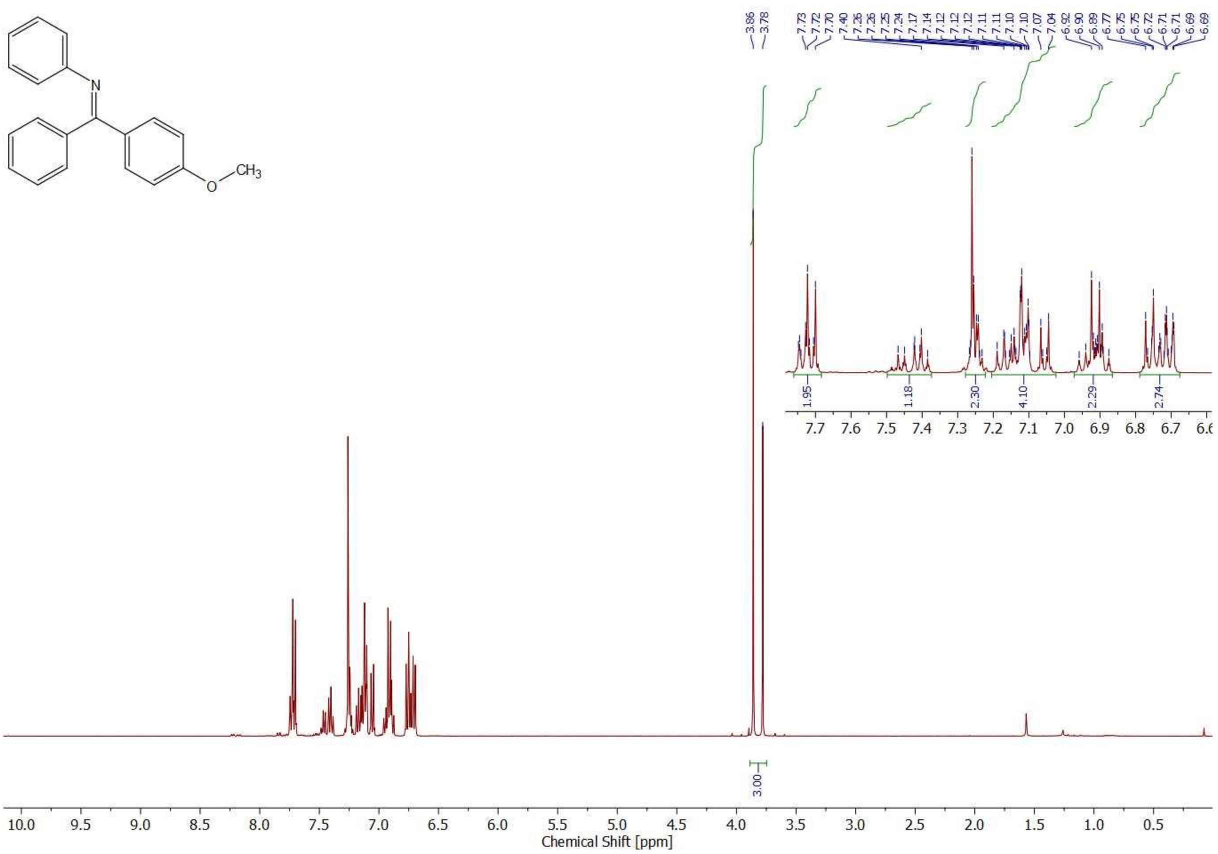

Figure S19. ^{1}H NMR spectrum of compound **1a** (400 MHz, $CDCl_3$)

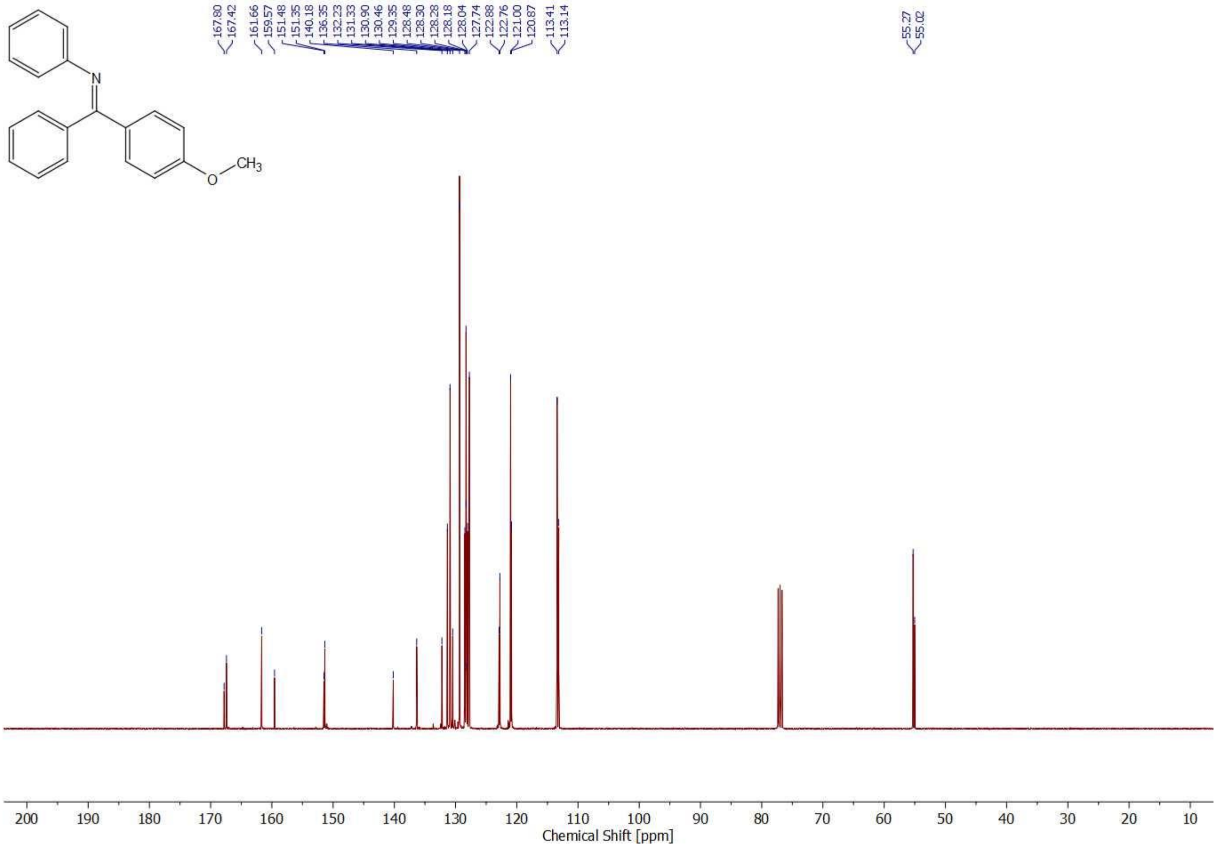

Figure S20. ^{13}C {^{1}H} NMR spectrum of compound **1a** (101 MHz, $CDCl_3$)

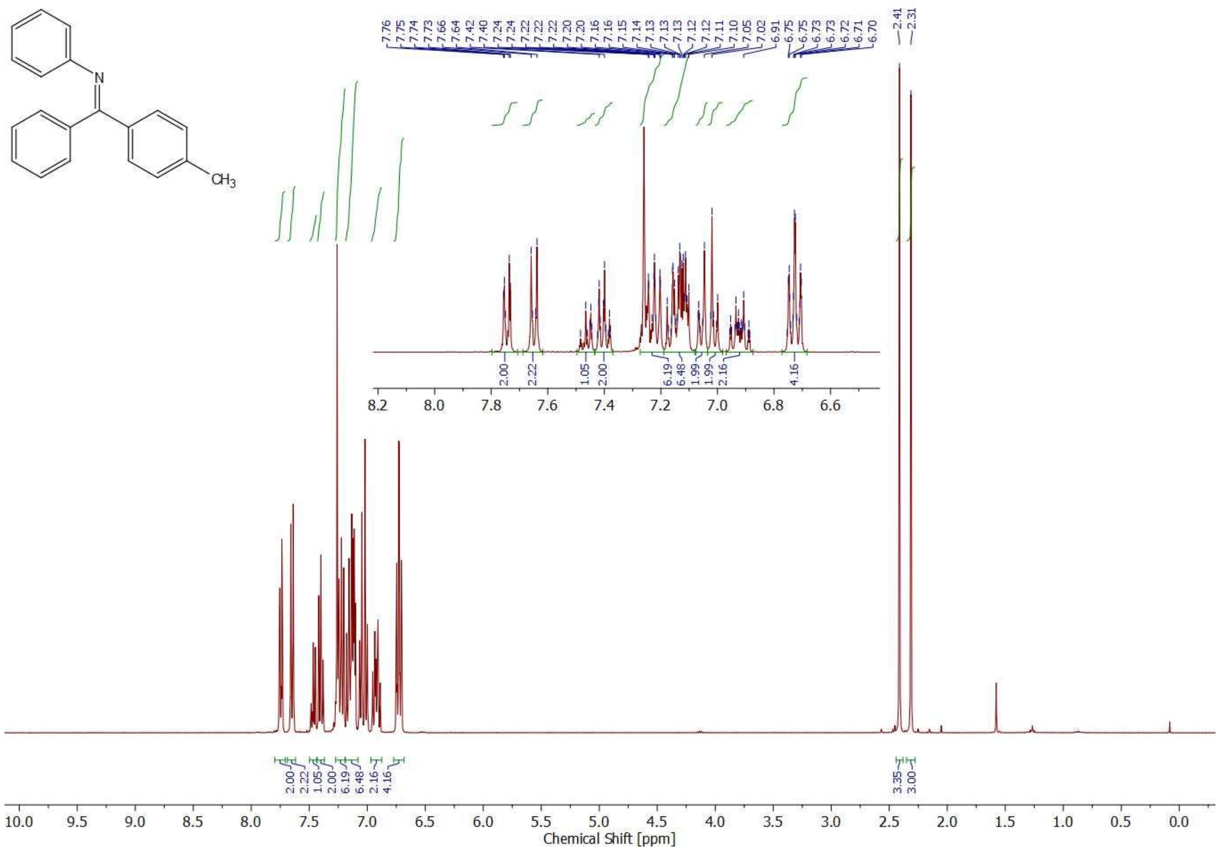

Figure S21. ^{1}H NMR spectrum of compound **1b** (400 MHz, $CDCl_3$)

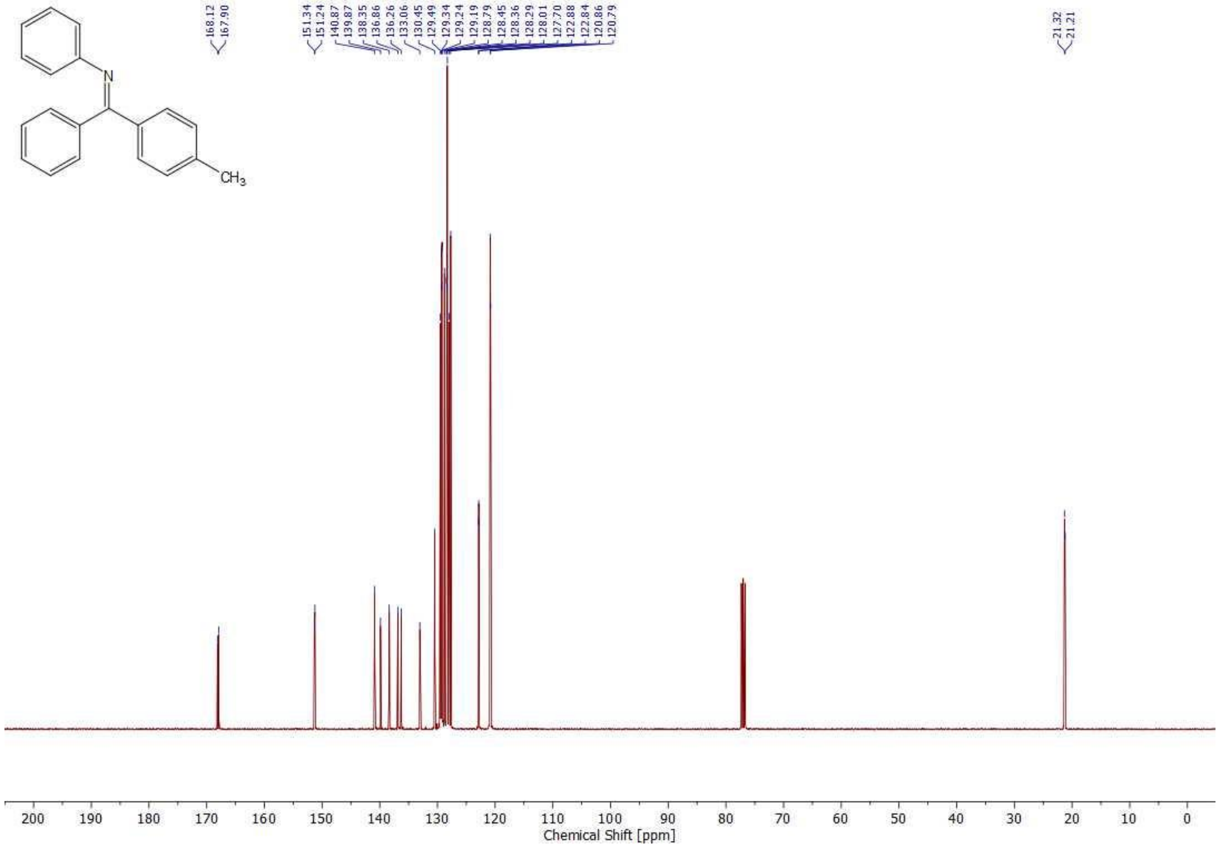

Figure S22. ^{13}C {^{1}H} NMR spectrum of compound **1b** (101 MHz, $CDCl_3$)

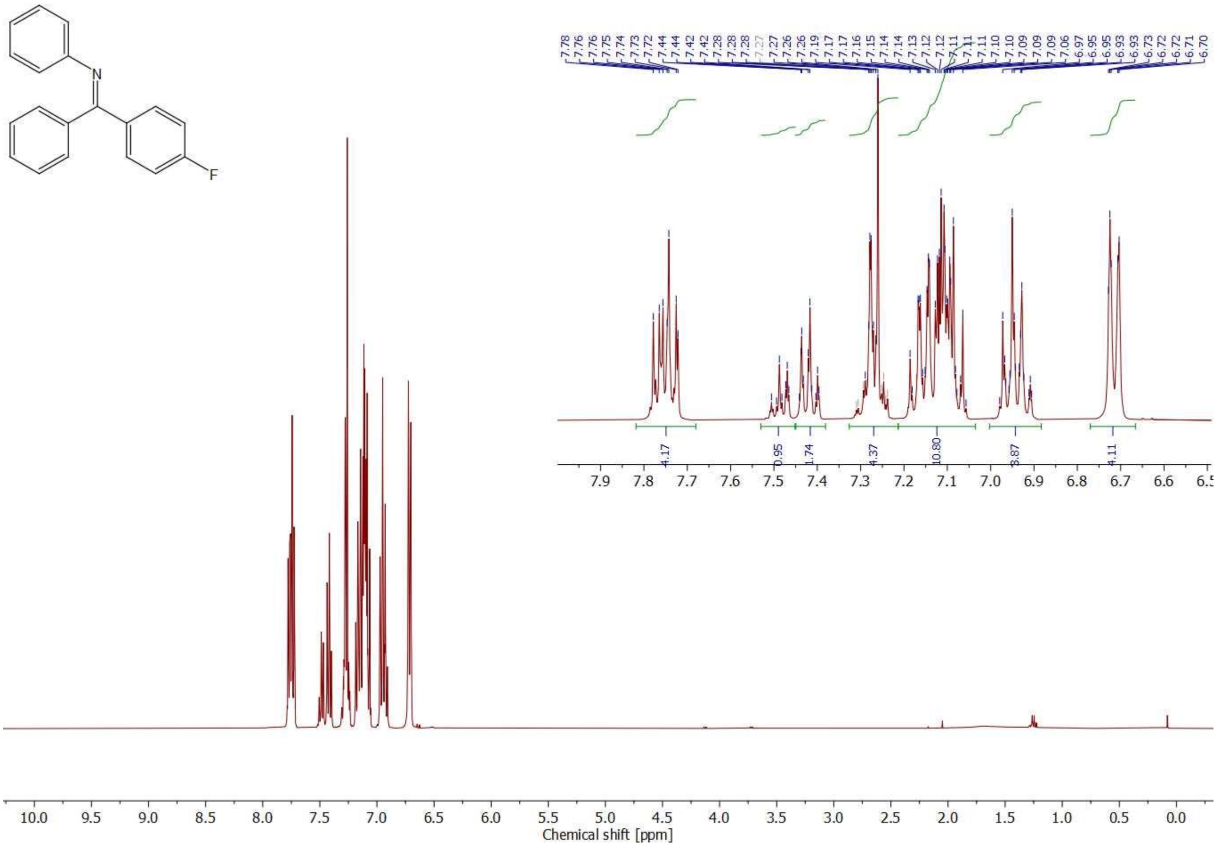

Figure S23. ^{1}H NMR spectrum of compound **1c** (400 MHz, $CDCl_3$)

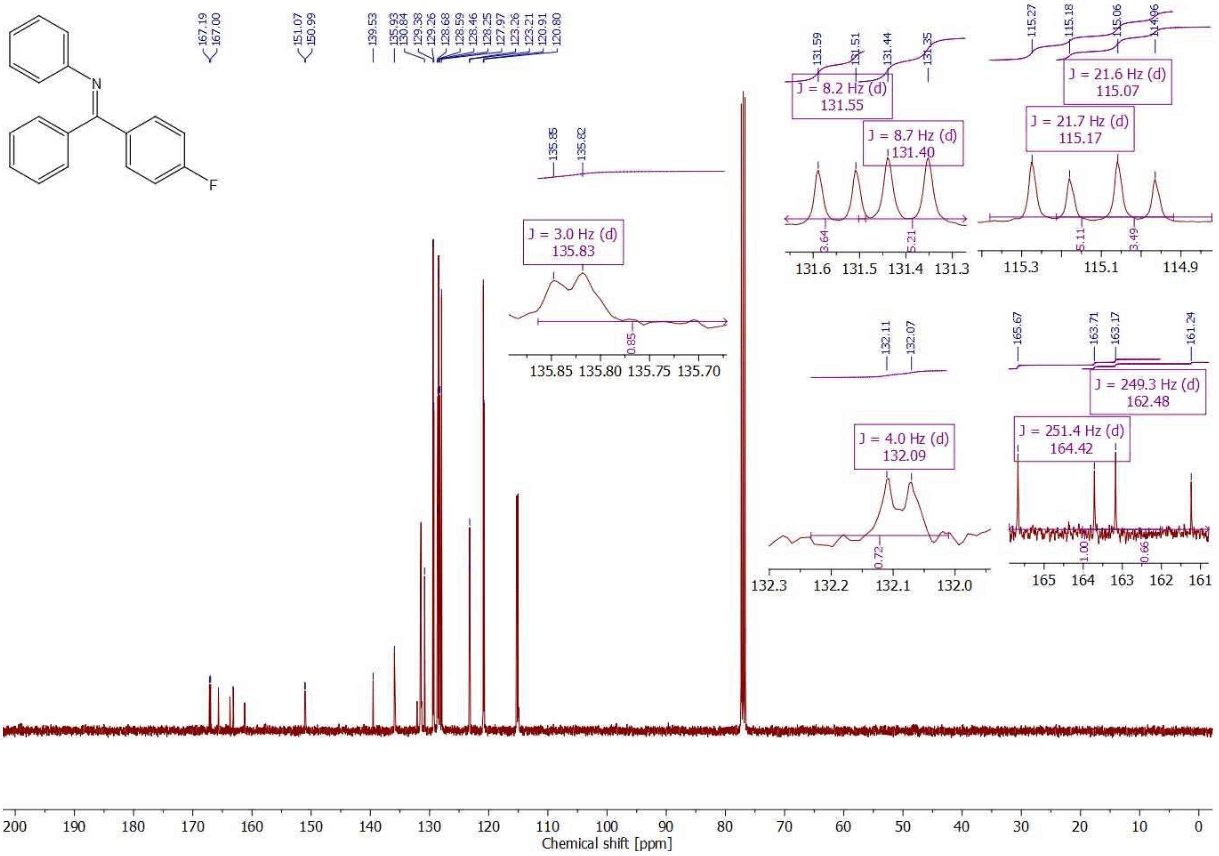

Figure S24. ^{13}C {^{1}H} NMR spectrum of compound **1c** (101 MHz, $CDCl_3$)

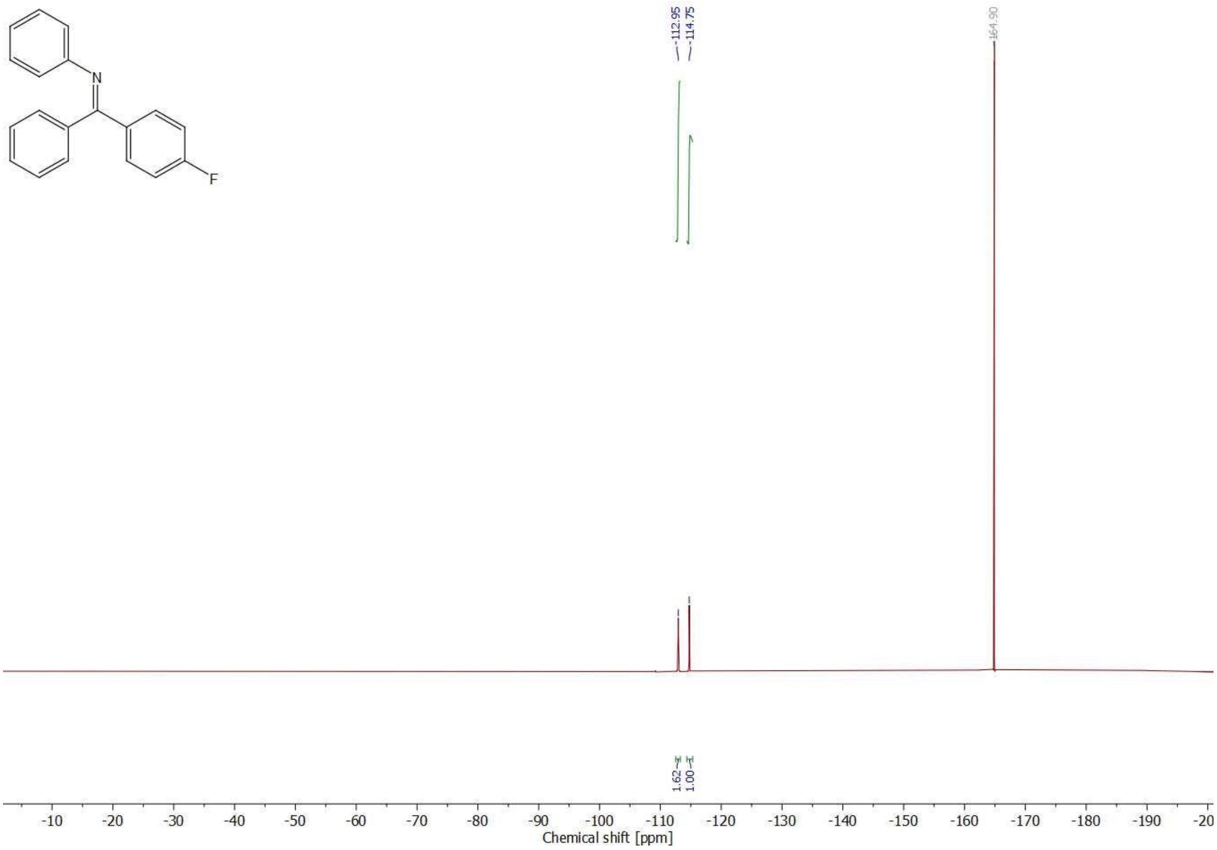

Figure S25. ^{19}F {^{1}H} NMR spectrum of compound **1d** (376 MHz, $CDCl_3$, C_6F_6)

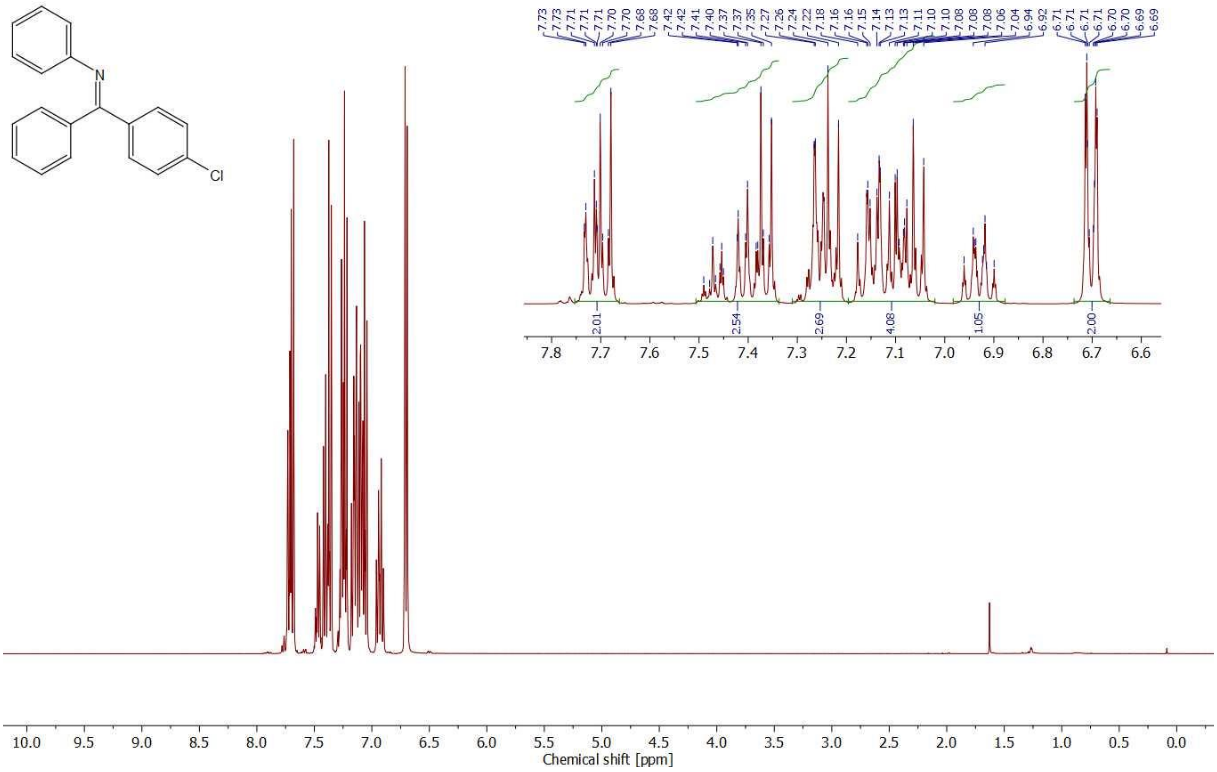

Figure S26. ^{1}H NMR spectrum of compound **1d** (400 MHz, $CDCl_3$)

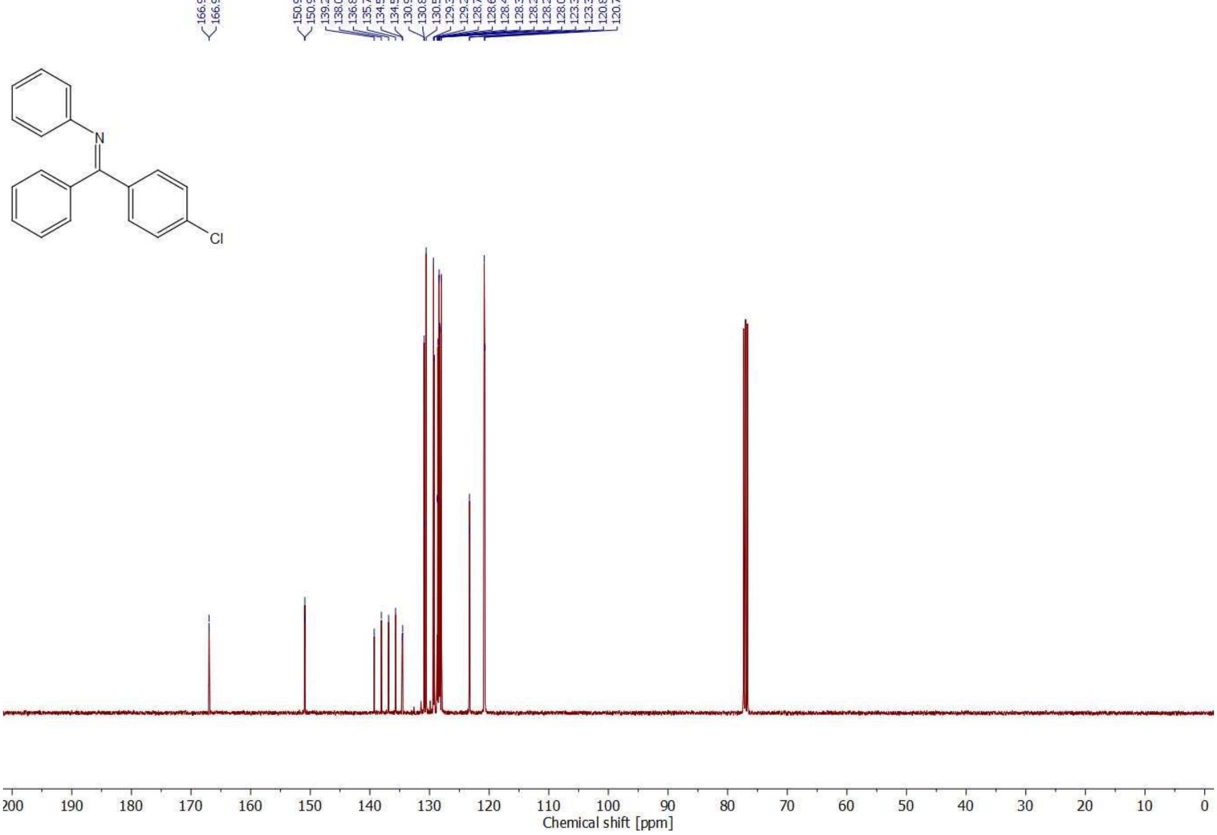

Figure S27. ^{13}C {^{1}H} NMR spectrum of compound **1d** (101 MHz, $CDCl_3$)

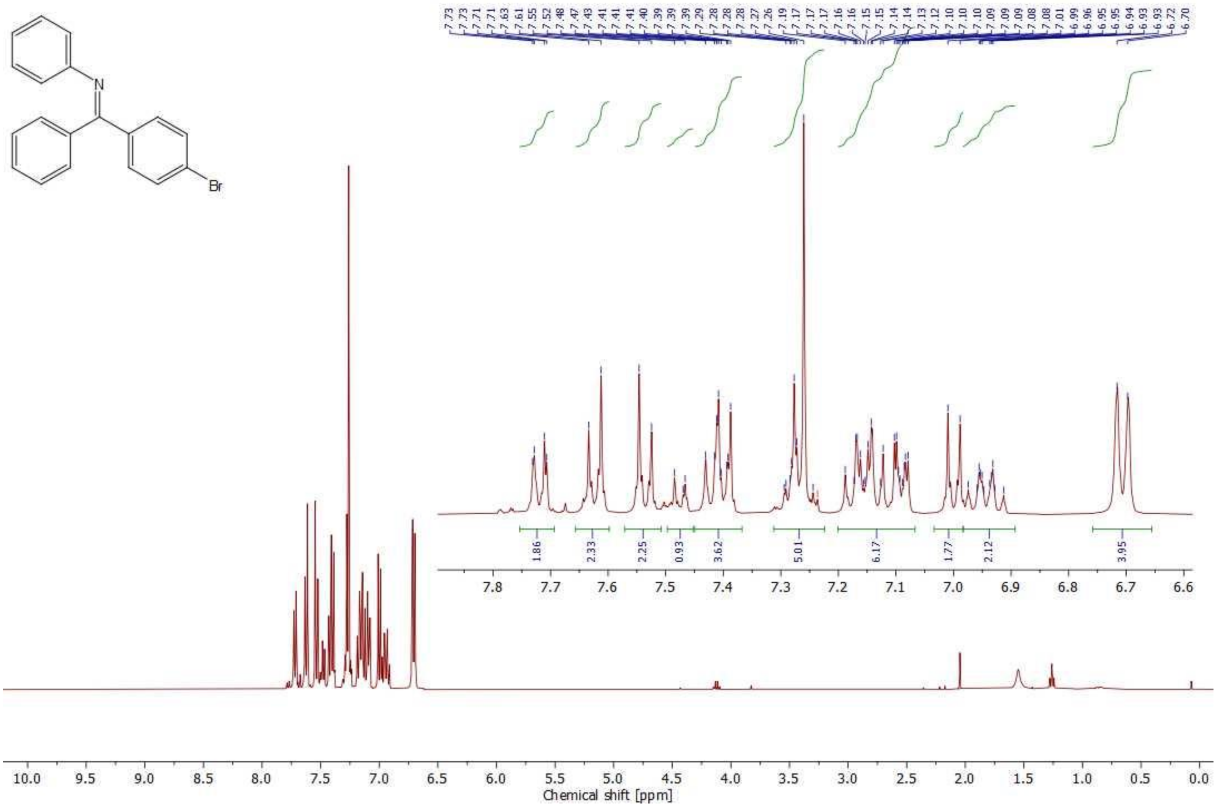

Figure S28. ^{1}H NMR spectrum of compound **1e** (400 MHz, $CDCl_3$)

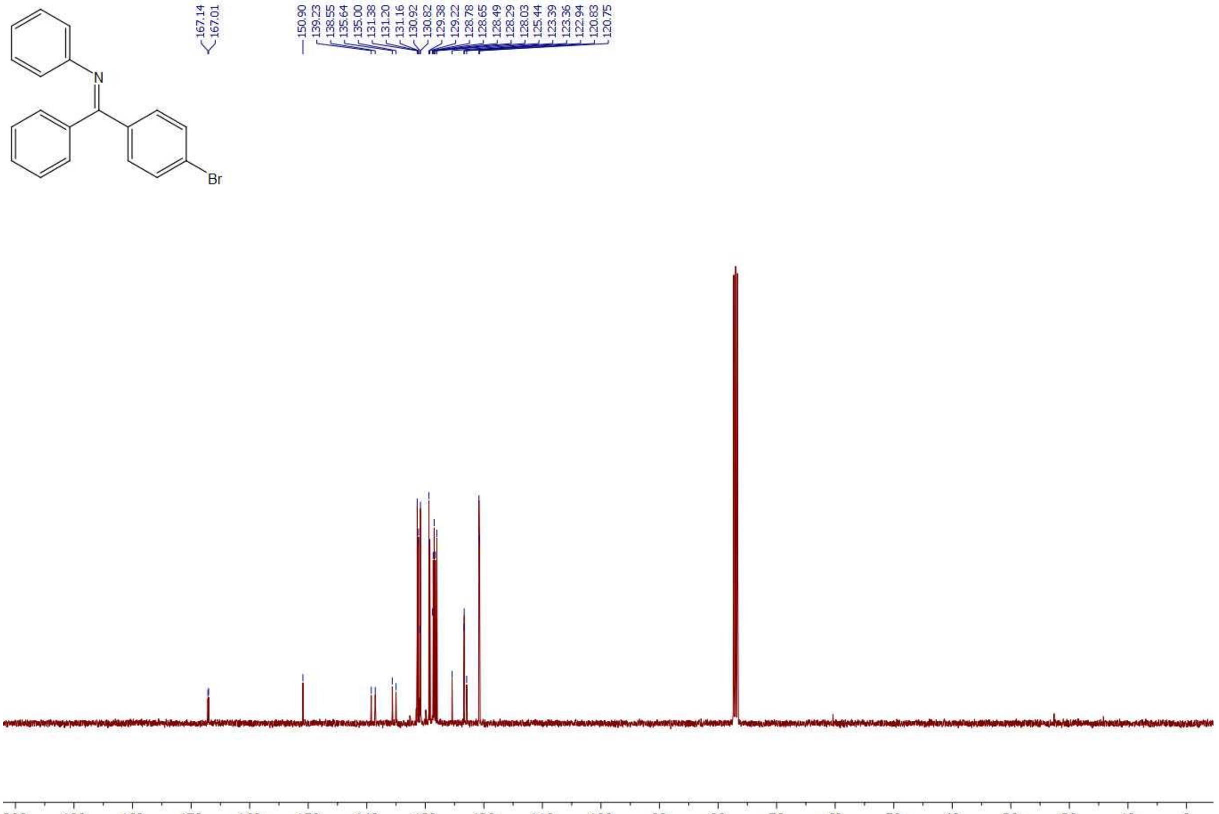

Figure S29. ^{13}C {^{1}H} NMR spectrum of compound **1e** (101 MHz, $CDCl_3$)

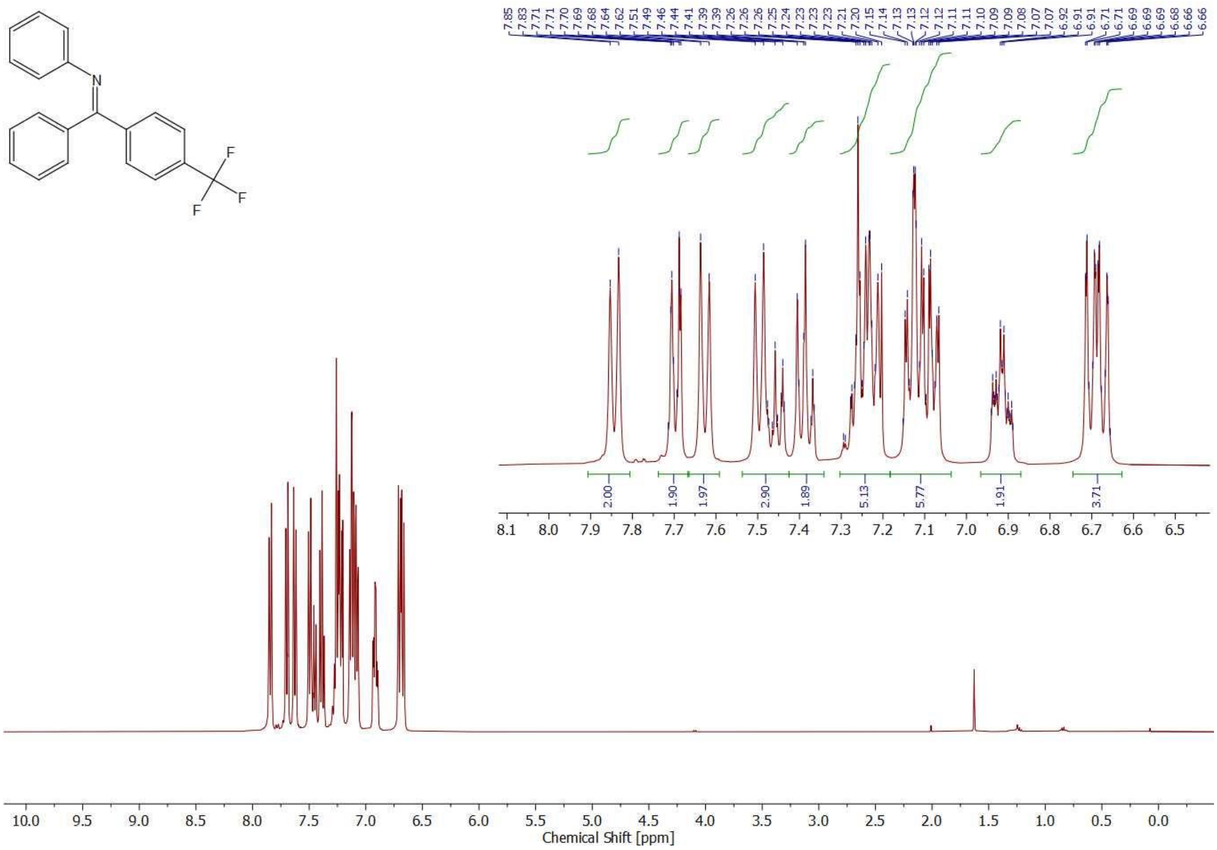

Figure S30. ^{1}H NMR spectrum of compound **1f** (400 MHz, $CDCl_3$)

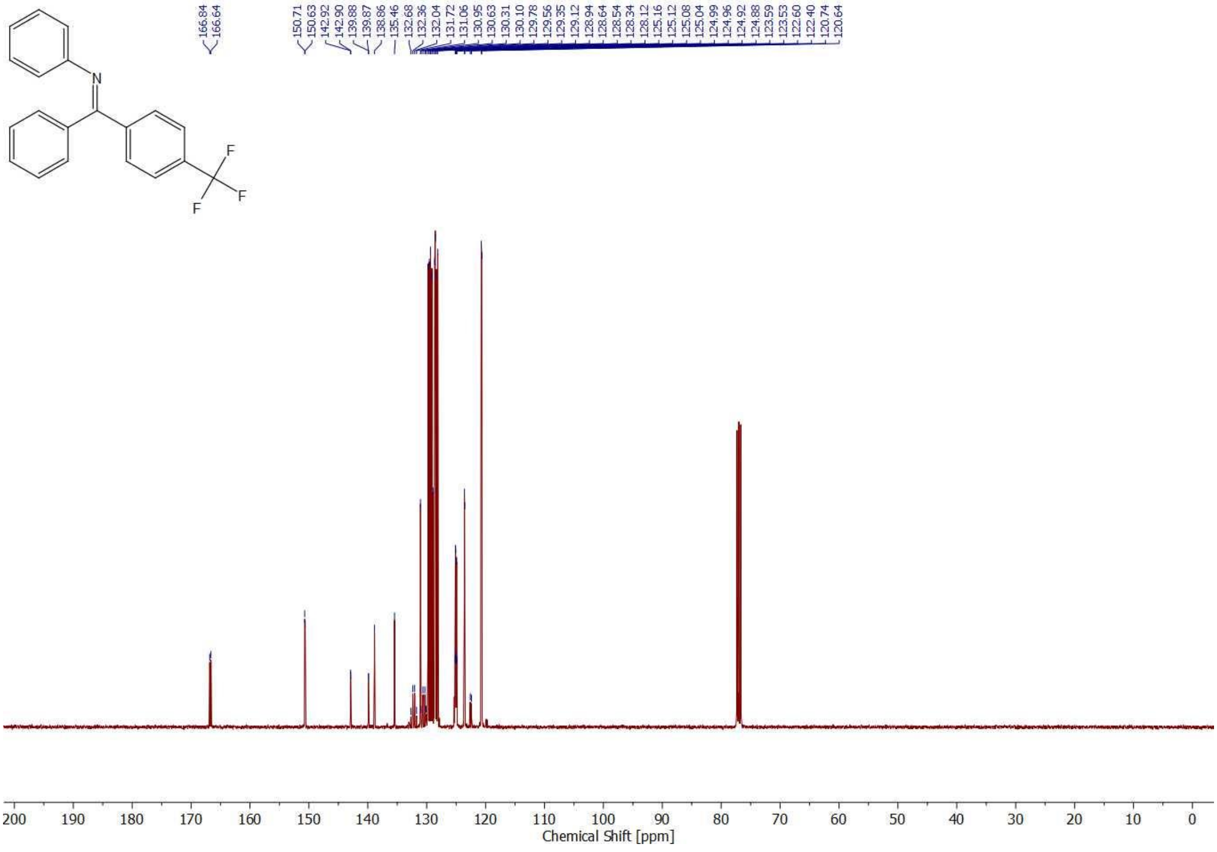

Figure S31. ^{13}C {^{1}H} NMR spectrum of compound **1f** (101 MHz, $CDCl_3$)

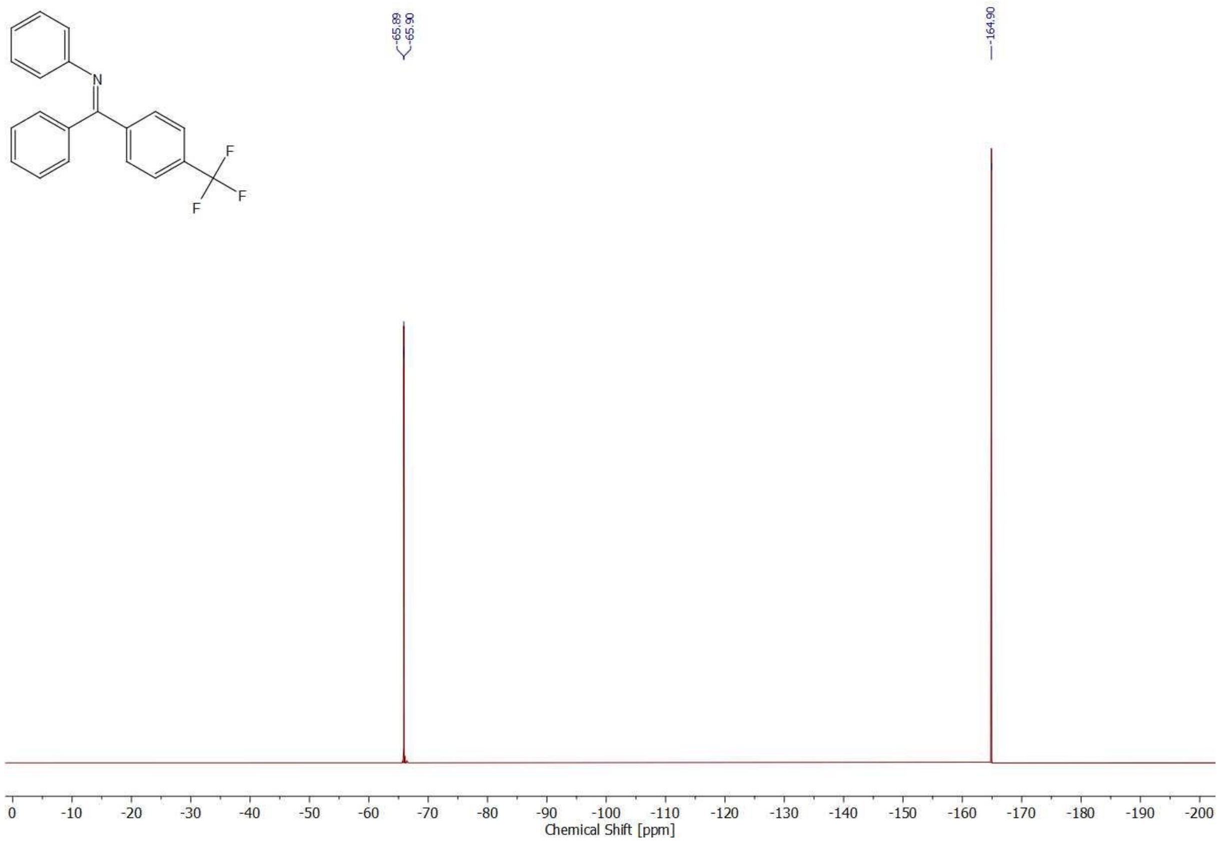

Figure S32. ^{19}F {^{1}H} NMR spectrum of compound **1f** (376 MHz, $CDCl_3$, C_6F_6)

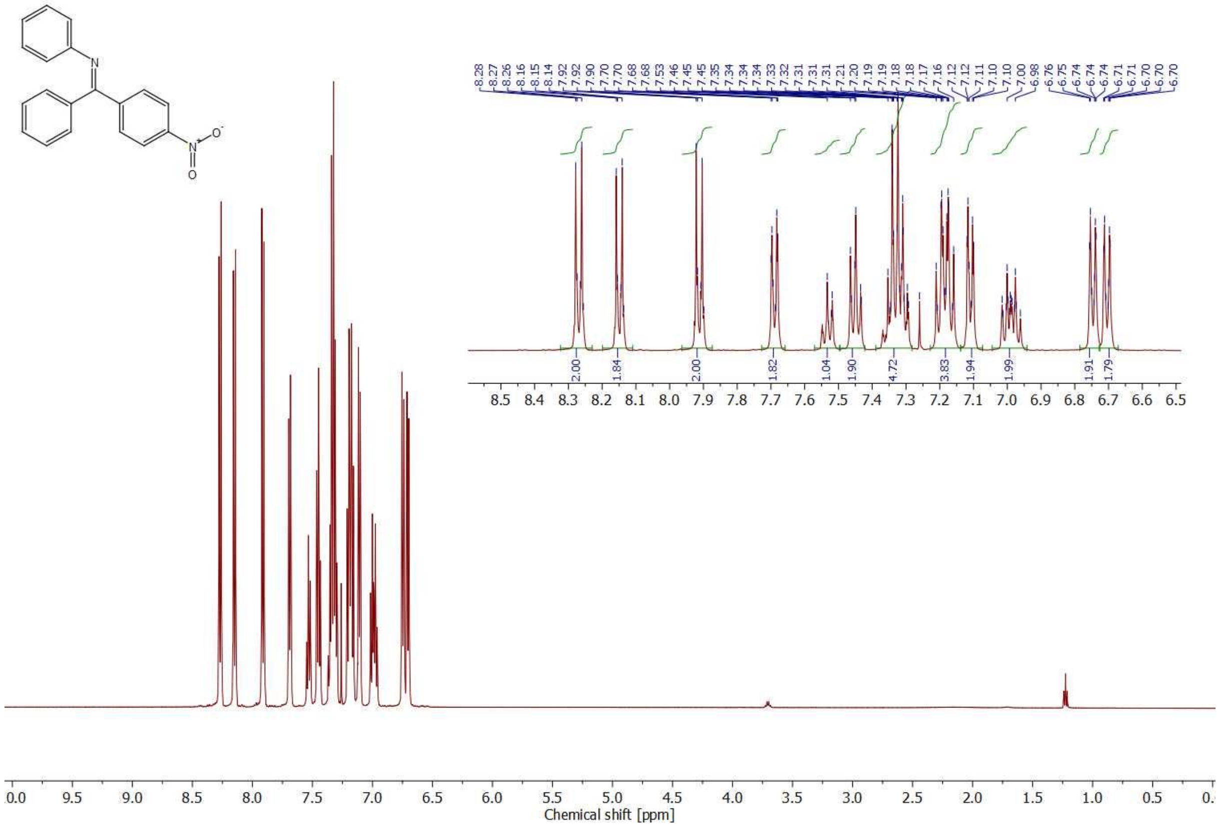

Figure S33. ^{1}H NMR spectrum of compound **1g** (500 MHz, $CDCl_3$)

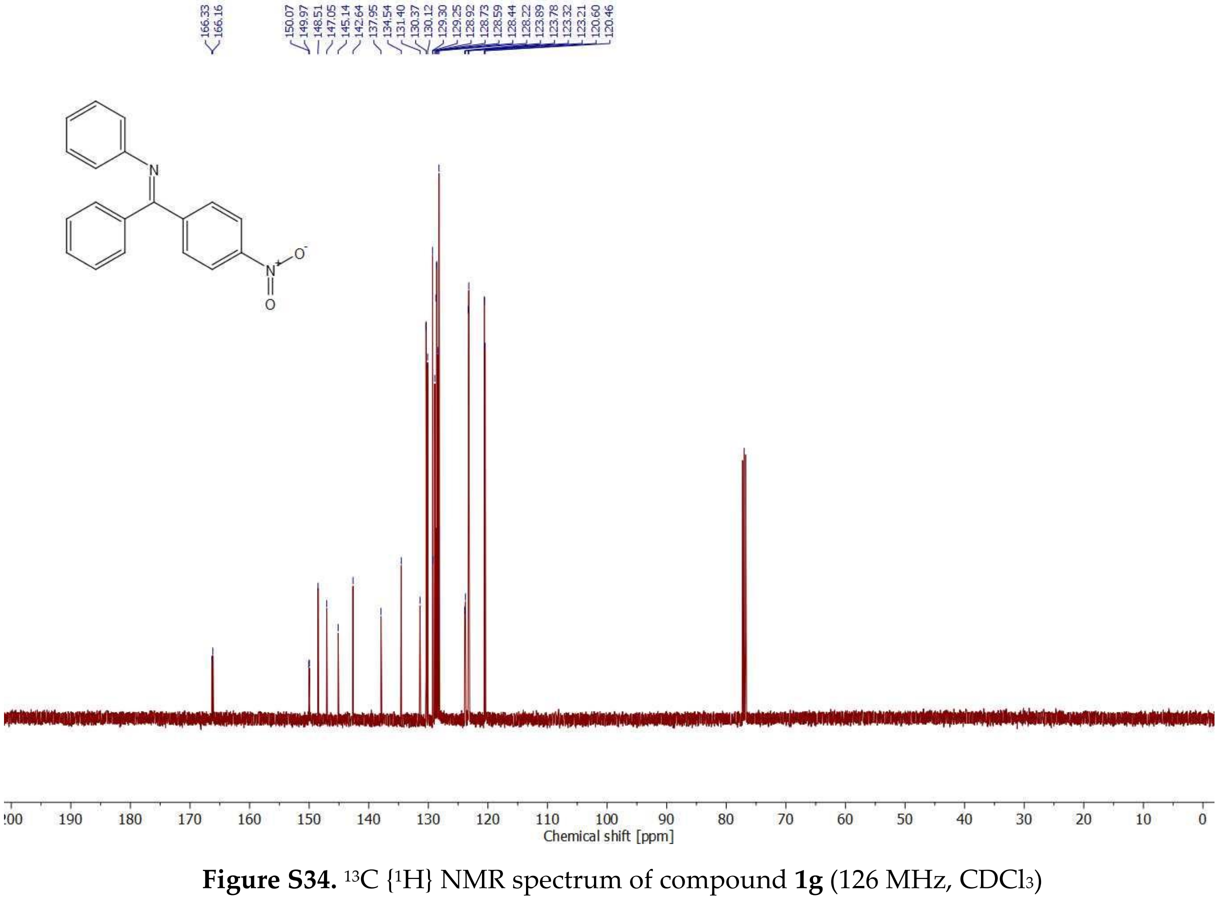

Figure S34. ^{13}C {^{1}H} NMR spectrum of compound **1g** (126 MHz, $CDCl_3$)

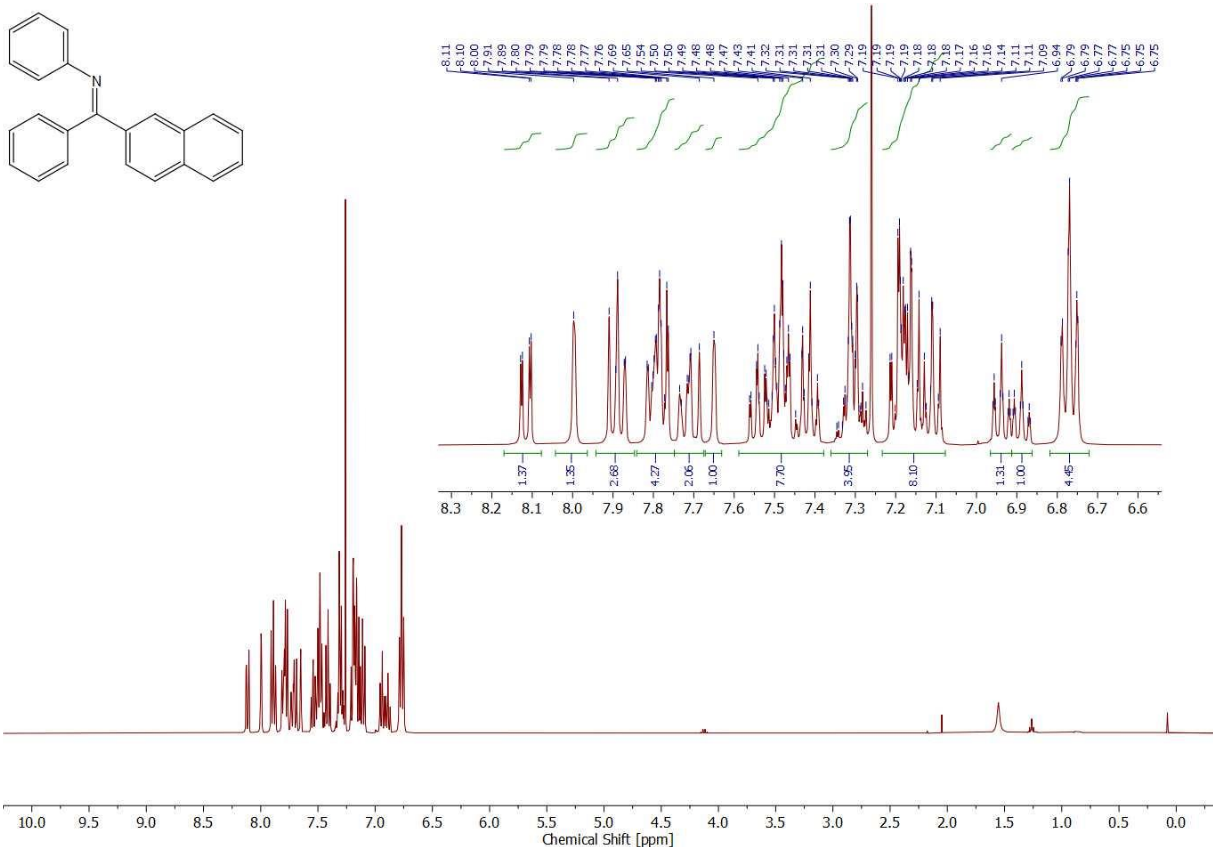

Figure S35. ^{1}H NMR spectrum of compound **1h** (400 MHz, $CDCl_3$)

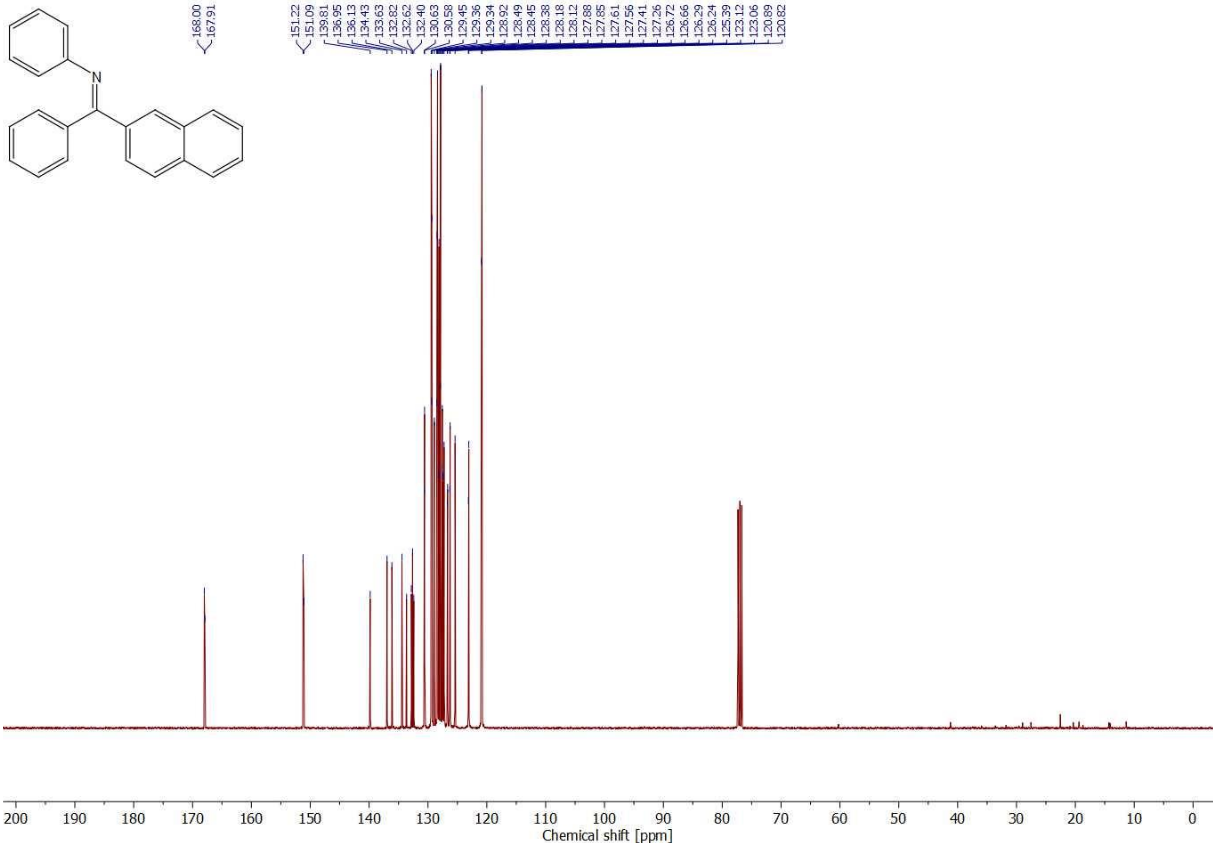

Figure S36. ^{13}C {^{1}H} NMR spectrum of compound **1h** (101 MHz, $CDCl_3$)

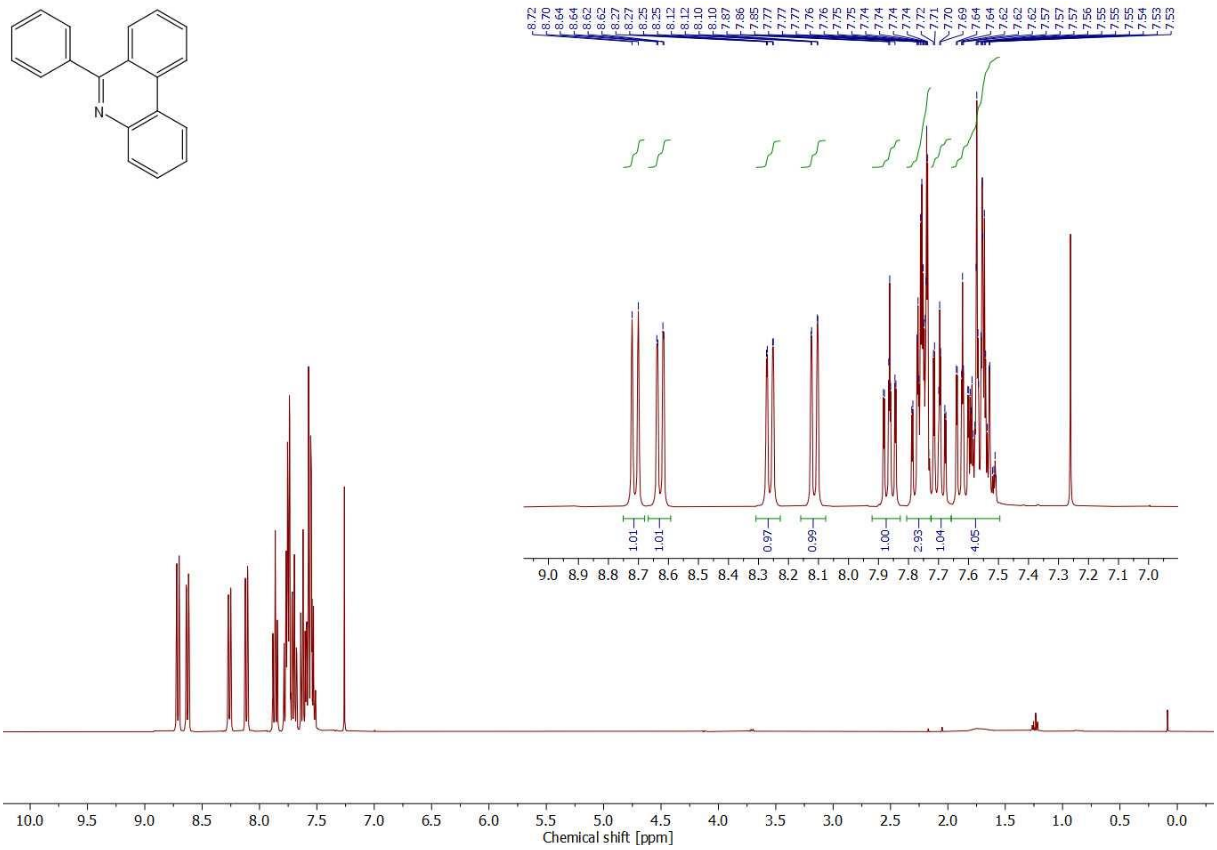

Figure S37. ^{1}H NMR spectrum of compound **2** (400 MHz, $CDCl_3$)

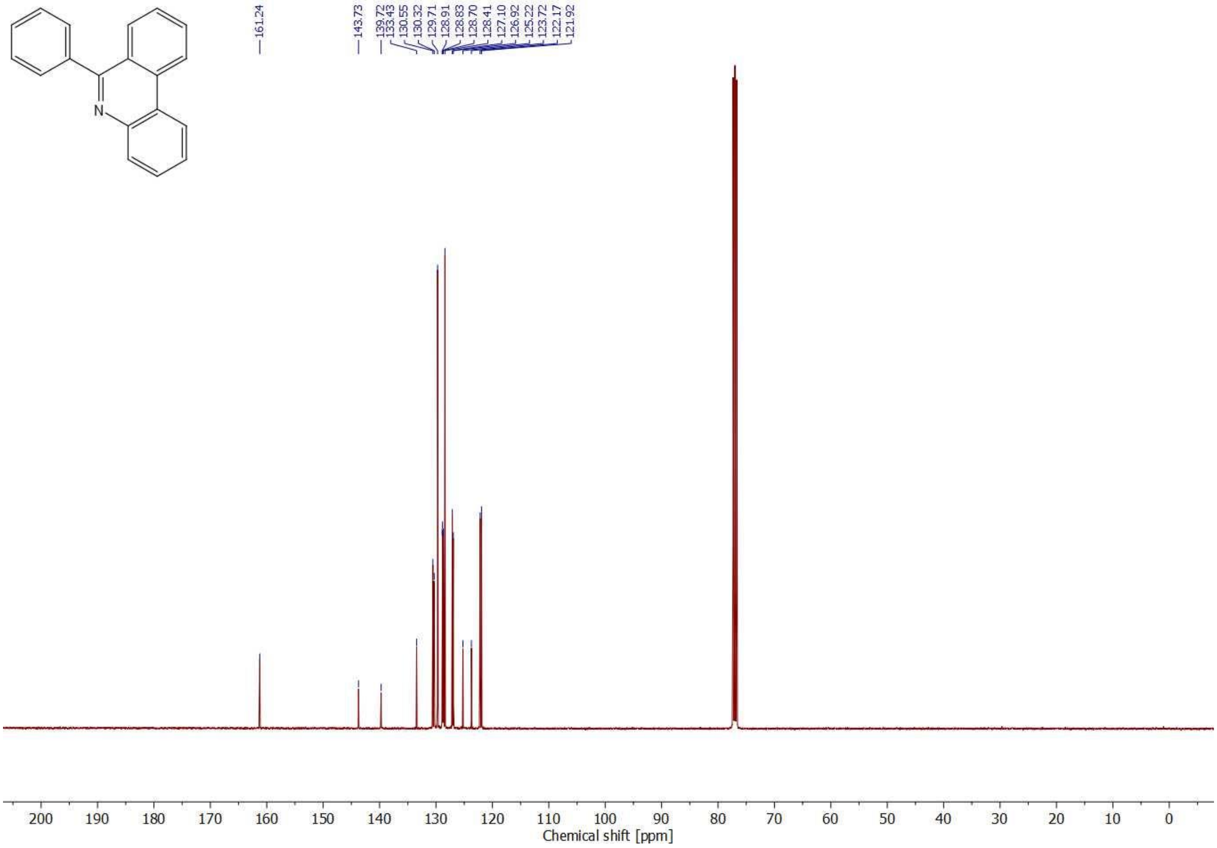

Figure S38. ^{13}C {^{1}H} NMR spectrum of compound **2** (101 MHz, $CDCl_3$)

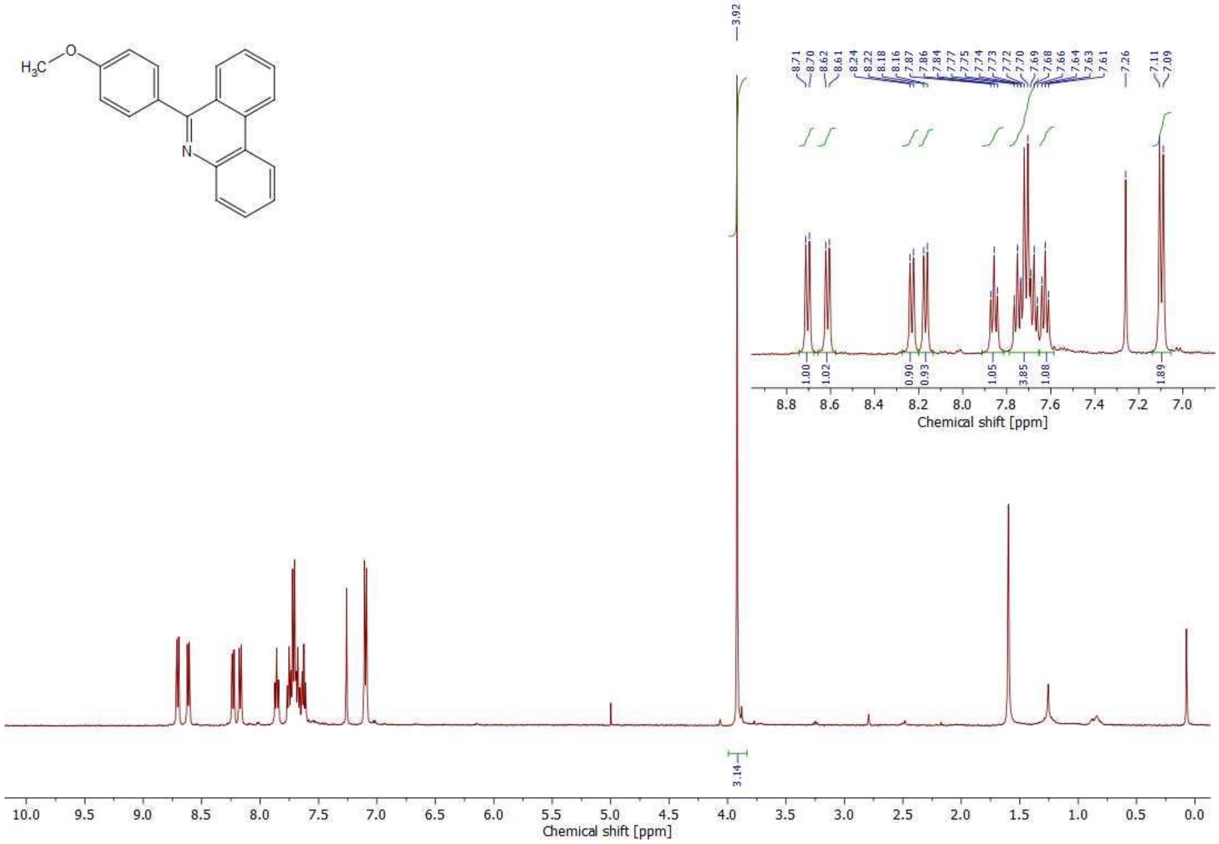

Figure S39. ^{1}H NMR spectrum of compound **2a** (500 MHz, $CDCl_3$)

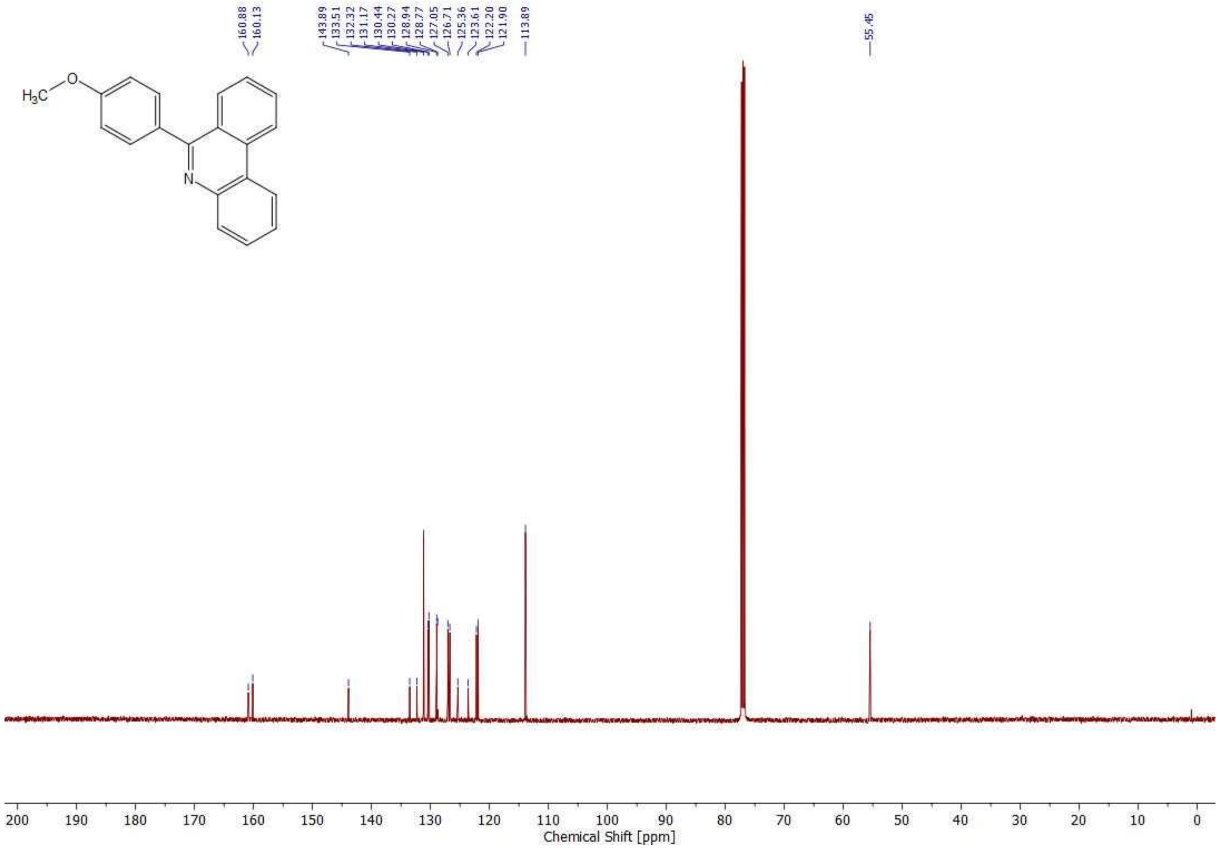

Figure S40. ^{13}C {^{1}H} NMR spectrum of compound **2a** (126 MHz, $CDCl_3$)

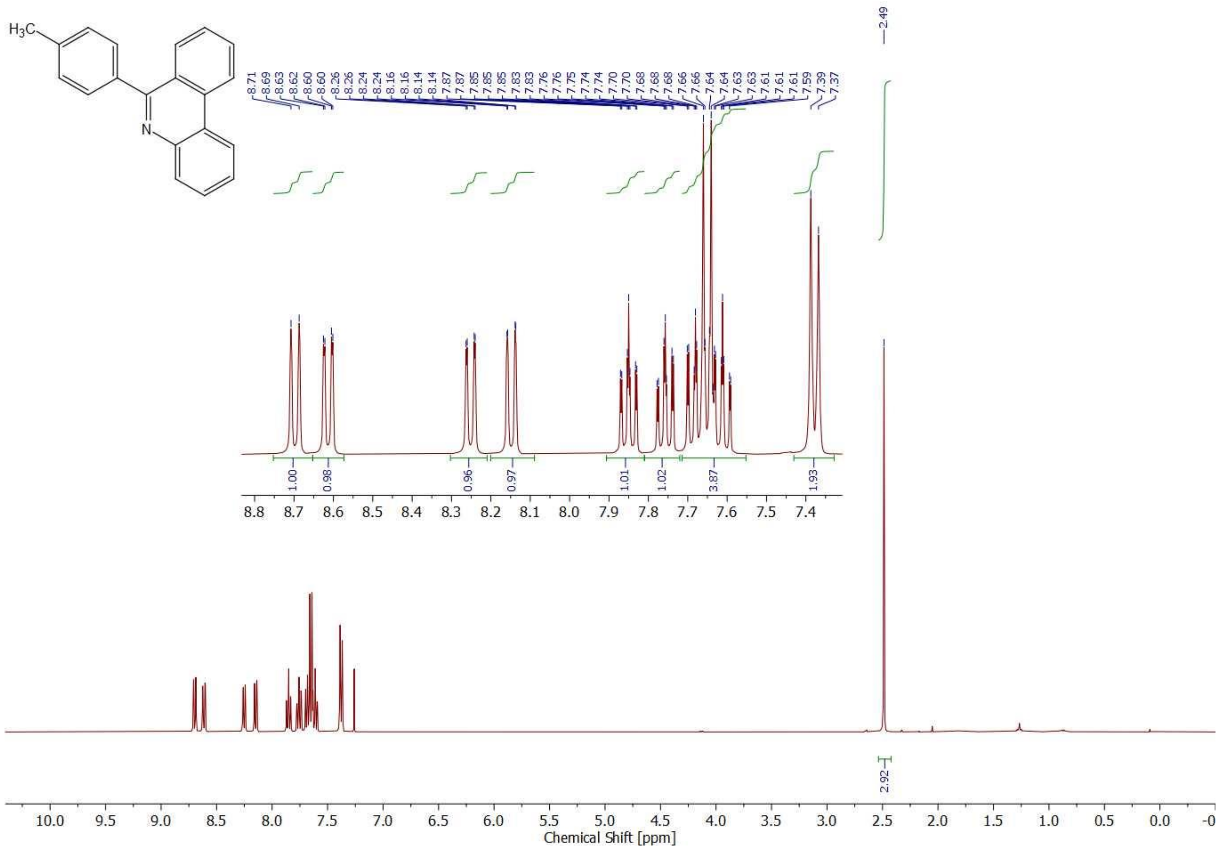

Figure S41. ^{1}H NMR spectrum of compound **2b** (400 MHz, $CDCl_3$)

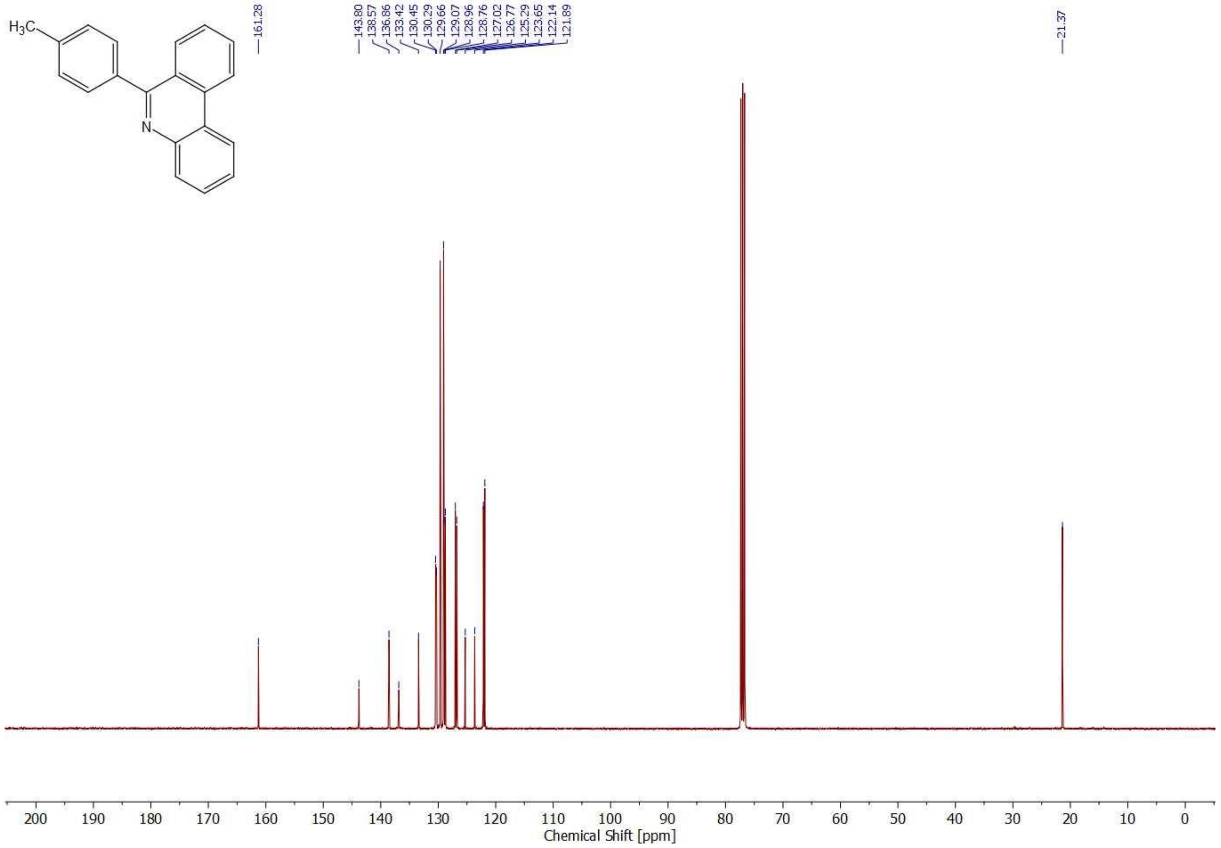

Figure S42. ^{13}C {^{1}H} NMR spectrum of compound **2b** (101 MHz, $CDCl_3$)

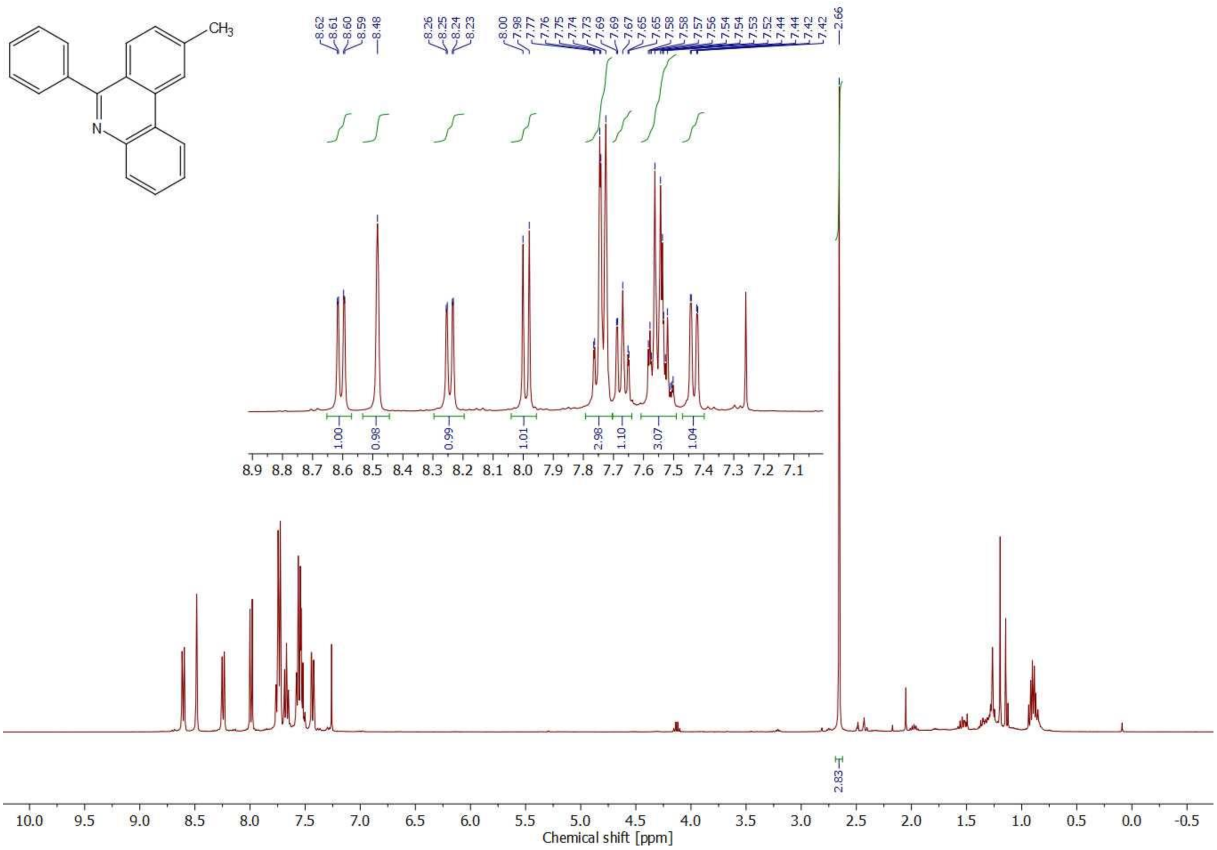

Figure S43. ^{1}H NMR spectrum of compound **3b** (400 MHz, $CDCl_3$)

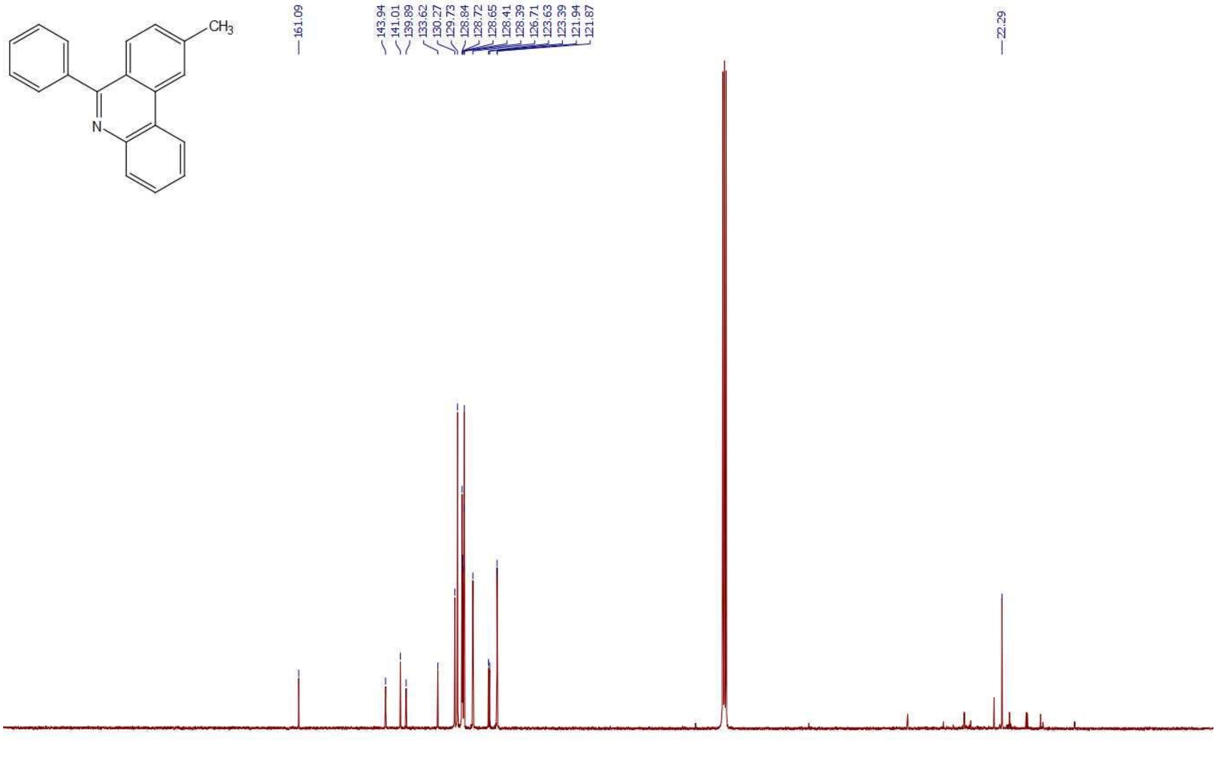

Figure S44. ^{13}C NMR spectrum of compound **3b** (101 MHz, $CDCl_3$)

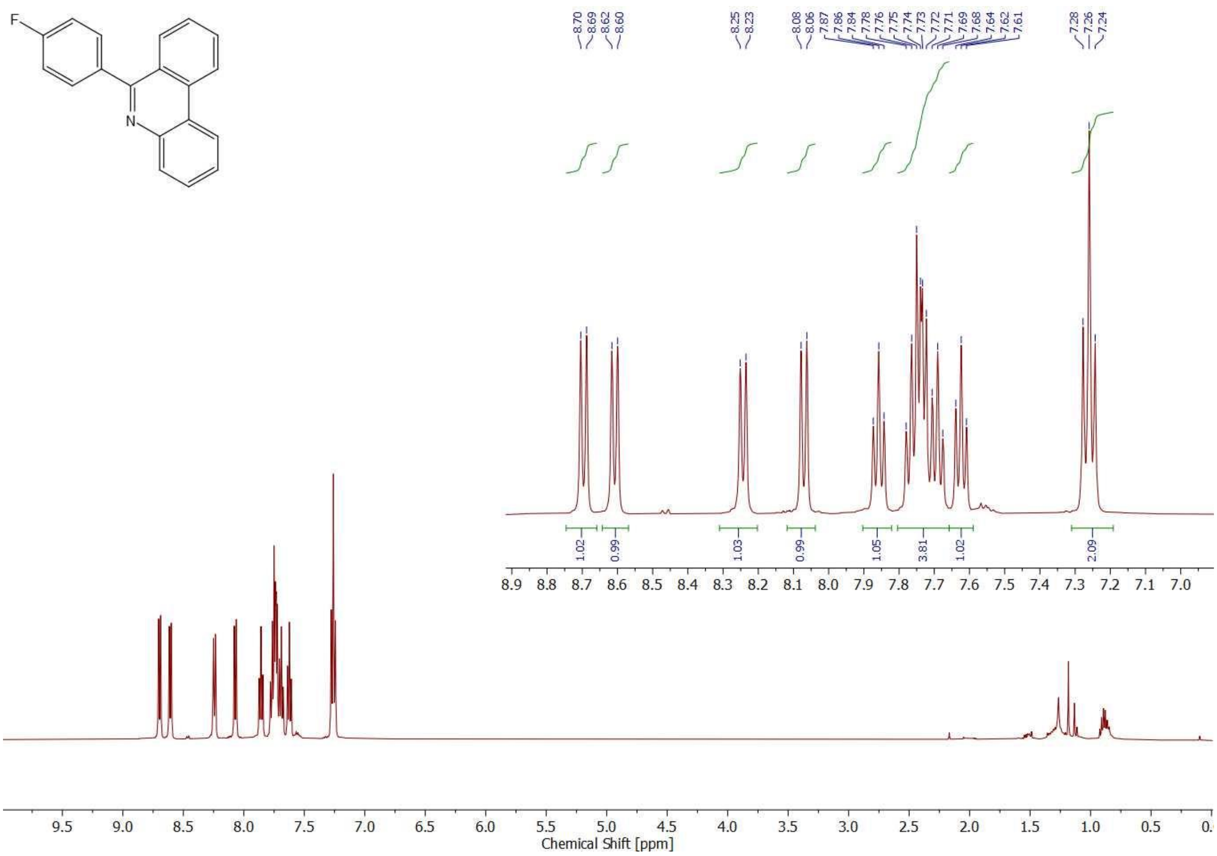

Figure S45. ^{1}H NMR spectrum of compound **2c** (500 MHz, $CDCl_3$)

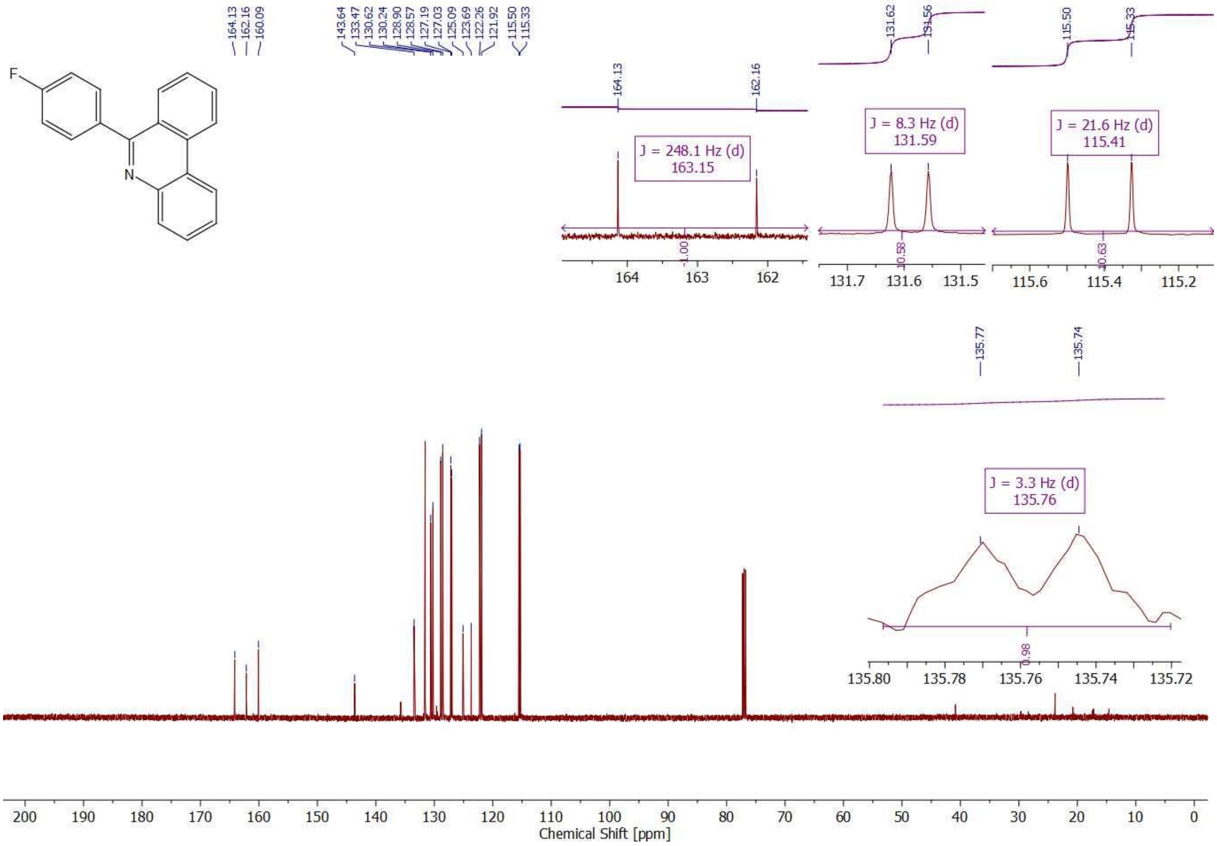

Figure S46. ^{13}C {^{1}H} NMR spectrum of compound **2c** (126 MHz, $CDCl_3$)

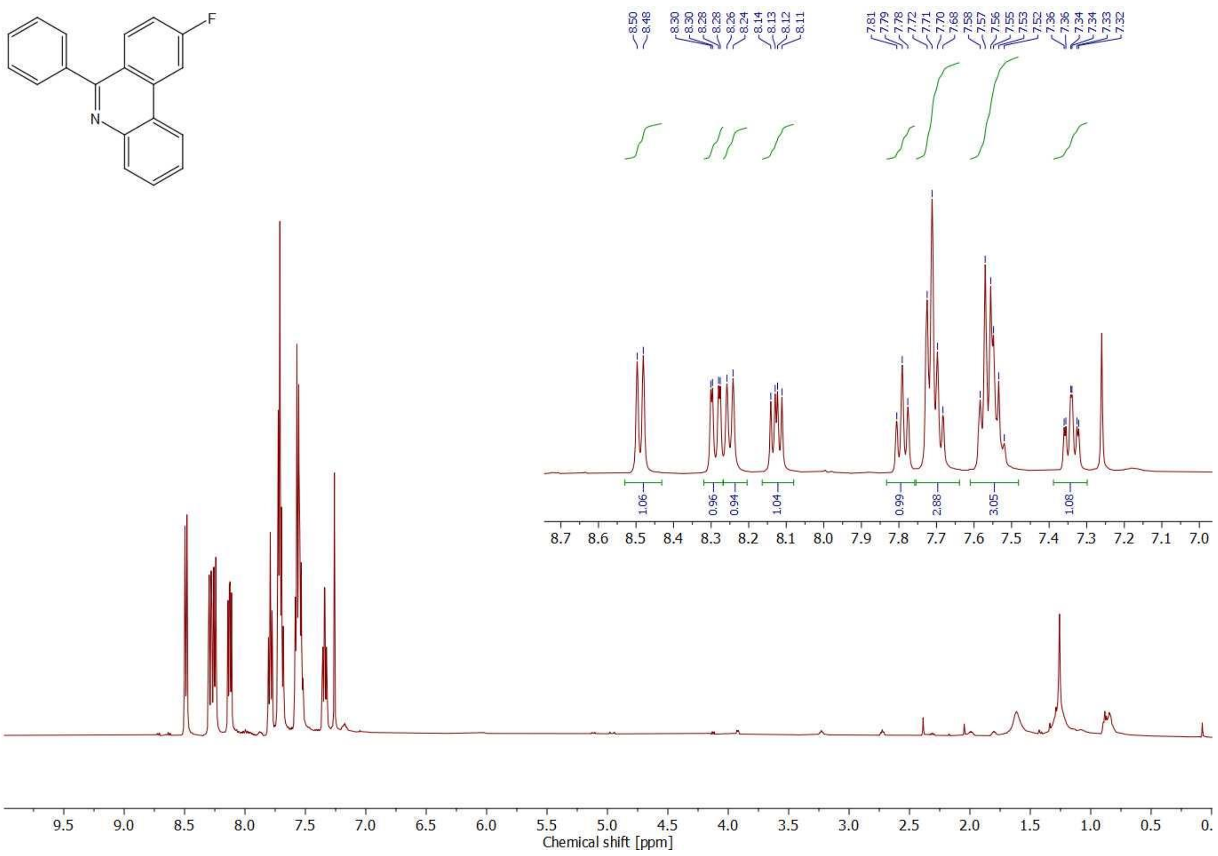

Figure S47. ^{1}H NMR spectrum of compound **3c** (500 MHz, $CDCl_3$)

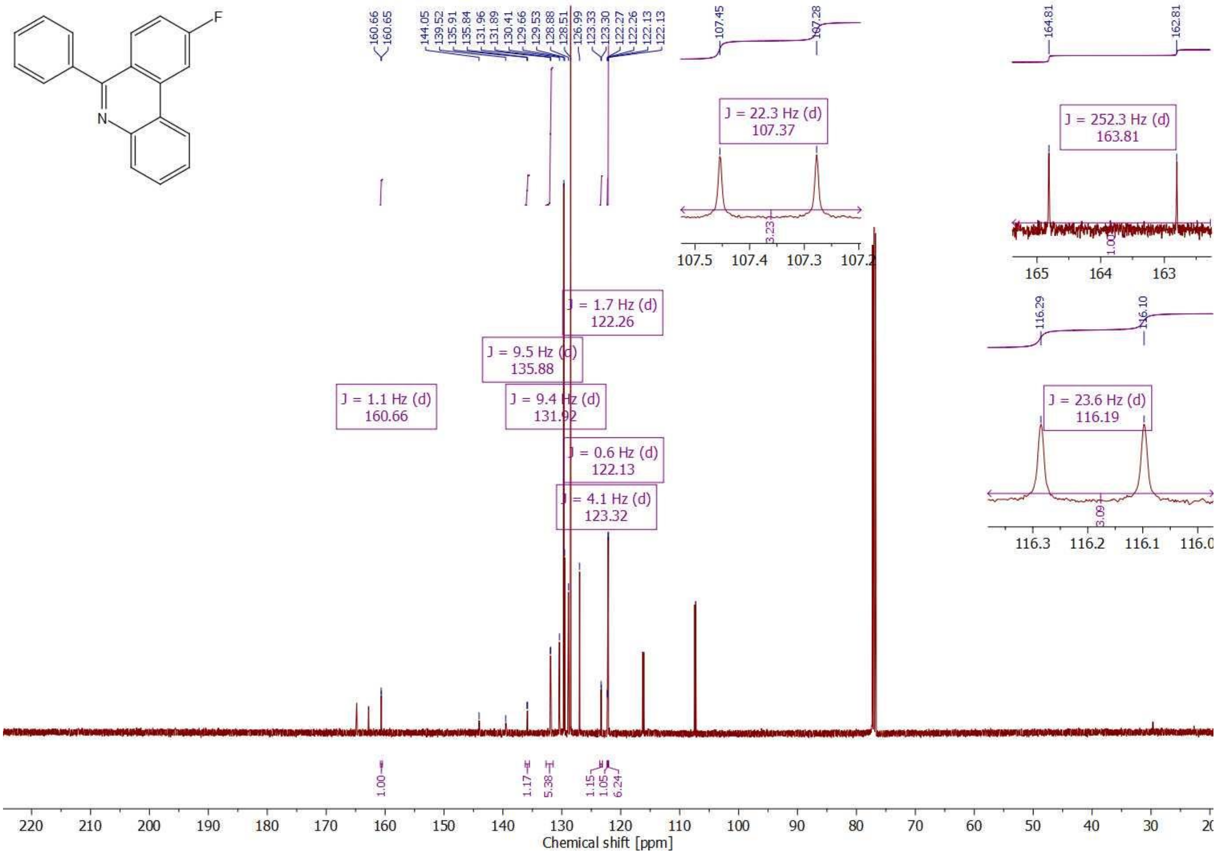

Figure S48. ^{13}C {^{1}H} NMR spectrum of compound **3c** (126 MHz, $CDCl_3$)

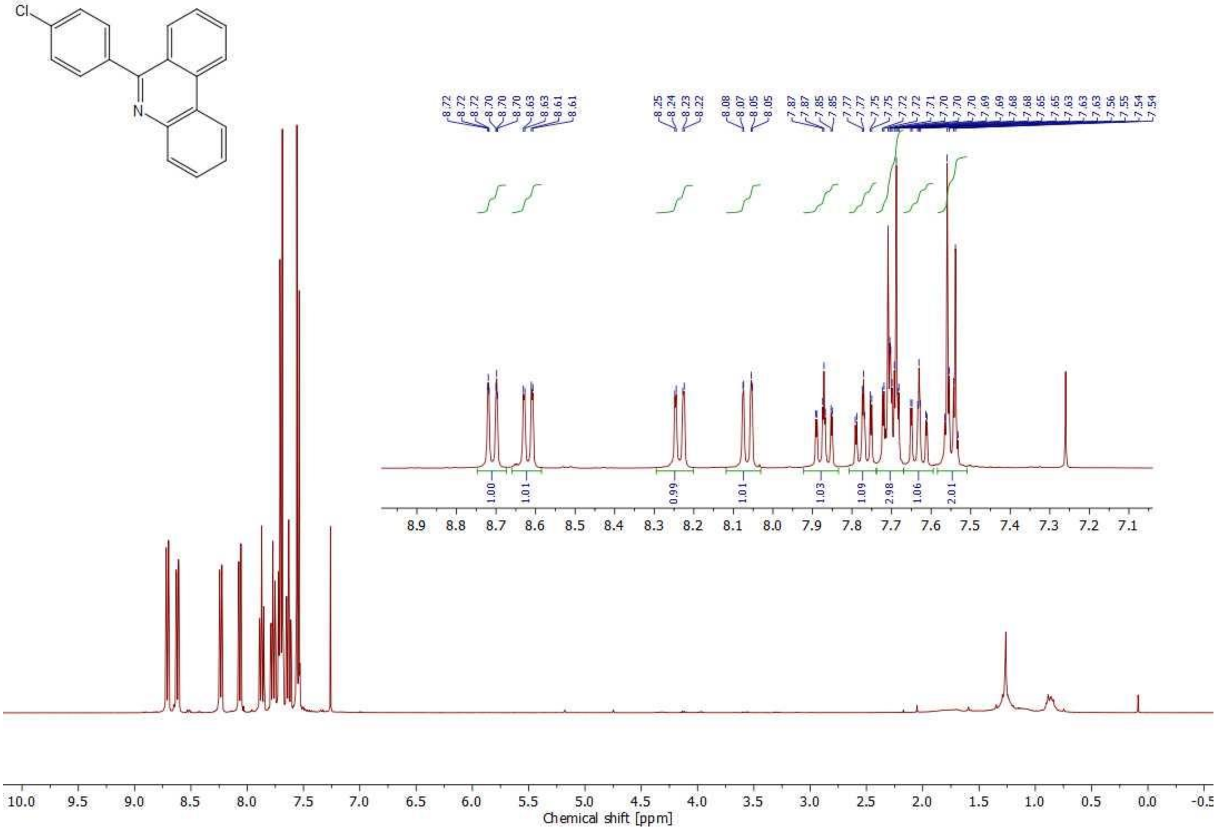

Figure S49. ^{1}H NMR spectrum of compound **2d** (400 MHz, $CDCl_3$)

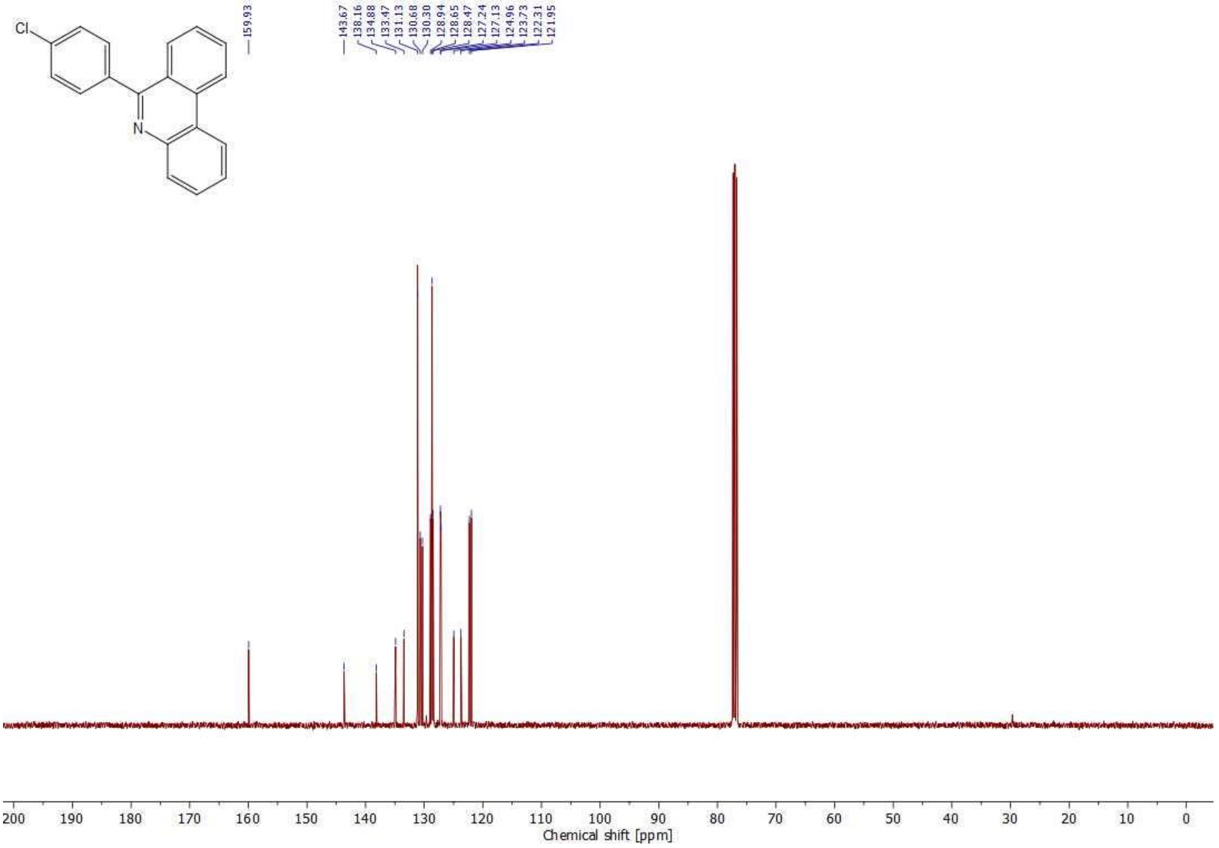

Figure S50. ^{13}C {^{1}H} NMR spectrum of compound **2d** (101 MHz, $CDCl_3$)

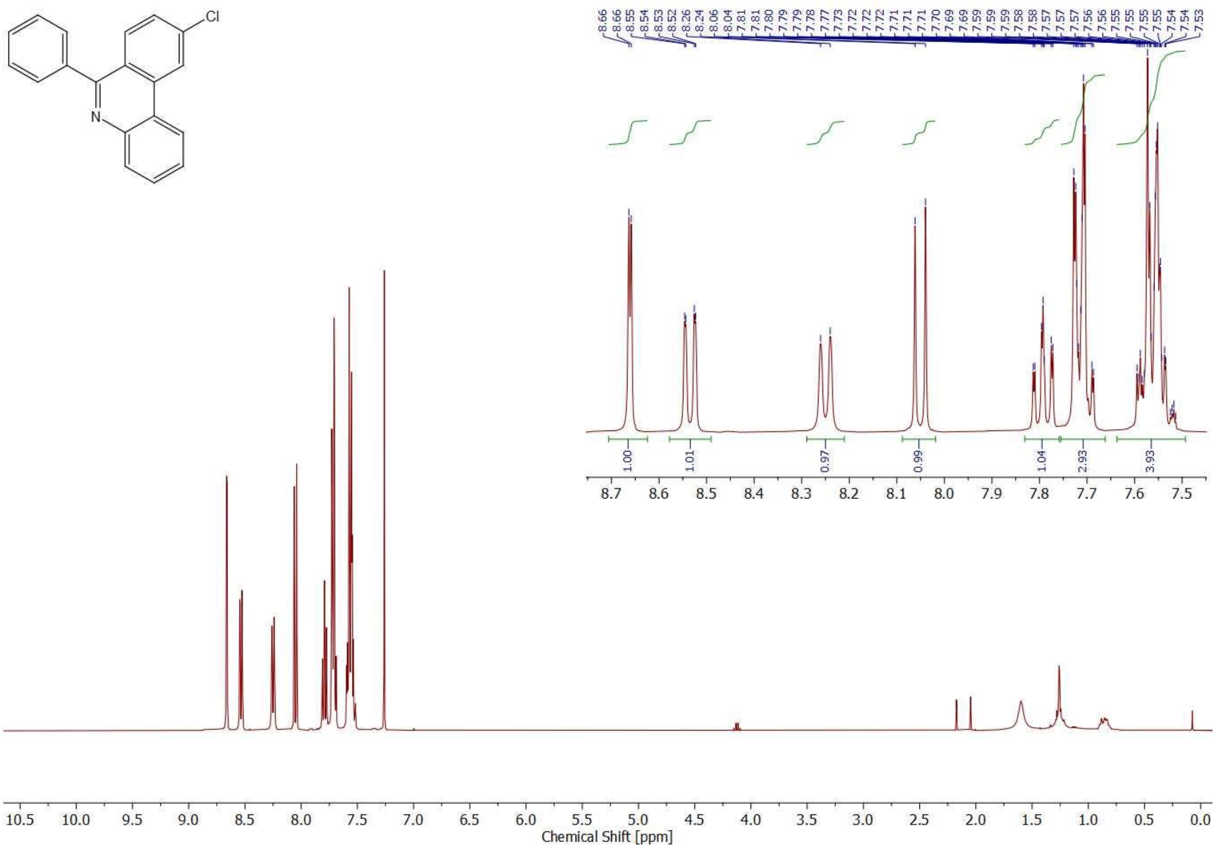

Figure S51. ^{1}H NMR spectrum of compound **3d** (400 MHz, $CDCl_3$)

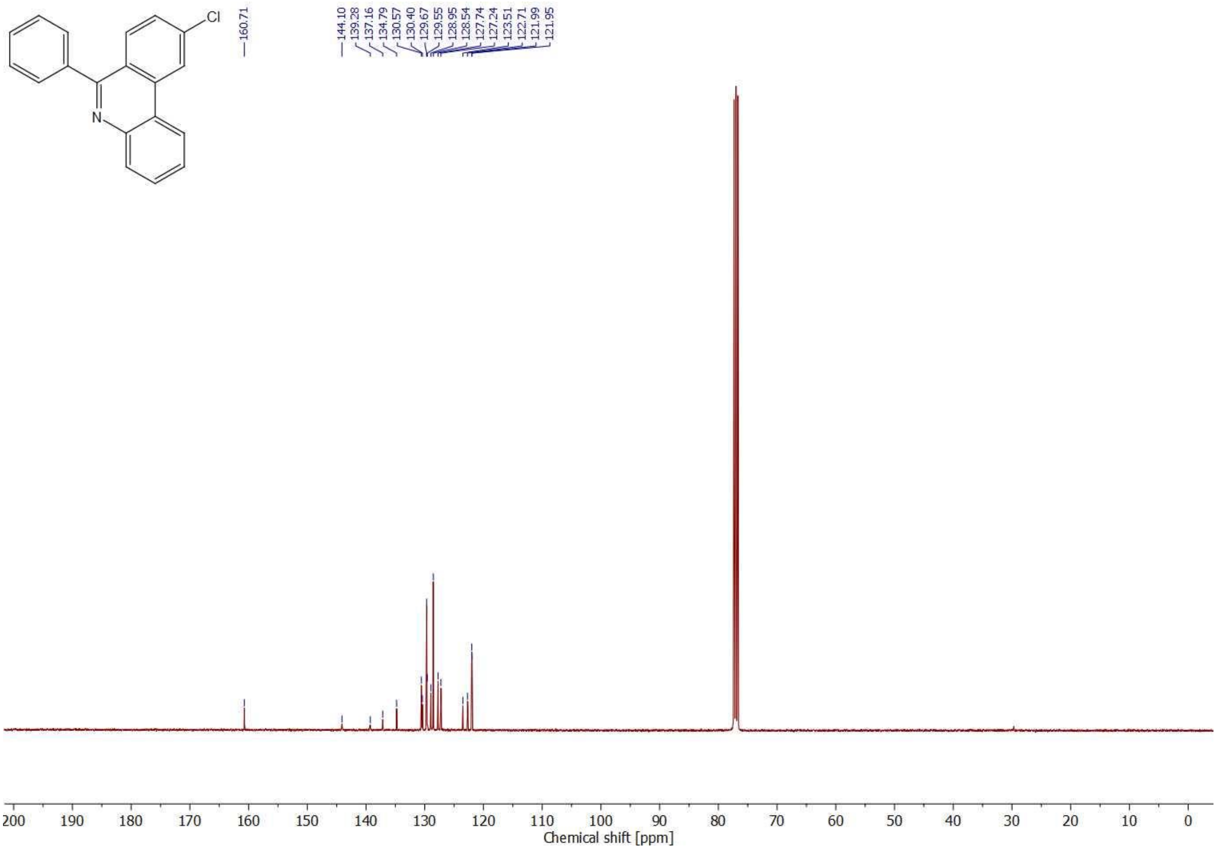

Figure S52. ^{13}C {^{1}H} NMR spectrum of compound **3d** (101 MHz, $CDCl_3$)

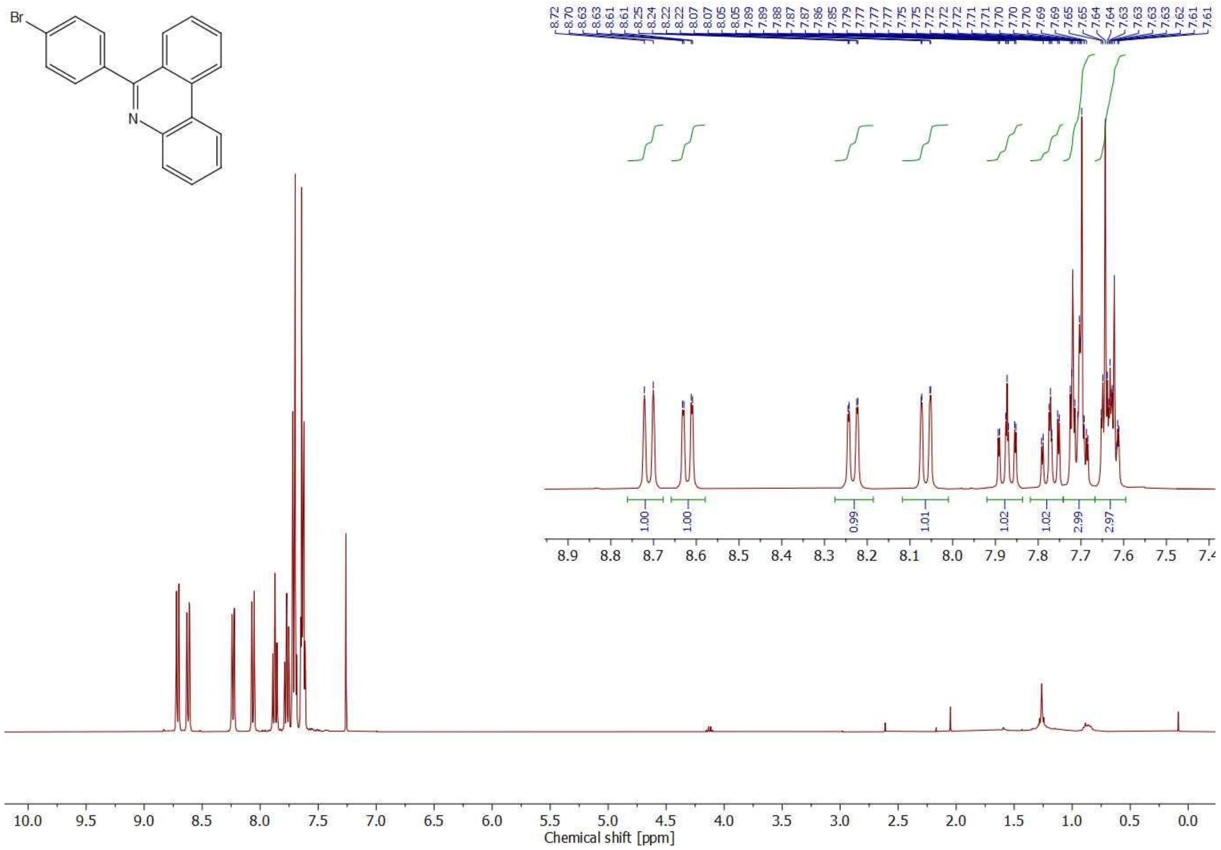

Figure S53. ^{1}H NMR spectrum of compound **2e** (400 MHz, $CDCl_3$)

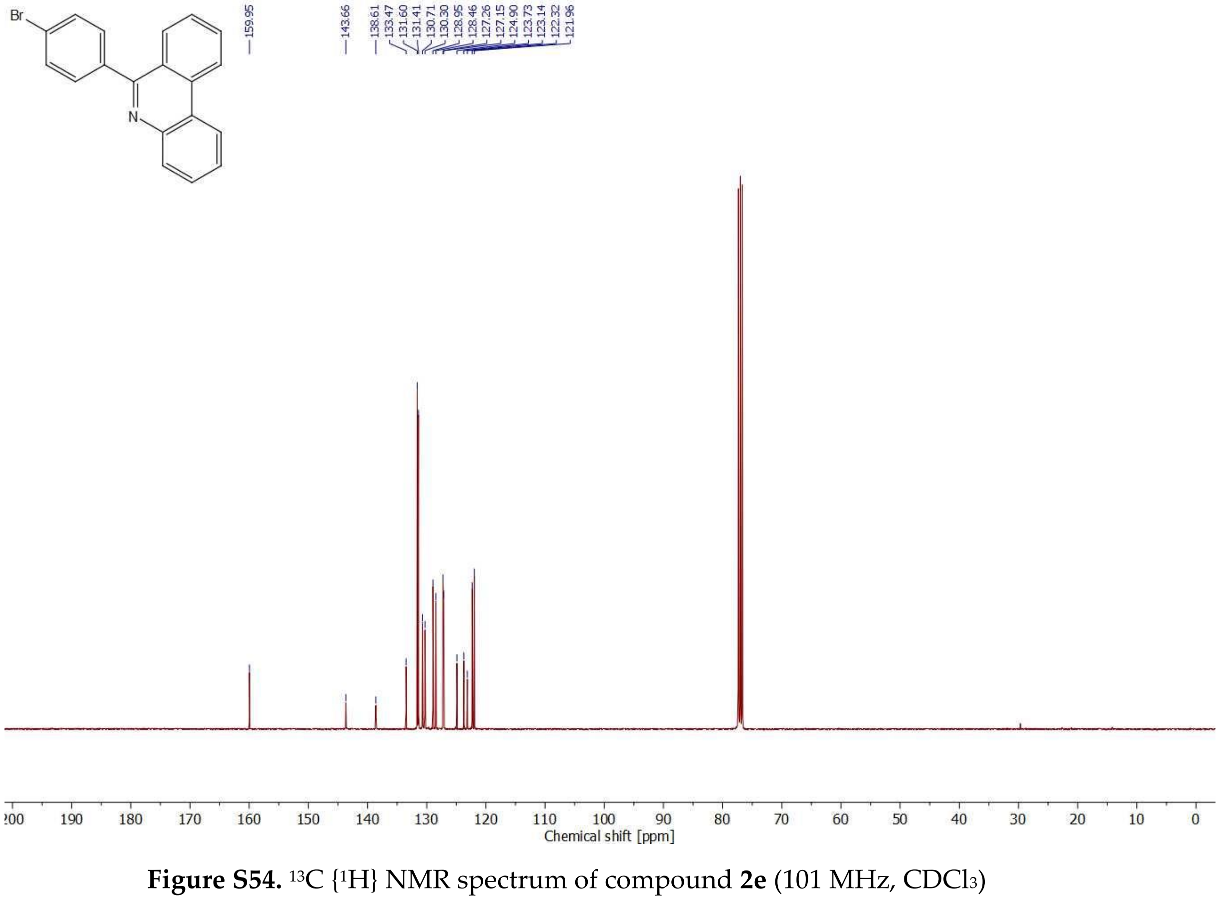

Figure S54. ^{13}C {^{1}H} NMR spectrum of compound **2e** (101 MHz, $CDCl_3$)

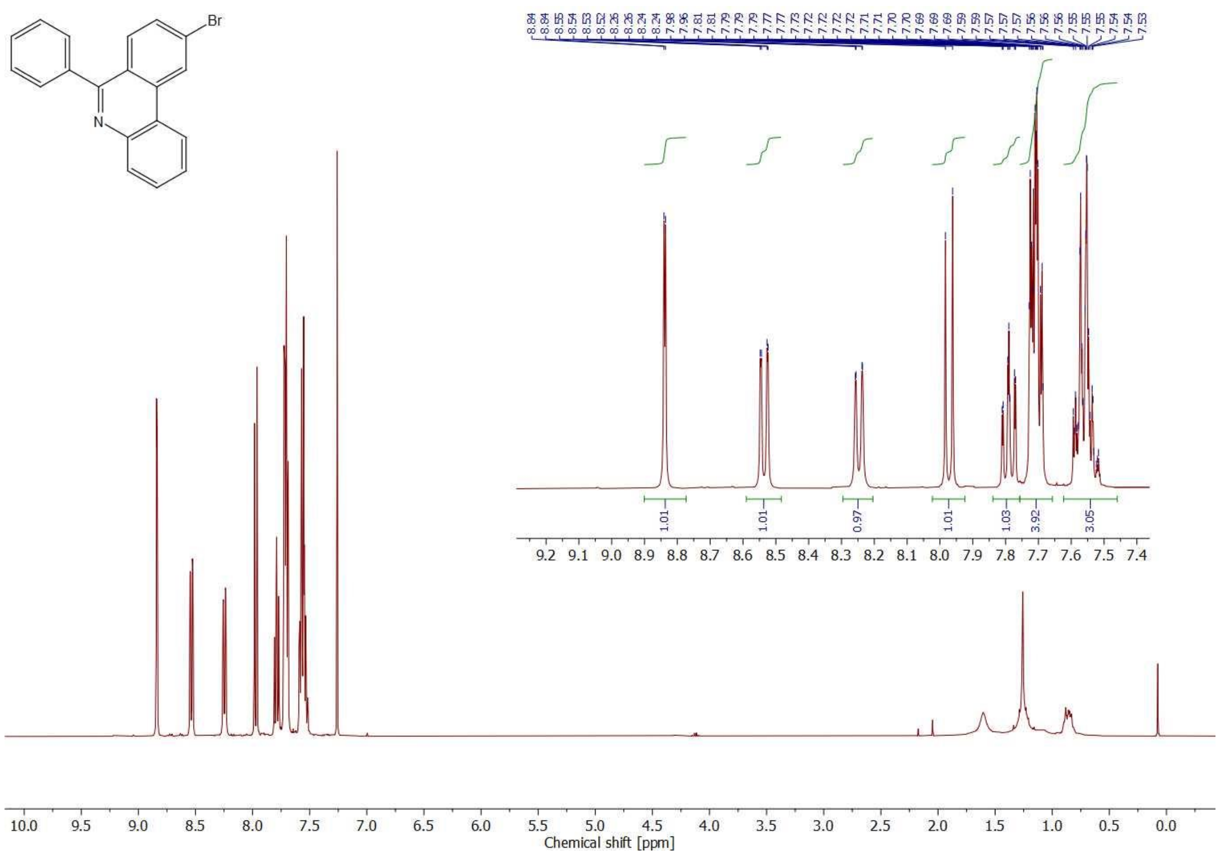

Figure S55. ^{1}H NMR spectrum of compound **3e** (400 MHz, $CDCl_3$)

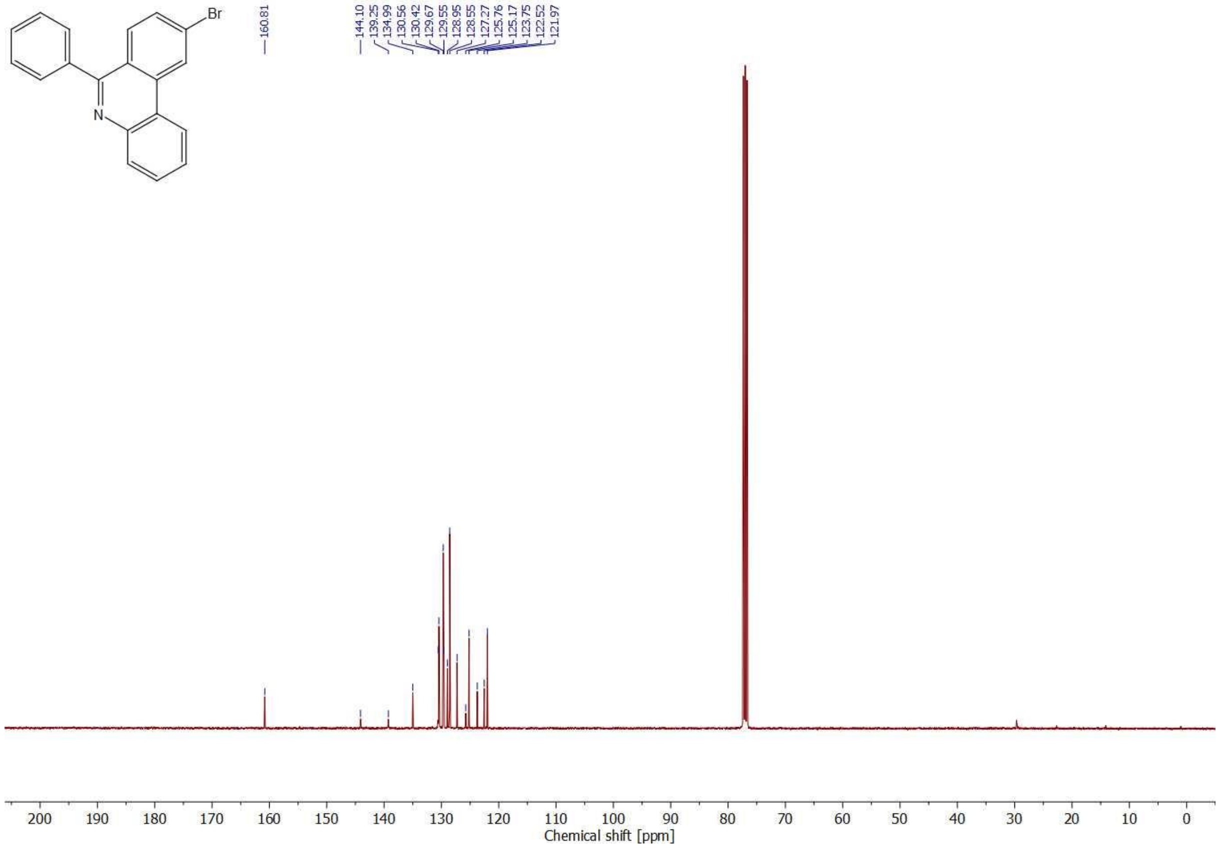

Figure S56. ^{13}C {^{1}H} NMR spectrum of compound **3e** (101 MHz, $CDCl_3$)

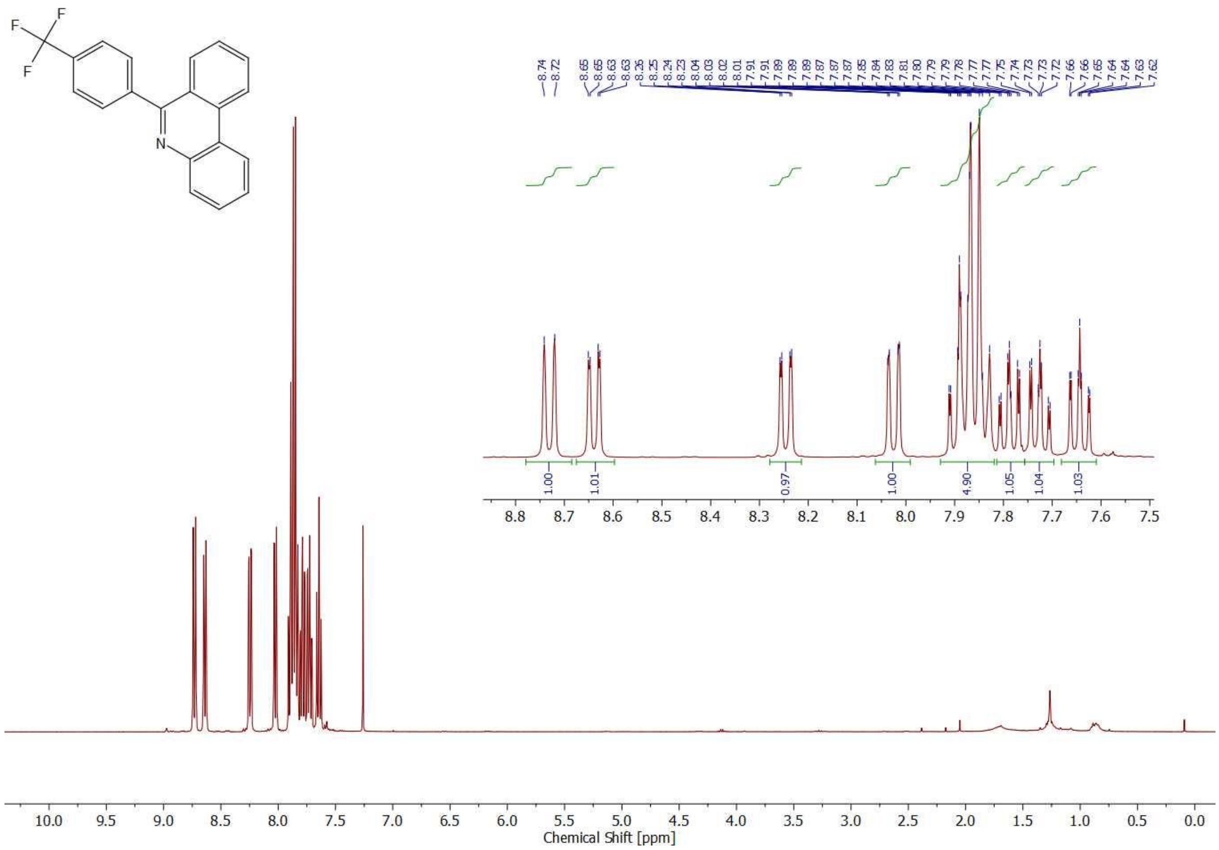

Figure S57. ^{1}H NMR spectrum of compound **2f** (400 MHz, $CDCl_3$)

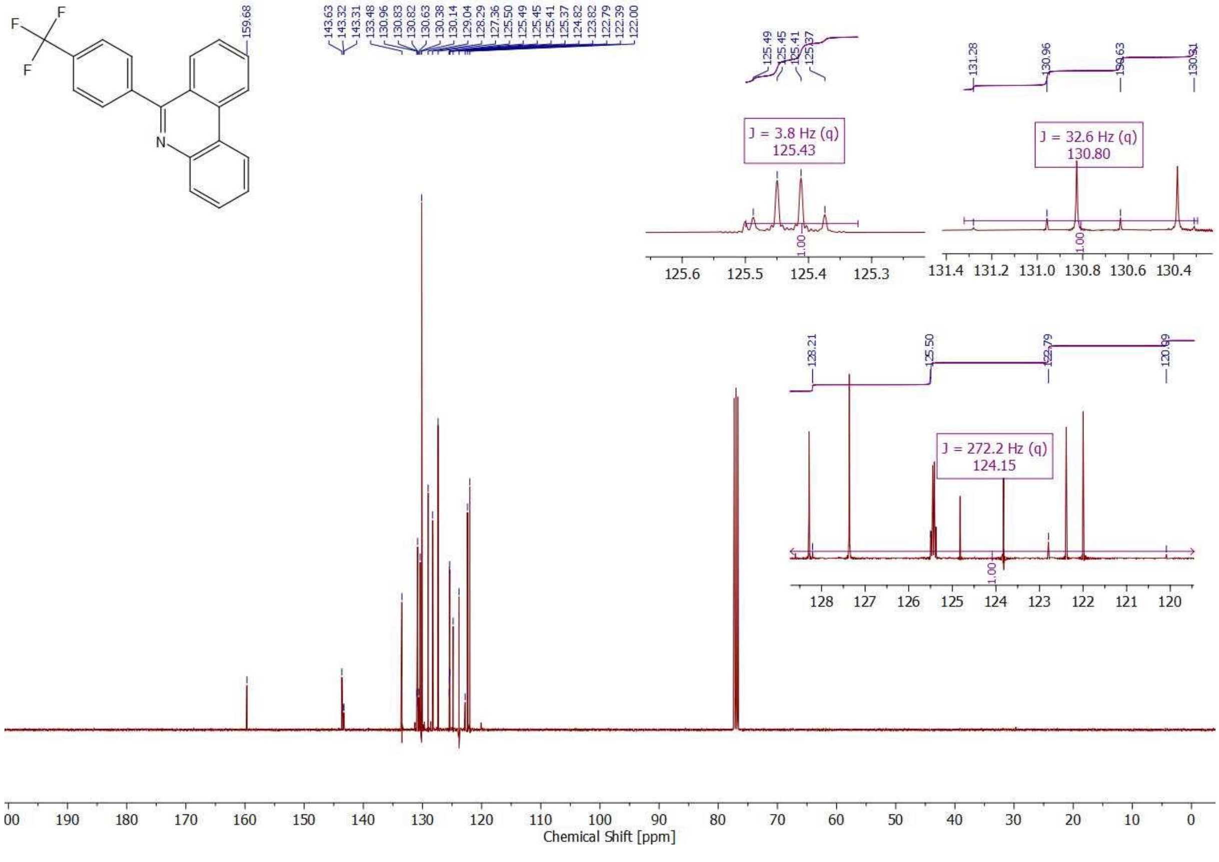

Figure S58. ^{13}C {^{1}H} NMR spectrum of compound **2f** (101 MHz, $CDCl_3$)

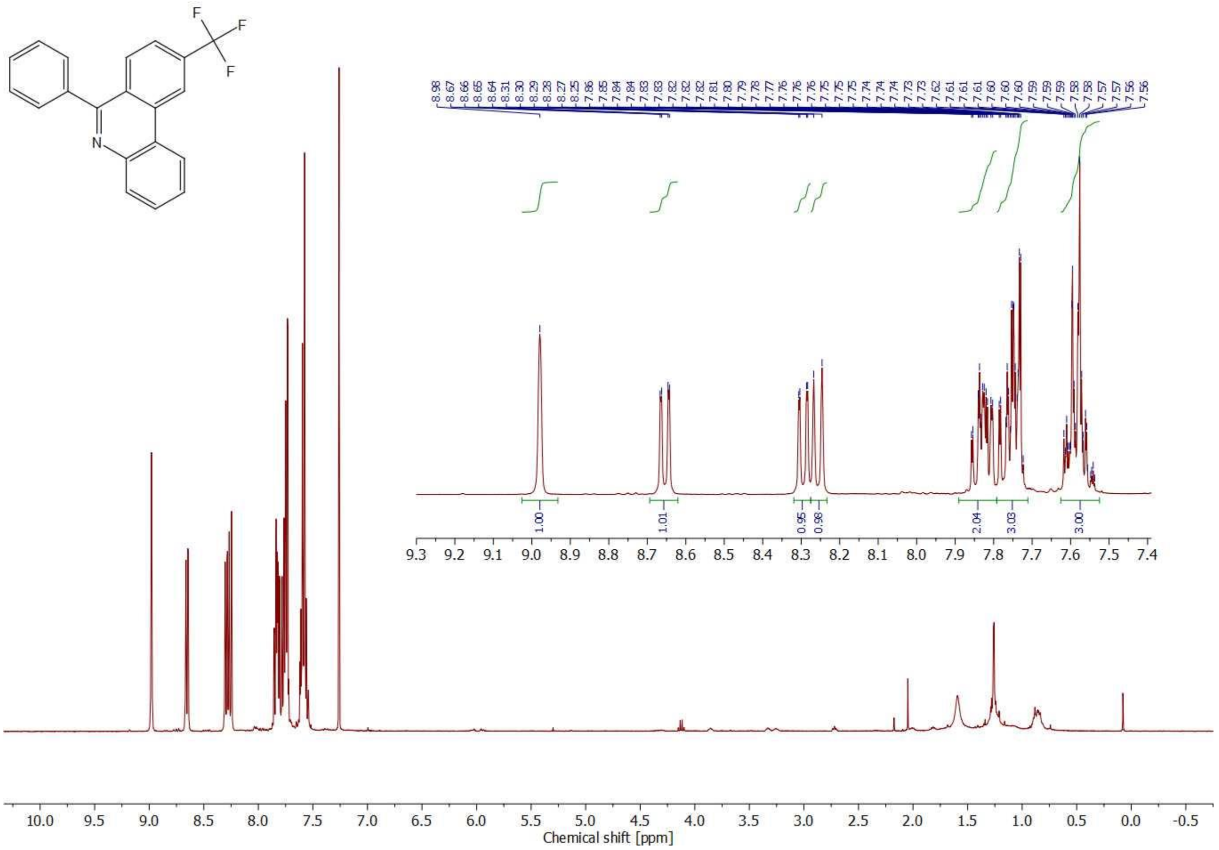

Figure S59. ^{1}H NMR spectrum of compound **3f** (400 MHz, $CDCl_3$)

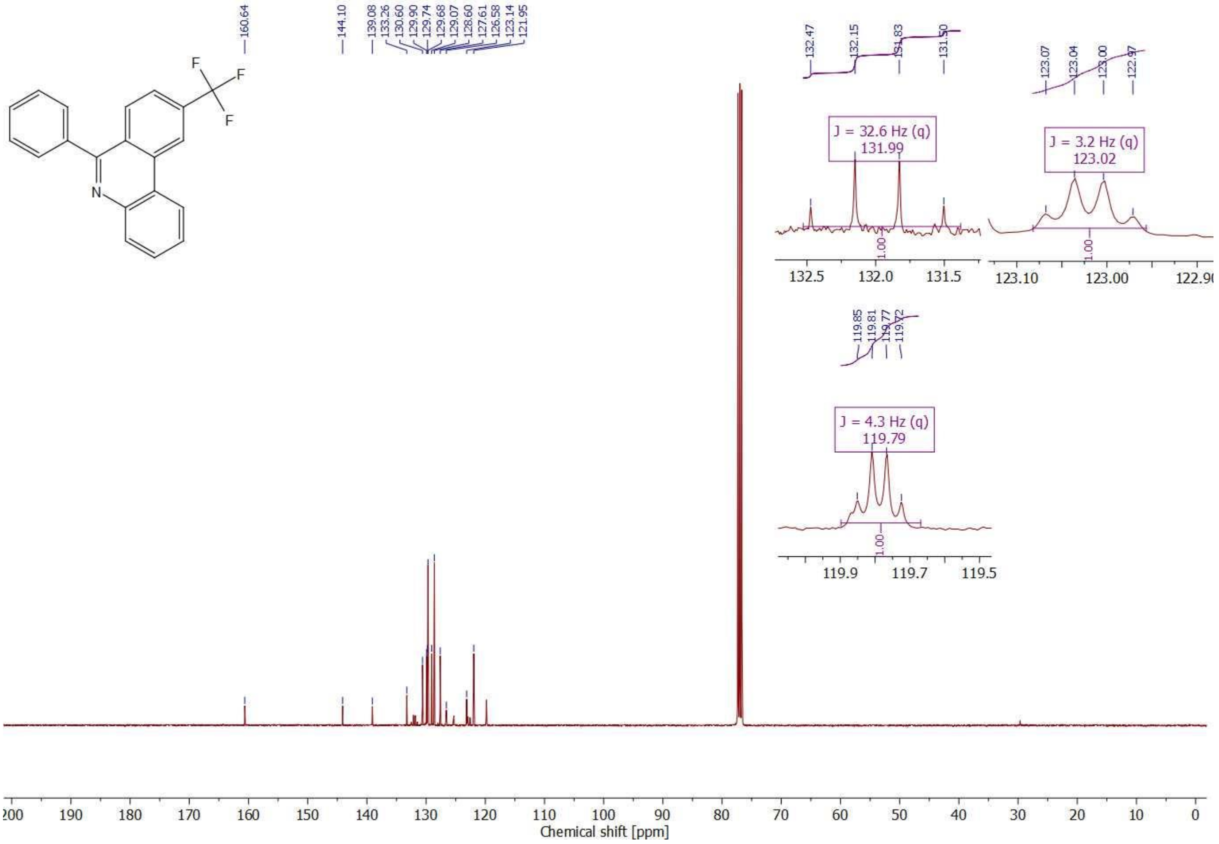

Figure S60. ^{13}C {^{1}H} NMR spectrum of compound **3f** (101 MHz, $CDCl_3$)

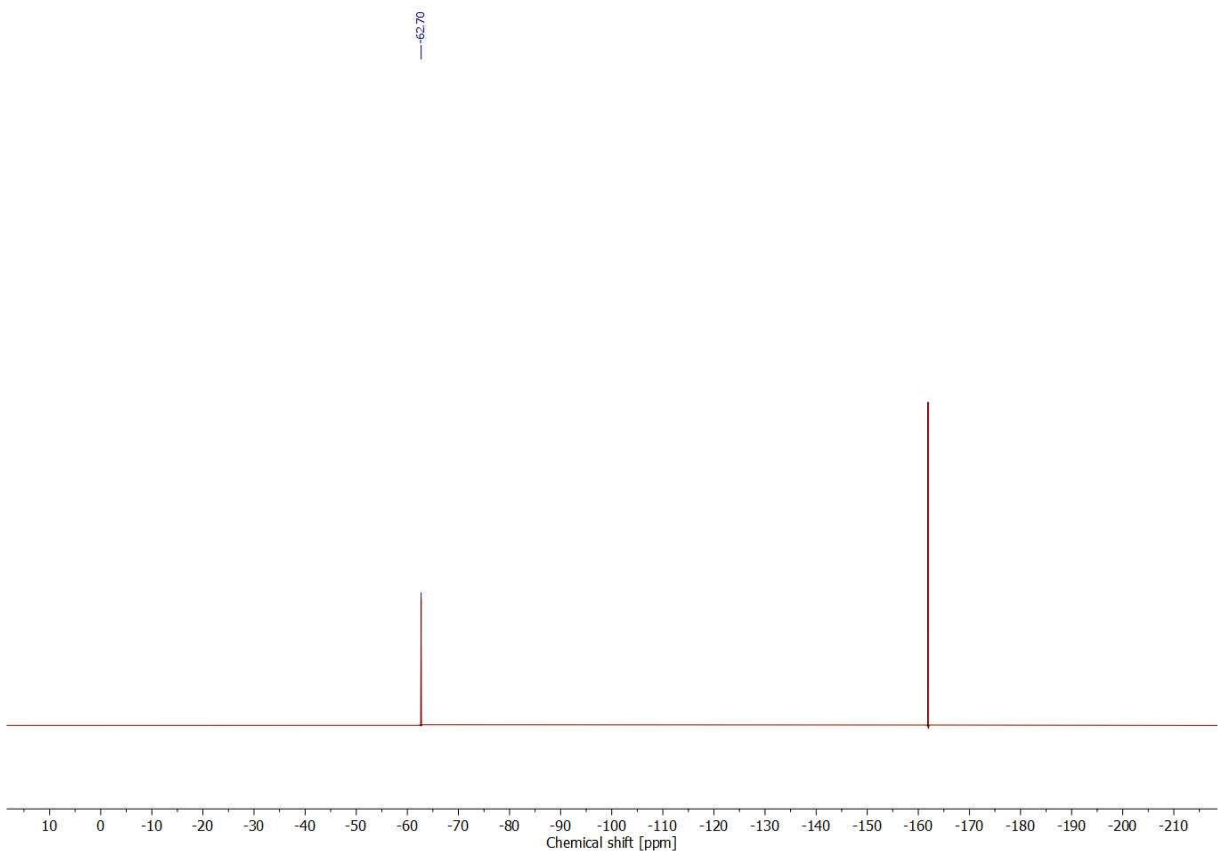

Figure S61. ^{19}F {^1H} NMR spectrum of compound **3f** (376 MHz, $CDCl_3$, C_6F_6)

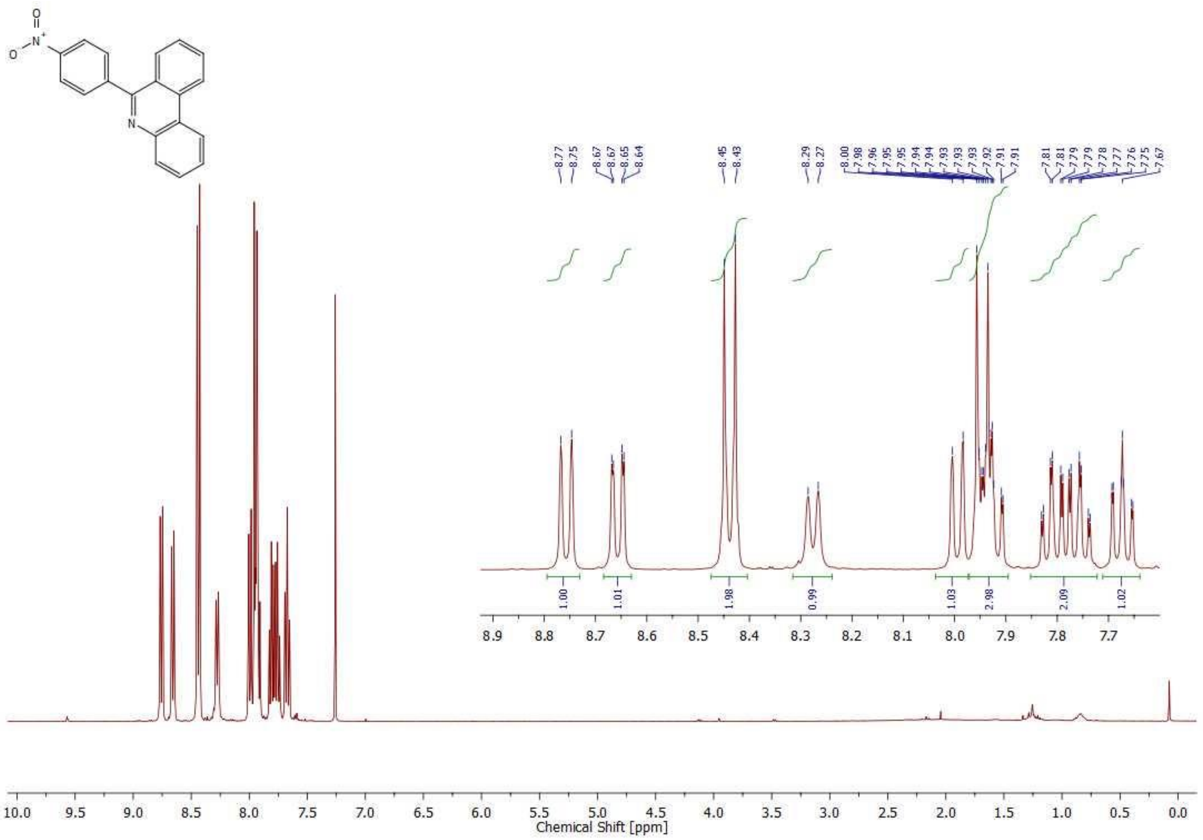

Figure S62. ^{1}H NMR spectrum of compound **2g** (400 MHz, $CDCl_3$)

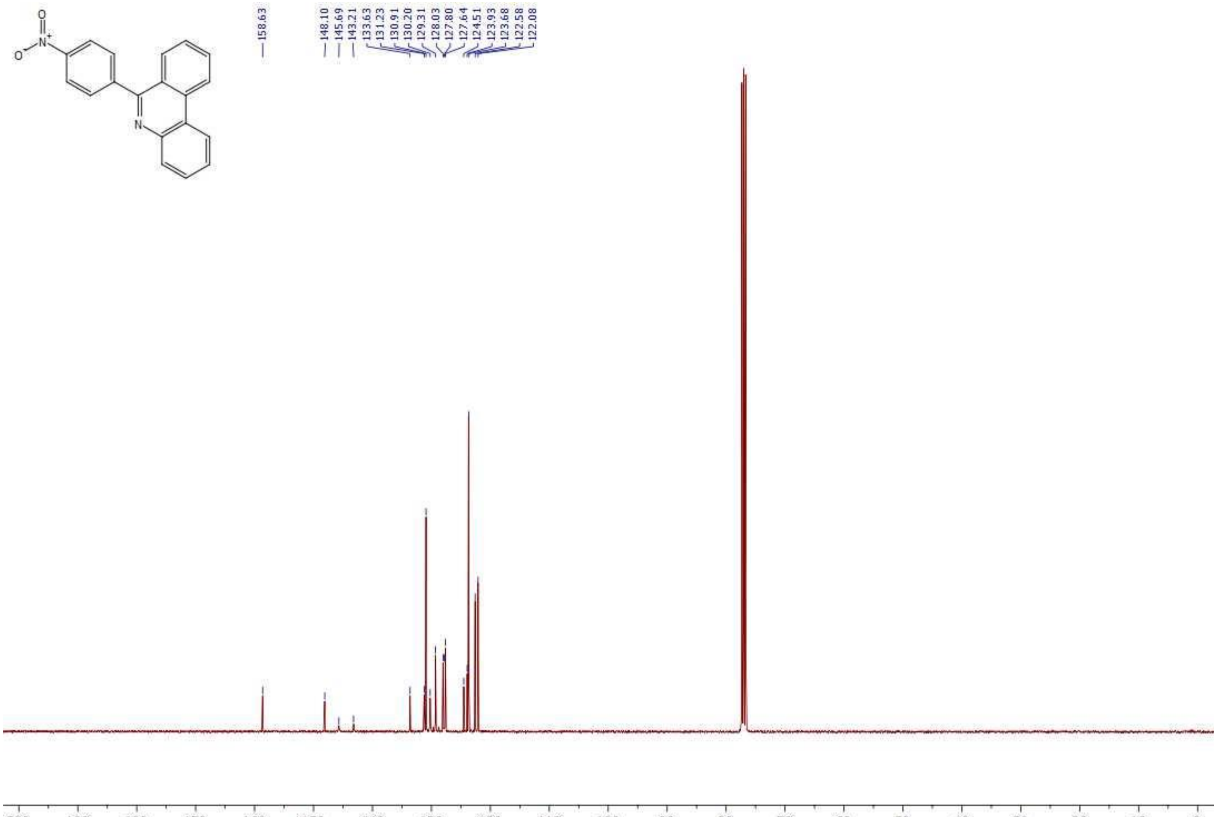

Figure S63. ^{13}C {^{1}H} NMR spectrum of compound **2g** (101 MHz, $CDCl_3$)

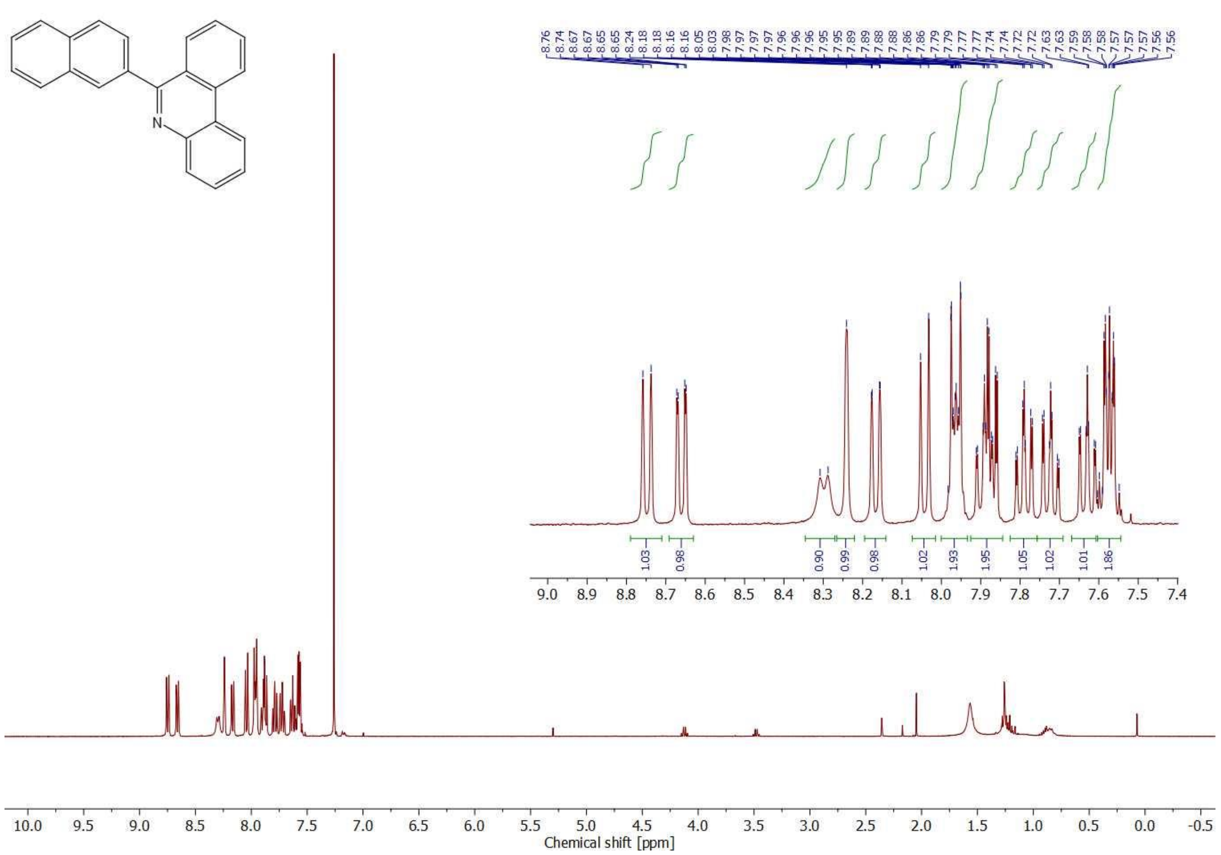

Figure S64. ^{1}H NMR spectrum of compound **2h** (400 MHz, $CDCl_3$)

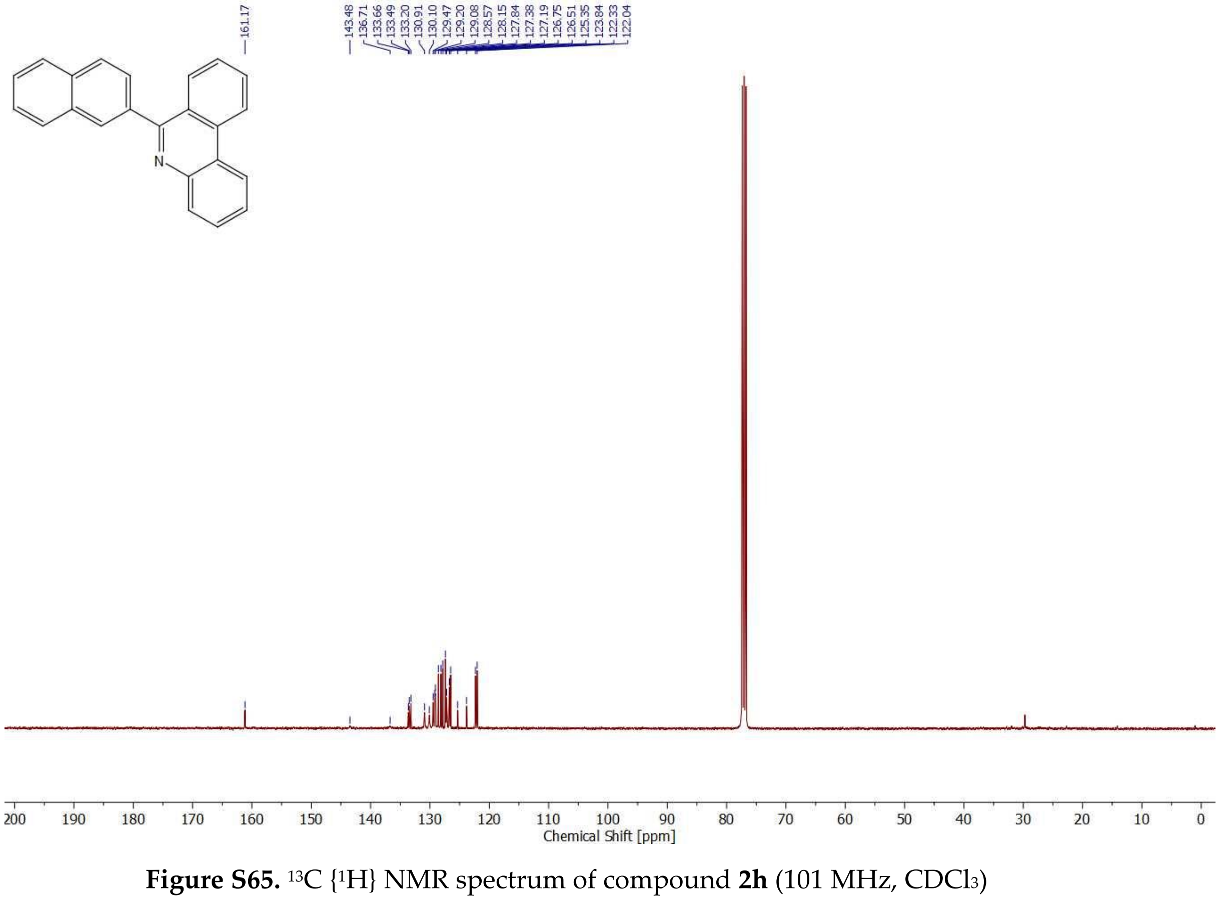

Figure S65. ^{13}C {^{1}H} NMR spectrum of compound **2h** (101 MHz, $CDCl_3$)

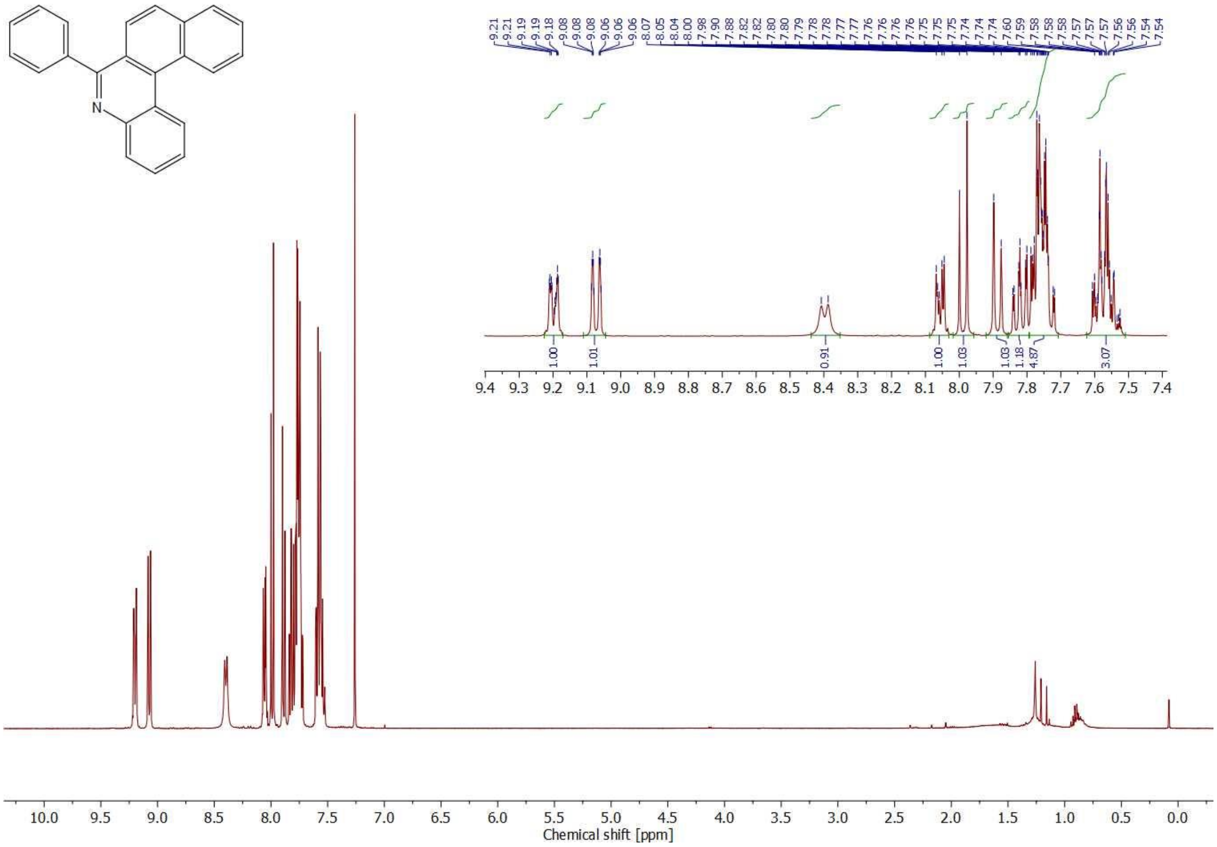

Figure S66. ^{1}H NMR spectrum of compound **3h** (400 MHz, $CDCl_3$)

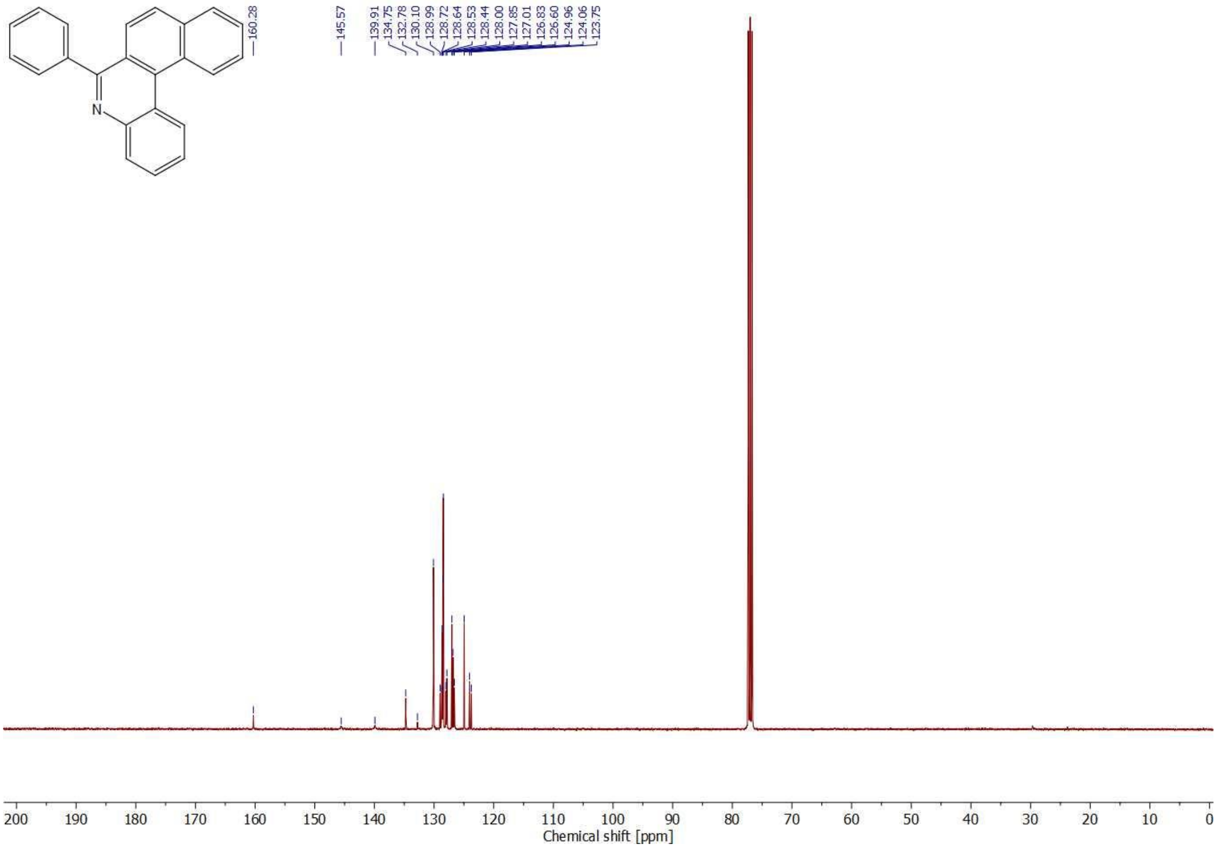

Figure S67. ^{13}C {^{1}H} NMR spectrum of compound **3h** (101 MHz, $CDCl_3$)

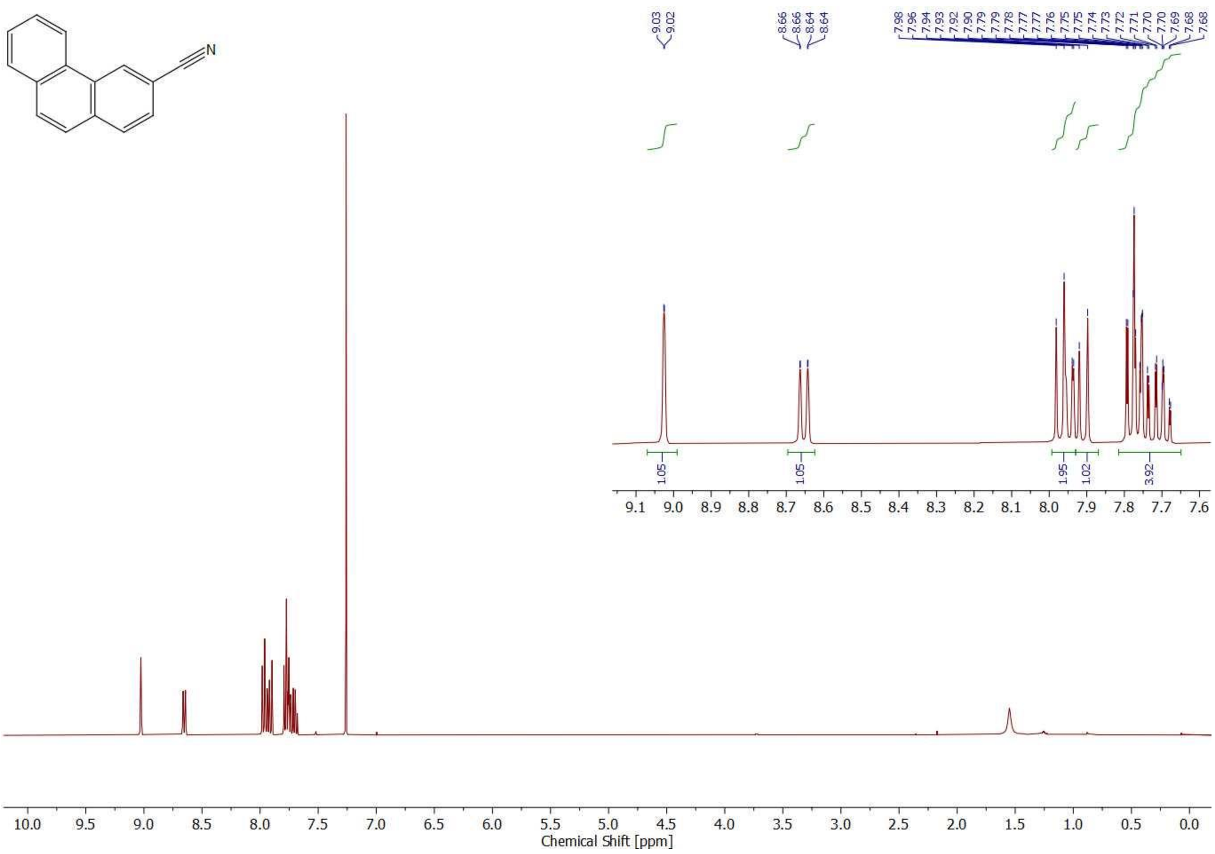

Figure S68. ^{1}H NMR spectrum of compound **4j** (400 MHz, $CDCl_3$)

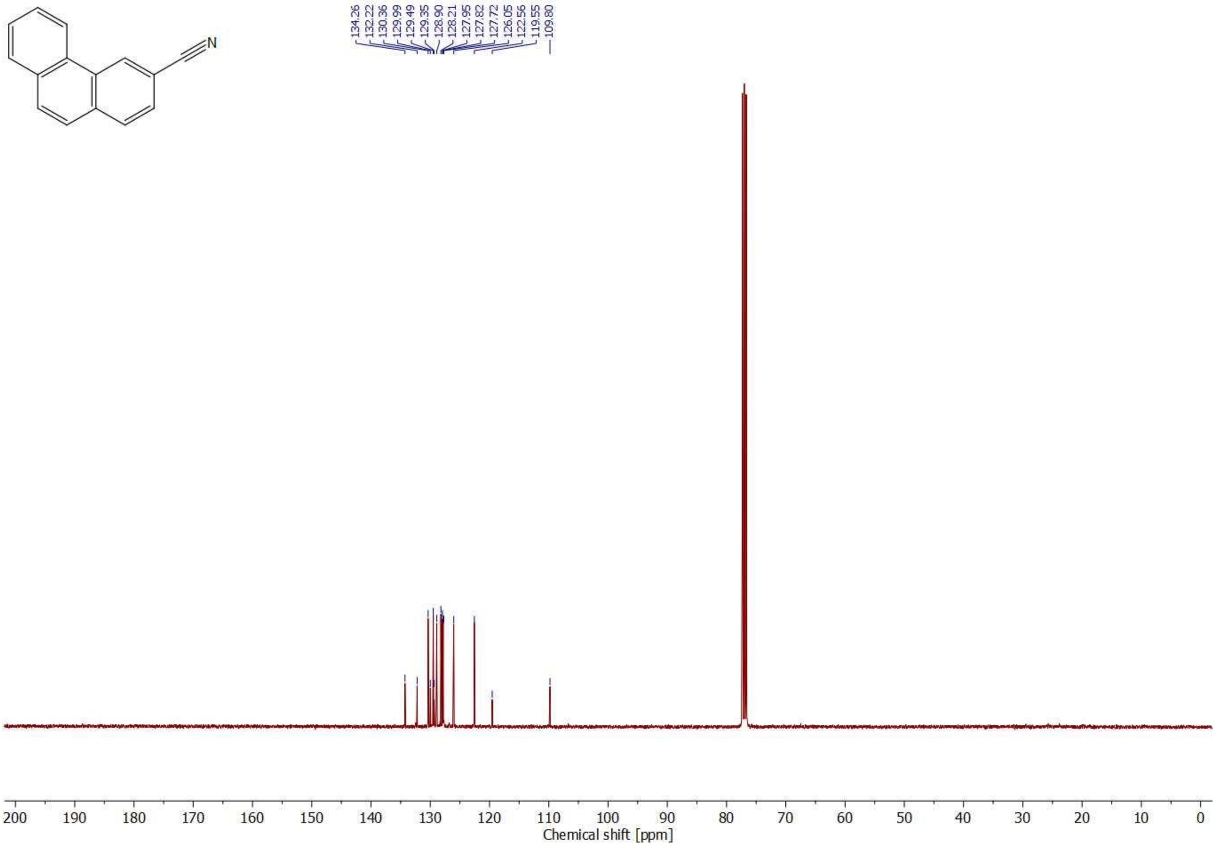

Figure S69. ^{1}H NMR spectrum of compound **4j** (101 MHz, $CDCl_3$)

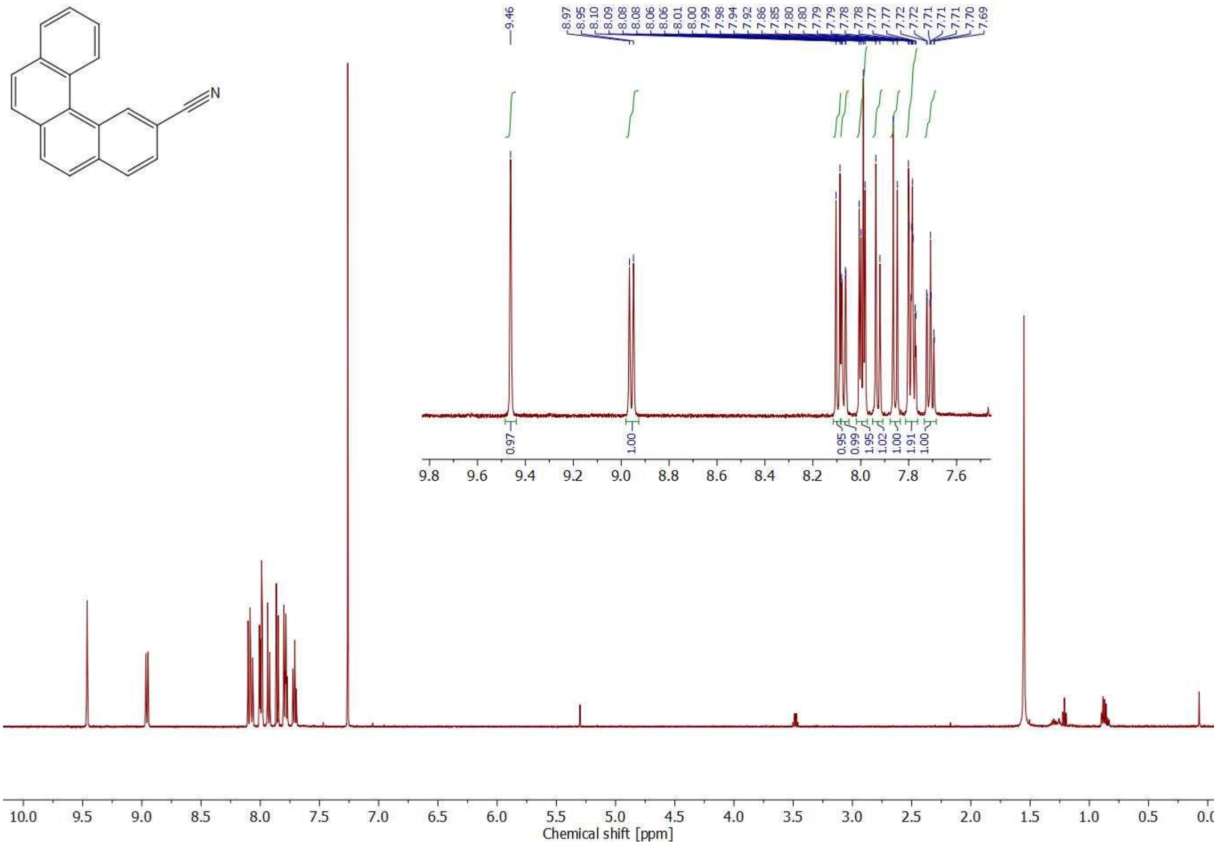

Figure S70. ^{1}H NMR spectrum of compound **4k** (500 MHz, $CDCl_3$)

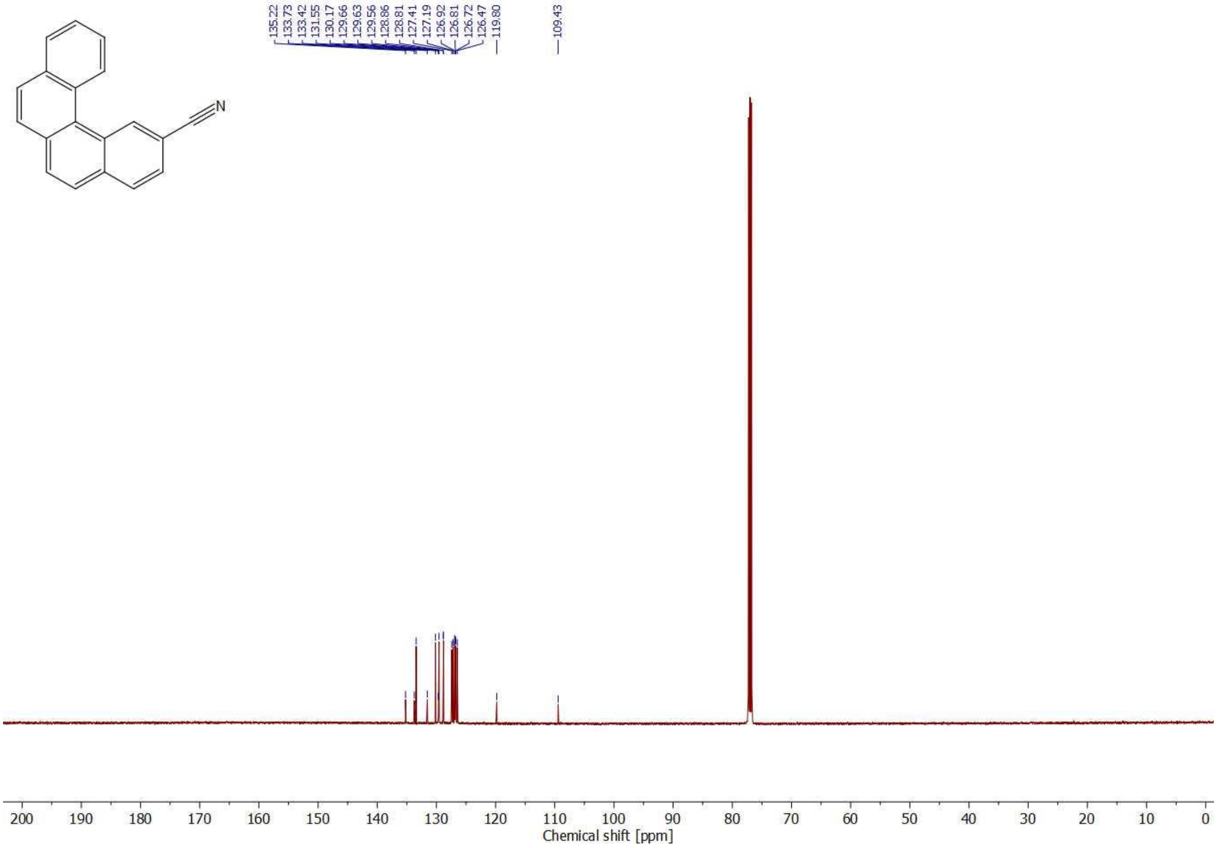

Figure S71. ^{13}C {^{1}H} NMR spectrum of compound **4k** (126 MHz, $CDCl_3$)

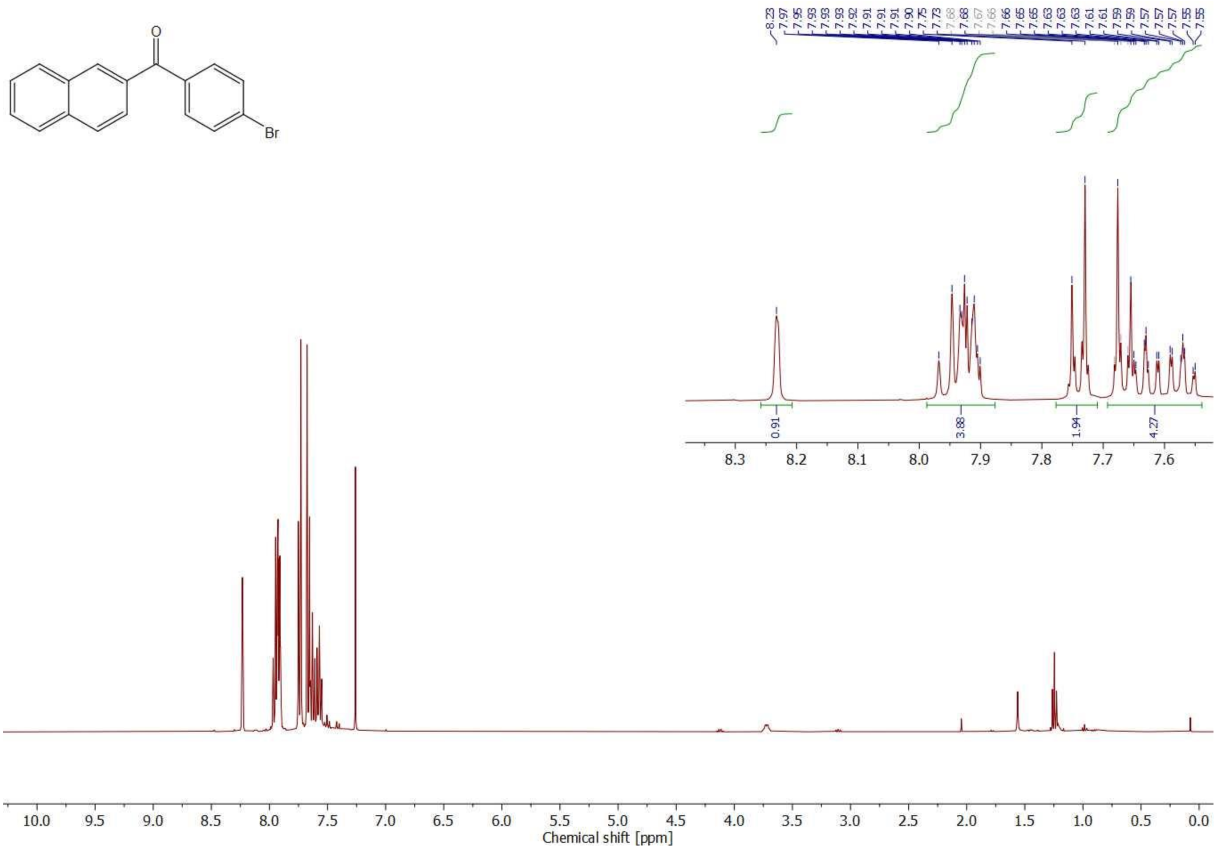

Figure S72. ^{1}H NMR spectrum of compound **5i** (400 MHz, $CDCl_3$)

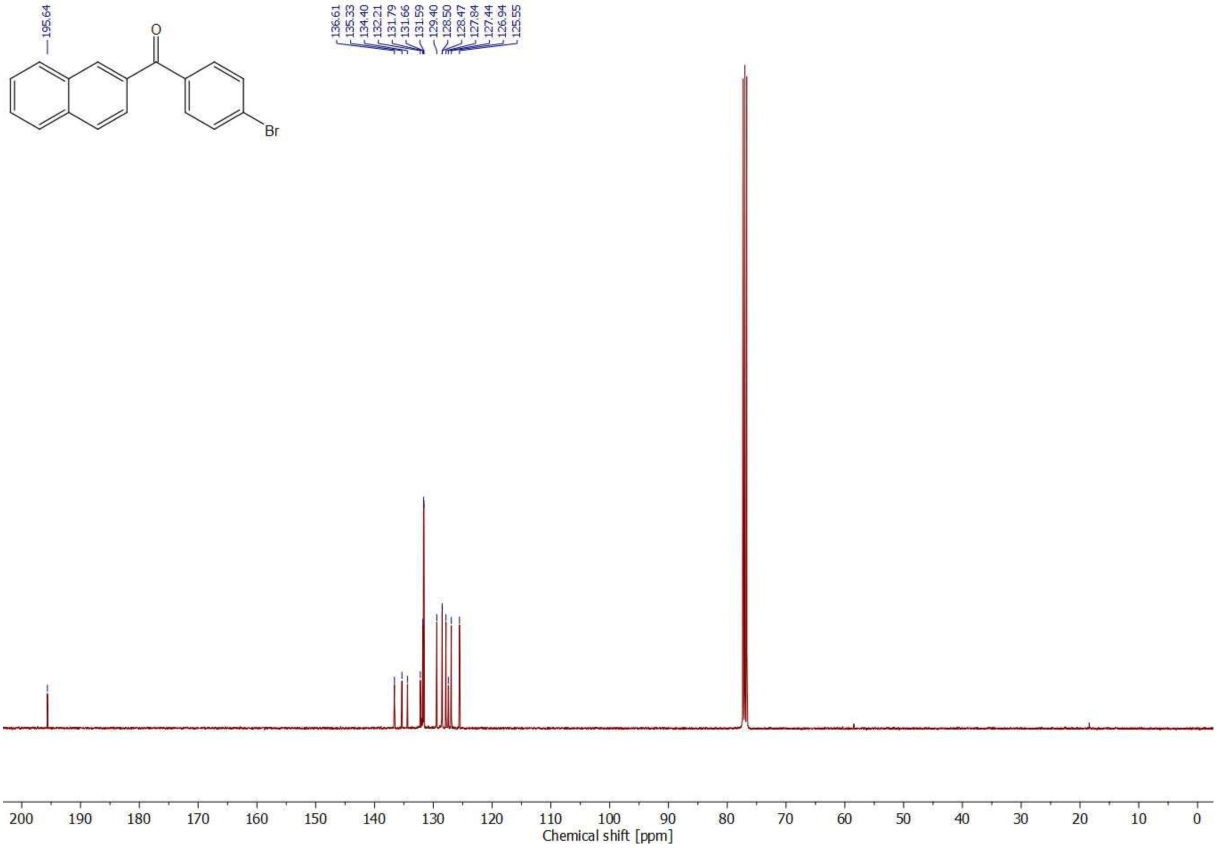

Figure S73. ^{13}C {^{1}H} NMR spectrum of compound **5i** (101 MHz, $CDCl_3$)

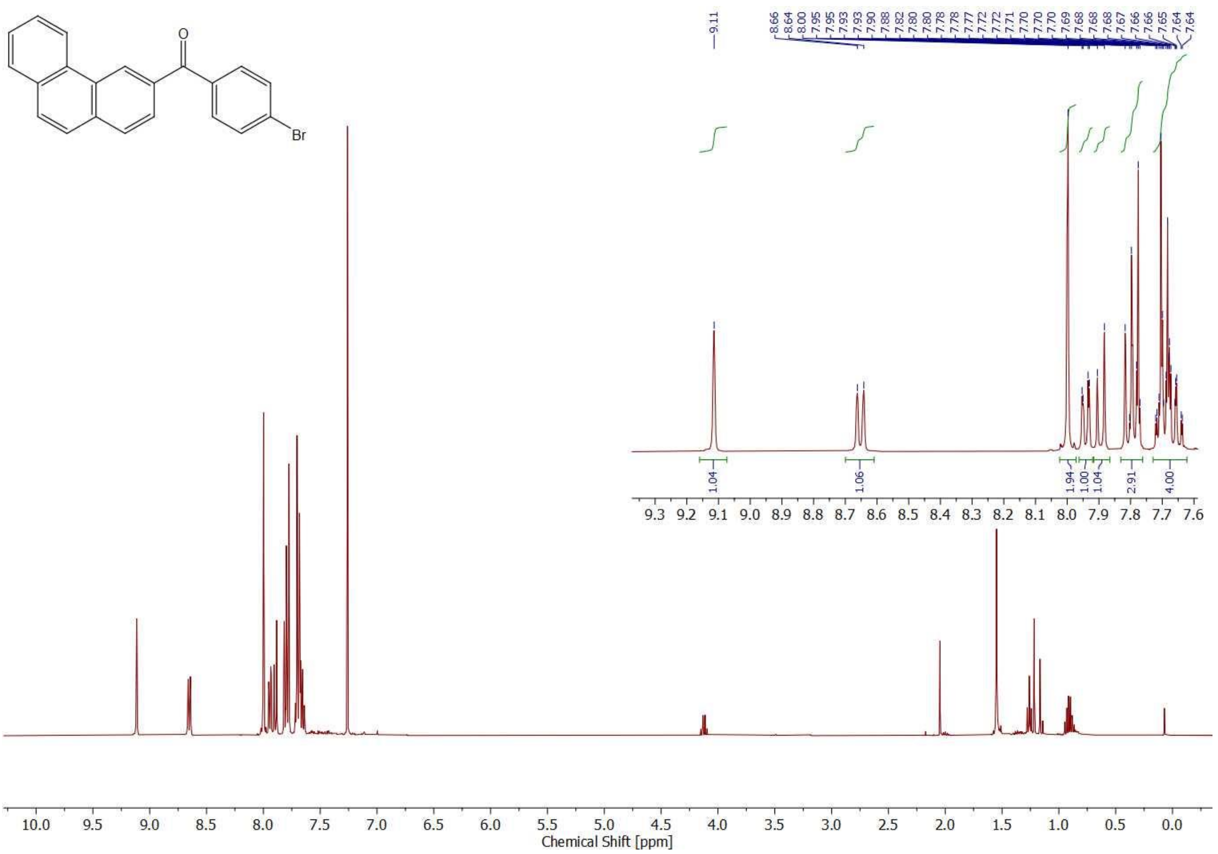

Figure S74. ^{1}H NMR spectrum of compound **5j** (400 MHz, $CDCl_3$)

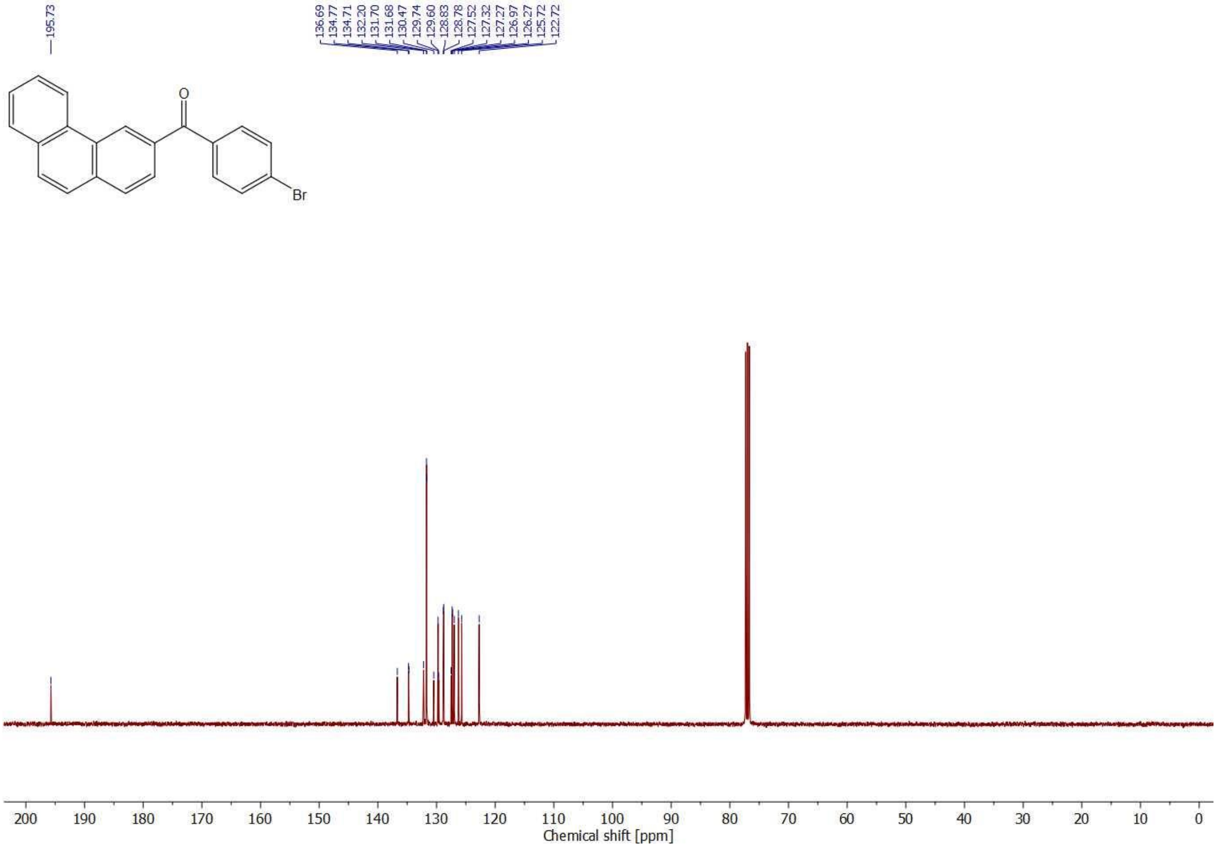

Figure S75. ^{13}C {^{1}H} NMR spectrum of compound **5j** (101 MHz, $CDCl_3$)

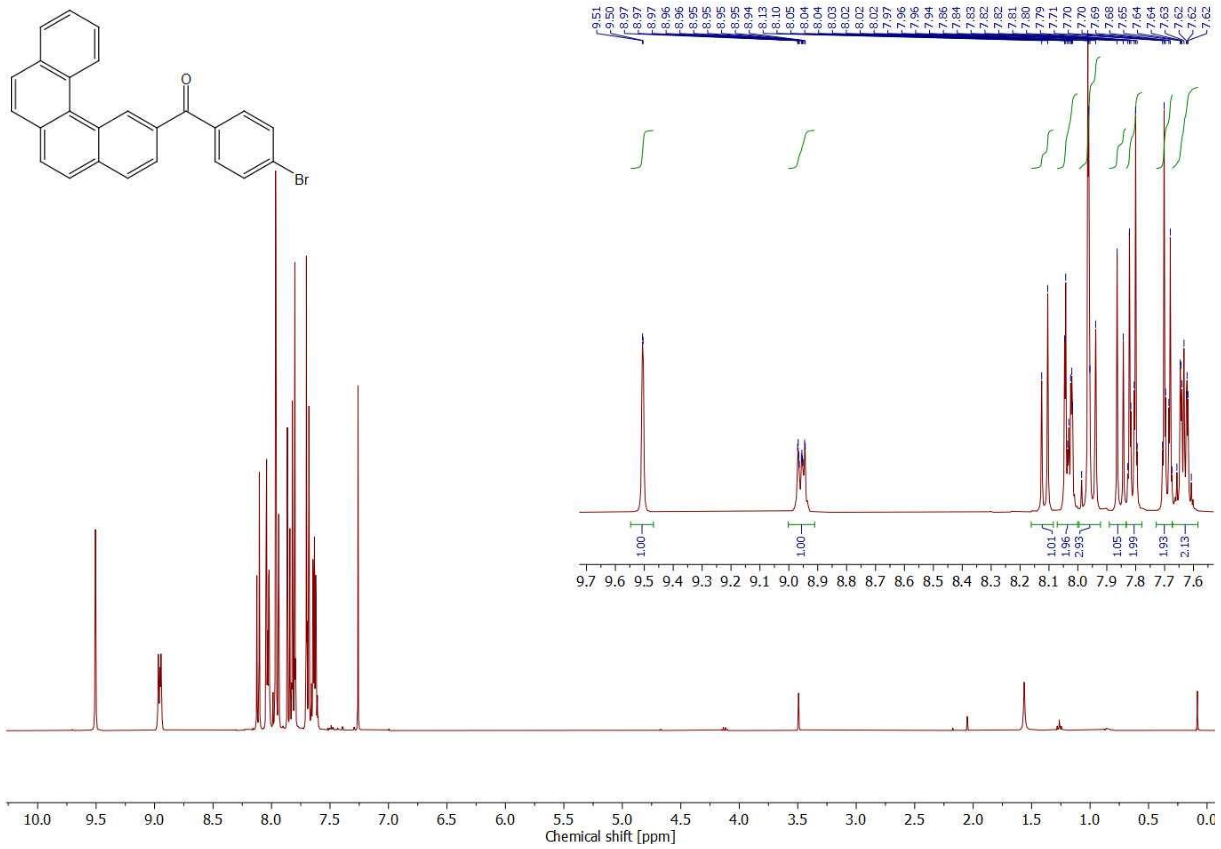

Figure S76. ^{1}H NMR spectrum of compound **5k** (400 MHz, $CDCl_3$)

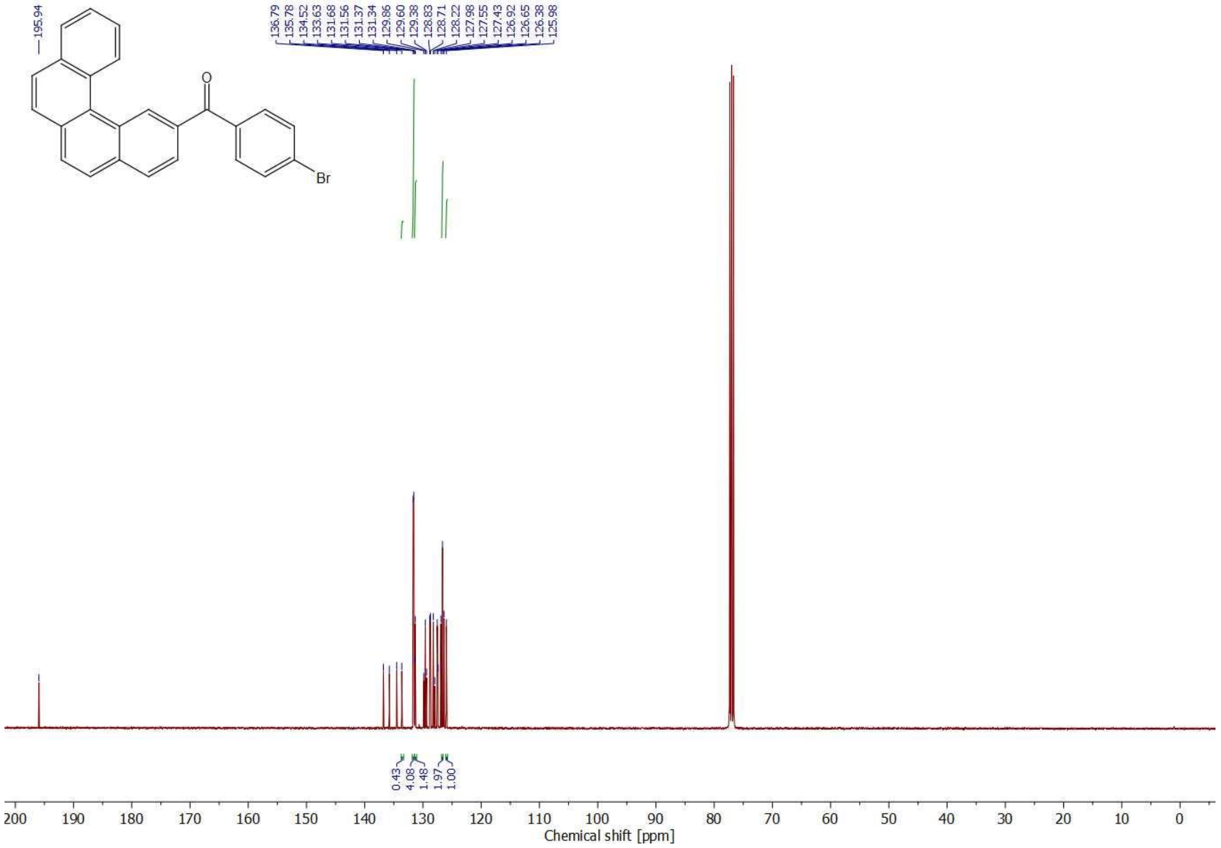

Figure S77. ^{13}C {^{1}H} NMR spectrum of compound **5k** (101 MHz, $CDCl_3$)

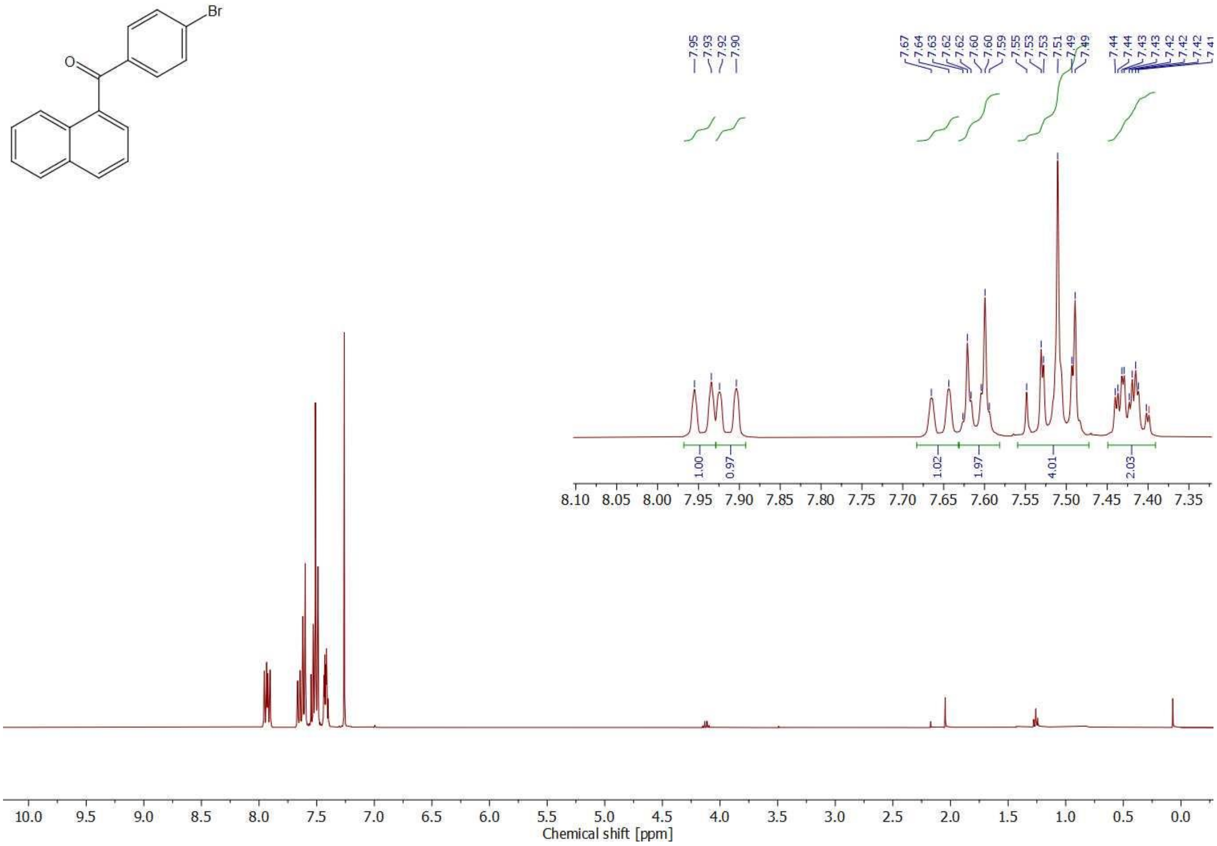

Figure S78. ^{1}H NMR spectrum of compound **5l** (400 MHz, $CDCl_3$)

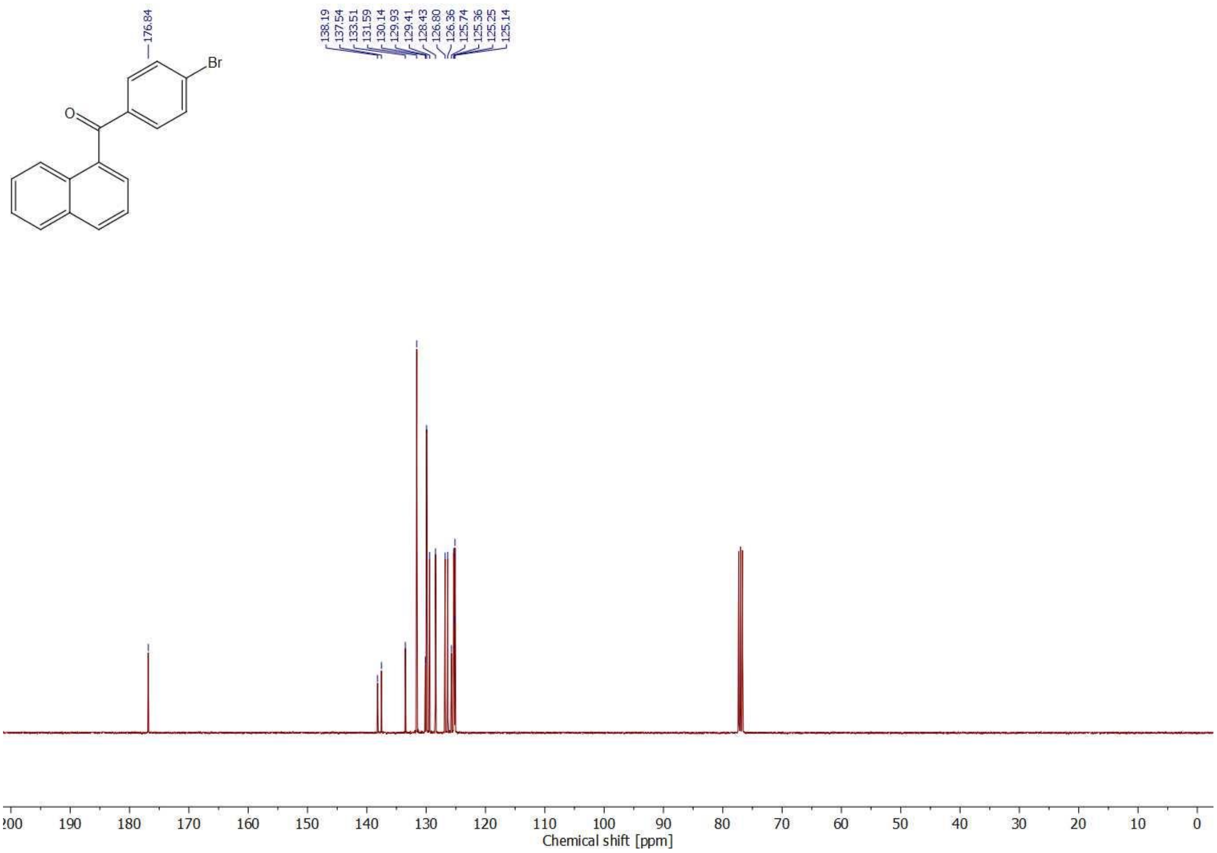

Figure S79. ^{13}C {^{1}H} NMR spectrum of compound **5l** (101 MHz, $CDCl_3$)

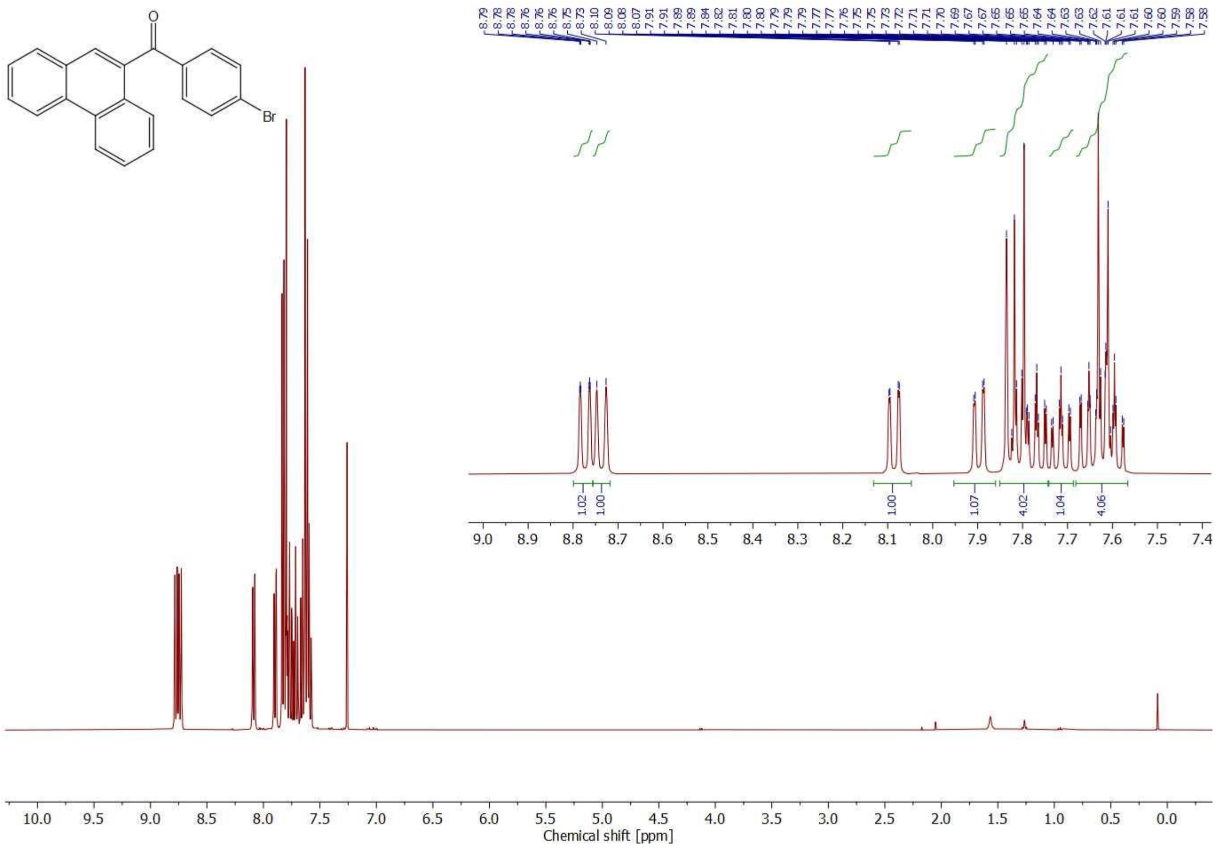

Figure S80. ^{1}H NMR spectrum of compound **5m** (400 MHz, $CDCl_3$)

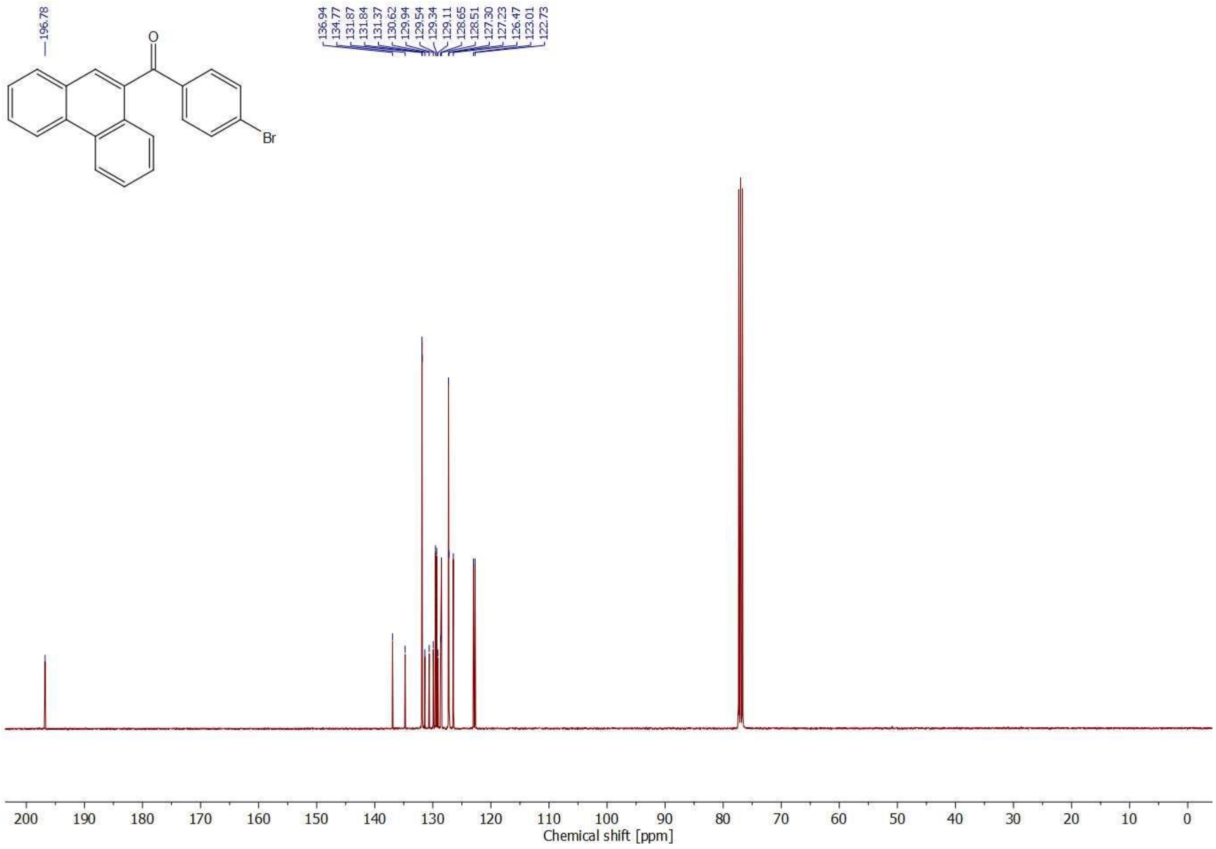

Figure S81. ^{13}C {^{1}H} NMR spectrum of compound **5m** (101 MHz, $CDCl_3$)

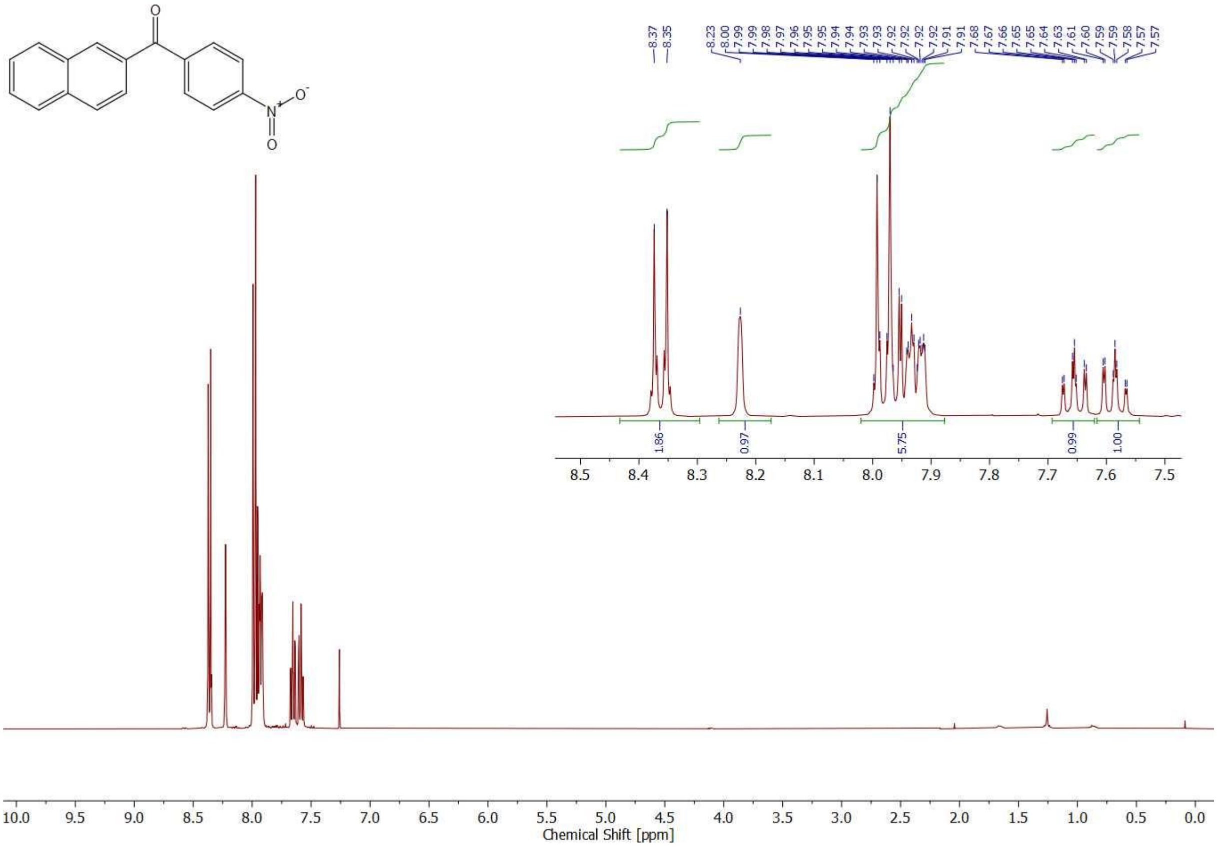

Figure S82. ^{1}H NMR spectrum of compound **6i** (400 MHz, $CDCl_3$)

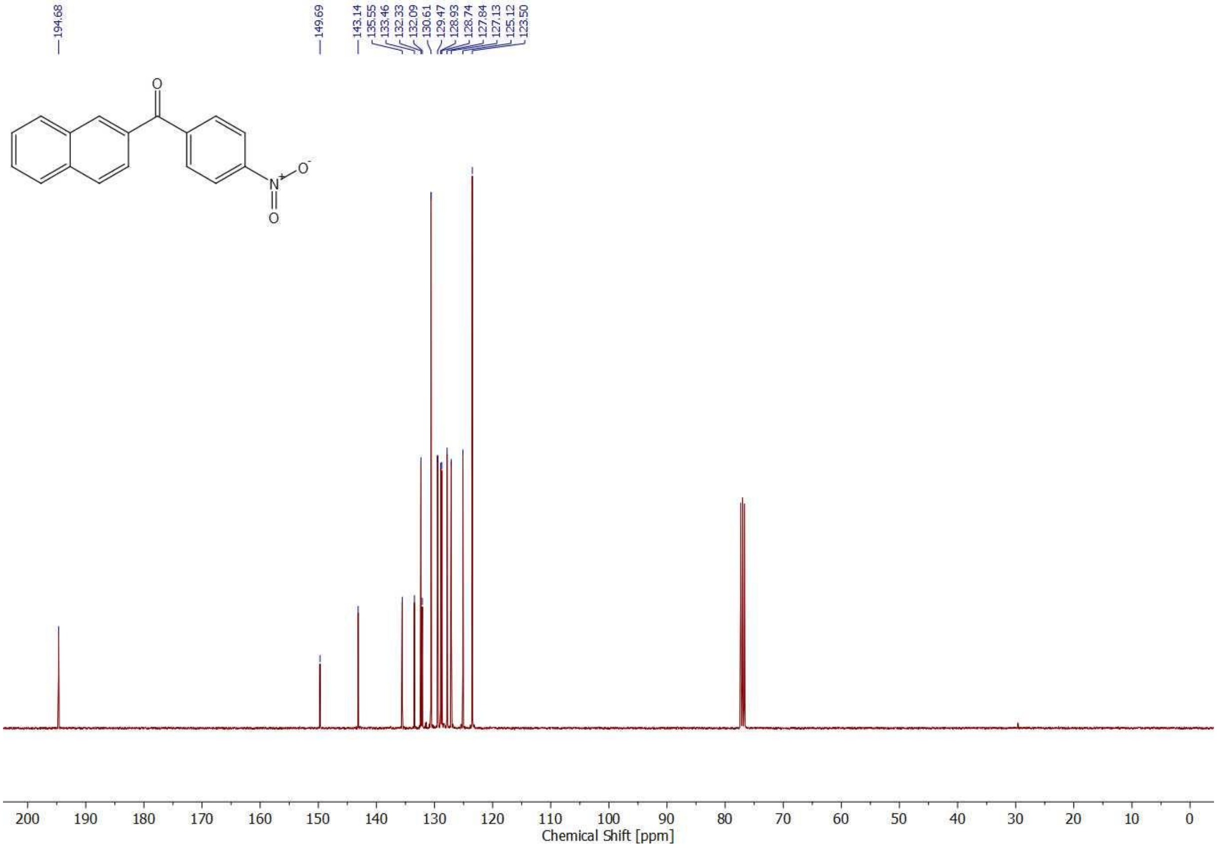

Figure S83. ^{13}C {^{1}H} NMR spectrum of compound **6i** (101 MHz, $CDCl_3$)

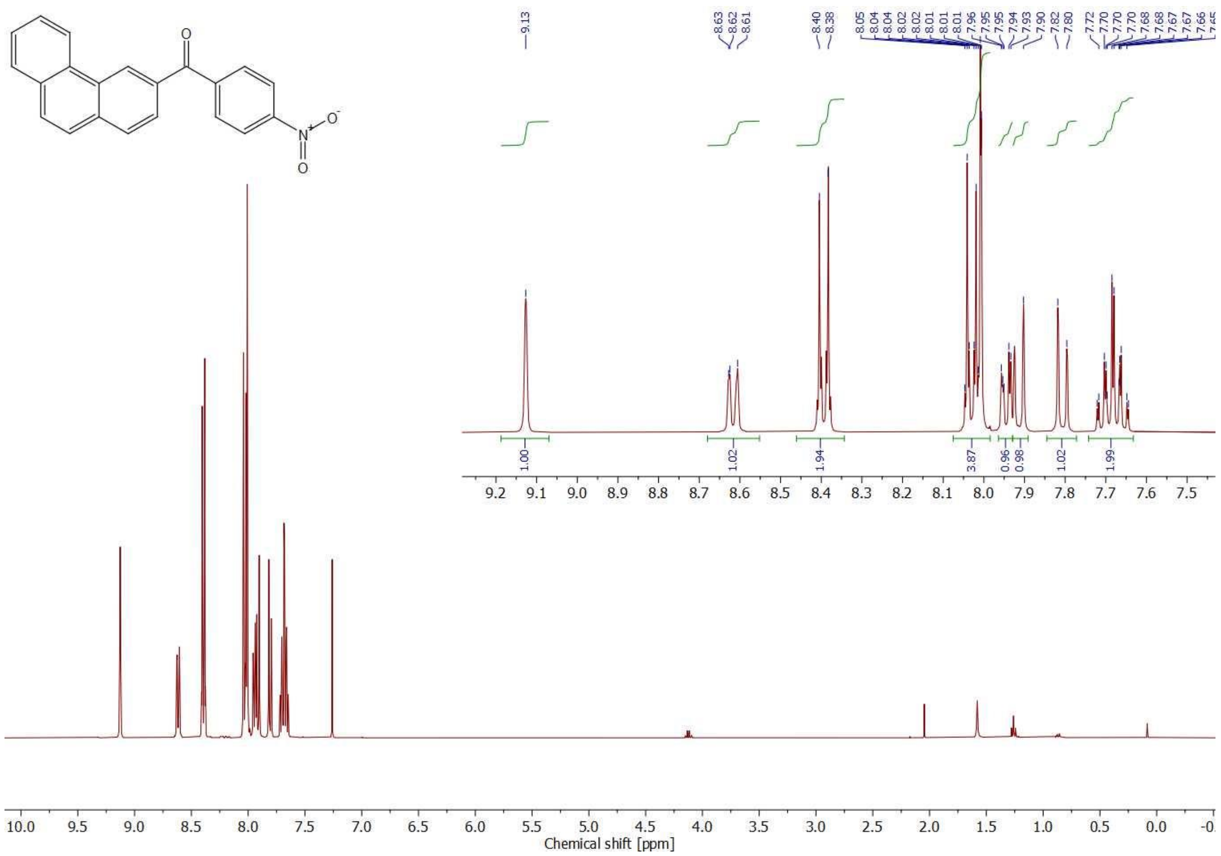

Figure S84. ^{1}H NMR spectrum of compound **6j** (400 MHz, $CDCl_3$)

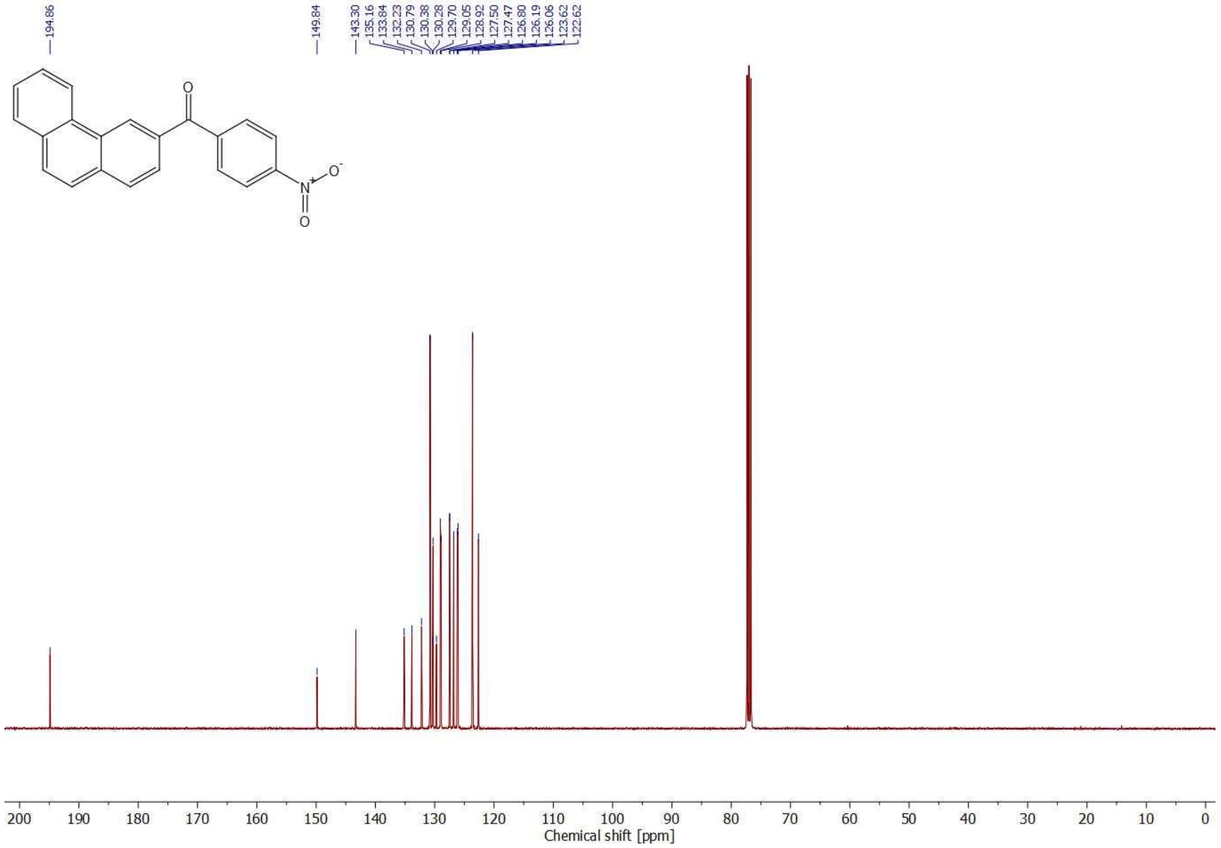

Figure S85. ^{13}C {^{1}H} NMR spectrum of compound **6j** (101 MHz, $CDCl_3$)

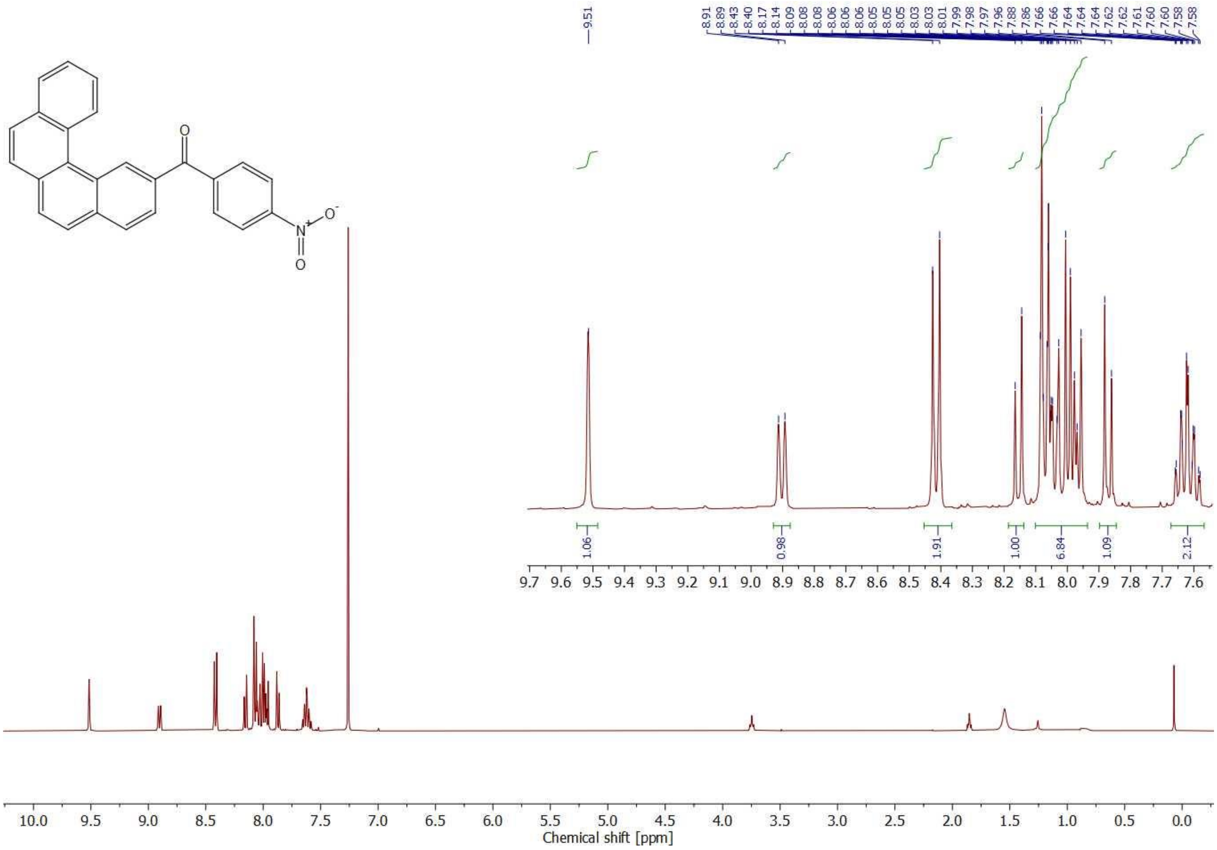

Figure S86. ^{1}H NMR spectrum of compound **6k** (400 MHz, $CDCl_3$)

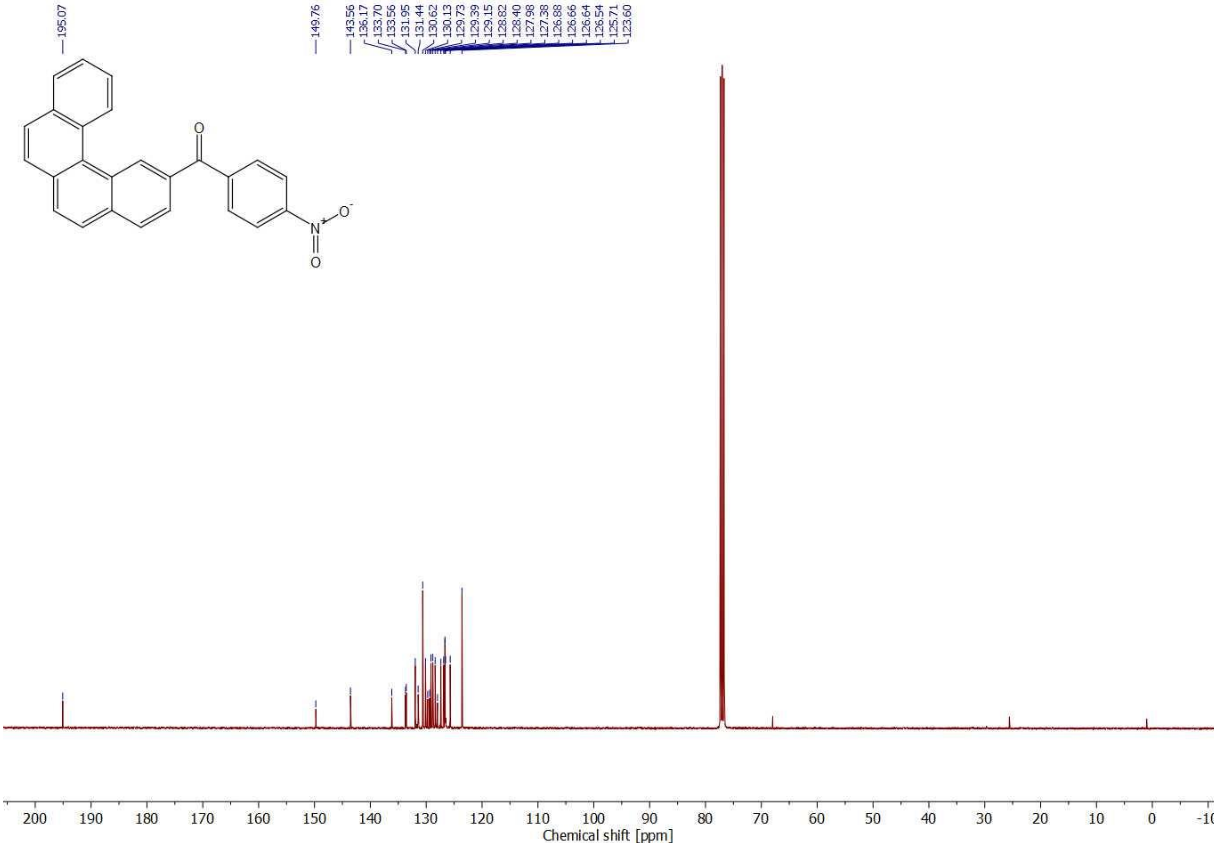

Figure S87. ^{13}C {^{1}H} NMR spectrum of compound **6k** (101 MHz, $CDCl_3$)

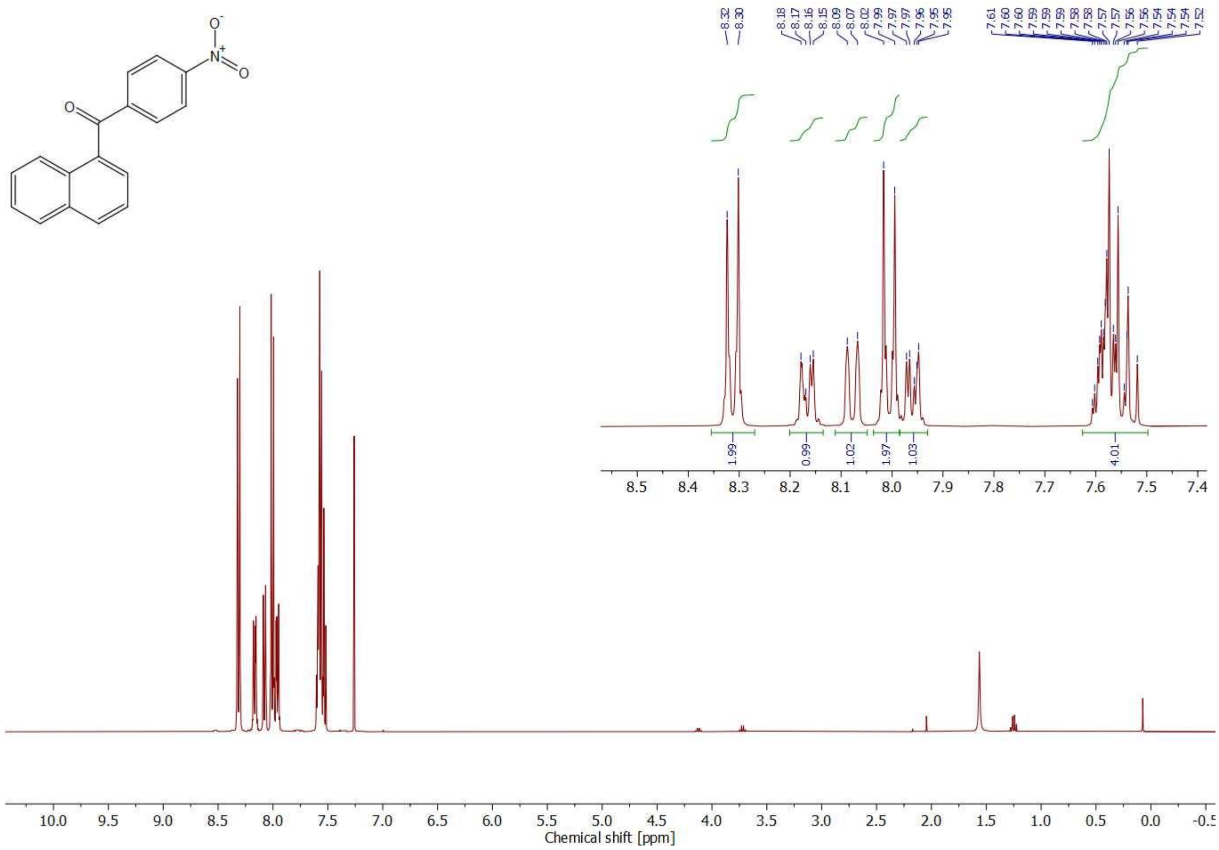

Figure S88. ^{1}H NMR spectrum of compound **6l** (400 MHz, $CDCl_3$)

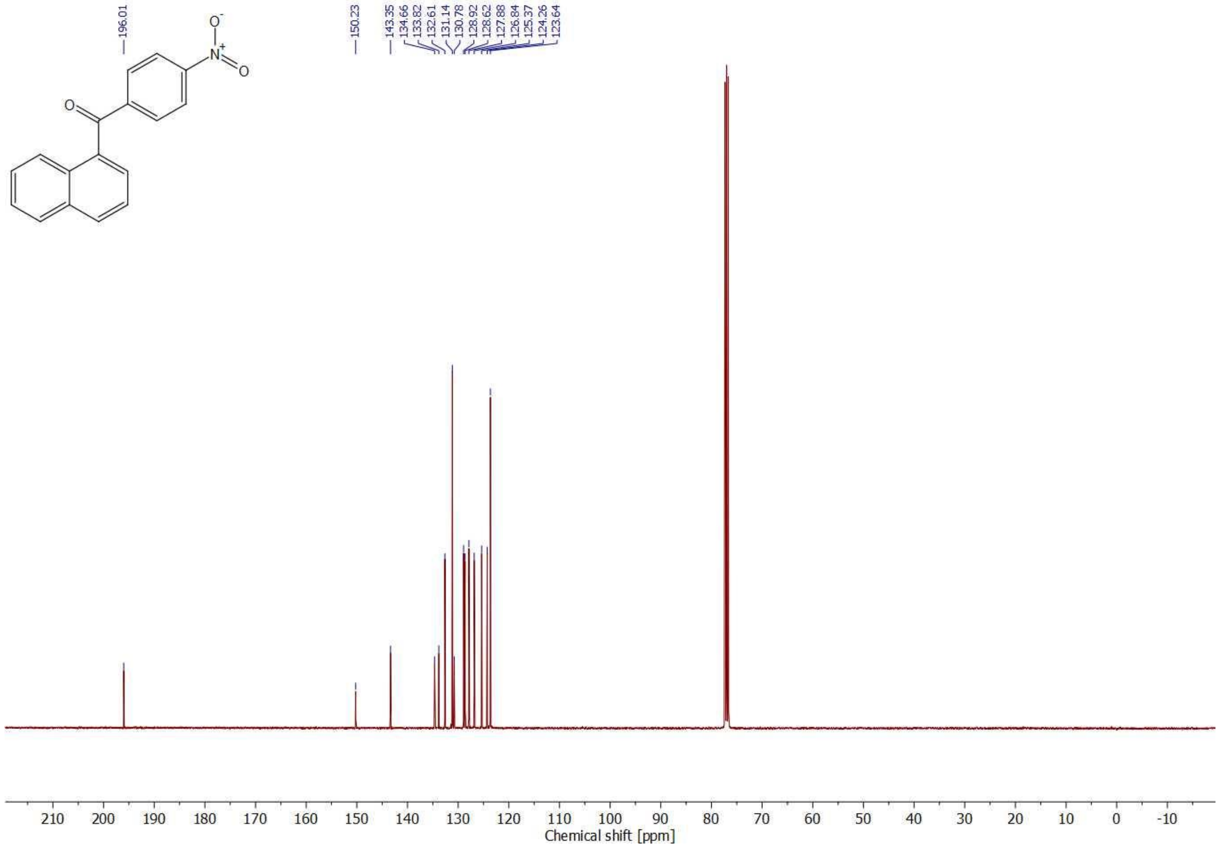

Figure S89. ^{13}C {^{1}H} NMR spectrum of compound **6l** (101 MHz, $CDCl_3$)

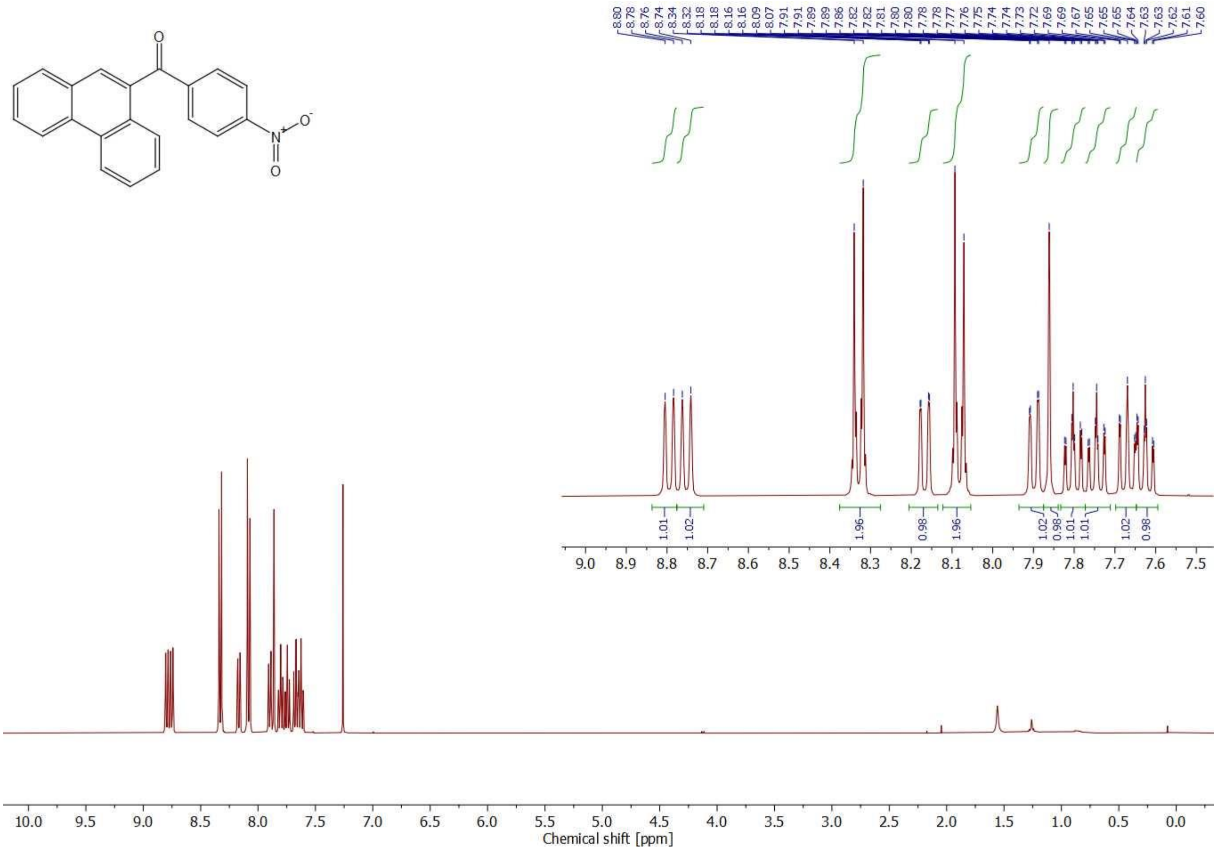

Figure S90. ^{1}H NMR spectrum of compound **6m** (400 MHz, $CDCl_3$)

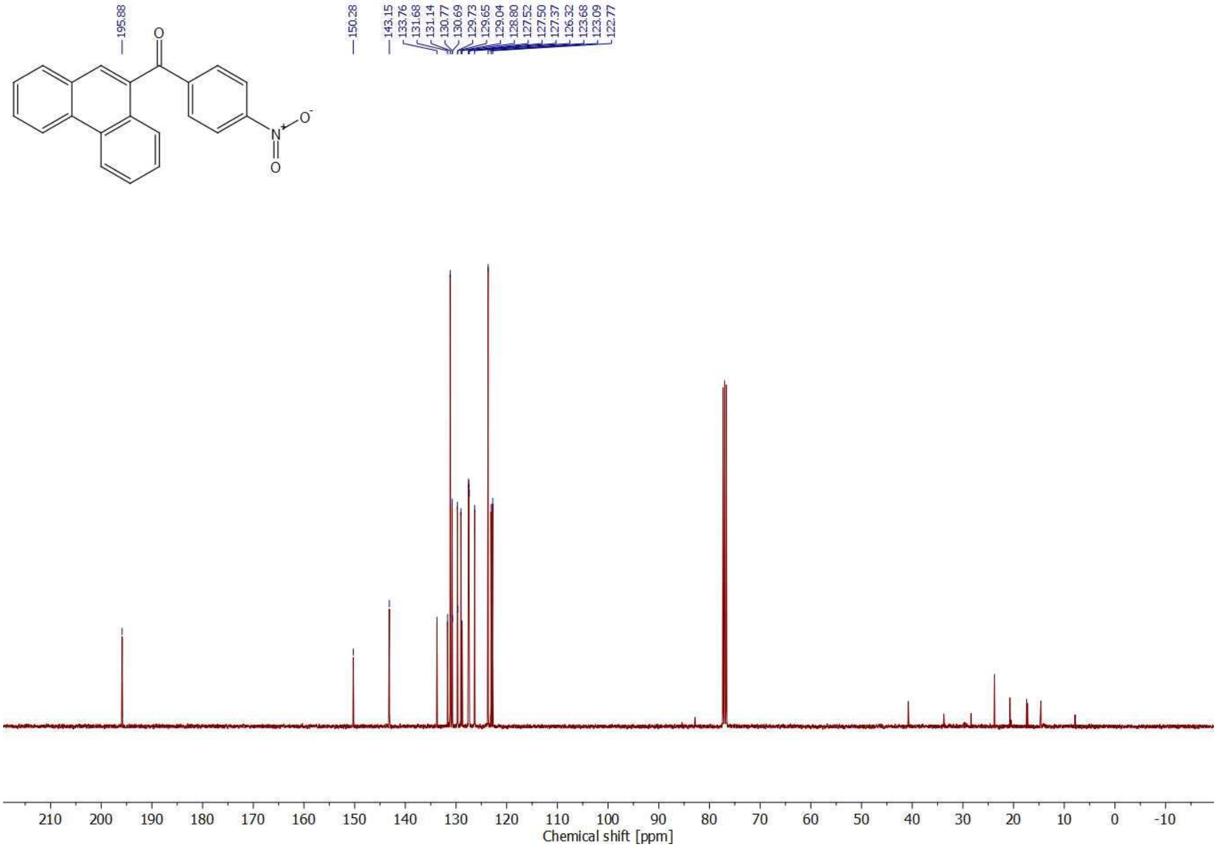

Figure S91. ^{13}C {^{1}H} NMR spectrum of compound **6m** (101 MHz, $CDCl_3$)

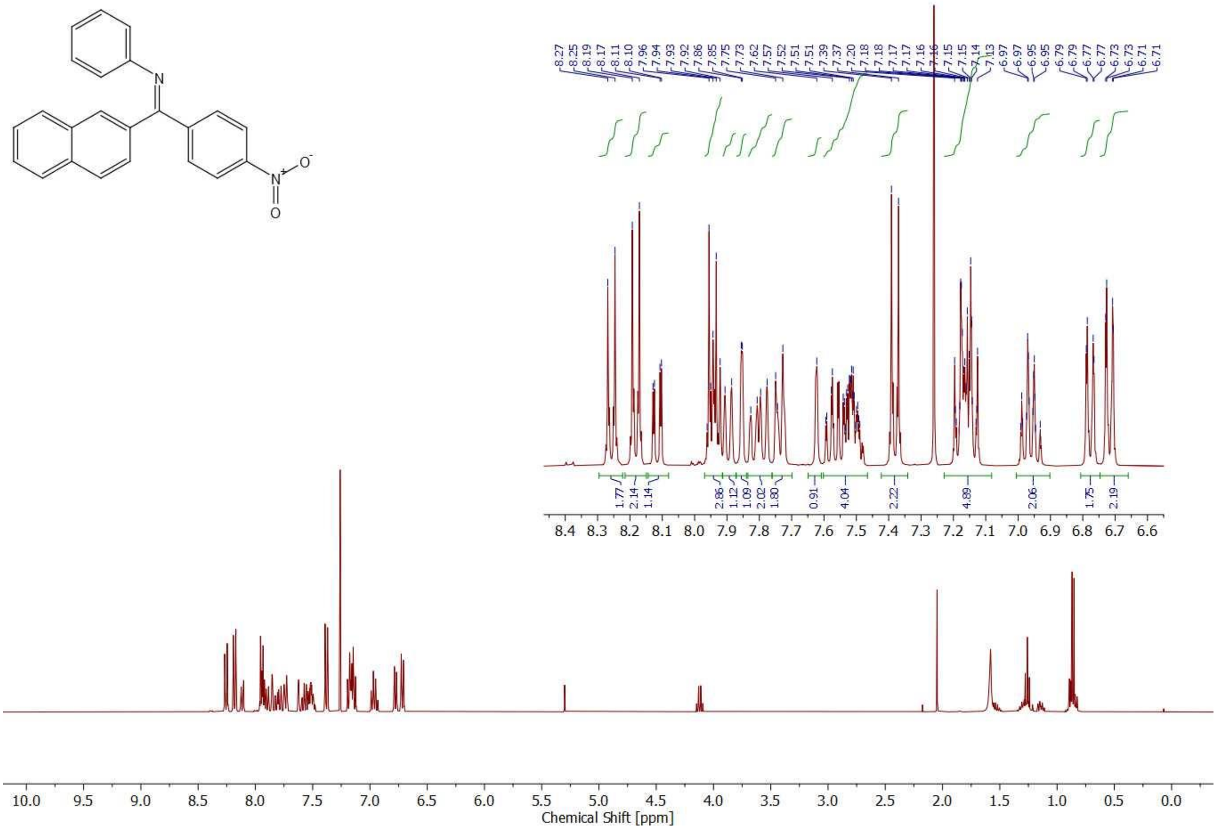

Figure S92. ^{1}H NMR spectrum of compound **1i** (400 MHz, $CDCl_3$)

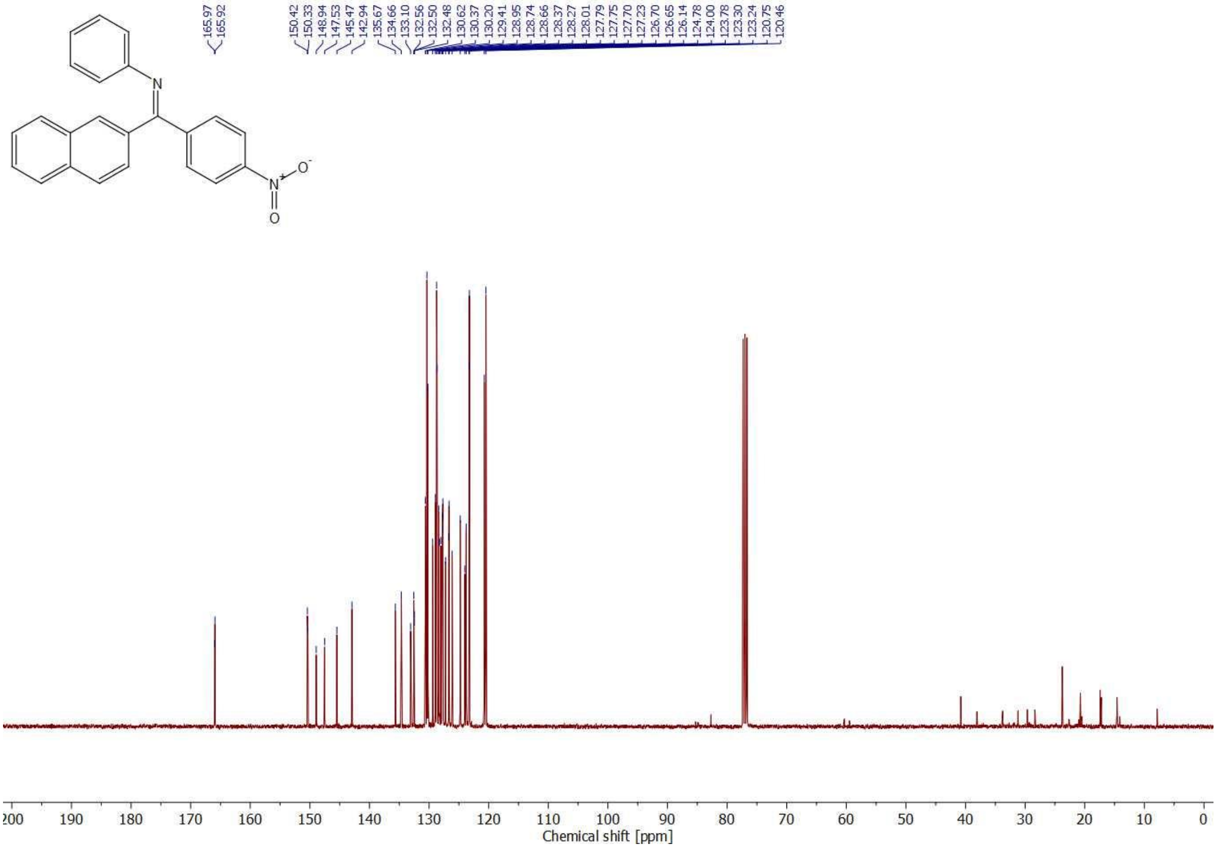

Figure S93. ^{13}C {^{1}H} NMR spectrum of compound **1i** (101 MHz, $CDCl_3$)

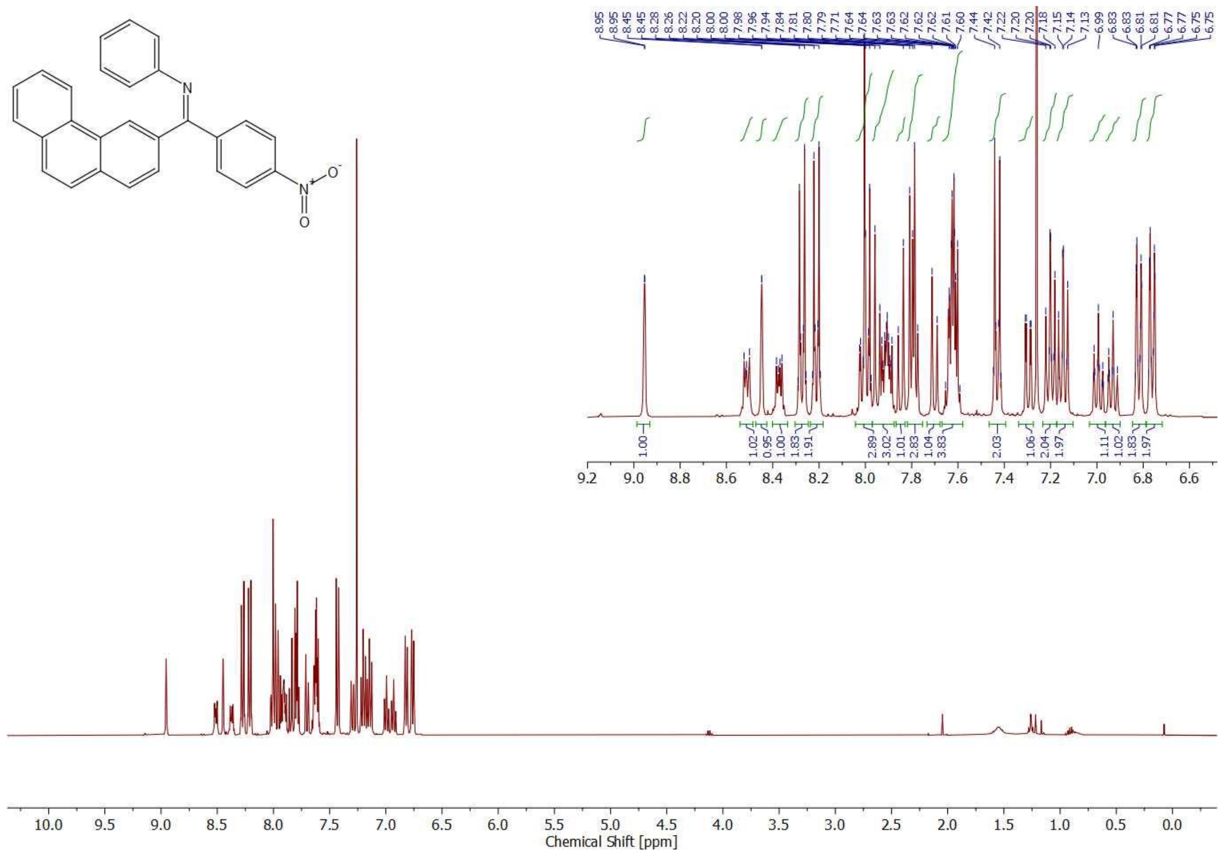

Figure S94. ^1H NMR spectrum of compound **1j** (400 MHz, $CDCl_3$)

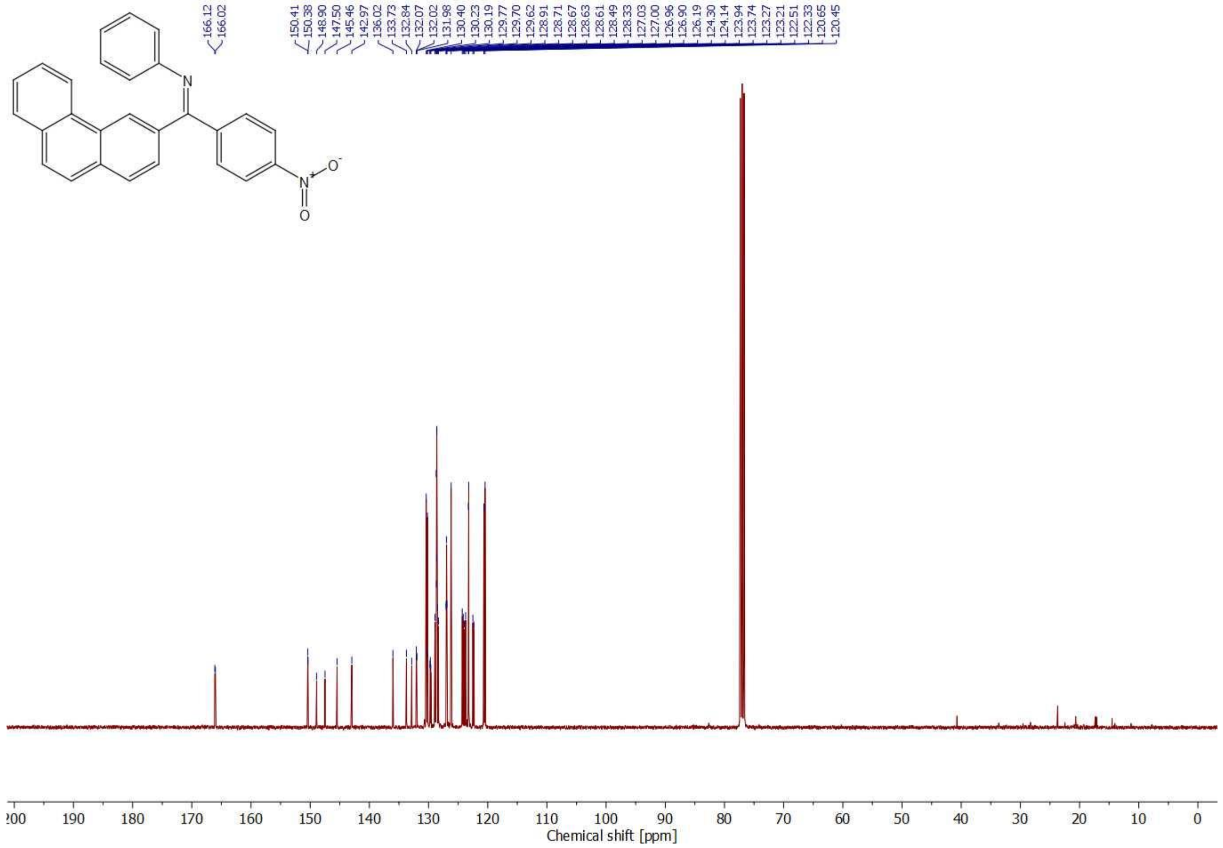

Figure S95. $^{13}C\ \{^1H\}$ NMR spectrum of compound **1j** (101 MHz, $CDCl_3$)

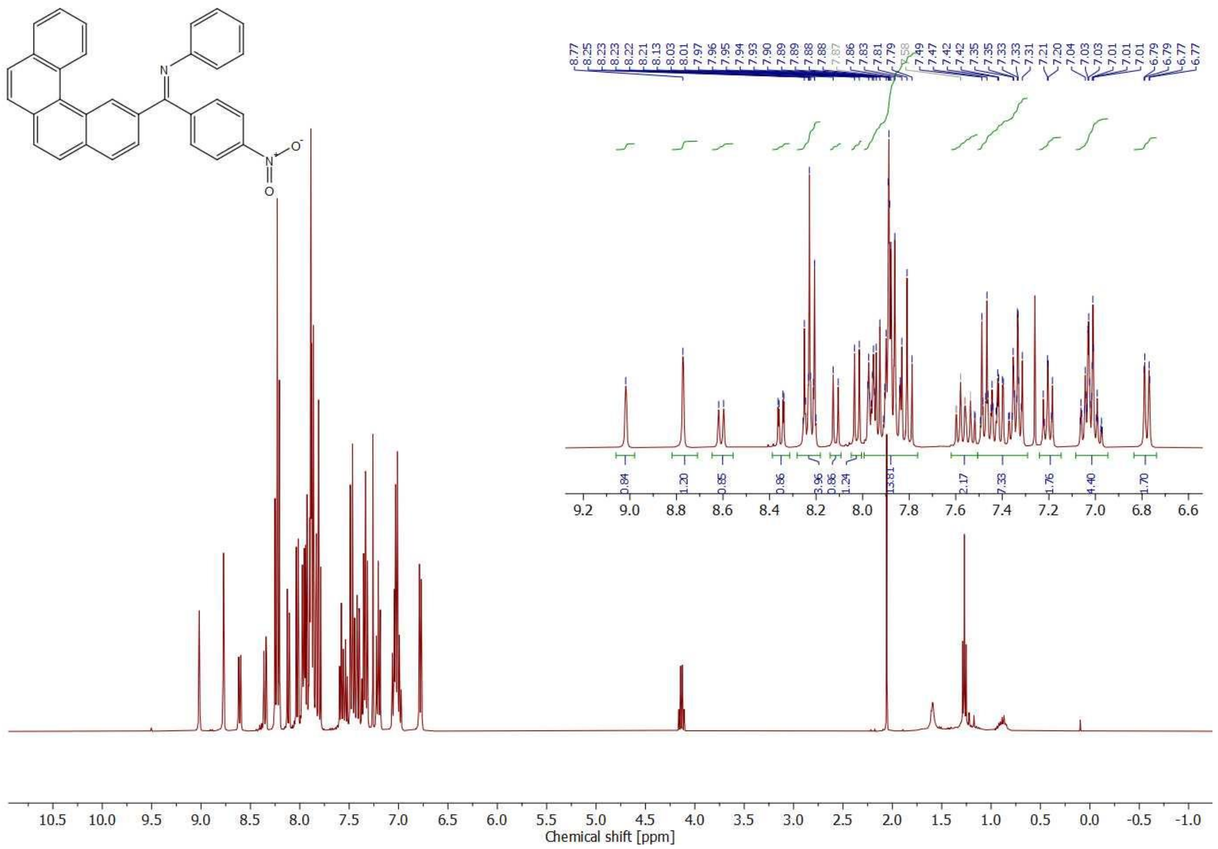

Figure S96. ^{1}H NMR spectrum of compound **1k** (400 MHz, $CDCl_3$)

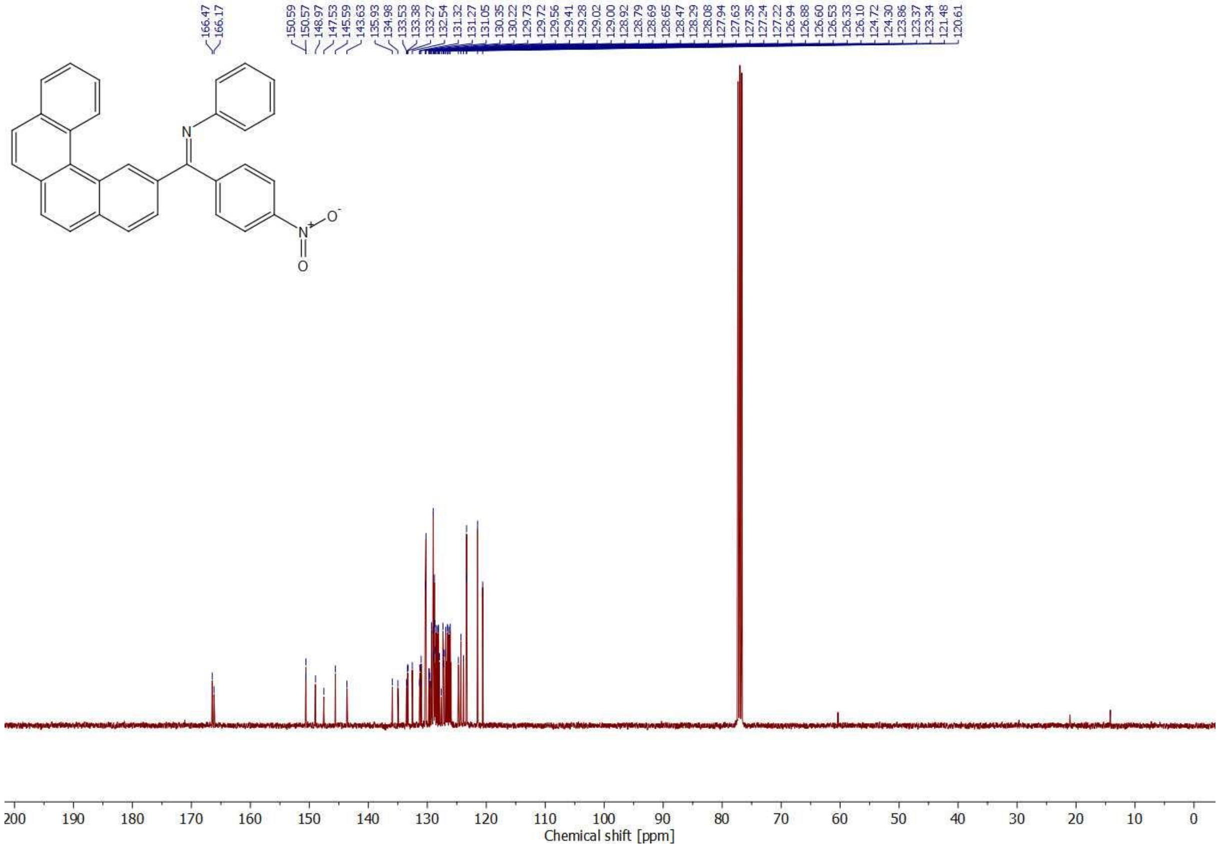

Figure S97. ^{13}C {^{1}H} NMR spectrum of compound **1k** (101 MHz, $CDCl_3$)

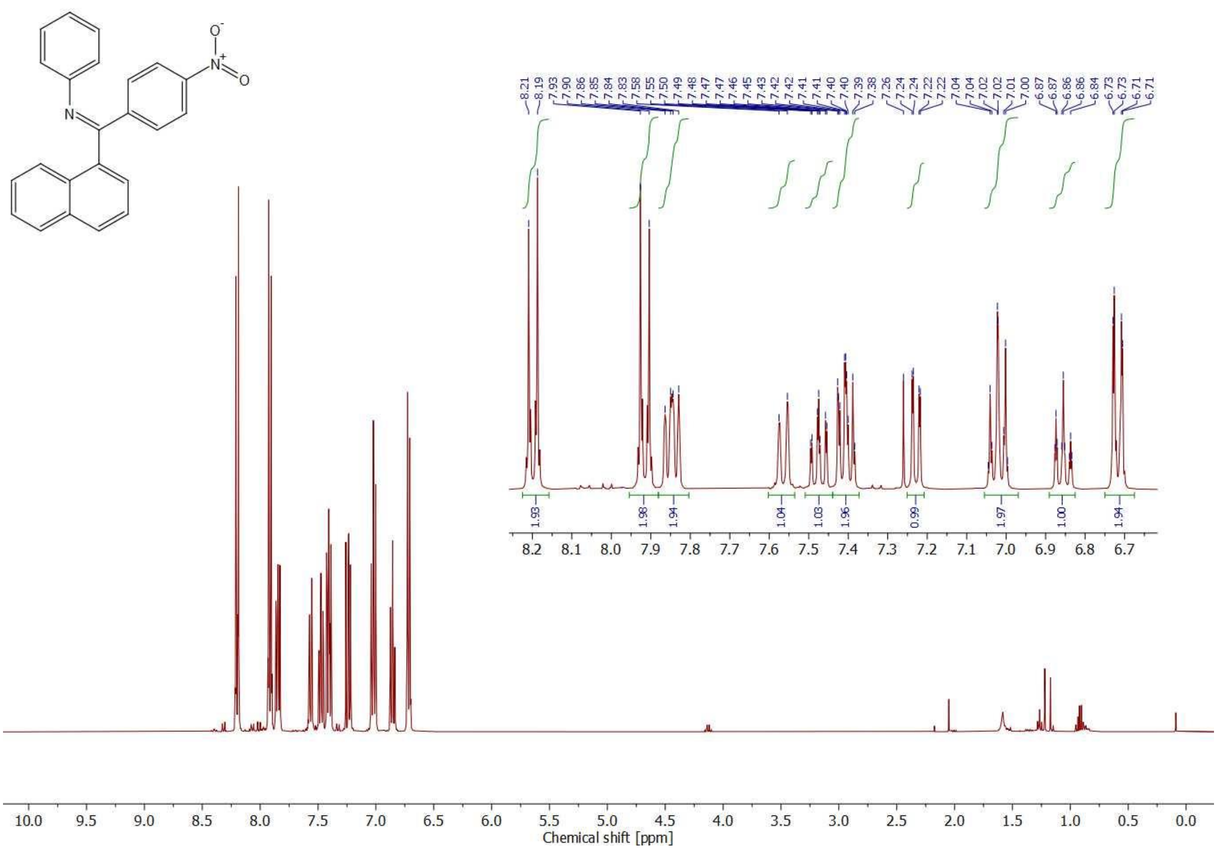

Figure S98. ^{1}H NMR spectrum of compound **1l** (400 MHz, $CDCl_3$)

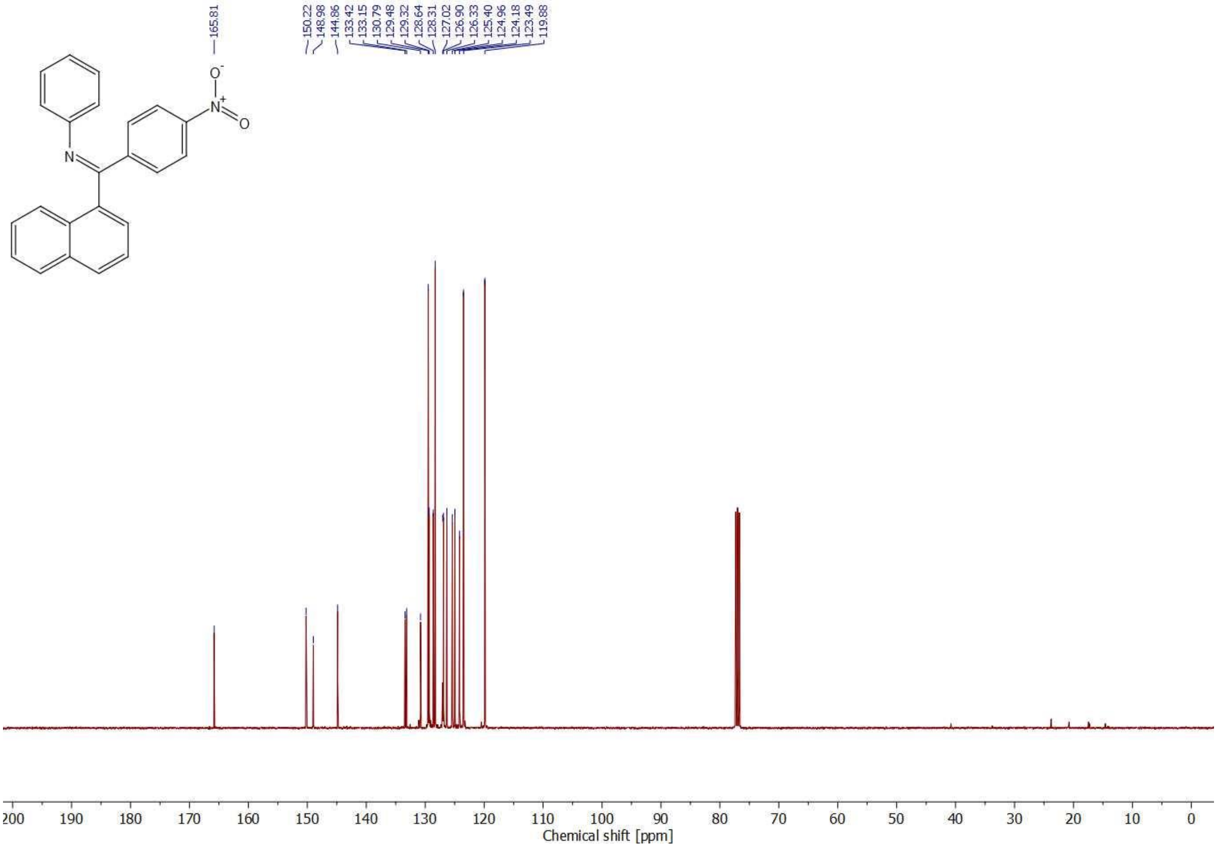

Figure S99. ^{13}C {^{1}H} NMR spectrum of compound **1l** (101 MHz, $CDCl_3$)

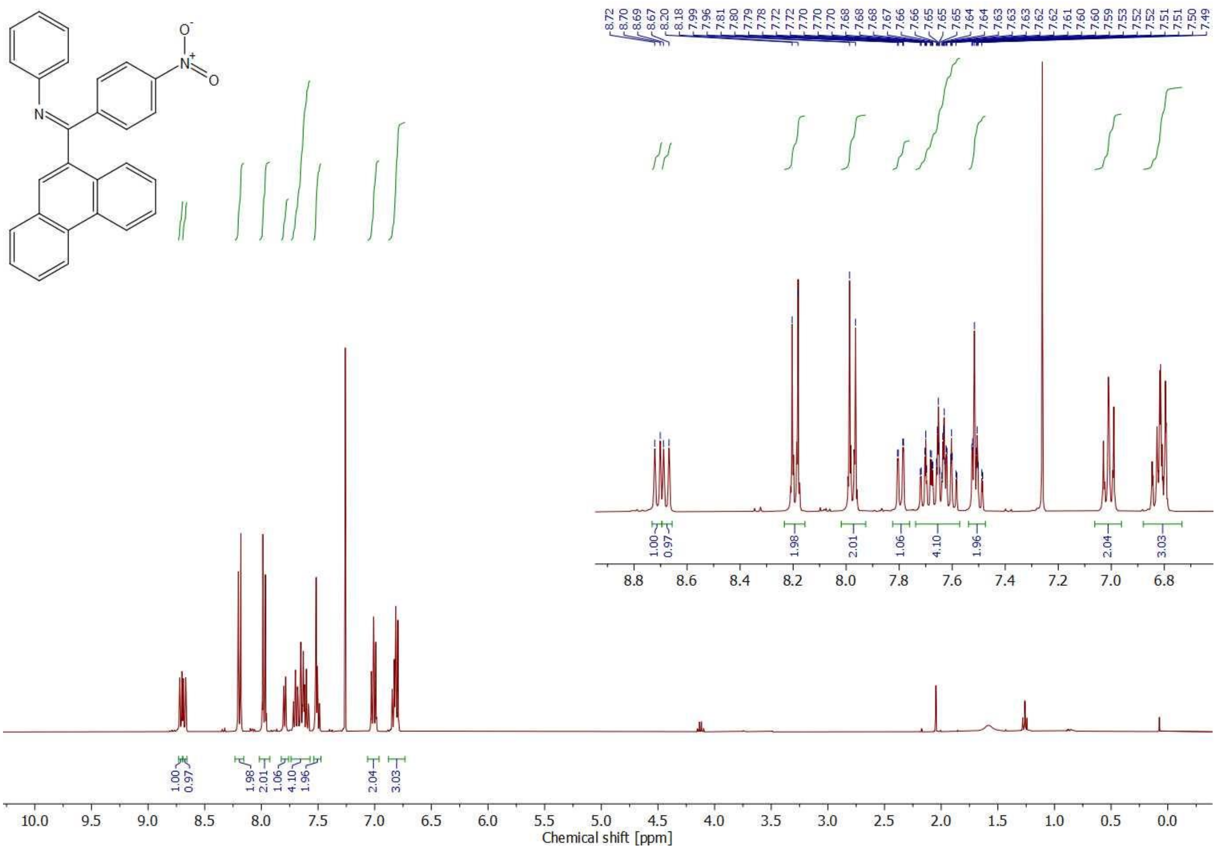

Figure S100 ^{1}H NMR spectrum of compound **1m** (400 MHz, $CDCl_3$)

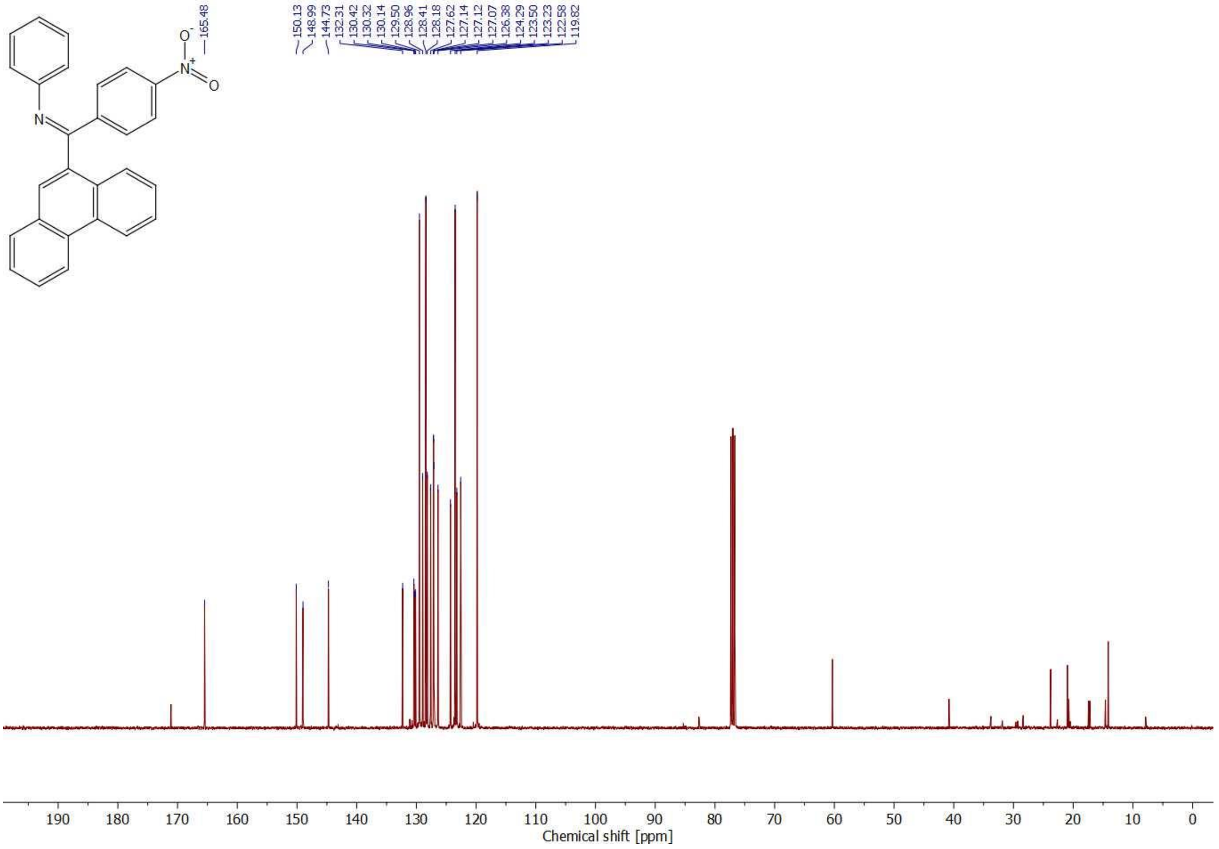

Figure S101 ^{13}C {^{1}H} NMR spectrum of compound **1m** (101 MHz, $CDCl_3$)

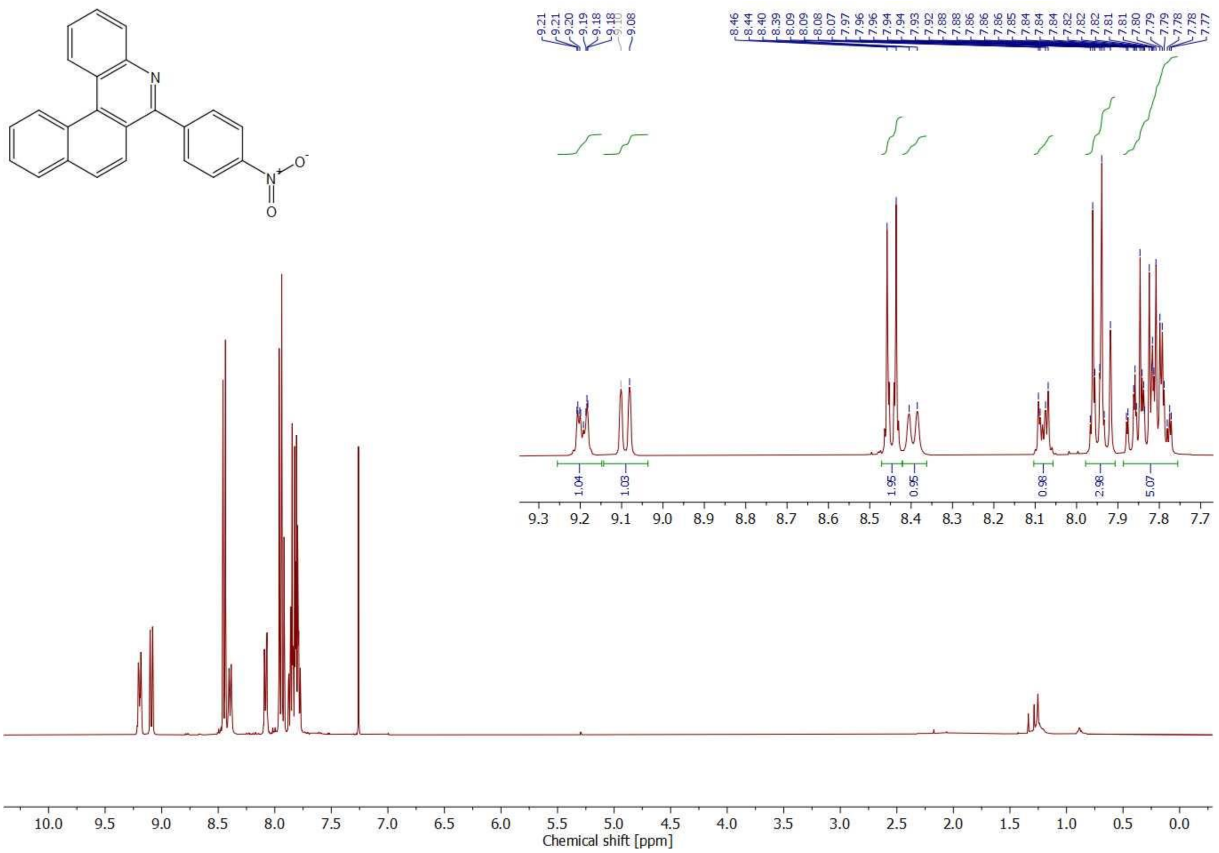

Figure S102. ^{1}H NMR spectrum of compound **3i** (400 MHz, $CDCl_3$)

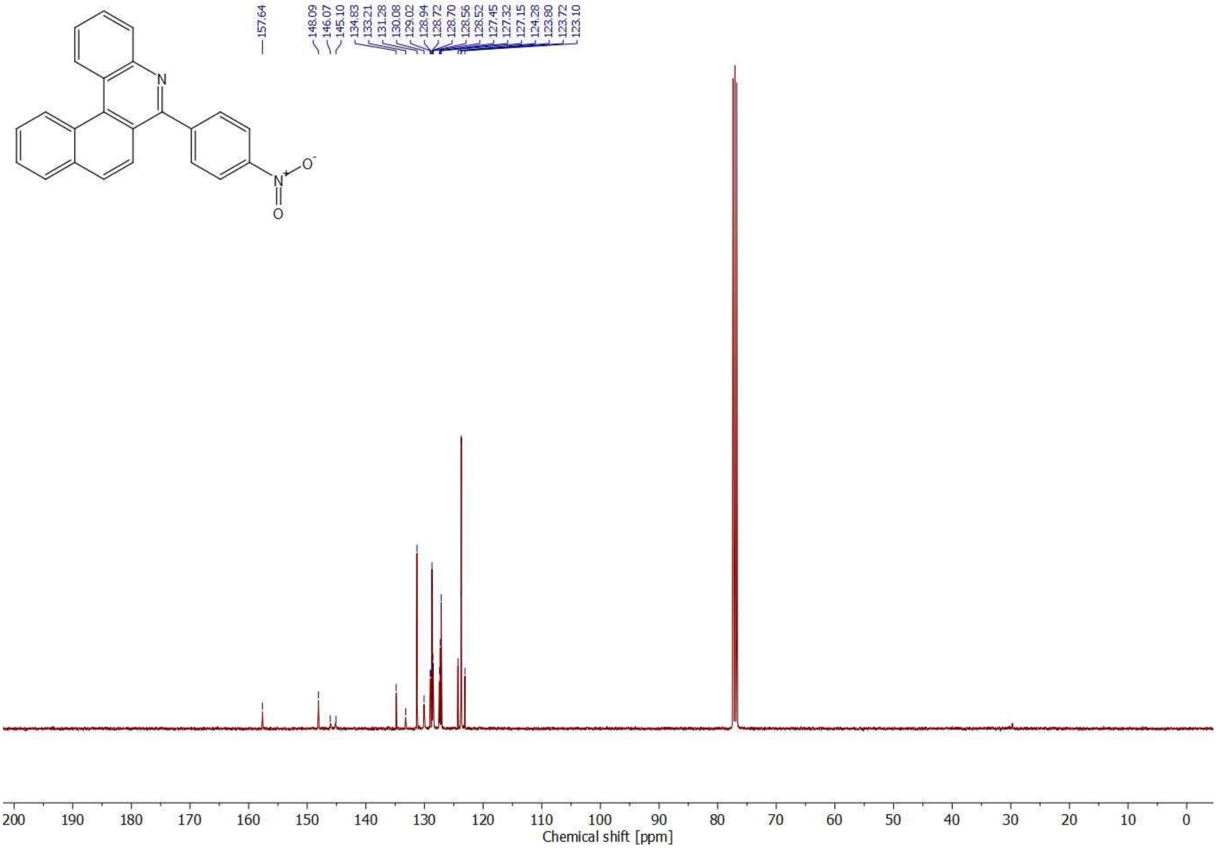

Figure S103. ^{13}C {^{1}H} NMR spectrum of compound **3i** (101 MHz, $CDCl_3$)

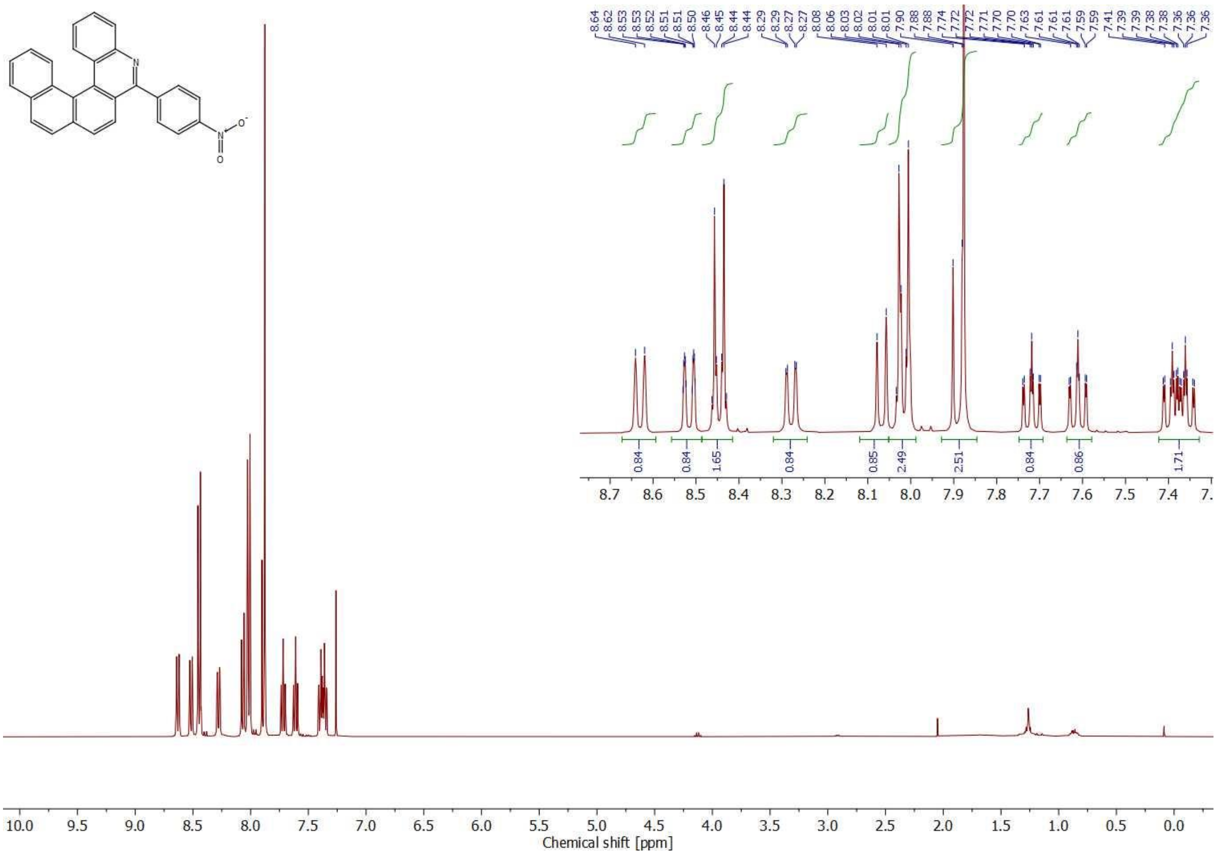

Figure S104. ^{1}H NMR spectrum of compound **3j** (400 MHz, $CDCl_3$)

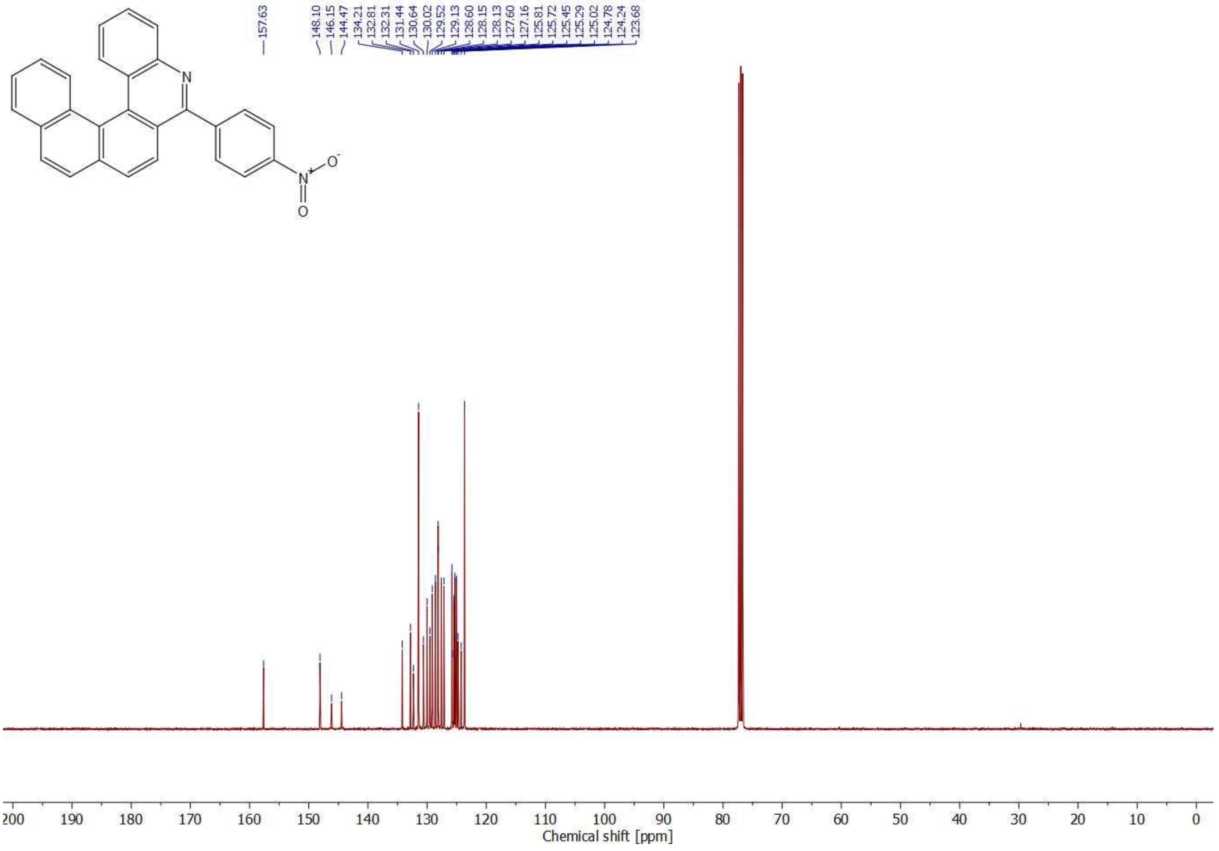

Figure S105. ^{13}C {^{1}H} NMR spectrum of compound **3j** (101 MHz, $CDCl_3$)

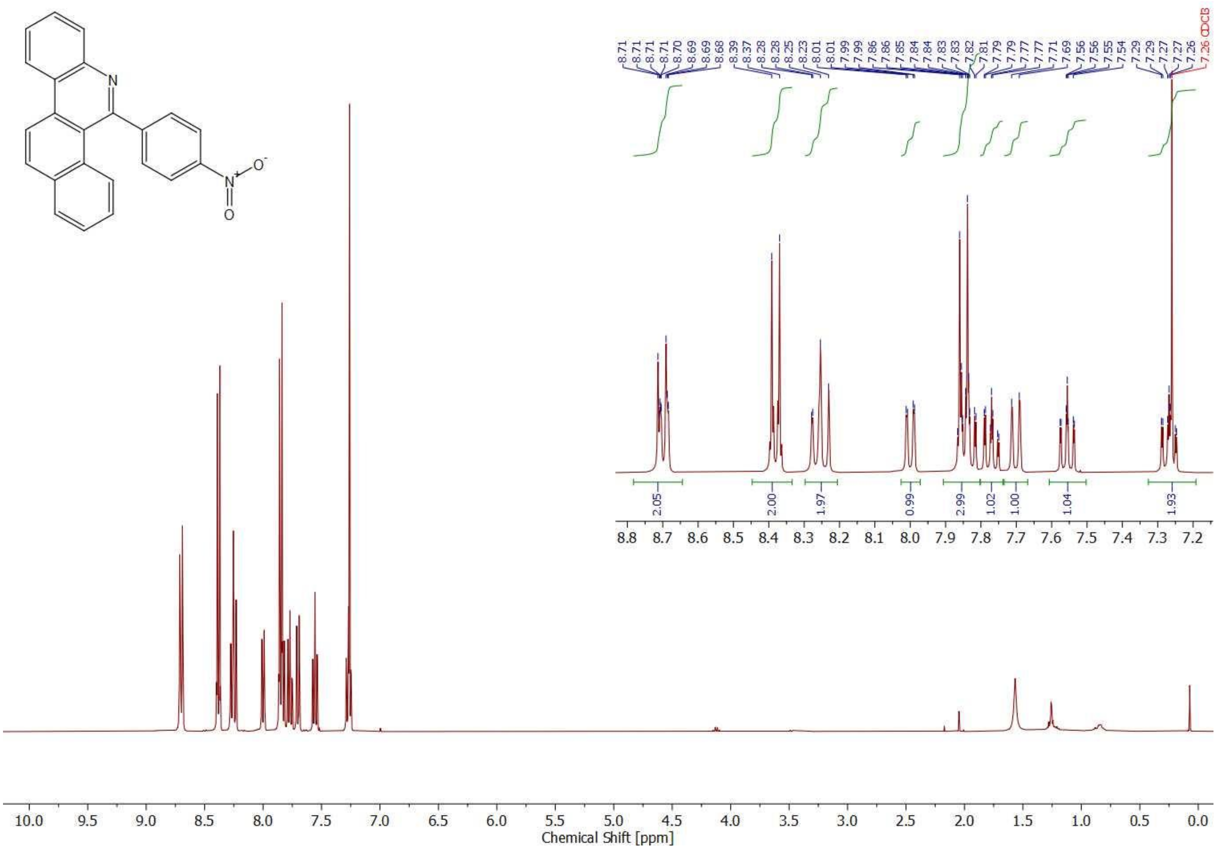

Figure S106. ^{1}H NMR spectrum of compound **3l** (400 MHz, $CDCl_3$)

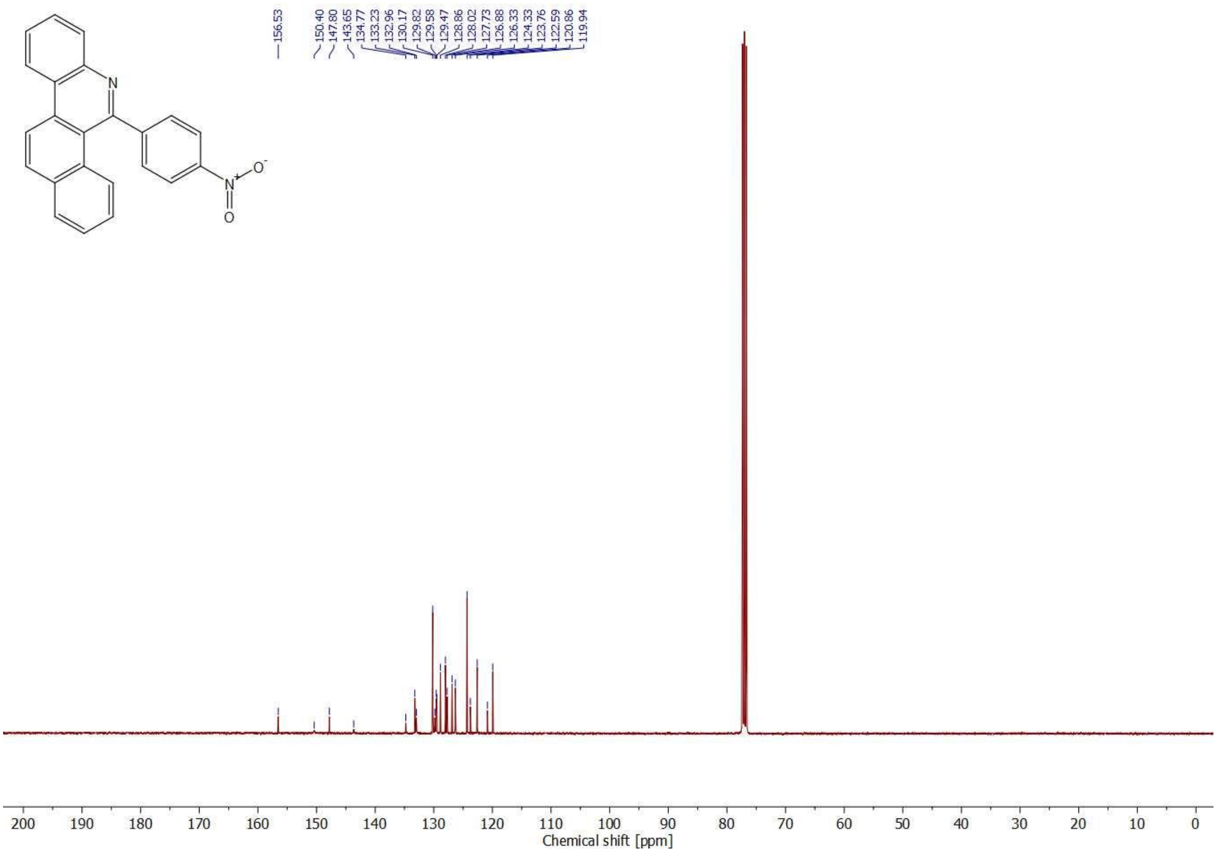

Figure S107. ^{13}C {^{1}H} NMR spectrum of compound **3l** (101 MHz, $CDCl_3$)

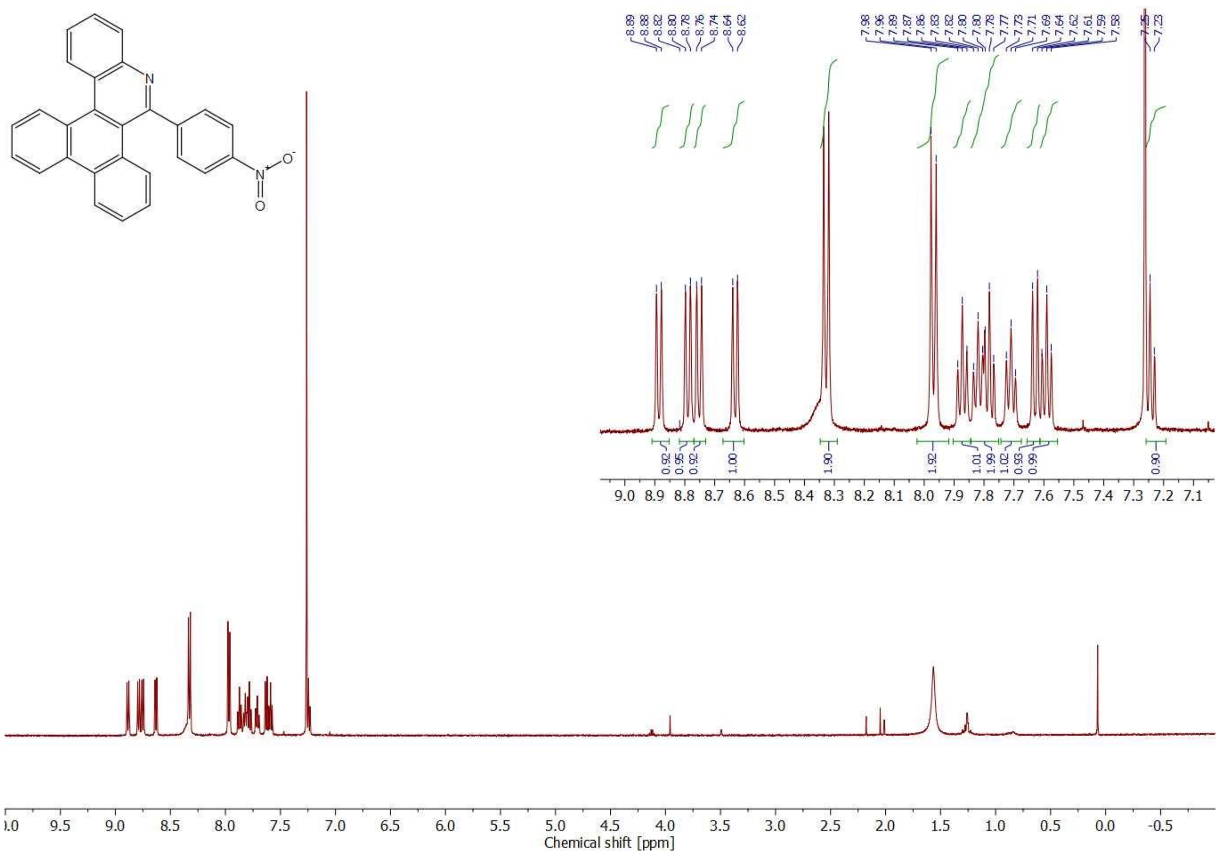

Figure S108. ^{1}H NMR spectrum of compound **3m** (500 MHz, $CDCl_3$)

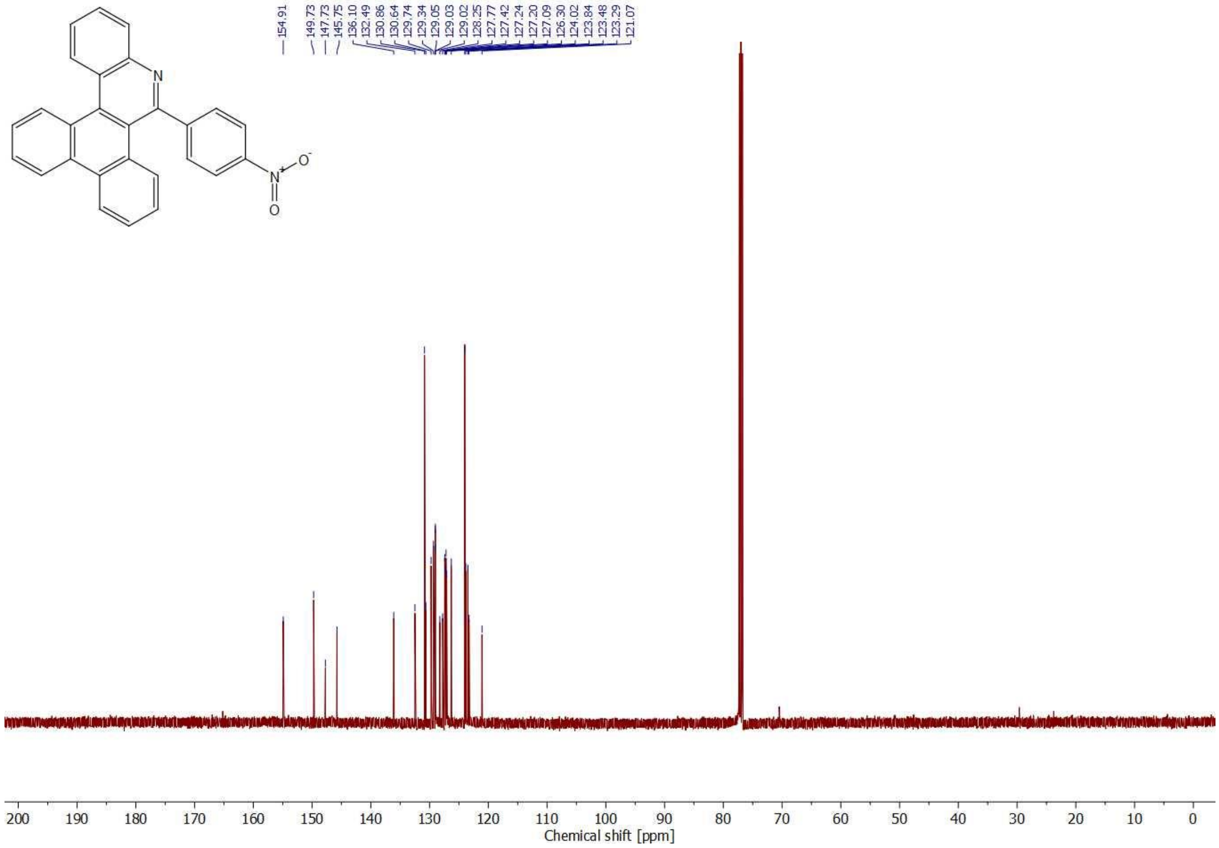

Figure S109. ^{13}C {^{1}H} NMR spectrum of compound **3m** (126 MHz, $CDCl_3$)

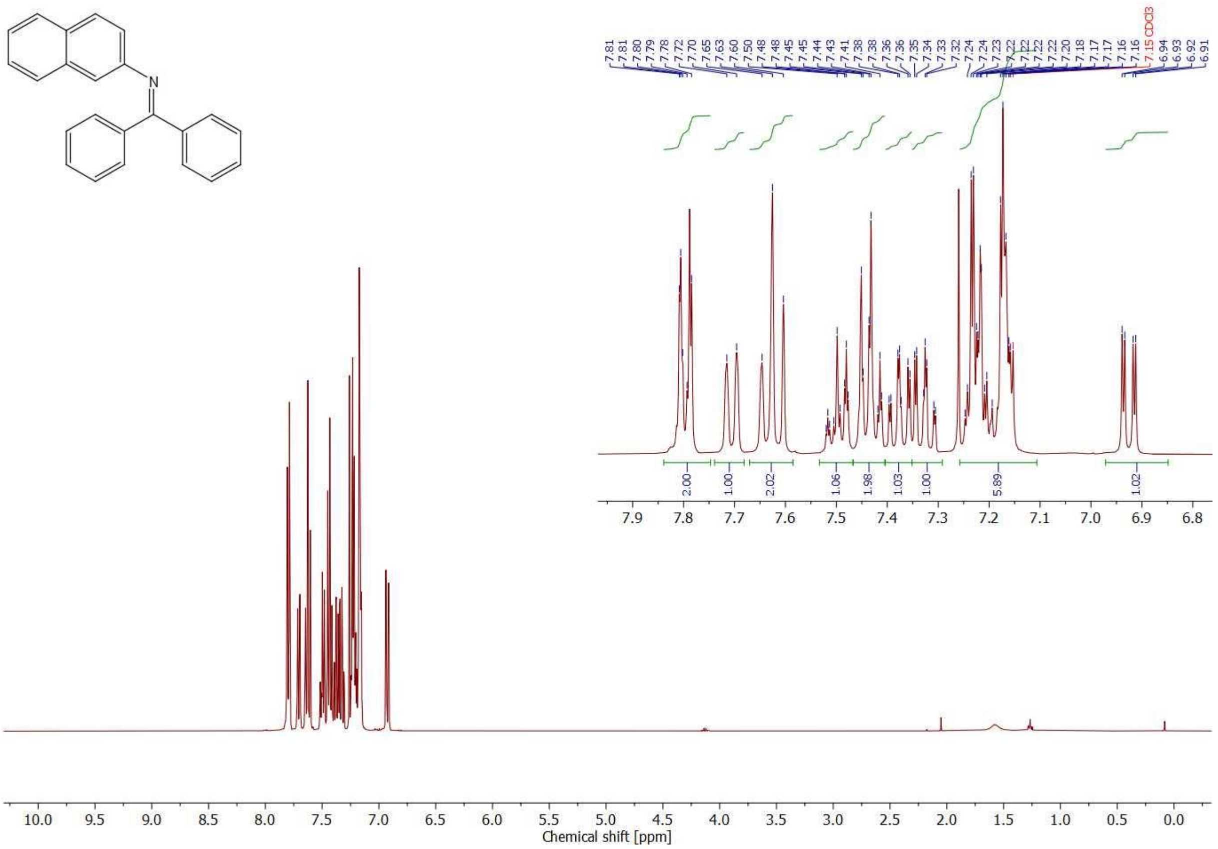

Figure S110. ^{1}H NMR spectrum of compound **1n** (400 MHz, $CDCl_3$)

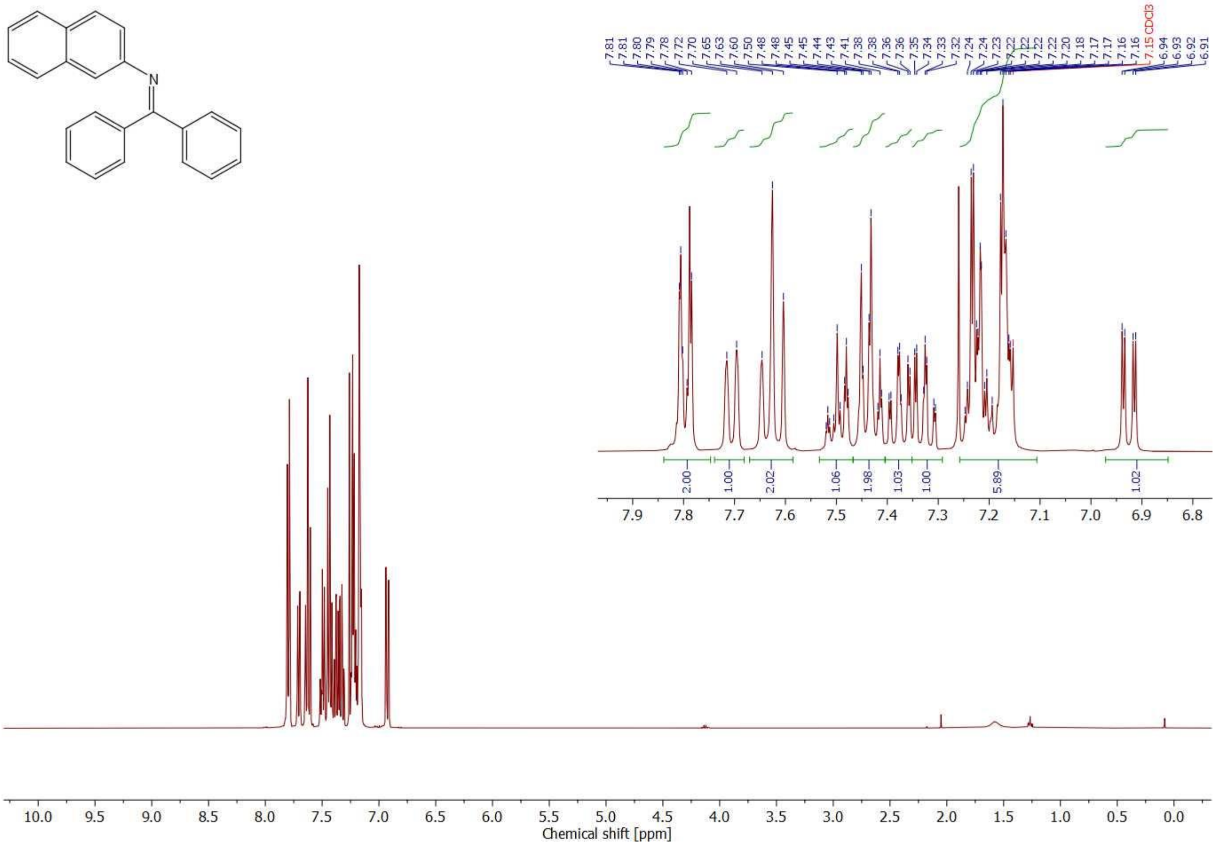

Figure S111. ^{13}C {^{1}H} NMR spectrum of compound **1n** (101 MHz, $CDCl_3$)

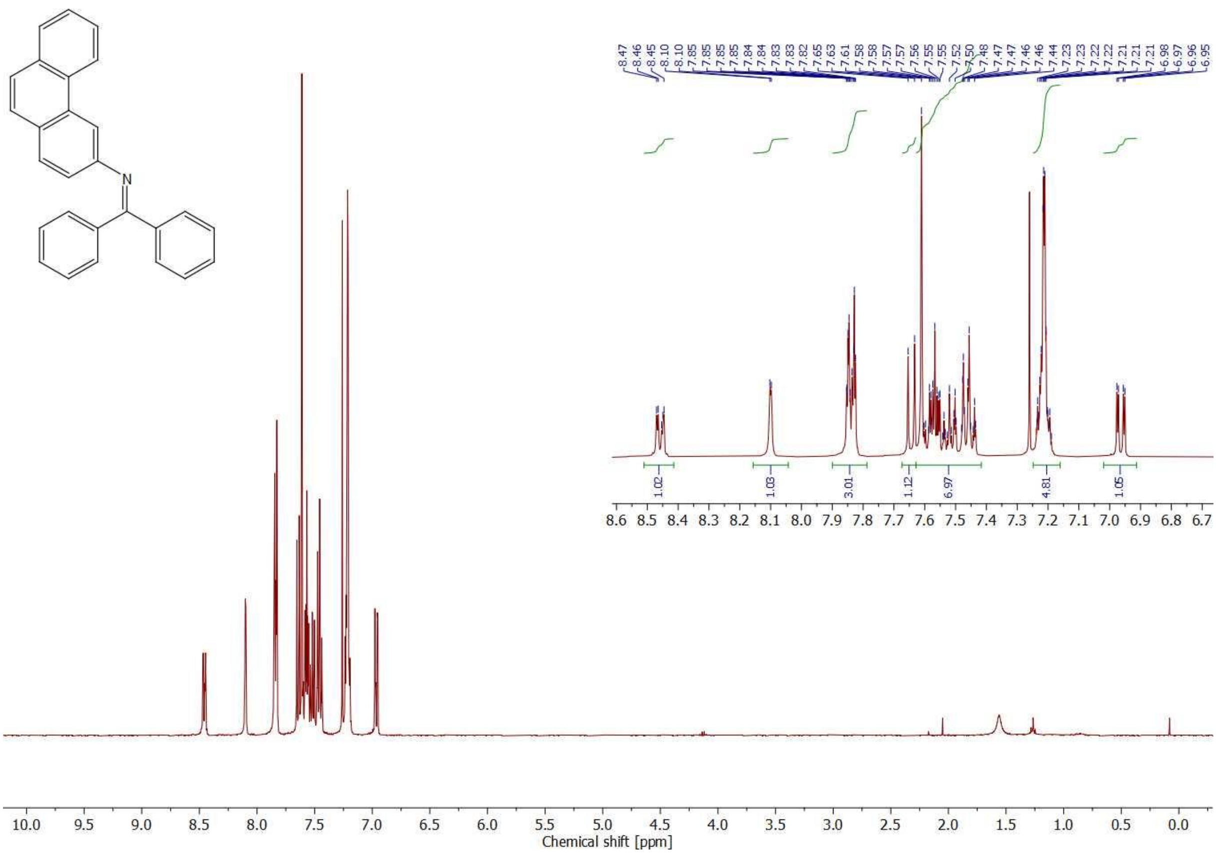

Figure S112. ^{1}H NMR spectrum of compound **1o** (400 MHz, $CDCl_3$)

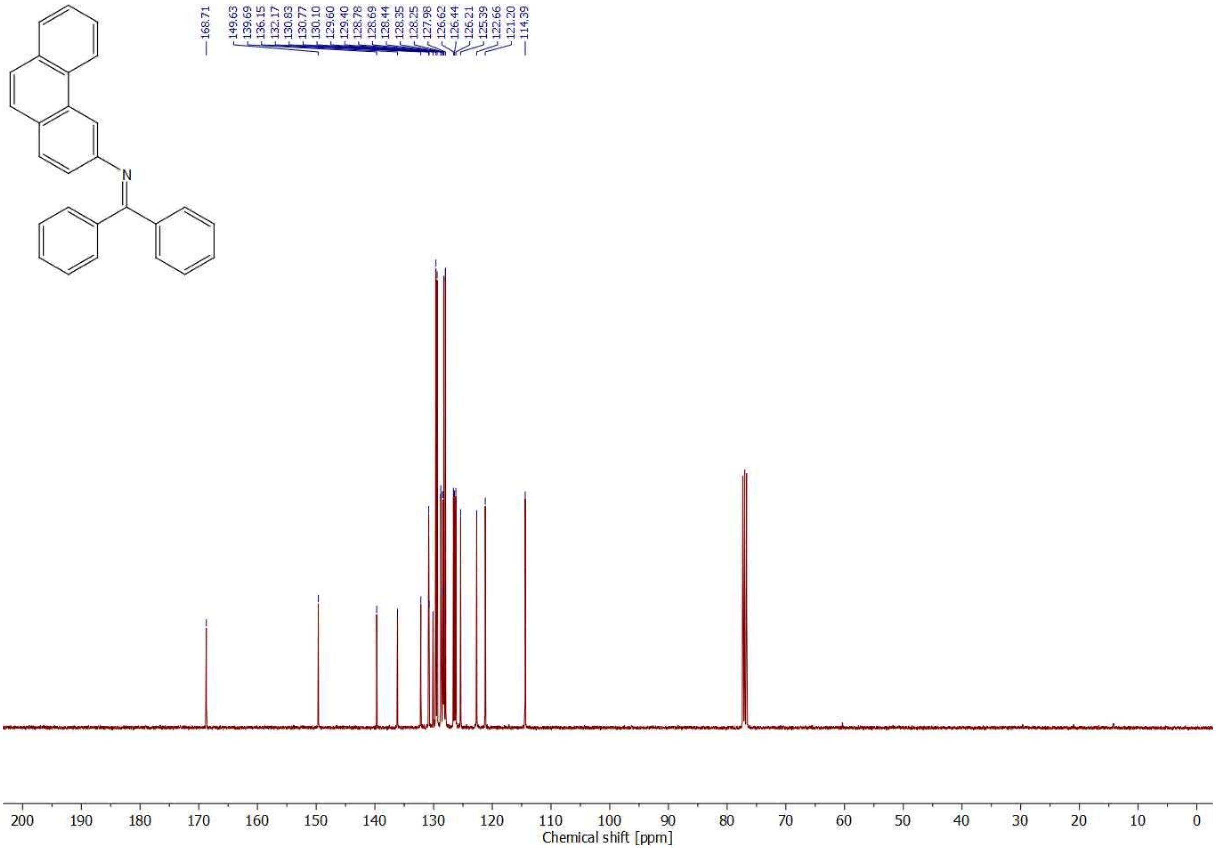

Figure S113. $^{13}C\{^{1}H\}$ NMR spectrum of compound **1o** (101 MHz, $CDCl_3$)

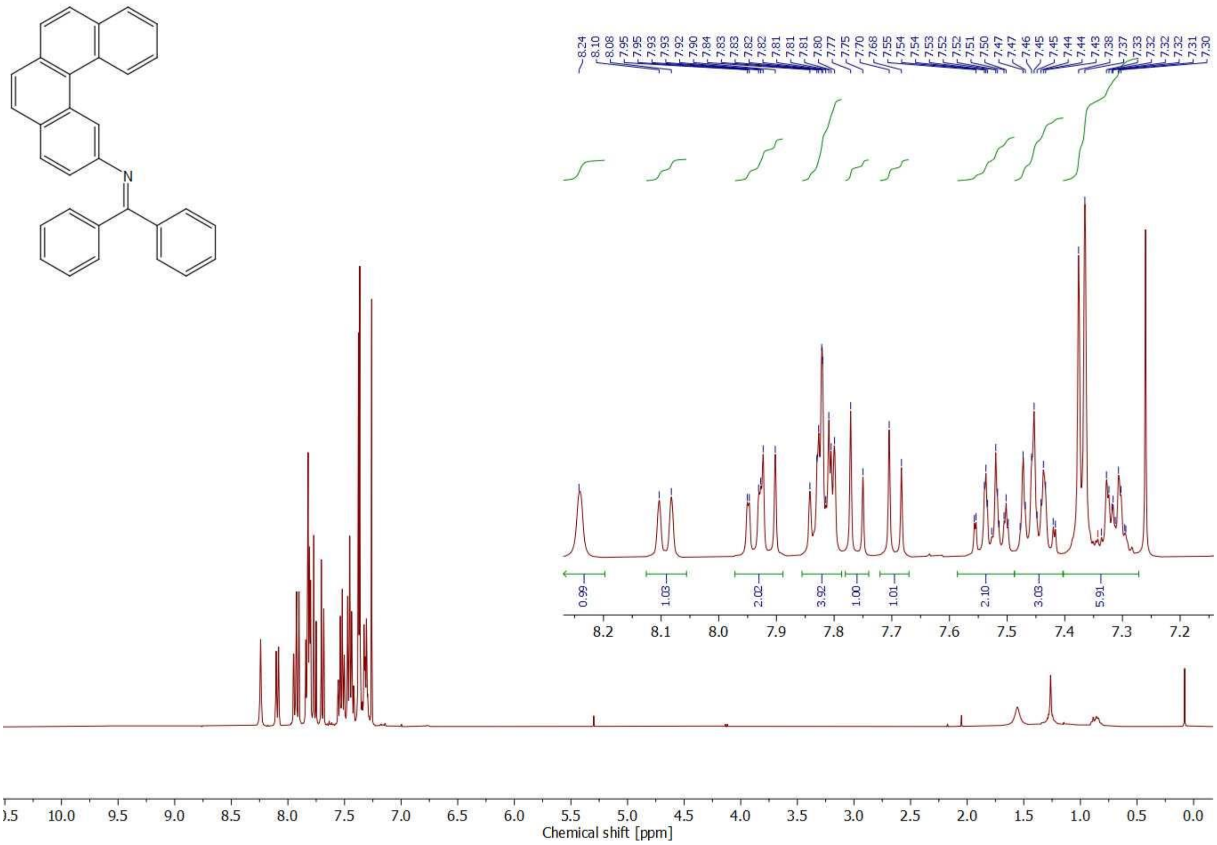

Figure S114. ^{1}H NMR spectrum of compound **1p** (400 MHz, $CDCl_3$)

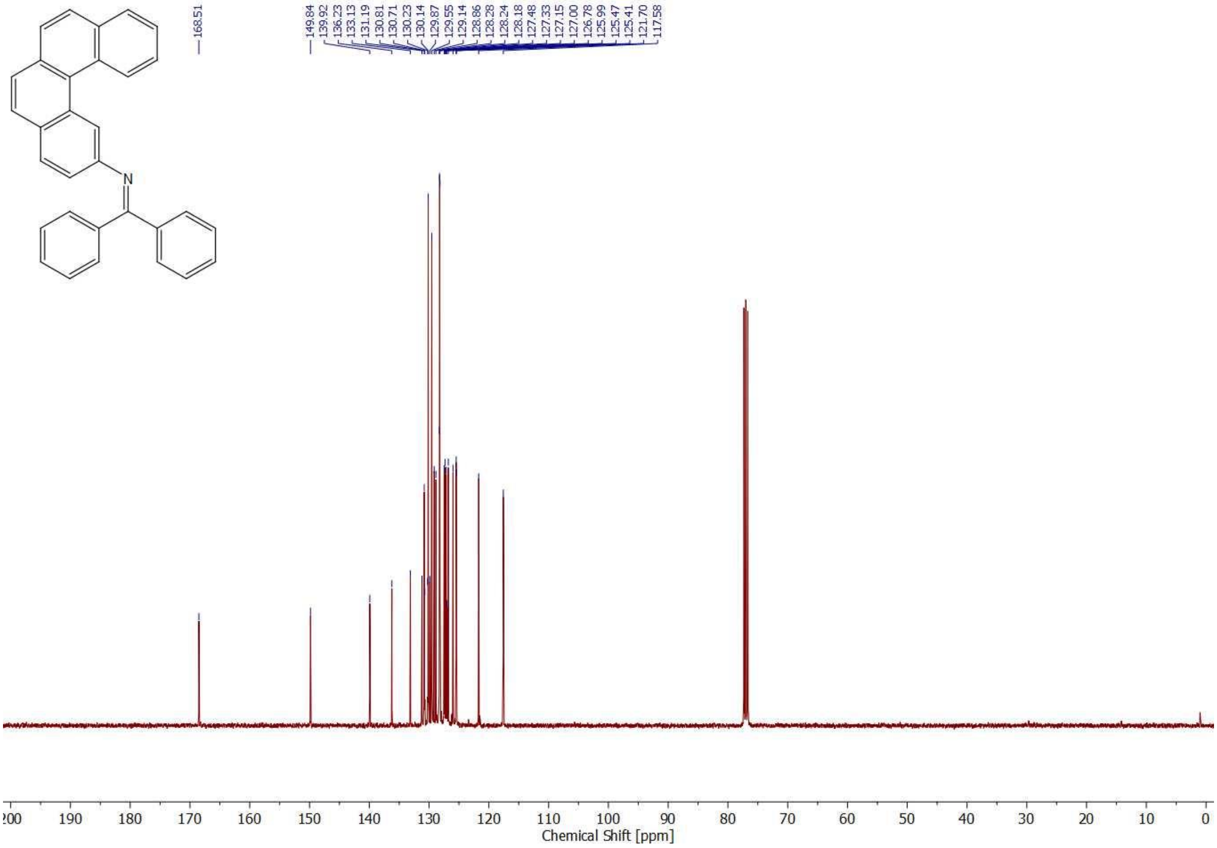

Figure S115. ^{13}C {^{1}H} NMR spectrum of compound **1p** (101 MHz, $CDCl_3$)

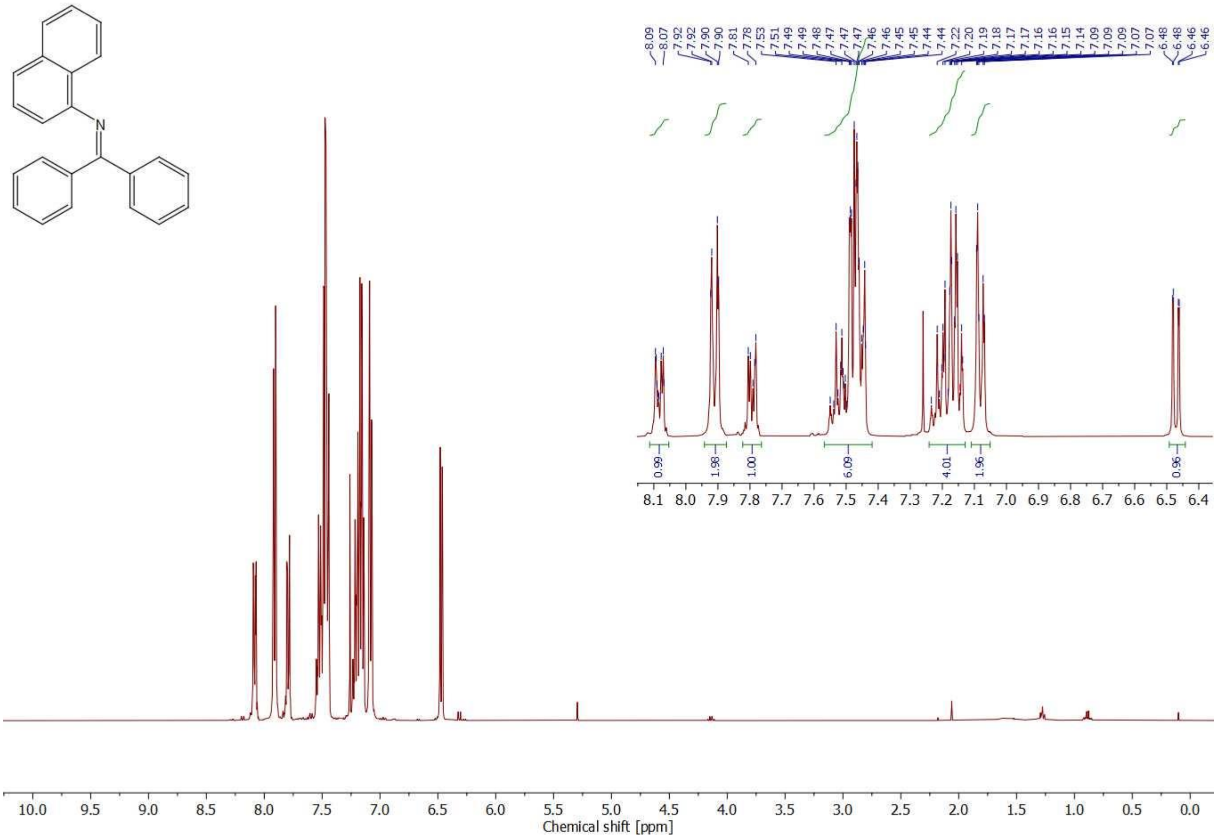

Figure S116. ^{1}H NMR spectrum of compound **1q** (400 MHz, $CDCl_3$)

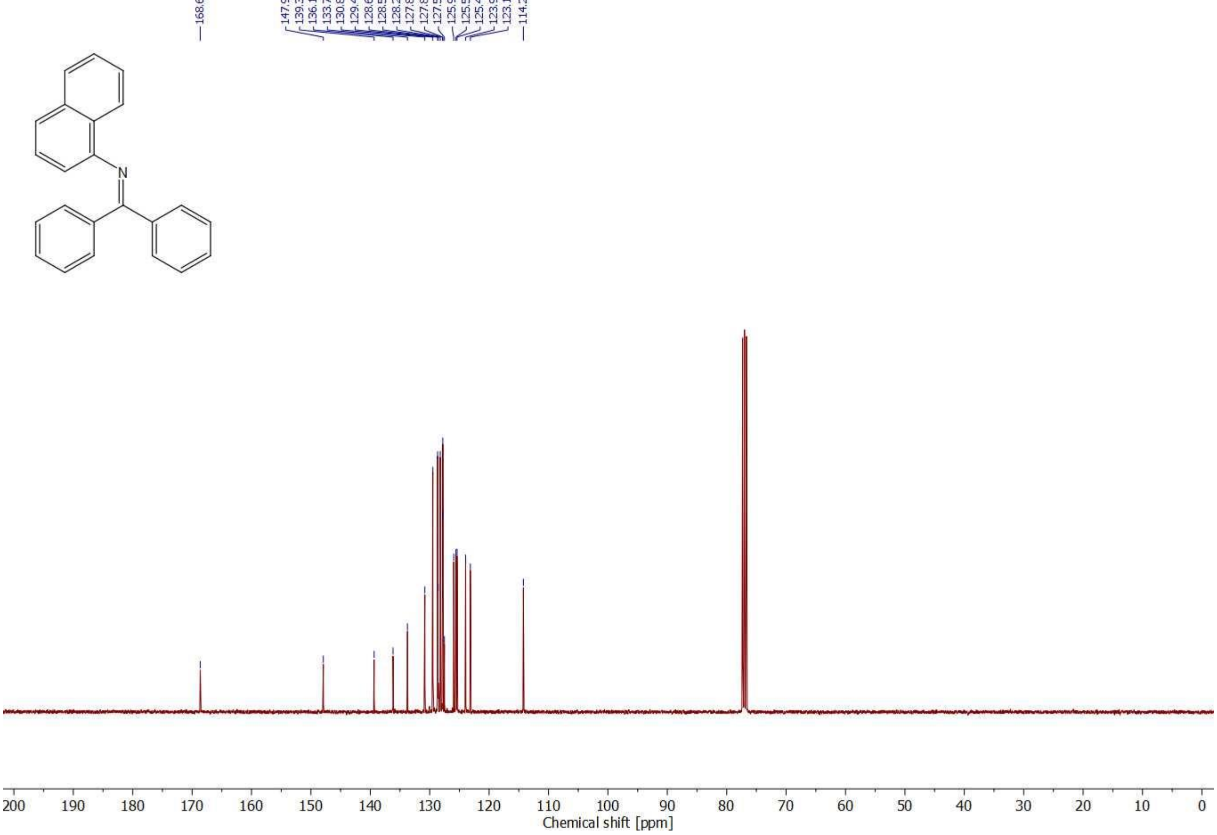

Figure S117. ^{13}C {^{1}H} NMR spectrum of compound **1q** (101 MHz, $CDCl_3$)

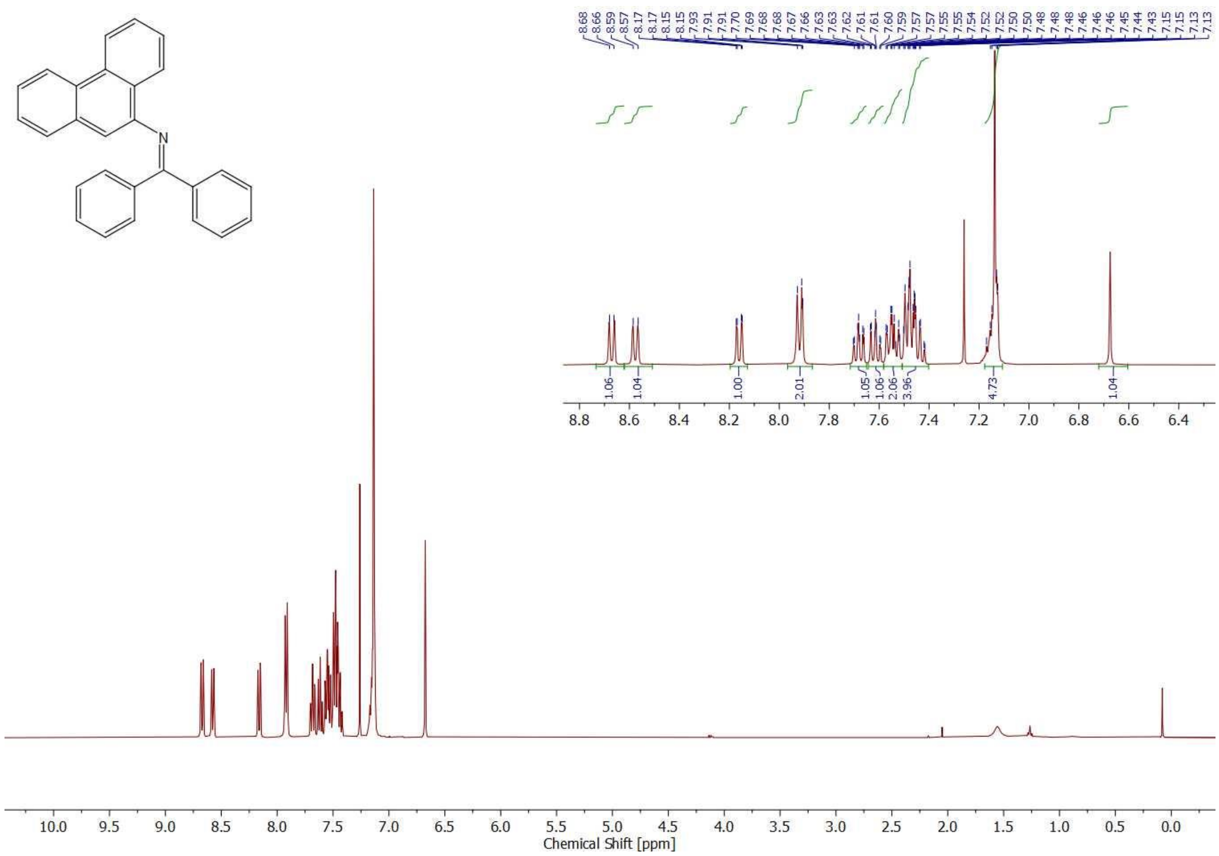

Figure S118. ^{1}H NMR spectrum of compound **1r** (400 MHz, $CDCl_3$)

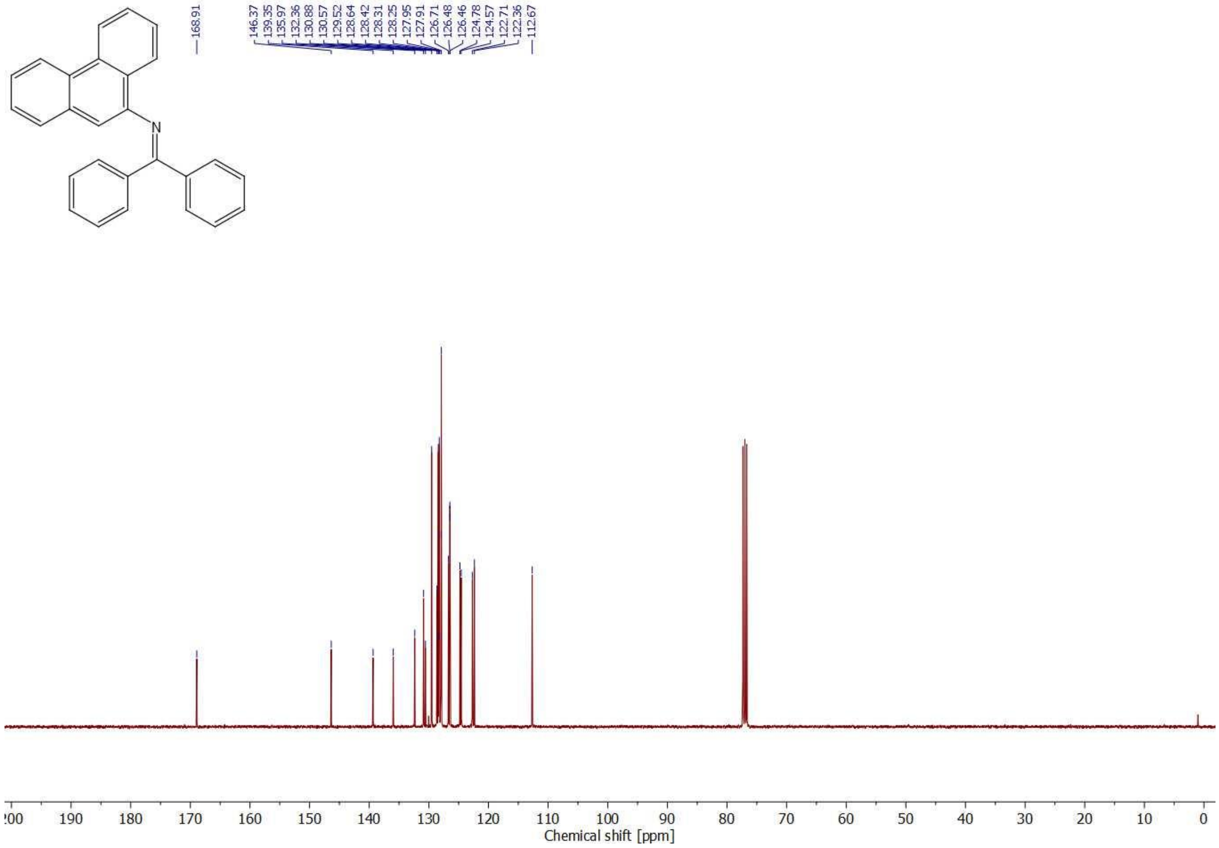

Figure S119. ^{13}C {^{1}H} NMR spectrum of compound **1r** (101 MHz, $CDCl_3$)

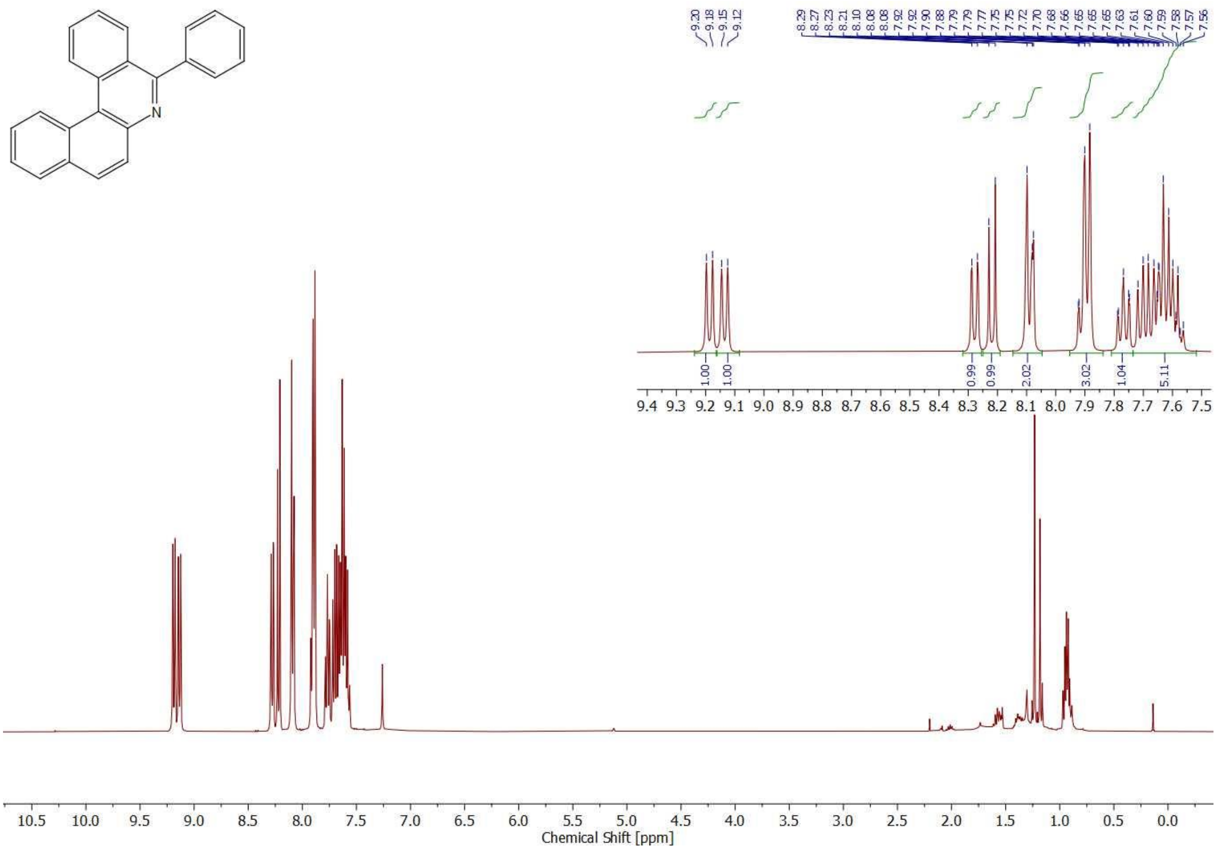

Figure S120. ^{1}H NMR spectrum of compound **3n** (400 MHz, $CDCl_3$)

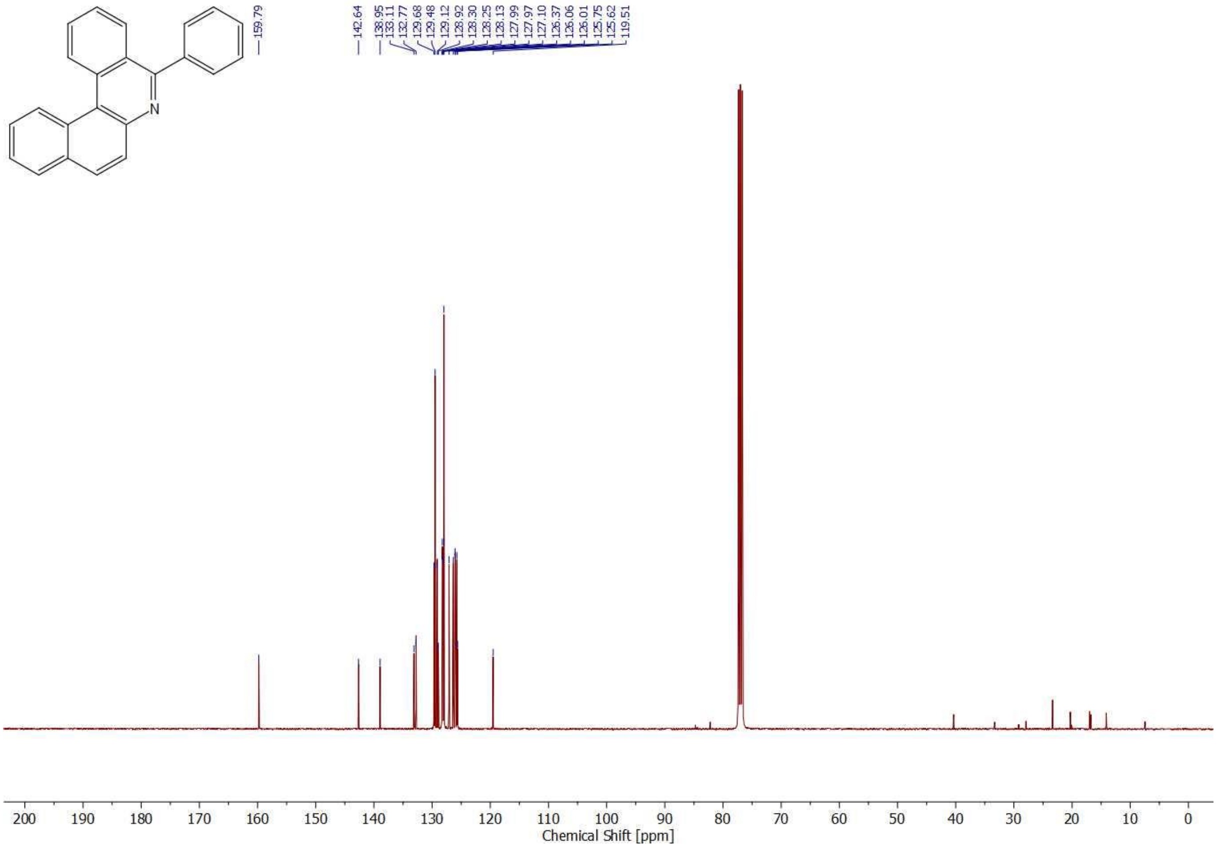

Figure S121. ^{13}C {^{1}H} NMR spectrum of compound **3n** (101 MHz, $CDCl_3$)

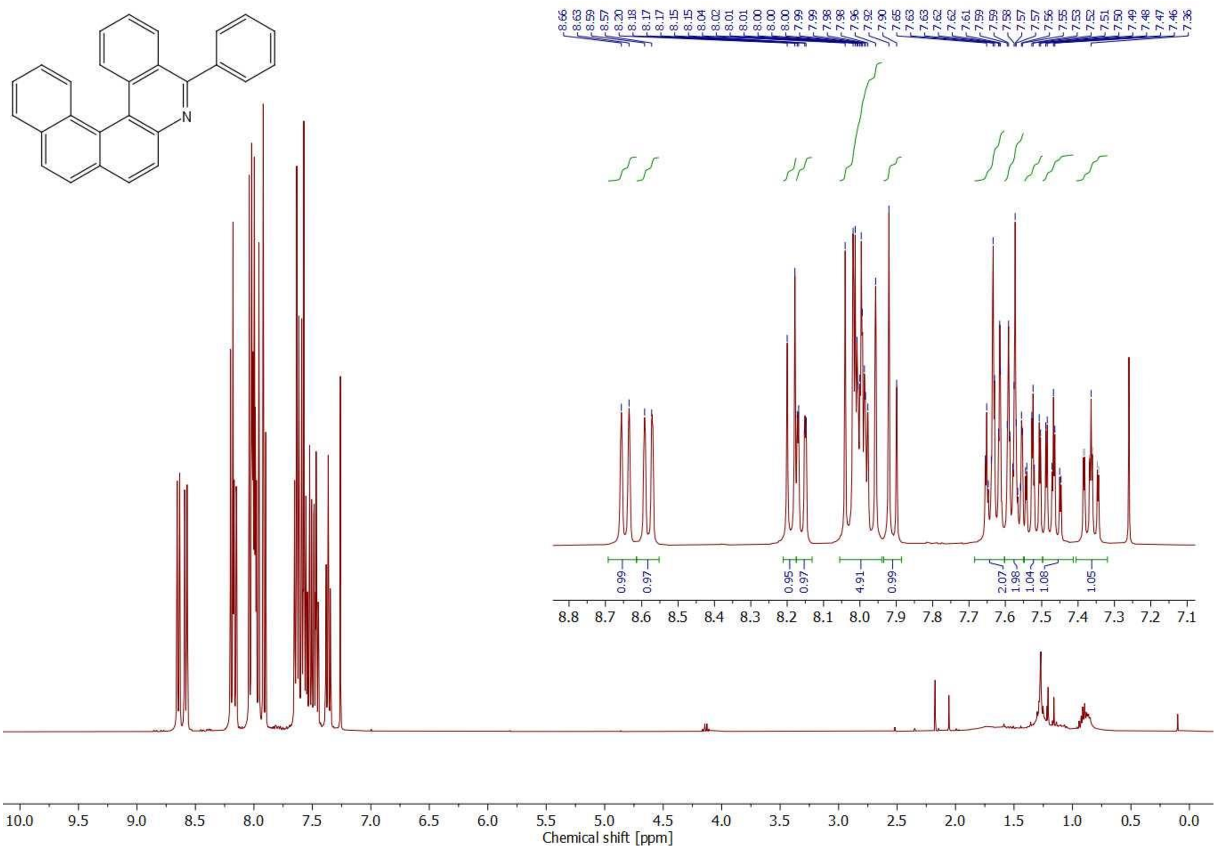

Figure S122. ^{1}H NMR spectrum of compound **3o** (400 MHz, $CDCl_3$)

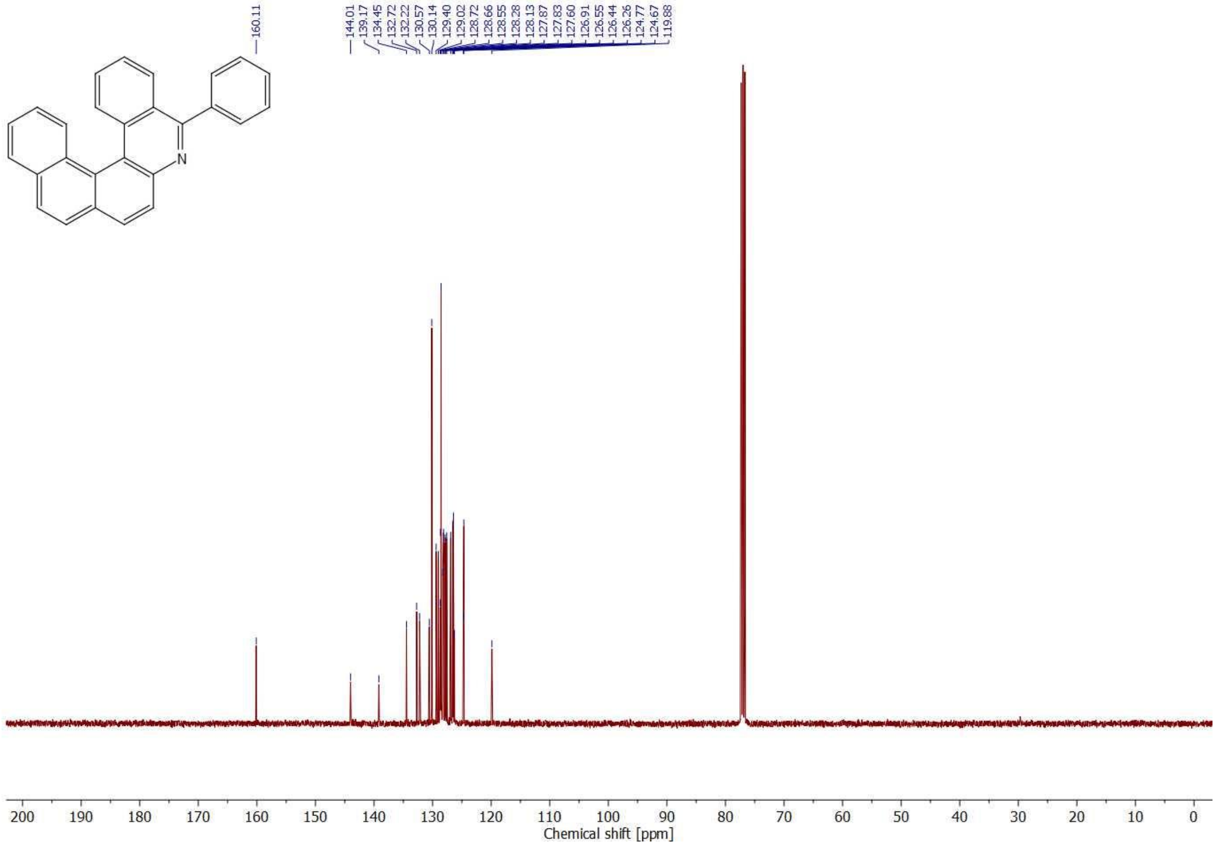

Figure S123. ^{13}C {^{1}H} NMR spectrum of compound **3o** (101 MHz, $CDCl_3$)

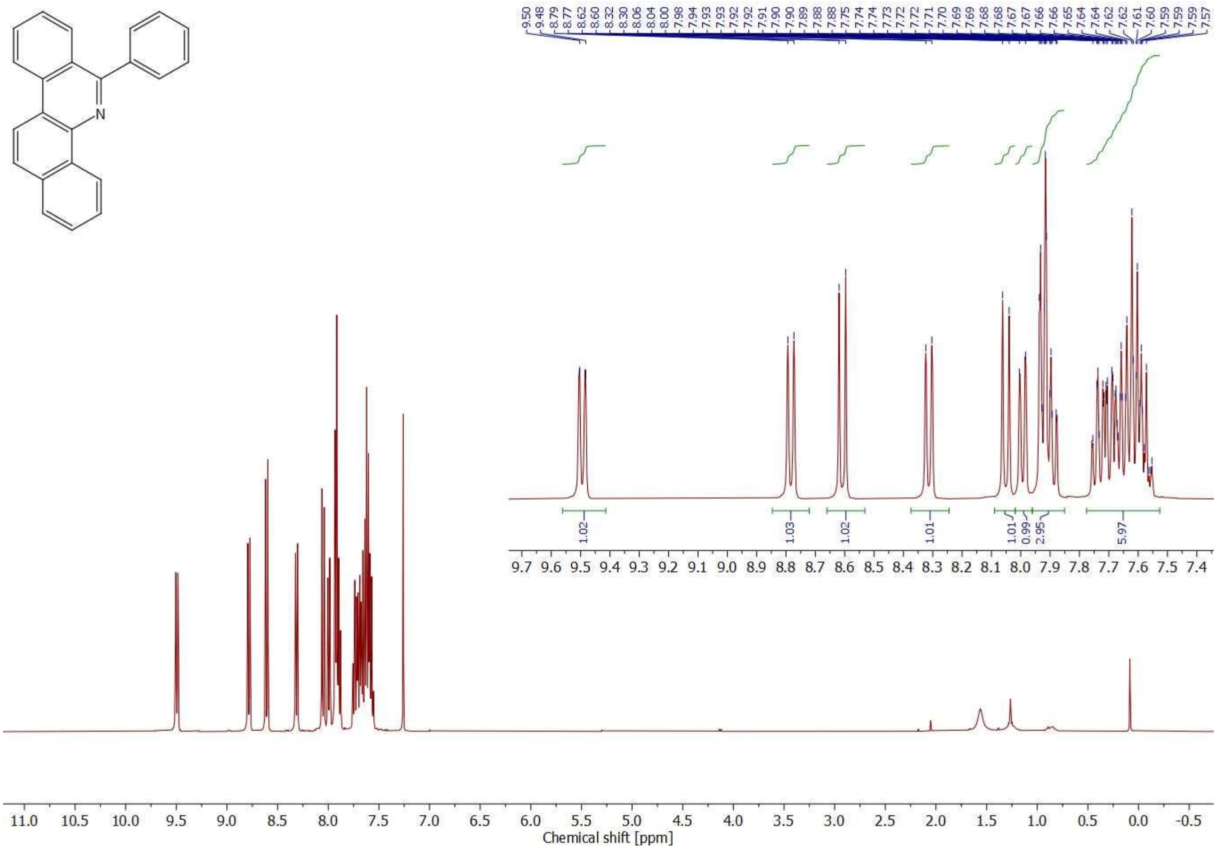

Figure S124. ^{1}H NMR spectrum of compound **3q** (400 MHz, $CDCl_3$)

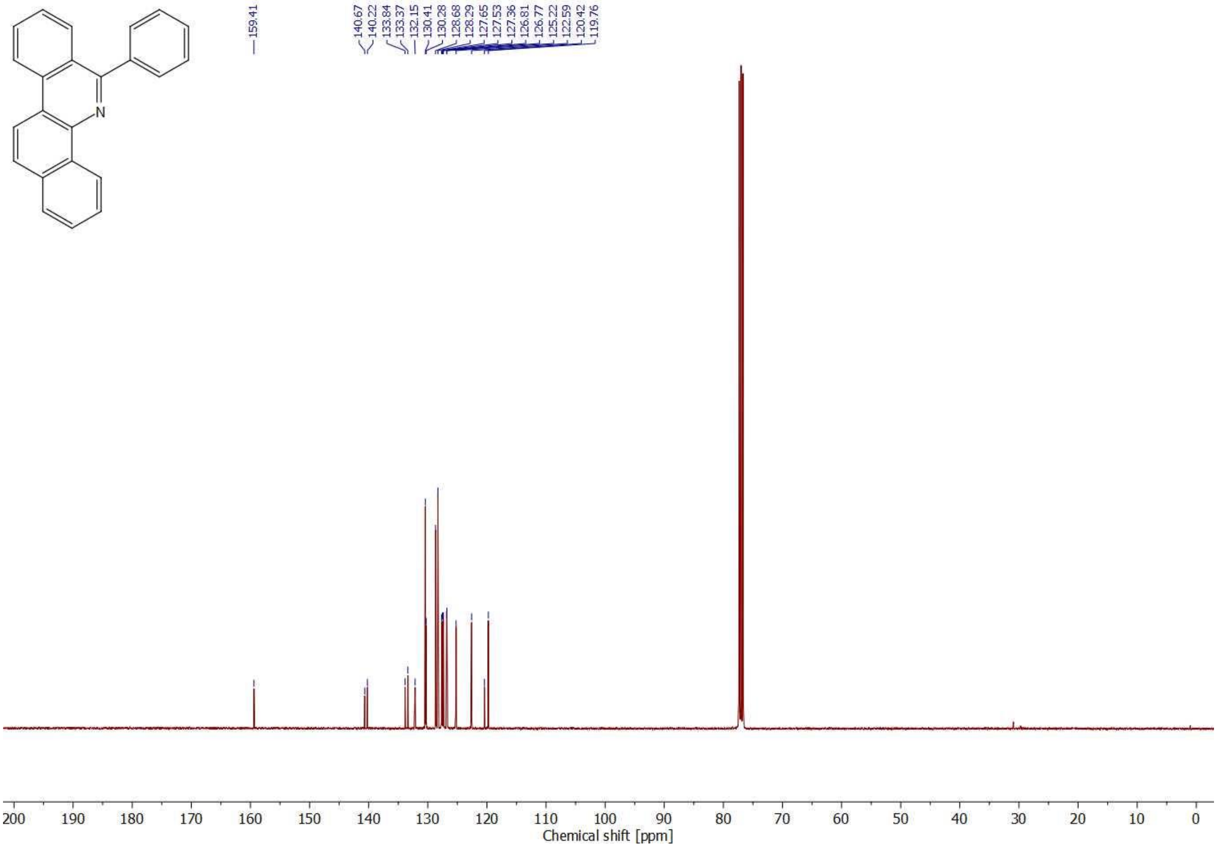

Figure S125. ^{13}C {^{1}H} NMR spectrum of compound **3q** (101 MHz, $CDCl_3$)

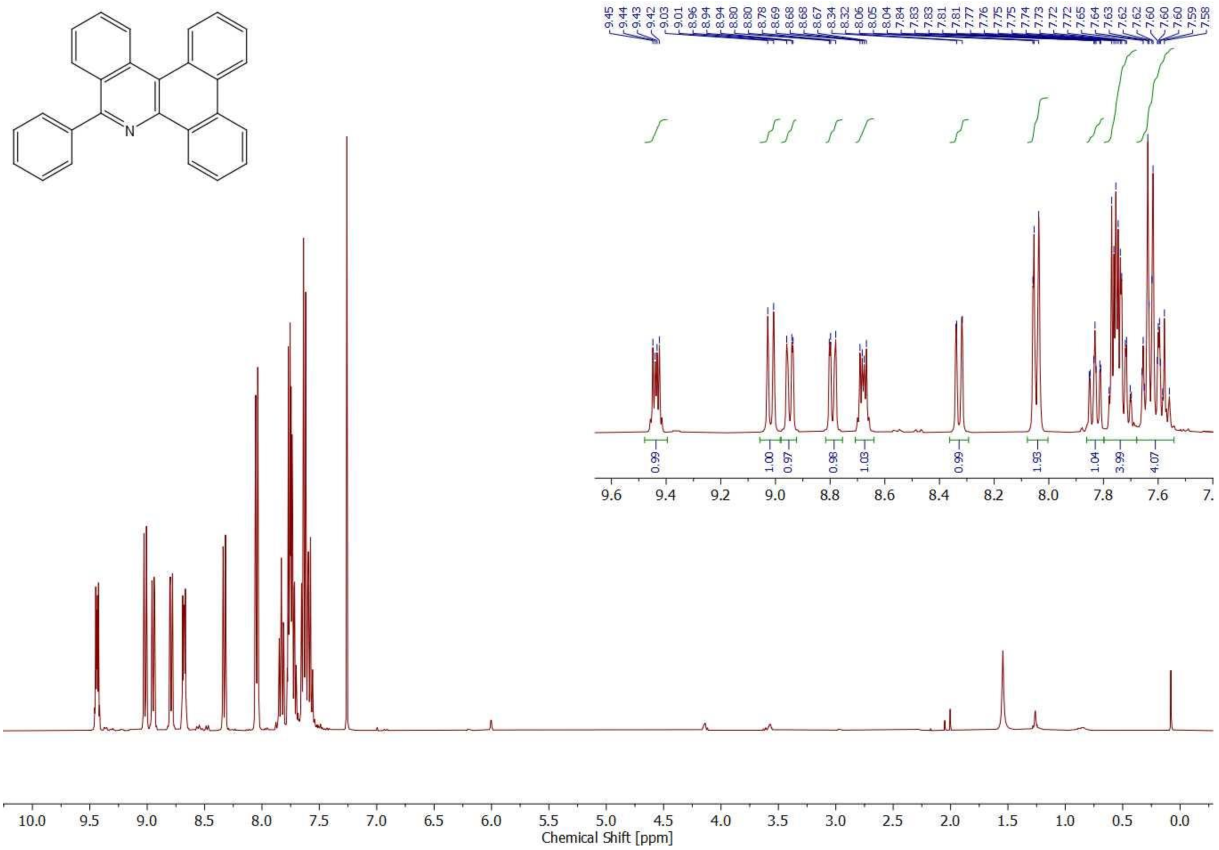

Figure S126. ^{1}H NMR spectrum of compound **3r** (400 MHz, $CDCl_3$)

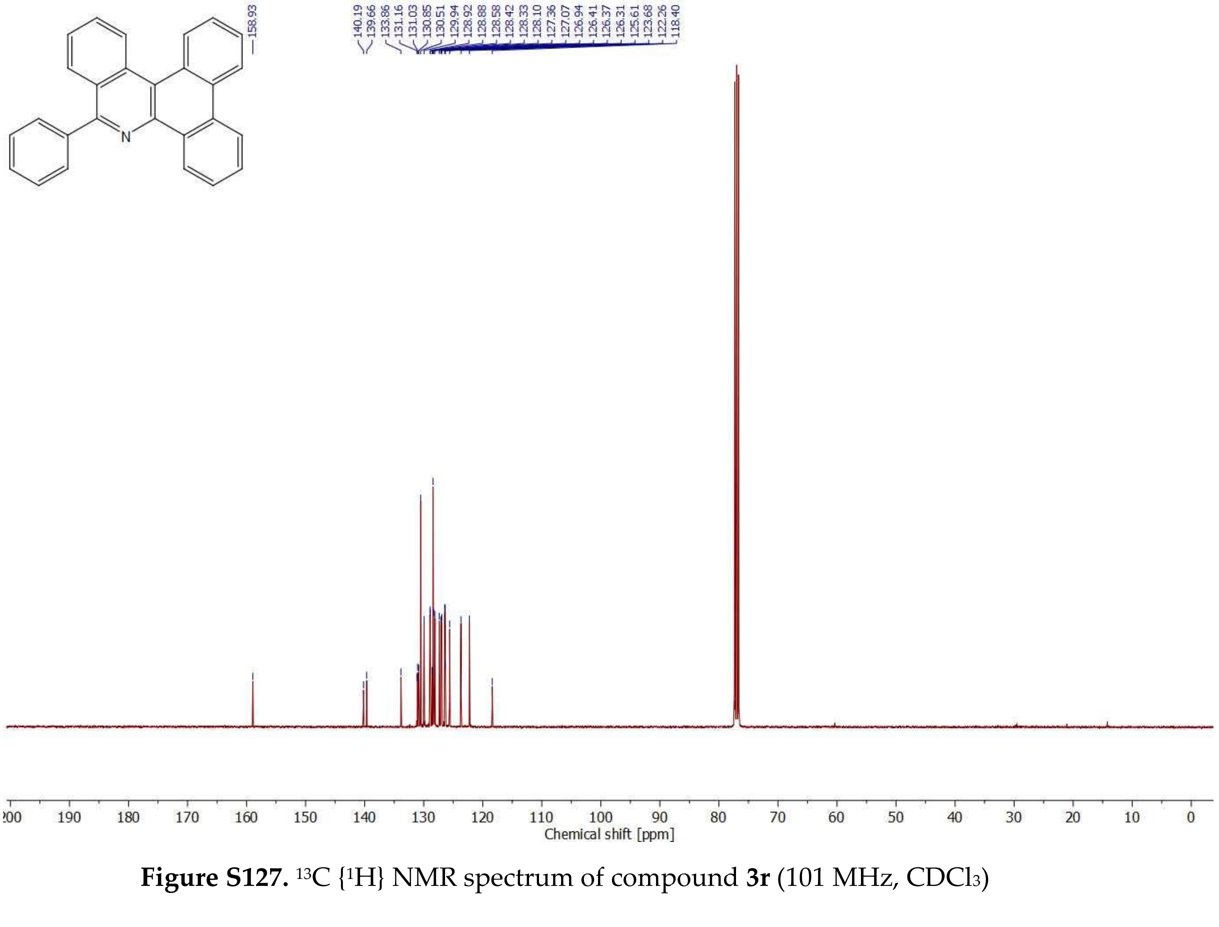

Figure S127. ^{13}C {^{1}H} NMR spectrum of compound **3r** (101 MHz, $CDCl_3$)

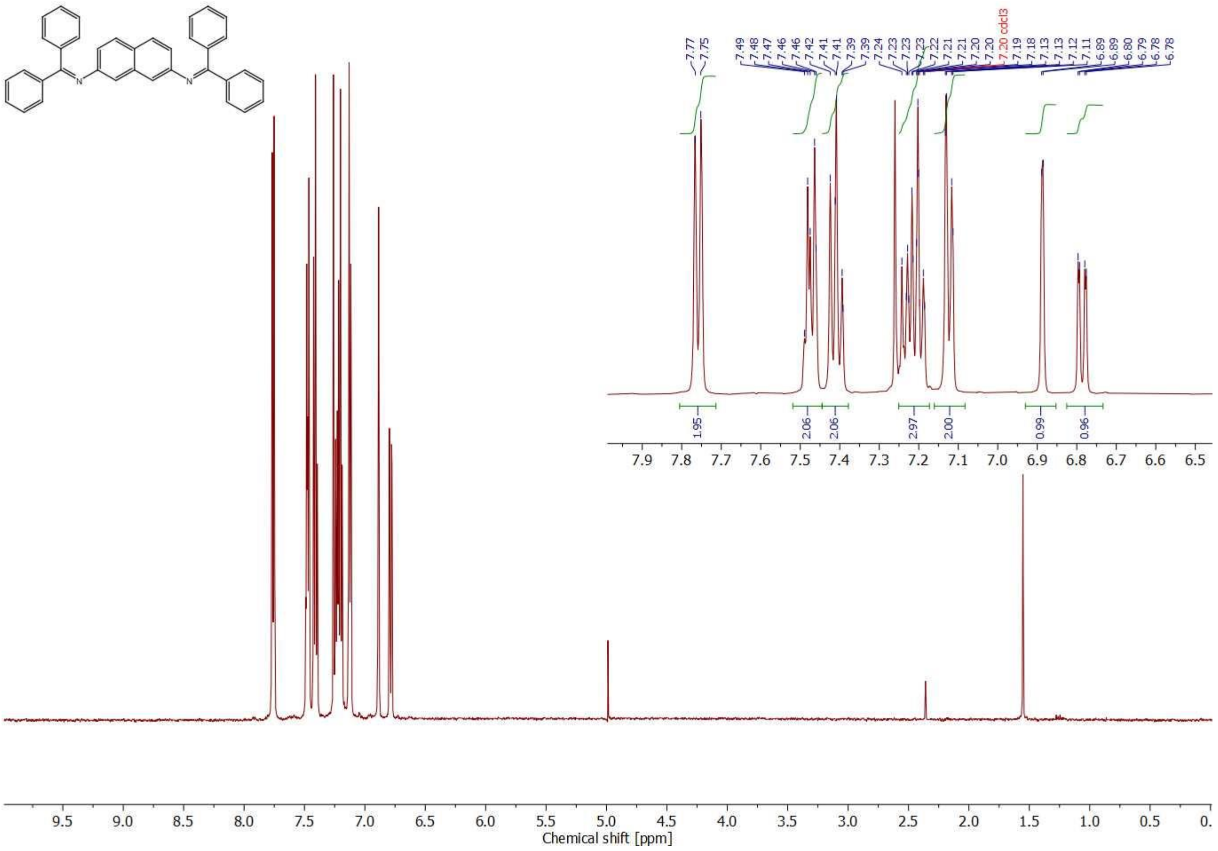

Figure S128. ^{1}H NMR spectrum of compound **9a** (500 MHz, $CDCl_3$)

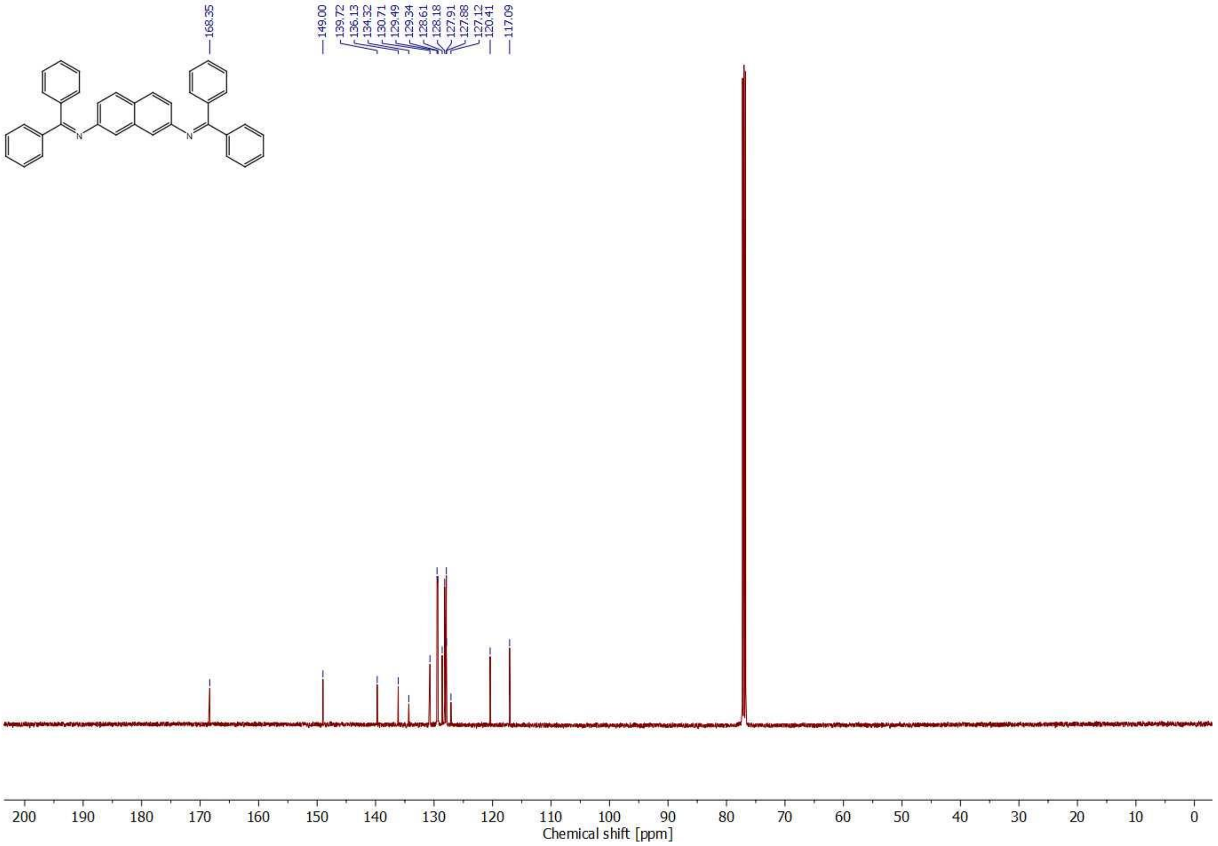

Figure S129. ^{13}C {^{1}H} NMR spectrum of compound **9a** (126 MHz, $CDCl_3$)

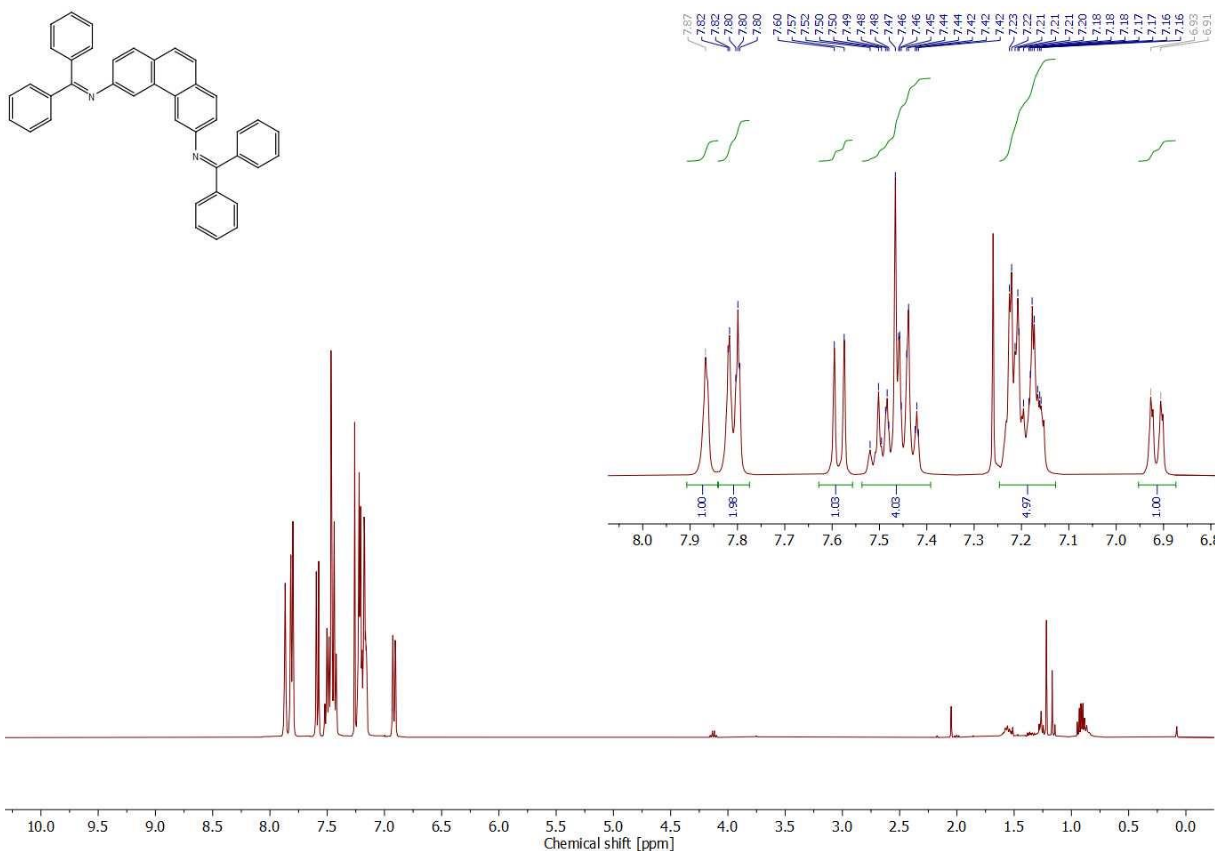

Figure S130. ^{1}H NMR spectrum of compound **9b** (400 MHz, $CDCl_3$)

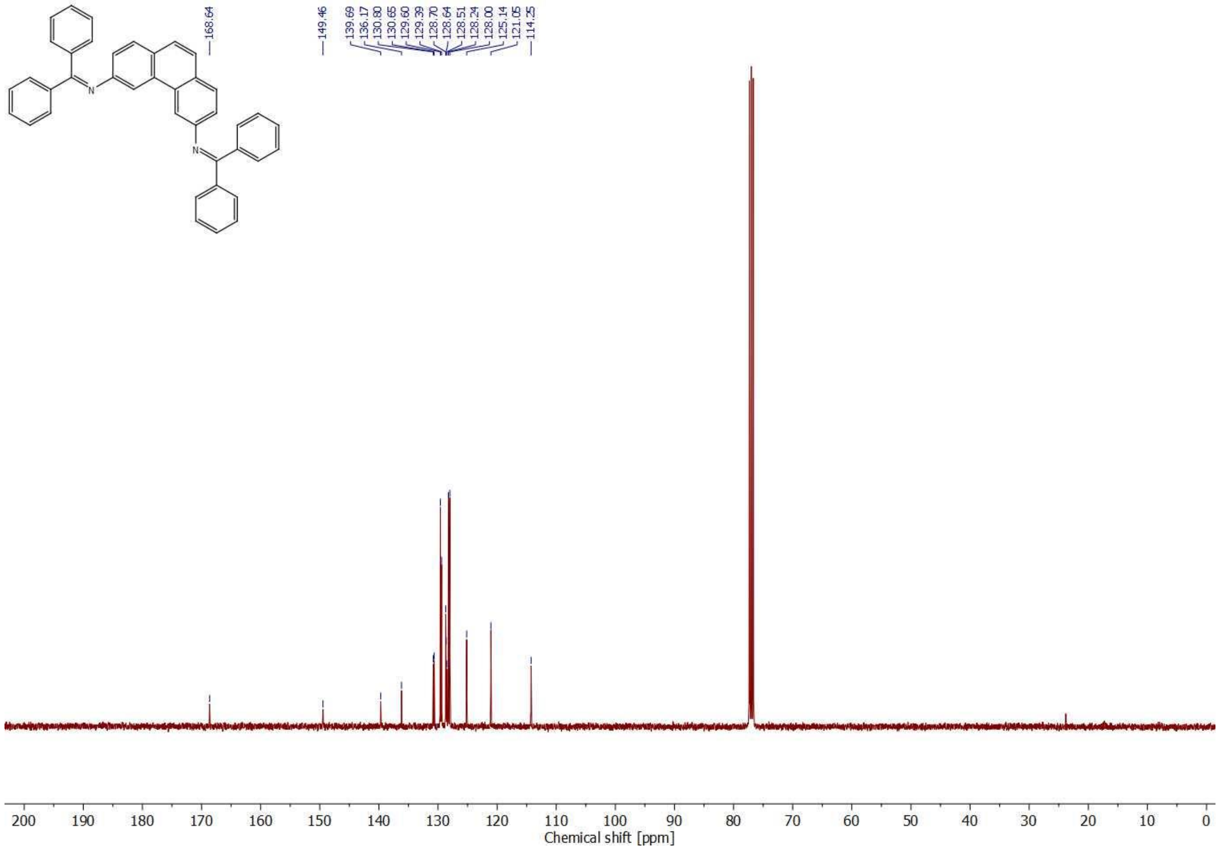

Figure S131. $^{13}C\{^1H\}$ NMR spectrum of compound **9b** (101 MHz, $CDCl_3$)

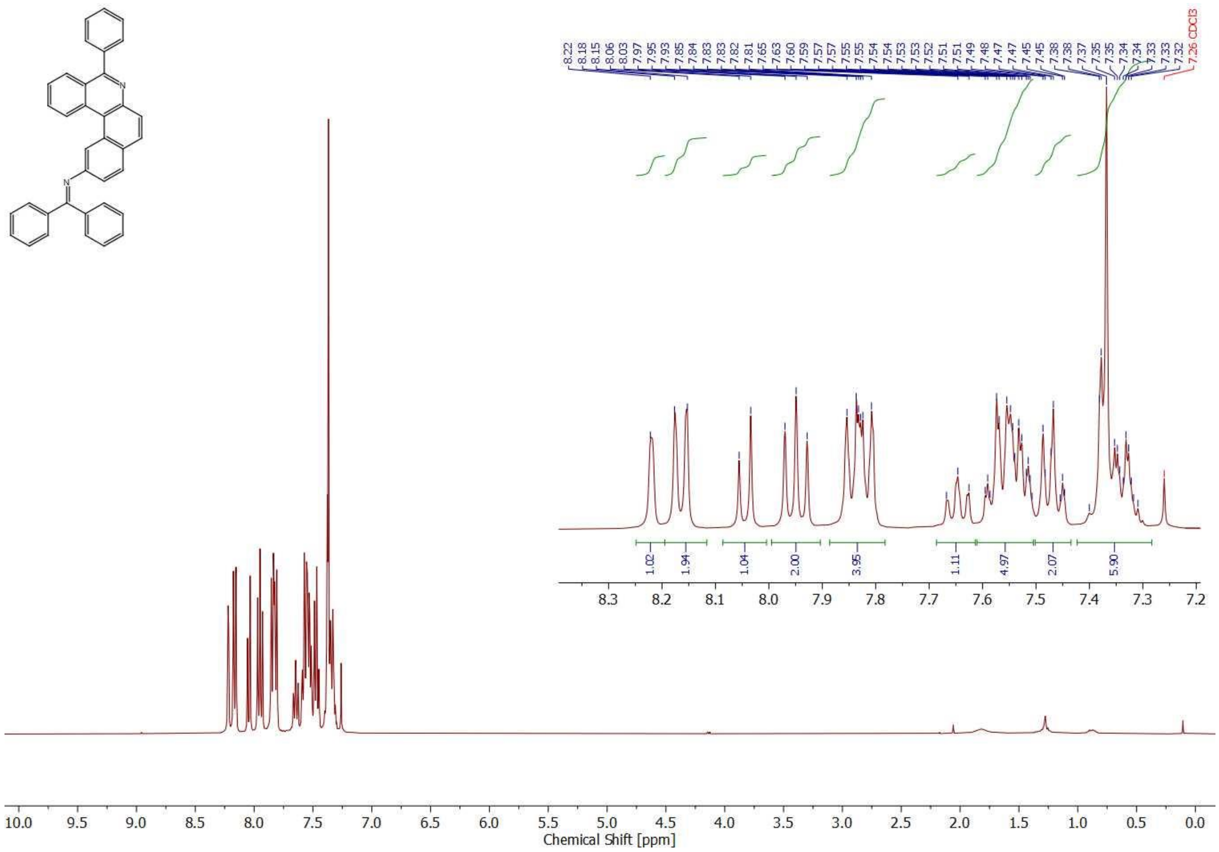

Figure S132. ^{1}H NMR spectrum of compound **10a** (500 MHz, $CDCl_3$)

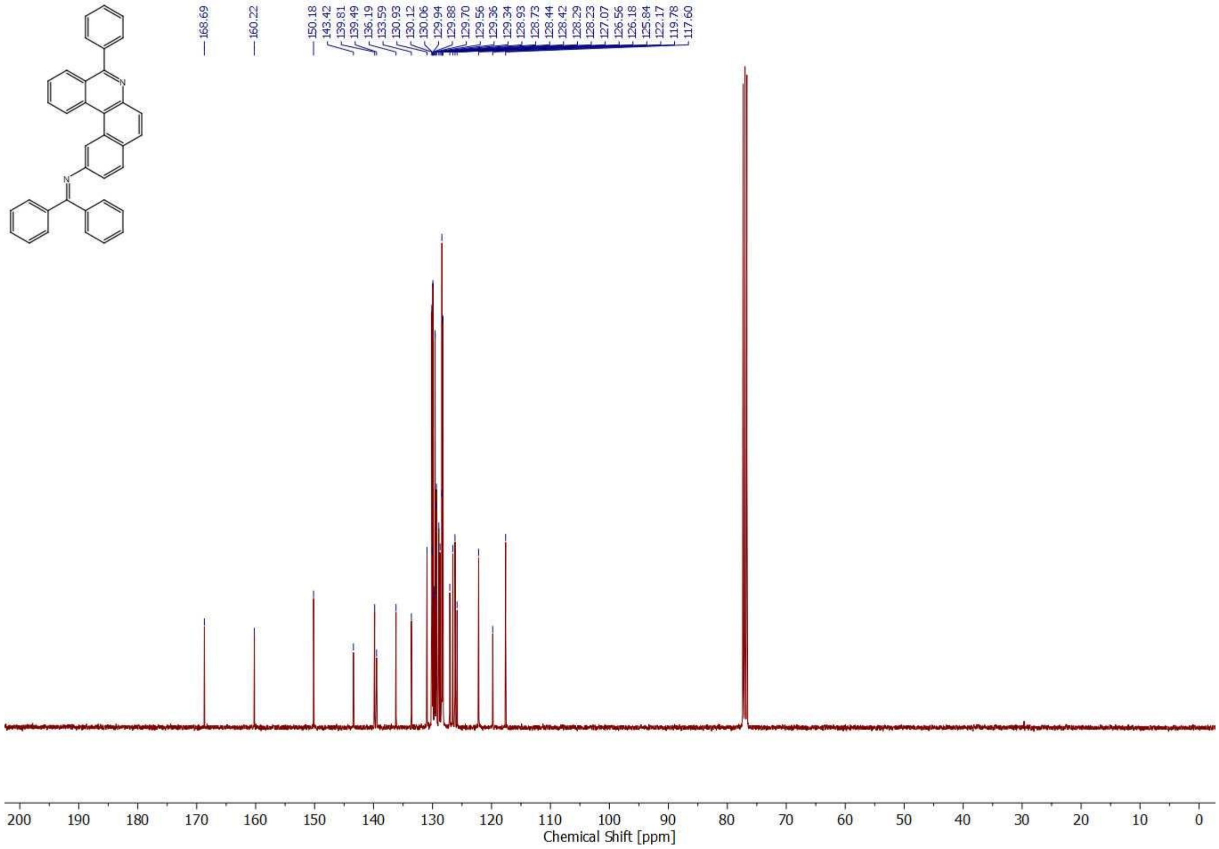

Figure S133. ^{13}C {^{1}H} NMR spectrum of compound **10a** (126 MHz, $CDCl_3$)

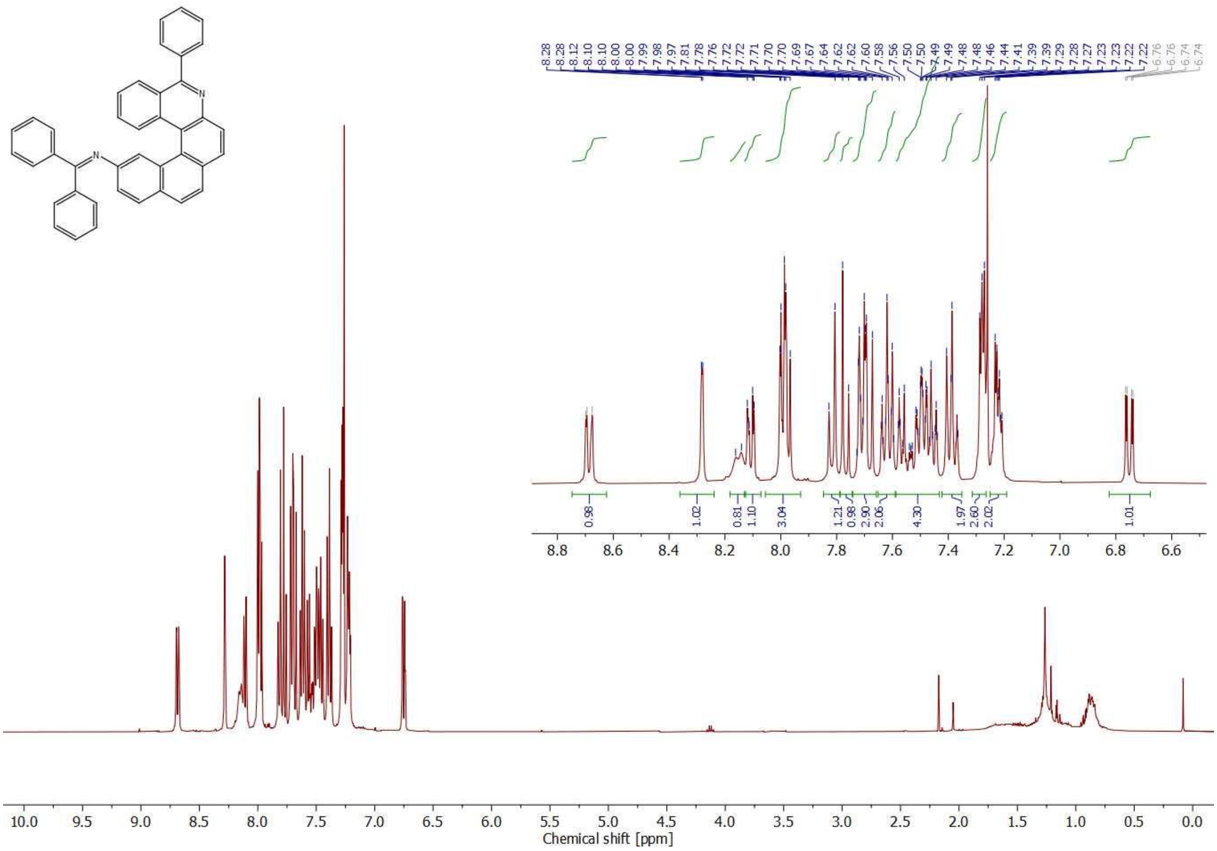

Figure S134. ^{1}H NMR spectrum of compound **10b** (400 MHz, $CDCl_3$)

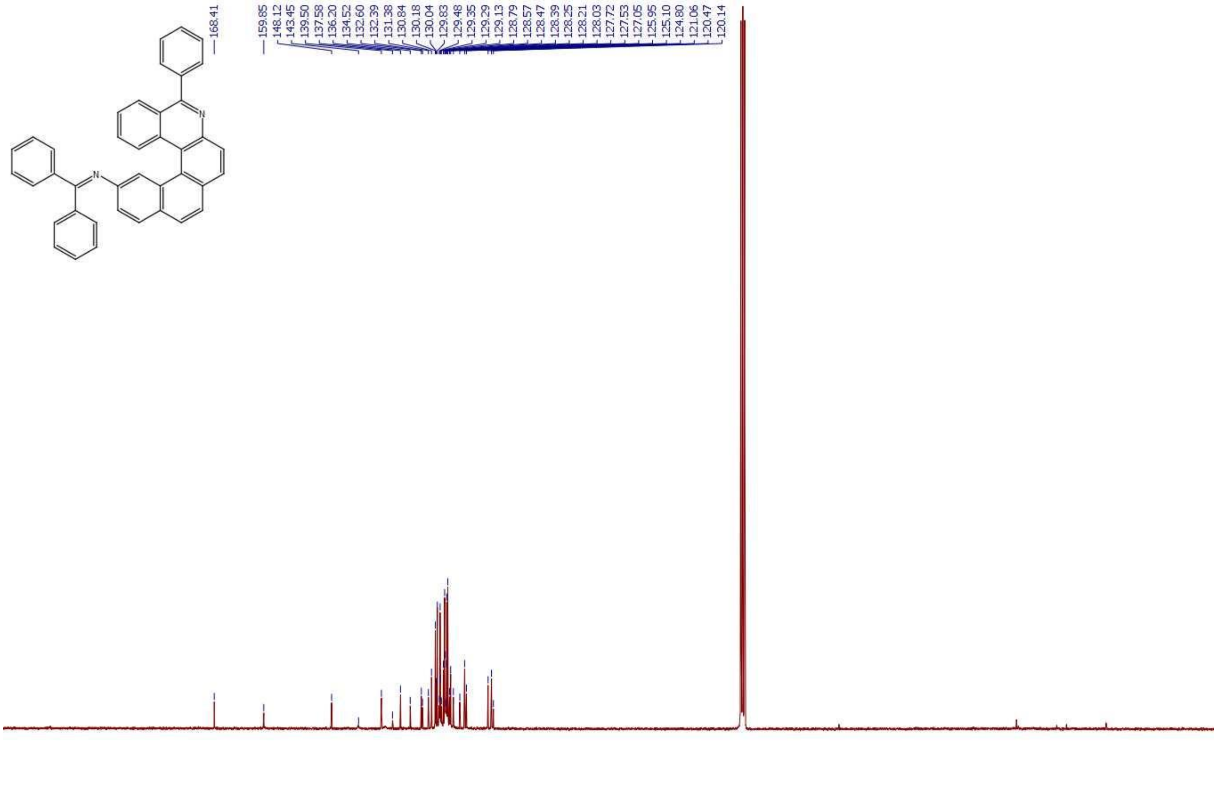

Figure S135. $^{13}C\{^{1}H\}$ NMR spectrum of compound **10b** (101 MHz, $CDCl_3$)
